# The cardiac METTL3/m6A pathway regulates the systemic response to Western diet

**DOI:** 10.1172/jci.insight.188414

**Published:** 2025-04-24

**Authors:** Charles Rabolli, Jacob Z. Longenecker, Isabel S. Naarmann-de Vries, Joan Serrano, Jennifer M. Petrosino, George A. Kyriazis, Christoph Dieterich, Federica Accornero

**Affiliations:** 1 Department of Physiology and Cell Biology, Dorothy M. Davis Heart and Lung Research Institute, The Ohio State University, Columbus, Ohio, USA.; 2 Department of Molecular Biology, Cell Biology and Biochemistry, Brown University, Providence, Rhode Island, USA.; 3 Section of Bioinformatics and Systems Cardiology, Klaus Tschira Institute for Integrative Computational Cardiology, and; 4 Department of Internal Medicine III (Cardiology, Angiology, and Pneumology), University Hospital Heidelberg, Heidelberg, Germany.; 5 German Centre for Cardiovascular Research (DZHK), Partner Site Heidelberg/Mannheim, Heidelberg, Germany.; 6 Department of Biological Chemistry and Pharmacology, College of Medicine, The Ohio State University, Columbus, Ohio, USA.

**Keywords:** Cardiology, Muscle biology, RNA processing

## Abstract

Regulation of organismal homeostasis in response to nutrient availability is a vital physiological process that involves interorgan communication. The role of the heart in controlling systemic metabolic health is not clear. Adopting a mouse model of diet-induced obesity, we found that the landscape of N6-methyladenosine (m6A) on cardiac mRNA was altered following high-fat/high-carbohydrate feeding (Western diet). m6A is a critical posttranscriptional regulator of gene expression, the formation of which is catalyzed by methyltransferase-like 3 (METTL3). Through parallel unbiased approaches of Nanopore sequencing, mass spectrometry, and protein array, we found regulation of circulating factors under the control of METTL3. Mice with cardiomyocyte-specific deletion of METTL3 showed a systemic inability to respond to nutritional challenge, thereby mitigating the detrimental effects of Western diet. Conversely, increasing cardiac METTL3 level exacerbated diet-induced body weight gain, adiposity, and glucose intolerance. Our findings position the heart at the center of systemic metabolism regulation and highlight an m6A-dependent pathway to be exploited for the battle against obesity.

## Abstract 2

This study shows how the heart regulates obesity onset

## Introduction

Animals regulate energy homeostasis through a multiorgan coordinated effort maintaining systemic metabolic rates. The past decades have been marked by a prevalence of high-fat, high-carbohydrate food intake (Western diet), leading to a dramatic increase in the prevalence of metabolic dysfunction and obesity in the human population (1). Obesity predisposes individuals to a litany of diseases, including type 2 diabetes mellitus, dyslipidemia, and hypertension, all of which are major risk factors for cardiovascular disease (2). Cardiovascular disease, the leading cause of morbidity and mortality in the world, accounts for more than two-thirds of the deaths of overweight individuals (3) and thus represents a major intersection between obesity and mortality. To mitigate the effects of increasing obesity prevalence on mortality, a better understanding of the crosstalk between the heart and obesogenic factors is necessary.

Interorgan communication through secreted molecules is a major mode of metabolic control at the organismal level. For instance, skeletal muscle releases circulating factors, which regulate energy homeostasis by acting on adipose and other tissue (4). Similarly, it is known that adipokines secreted by adipose tissue can interact with the heart to affect cardiovascular function (5). What is less clear is the role of the secretory molecules released by the heart in regulating the whole-body response to overnutrition, though decreased cardiac secretion of natriuretic peptides has been reported in obesity (6). Importantly, our group has previously discovered a link between the secretion of cardiac derived TGF-β1 and regulation of the whole-body response to diet-induced obesity (7). Given the strong connection between cardiac disease and metabolism, it stands to reason that the heart should be an integral player in communicating metabolic needs, yet how this communication occurs remains understudied.

Multiple gene expression steps can modulate the levels of circulating proteins. Dysregulation of transcription factors, such as peroxisome proliferator–activated receptors (PPARs), PPAR-γ coactivator 1α (PGC-1α), CCAAT/enhancer-binding protein, and others, has been identified as a contributor to energy imbalances through transcriptional gene control. Less well understood, however, is the contribution of posttranscriptional mechanisms that regulate metabolic functions. Over 150 chemical RNA modifications have been identified (8), with the most prevalent on mRNA being a methyl addition to the N6 position of adenosine, termed m6A (9). This reaction is catalyzed by methyltransferase-like 3 (METTL3). m6A can regulate nearly all steps of mRNA processing and can, therefore, have a profound effect on protein expression, even when gene transcription remains unchanged.

As a critical posttranscriptional level of control, m6A can affect metabolism as well. In white adipose tissue, m6A has been implicated in controlling adipogenesis (10) and in promoting browning (11). Furthermore, the m6A demethylase fat mass and obesity-associated protein has been linked to maintenance of whole-body adiposity (12). Moreover, decreased m6A abundance in the pancreas can drive insulin resistance (13), which promotes the onset of obesity. While it is straightforward that mutations in adipose or pancreatic tissue can affect systemic metabolism, it is unknown how alterations in the METTL3/m6A pathway in the heart affect metabolic homeostasis.

Here, we show that altering the METTL3/m6A pathway in cardiomyocytes dramatically affects systemic metabolism and organismal homeostasis. We present unbiased m6A profile remodeling in the heart following Western diet and find METTL3-mediated regulation of modified transcripts encoding secreted factors, thus positioning the heart as a pivotal secretory organ and metabolic modulator. Through parallel unbiased approaches of nanopore sequencing, mass spectrometry, and protein array, we find regulation of fibroblast growth factor 1 (FGF1) under the control of METTL3. Mice with cardiomyocyte-specific deletion of METTL3 show a systemic inability to respond to nutritional challenge, thereby mitigating the detrimental effects of Western diet. Conversely, increasing cardiac METTL3 levels exacerbates diet-induced body weight gain, adiposity, and glucose intolerance. Together, these data position the cardiac METTL3/m6A pathway as a component of organismal metabolic control.

## Results

### Western diet remodels the cardiac m6A profile.

**Figure 1:**
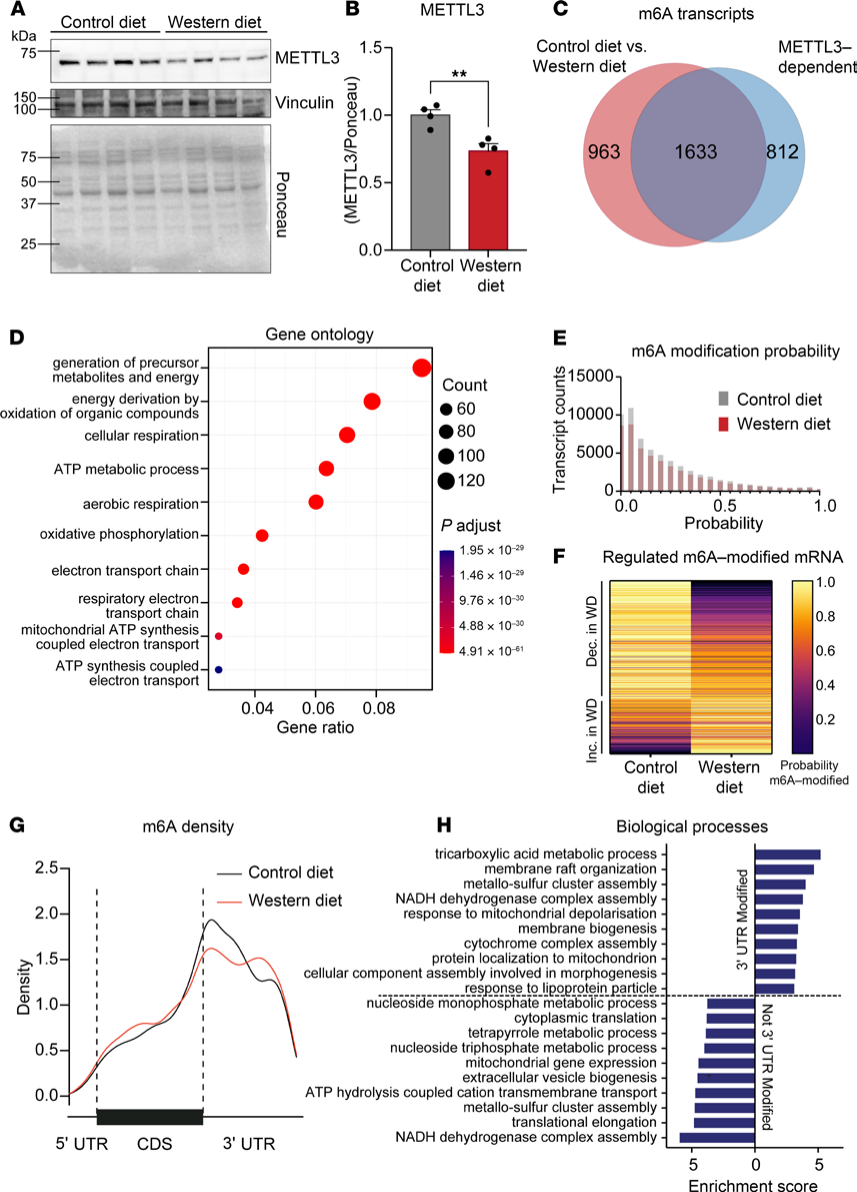
The m6A landscape is modified by Western diet. (**A** and **B**) Western blot and quantification of METTL3 expression following 2 weeks of Western diet on wild-type mice, normalized to total protein shown in Ponceau, relative to control diet. *n* = 4 per group. (**C**) Venn diagram showing the overlap of transcripts detected via Nanopore between different conditions. “Control diet vs. Western diet” refers to transcripts that are differentially m6A methylated when comparing wild-type hearts from mice on Western and control diets. “METTL3-dependent” refers to transcripts whose m6A sites are present in control hearts but lost in METTL3-knockout samples. (**D**) Gene Ontology analysis highlighting differentially expressed pathways on the overlapping 1,633 transcripts identified via Nanopore. (**E**) m6anet analysis of probability that a given transcript is modified at a specific site. (**F**) Heatmap showing the transcripts that have a differential modification probability of at least 10% between diet conditions. (**G**) Density plot showing the location of the m6A mark across all transcripts detected in control or Western diet. (**H**) Biological processes for transcripts that contain m6A in the 3′ UTR (top) and those that contain an m6A mark in a location other than the 3′ UTR (bottom). Data shown as mean ± SEM. Unpaired *t* test was used (**B**); **P < 0.01. CDS, coding sequence.

Analysis of METTL3-dependent m6A sites in cardiomyocytes hinted at a role for this pathway in cardiac cell metabolism. This prompted us to test if METTL3 and m6A in the heart are responsive and contributory to Western diet challenges. We found a reduction in the cardiac levels of METTL3 following 2 weeks of Western diet (Figure 1, A and B), a time point chosen to precede obesity onset and therefore avoid disease-derived confounding effects. We further assessed transcriptome-wide m6A sites in the hearts of mice fed a control or Western diet using direct RNA sequencing via Oxford Nanopore technology (14). We detected 1,633 high-fidelity m6A-modified mRNAs that were both METTL3 dependent and altered by Western diet (Figure 1C and Supplemental Table 1; supplemental material available online with this article; https://doi.org/10.1172/jci.insight.188414DS1). Gene ontology on these 1,633 transcripts identified pathways that regulate energy metabolism, emphasizing the impact of METTL3 on metabolic homeostasis (Figure 1D). Interestingly, m6anet probability analysis (15) showed fewer transcripts modified at all probability levels following Western diet (Figure 1E). When considering only transcripts whose modification probability differed by more than 10% in either direction because of diet, we found a higher number of transcripts showing decreased versus increased m6A probability under Western diet (Figure 1F), further verifying the effect of diet on the regulation of m6A methylation. We then analyzed m6A density to assess the location of the modification on cardiomyocyte mRNA and found that Western diet shifted the position of the m6A mark, with the most striking changes observed at the 3′ UTR (Figure 1G). When comparing the biological processes of the genes affected by Western diet with an m6A mark in the 3′ UTR versus those modified anywhere except the 3′ UTR, we found shared (i.e., metabolic processes) but also unique (i.e., membrane and translation regulation) pathways being responsive to Western diet in a location-specific manner (Figure 1H). These findings hint at a phenomenon by which, while the total abundance of m6A is still important, the relative location of the modification could add an additional layer of regulation.

### Cardiomyocyte-specific METTL3 deficiency prevents the systemic response to Western diet.

**Figure 2:**
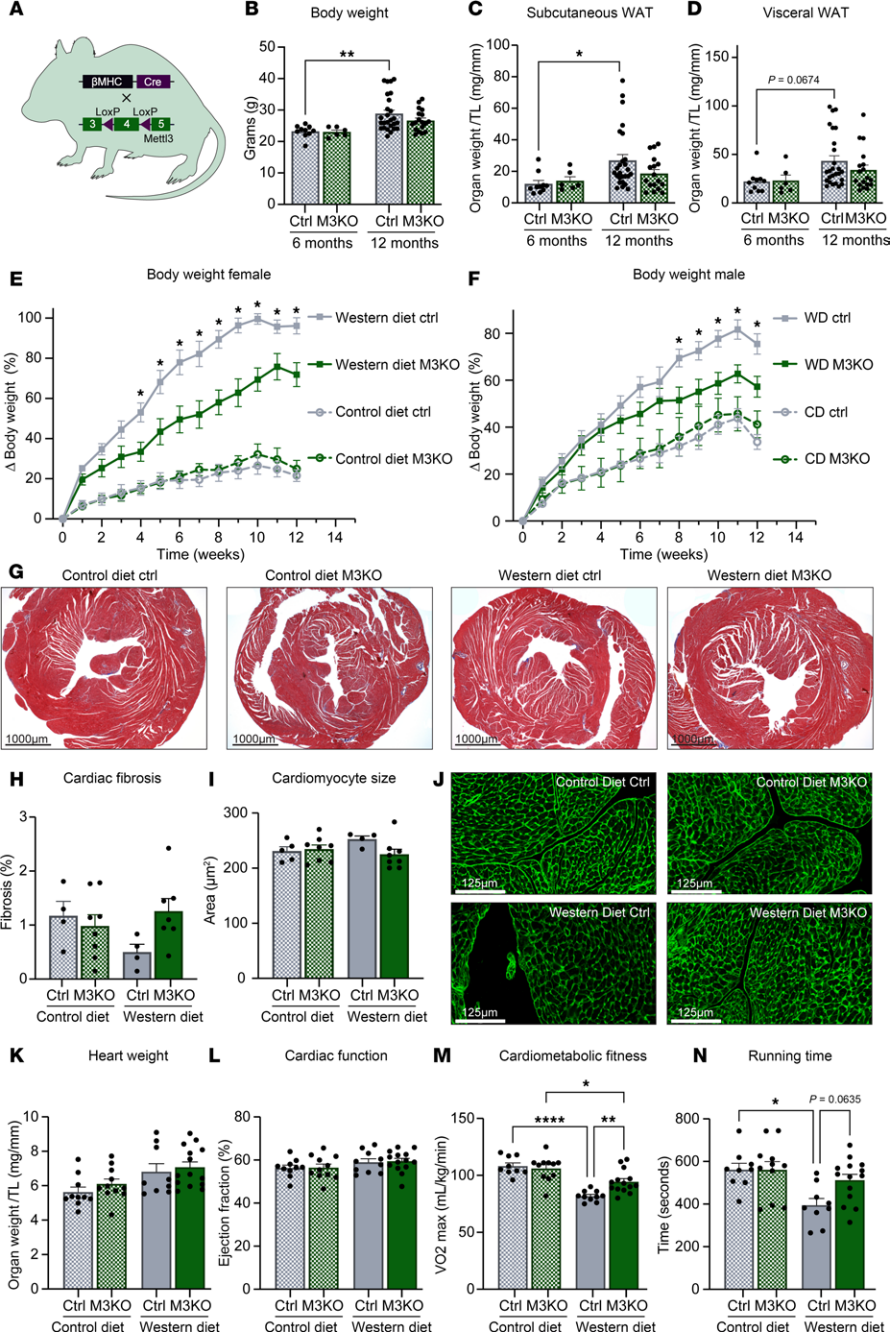
Cardiac M3KO mice have unchanged cardiac structure and function. (**A**) Schematic overview of the creation of METTL3 cardiomyocyte-specific knockout (M3KO). (**B**–**D**) Body weight and subcutaneous and visceral white adipose tissue (WAT) of control and M3KO mice at 6 and 12 months of age. *n* = (10; 6; 26; 17/18) (Ctrl 6 mo.; M3KO 6 mo.; Ctrl 12 mo.; M3KO 12 mo.). (**E** and **F**) Percentage increase of weekly body weights of female and male mice for 12 weeks following control or Western diet in control or M3KO animals. *n* (females) = (6; 8; 5; 8) and *n* (males) = (4; 3; 6; 6) (CD Ctrl; CD M3KO; WD Ctrl; WD M3KO). (**G** and **H**) Representative Masson’s trichrome–stained cardiac cross sections from the indicated groups and fibrosis quantification. *n* = (4; 8; 4; 7) (CD Ctrl; CD M3KO; WD Ctrl; WD M3KO). (**I** and **J**) Cardiomyocyte cross-sectional area determined by wheat germ agglutinin (WGA) staining. Scale bar = 125 μm. *n* = (5; 8; 4; 8) (CD Ctrl; CD M3KO; WD Ctrl; WD M3KO). (**K**) Heart weight normalized to tibia length after 12 weeks on control diet or Western diet. *n* = (10; 11; 9; 14) (CD Ctrl; CD M3KO; WD Ctrl; WD M3KO). (**L**) Cardiac ejection fraction measured via echocardiography after 12 weeks of diet. *n* = (10; 11; 10; 14) (CD Ctrl; CD M3KO; WD Ctrl; WD M3KO). (**M** and **N**) Metabolic treadmill test for maximal oxygen consumption (VO_2_ max) and running time until exhaustion determined via a graded exercise protocol. *n* = (9; 11; 9/10; 14) (CD Ctrl; CD M3KO; WD Ctrl; WD M3KO). Data shown as mean ± SEM. Two-way ANOVA with multiple comparisons test were used (**B**–**F** and **H**–**N**); *P < 0.05, **P < 0.01, ****P < 0.0001. βMHC, β–myosin heavy chain.

To assess if manipulation of the METTL3/m6A pathway in the heart affects metabolic homeostasis, we utilized a mouse model of cardiomyocyte-specific METTL3 knockout (M3KO) (16) (Figure 2A). We noticed that cardiac METTL3 deficiency blunted the naturally occurring, age-induced increase in body weight and white adipose tissue mass that occurs between 6 months and 12 months of age in control mice (Figure 2, B–D). These initial observations led us to investigate whether cardiomyocyte regulation of METTL3 could indeed affect global metabolism. We then subjected 3-month-old M3KO and littermate controls to 12 weeks of Western or control diet. The timeline of these experiments was chosen to avoid potential confounding factors derived by cardiac baseline phenotypes that develop in METTL3-mutant mice at 8 months of age (16). Strikingly, mice lacking METTL3 in cardiomyocytes gained significantly less weight on Western diet compared with controls (Figure 2, E and F). It is important to note that we verified the absence of significant changes in cardiac structure (no histopathology or cardiomyocyte size differences) or function (measured by echocardiography) in the tested conditions (Figure 2, G–L, and Supplemental Table 2). However, when assessed for cardiometabolic fitness on a treadmill protocol, M3KO mice outperformed their control littermates under Western diet as indicated by the higher maximal oxygen consumption and preserved time to exhaustion (Figure 2, M and N). Together, cardiomyocyte-specific lack of METTL3 led to diet-responsive phenotypes independent from cardiac remodeling.

**Figure 3:**
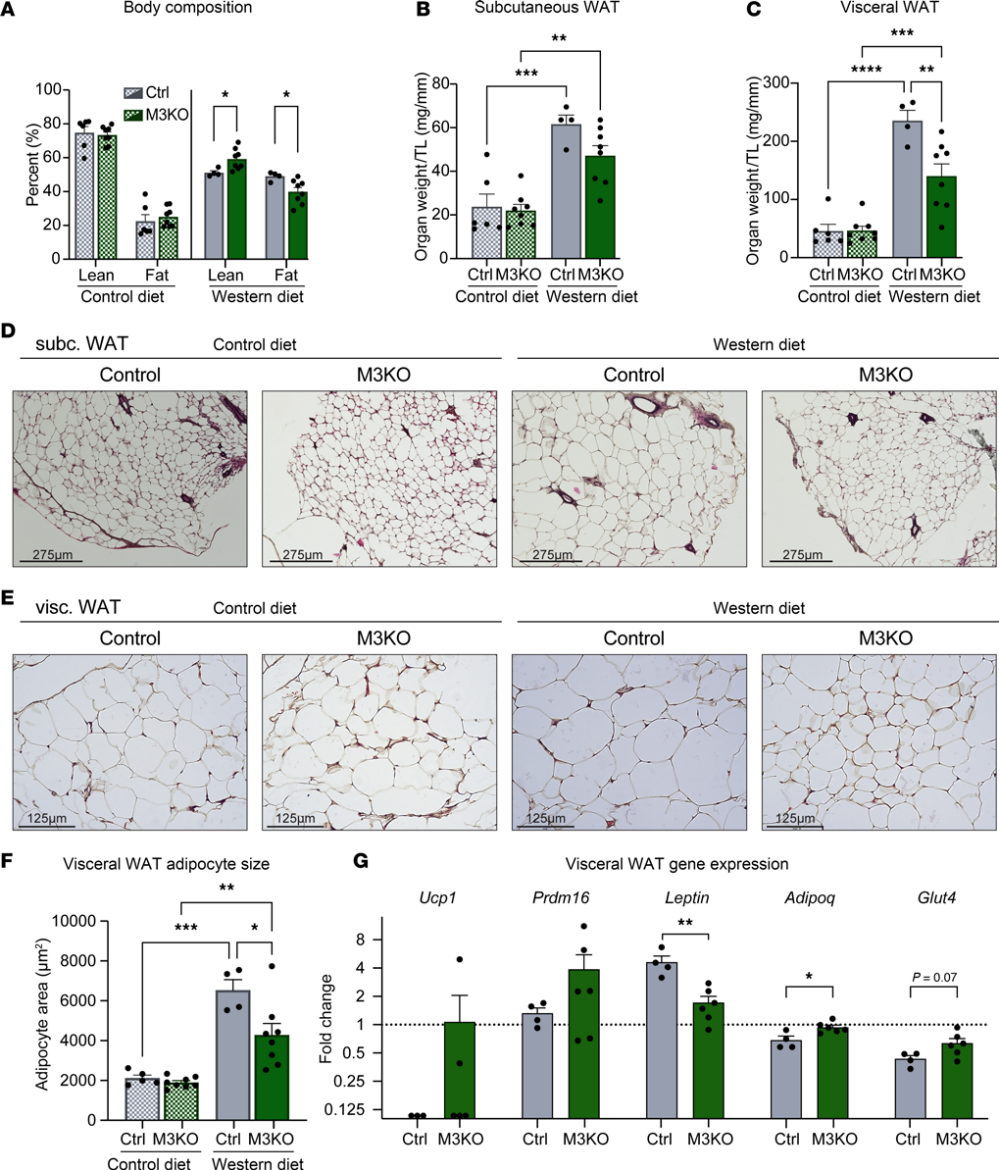
White adipose tissue depots are affected by cardiac M3KO. (**A**) EchoMRI detection of body composition after 12 weeks on either control diet or Western diet. Control diet *n* = (6; 8; 6; 8), Western diet *n* = (4; 8; 4; 8) (Lean Ctrl; Lean M3KO; Fat Ctrl; Fat M3KO). (**B**) Subcutaneous WAT and (**C**) visceral WAT weight after 12 weeks on either control diet or Western diet, normalized to tibia length. *n* = (6; 8; 4; 8) (CD Ctrl; CD M3KO; WD Ctrl; WD M3KO). Representative H&E images from (**D**) subcutaneous WAT (scale = 275 μm) and (**E**) visceral WAT (scale = 125 μm). (**F**) Quantification of visceral WAT adipocyte size through ImageJ (NIH) based on histological section analysis. (**G**) Real-time quantitative PCR (qPCR) analysis of the indicated genes on visceral WAT. Gene expression normalized to *Rpl7*. Data normalized to expression of control animals on control diet (dashed line). Data shown as mean ± SEM. Two-way ANOVA with multiple comparisons test (**A**–**C** and **F**) and unpaired *t* tests (**G**) were used. *P < 0.05, **P < 0.01, ***P < 0.001, ****P < 0.0001. AdipoQ, adiponectin, C1Q and collagen domain containing; Glut4, solute carrier family 2.

We then asked if the detrimental effects of Western diet on body composition were mitigated by cardiac METTL3 deficiency and found that with Western diet feeding M3KO mice better preserved the lean/fat mass ratio compared with control mice (Figure 3A). Specifically, M3KO mice showed a blunted increase in white adipose tissue, which was significant for the visceral depot, when placed on a Western diet (Figure 3, B and C). Histological analysis of subcutaneous and visceral white adipose tissues revealed preserved architecture, fewer and smaller lipid droplets, and decreased adipocyte size in M3KO mice on a Western diet (Figure 3, D–F). Molecular analysis of white adipose tissue samples revealed no significant change in the expression of markers of browning, such as uncoupling protein 1 (*Ucp1*) and PR domain containing 16 (*Prdm16*), despite the observed higher average level in M3KO mice upon Western diet (Figure 3G). On the other hand, expression of *Leptin* and *AdipoQ*, known to increase and decrease, respectively, under Western diet stress, revealed resistance to pathological changes in M3KO samples (Figure 3G). A trending preservation of *Glut4* expression was also present (Figure 3G).

**Figure 4:**
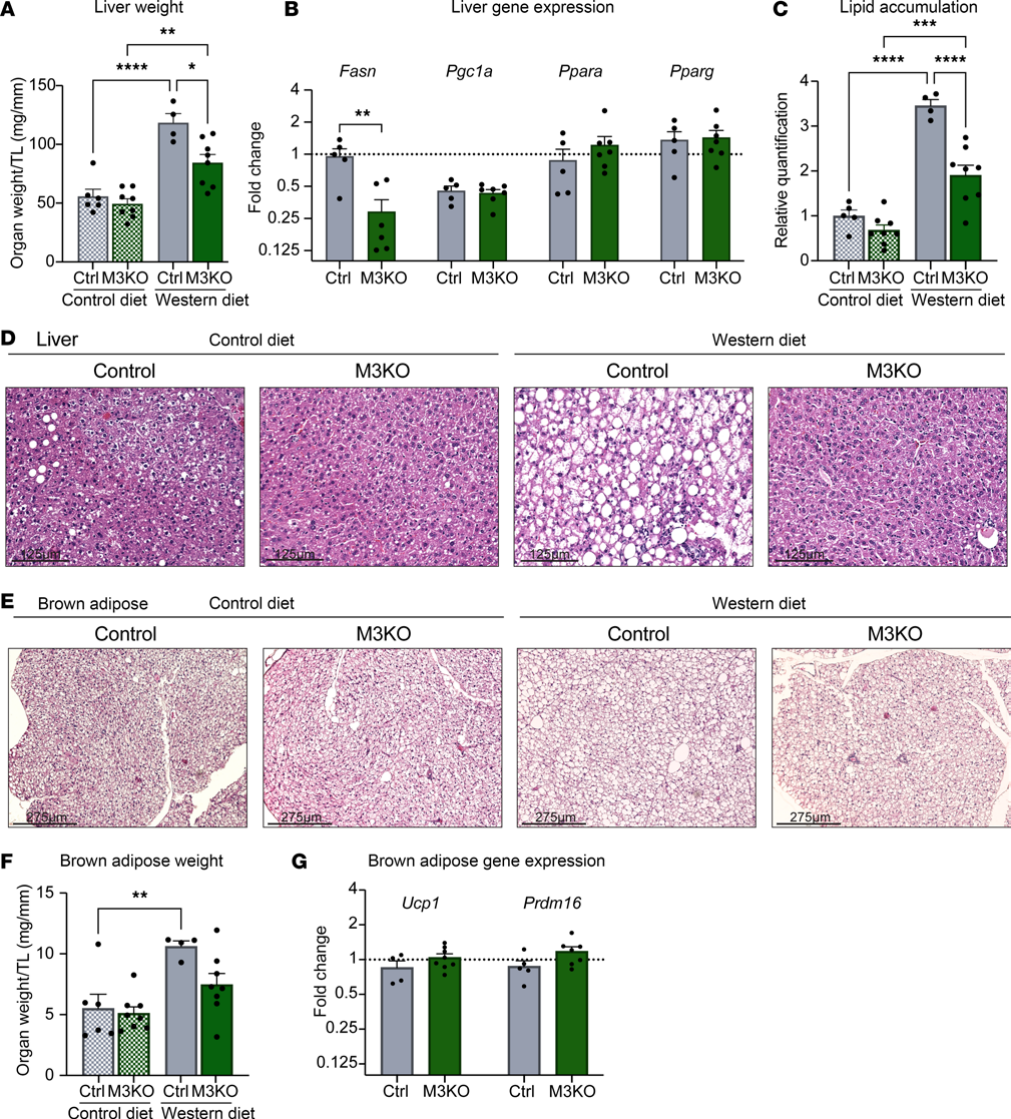
Liver and brown adipose remodeling following loss of METTL3. (**A**) Liver weight after 12 weeks on either control diet or Western diet, normalized to tibia length. *n* = (6; 8; 4; 8) (CD Ctrl; CD M3KO; WD Ctrl; WD M3KO). (**B**) qPCR analysis of the indicated genes on liver. Gene expression normalized to *Rpl7*. Data normalized to expression of control animals on control diet (dashed line). (**C** and **D**) Lipid accumulation quantification and representative H&E images from liver (scale = 125 μm). (**E**) Representative H&E images from brown adipose tissue (BAT) (scale = 275 μm). (**F**) Brown adipose weight after 12 weeks on either control diet or Western diet, normalized to tibia length. *n* = (6; 8; 4; 8) (CD Ctrl; CD M3KO; WD Ctrl; WD M3KO). (**G**) qPCR analysis of the indicated genes on BAT. Gene expression normalized to *Rpl7*. Data normalized to expression of control animals on control diet (dashed line). Data shown as mean ± SEM. Two-way ANOVA with multiple comparisons test (**A**, **C**, and **F**) and unpaired *t* tests (**B** and **G**) were used. *P < 0.05, **P < 0.01, ***P < 0.001, ****P < 0.0001.

Next, we checked if other organs known to play a central role in the regulation of systemic metabolism were affected by the loss of cardiac METTL3. We found decreased liver weight in M3KO mice fed a Western diet (Figure 4A), which was accompanied by decreased expression of fatty acid synthase (*Fasn*) and lower lipid accumulation (Figure 4, B–D). We subsequently analyzed brown adipose tissue (Figure 4, E–G), which increased in weight only in control mice (Figure 4F), without showing a significant difference in *Ucp1* and *Prdm16* expression between genotypes (Figure 4G).

**Figure 5:**
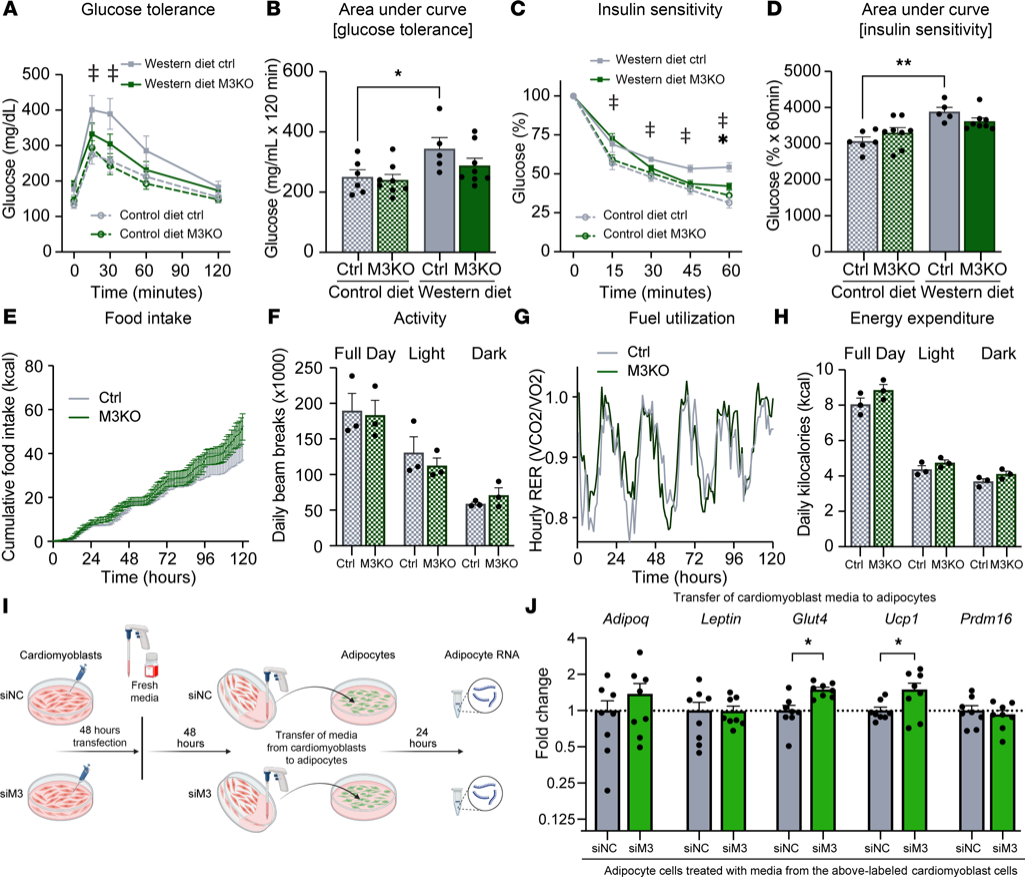
M3KO mice have no behavioral differences. (**A** and **B**) Glucose tolerance test (GTT) and (**C** and **D**) insulin sensitivity test (ITT) after 12 weeks on control or Western diet. *n* = (6; 8; 5; 8) (CD Ctrl; CD M3KO; WD Ctrl; WD M3KO). (**E**) Food consumption, (**F**) activity, (**G**) fuel utilization, and (**H**) energy expenditure collected via metabolic cages over 5 days from animals on control diet. *n* = (3; 3) (Ctrl; M3KO). (**I**) Schematic overview of conditioned media experiment. Figure created using BioRender.com. (**J**) qPCR analysis of the indicated genes on 3T3-L1 cells. Gene expression normalized to *Rpl7*. Data normalized to expression of cells conditioned with media from siRNA negative control siRNA–treated (siNC-treated) cardiomyoblasts. *n* = 8 per group. In **A** and **C**, ^‡^ represents significant (*P* ≤ 0.05) difference between Western diet Ctrl and control diet Ctrl; * represents significant (*P* ≤ 0.05) difference between Western diet Ctrl and Western diet M3KO. Data shown as mean ± SEM. Two-way ANOVA with multiple comparisons test (**A**–**D**, **F**, and **H**) and multiple unpaired *t* tests (**E**, **G**, and **J**) were used. *P < 0.05, **P < 0.01. RER, respiratory exchange ratio; siM3, siRNA against METTL3.

Upon examining global metabolic function, we observed that M3KO mice had preserved glucose tolerance (Figure 5, A and B) and insulin sensitivity (Figure 5, C and D) following Western diet challenge. Notably, these findings could not be explained by innate differences in food intake (Figure 5E), daily activity (Figure 5F), fuel utilization (Figure 5G), or energy expenditure (Figure 5H). These pertinent negatives emphasize that the discovered phenotypic differences are likely not due to behavioral changes. Collectively, as the only genotype-specific differences in these mice occurred within cardiomyocytes, yet the phenotypic differences occurred systemically, the results suggest that cardiac METTL3 might regulate the secretion of factors from the heart. Indeed, media transfer from cardiomyoblasts knocked down for METTL3 to 3T3-L1 adipocytes induced molecular alterations that reached significance for *Glut4* and *Ucp1* expression (Figure 5, I and J).

### FGF1 is a putative m6A target affected by cardiomyocyte METTL3.

**Figure 6:**
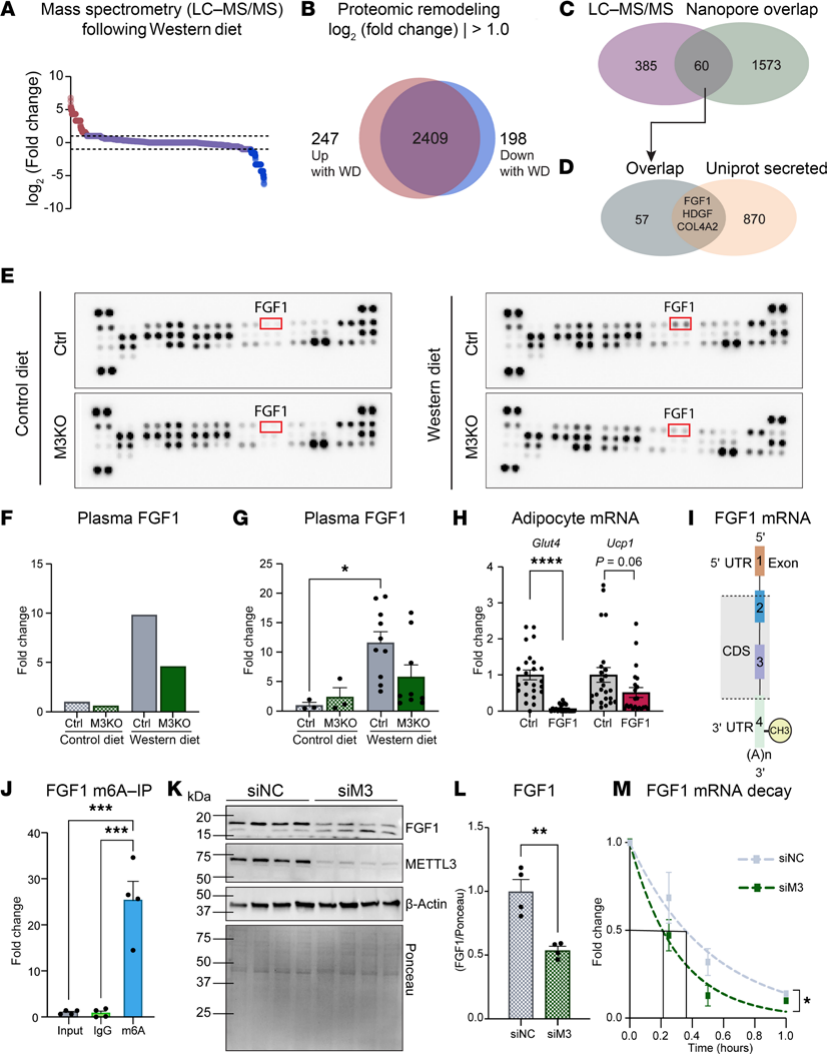
Loss of METTL3 alters FGF1 levels. (**A**) Proteomic changes in the heart following 2 weeks of Western diet. (**B**) Venn diagram showing 247 and 198 proteins were increased and decreased, respectively, by Western diet with log2(fold-change) greater than 1.0 or less than –1.0. *n* = 3 pooled samples per condition. (**C**) Overlap between 445 differentially expressed proteins detected by mass spectrometry and 1,633 transcripts identified by Nanopore. (**D**) Overlap between 60 transcript/protein targets and 873 proteins annotated as secreted on Uniprot. (**E** and **F**) Adipokine arrays and quantification of plasma pooled from *n* = 3 (CD Ctrl and M3KO) or *n* = 4 (WD Ctrl and M3KO) mice after 2 weeks on diet. (**G**) FGF1 ELISA on plasma isolated from mice after 2 weeks on diet. *n* = (3; 3; 10; 9) (CD Ctrl; CD M3KO; WD Ctrl; WD M3KO). (**H**) qPCR on 3T3-L1 adipocytes following FGF1 or control treatment. Gene expression normalized to *Rpl7*. (**I**) Schematic of Fgf1 showing location of m6A methylation upon Western diet in the 3’ UTR. (**J**) m6A-immunoprecipitation to identify presence of m6A on transcripts, normalized to input. Input and IgG used as controls. *n* = 4 per group. (**K** and **L**) Western blot from H9C2 cardiomyoblasts transfected with small interfering negative control (siNC) or small interfering against METTL3 (siM3). FGF1 expression normalized to Ponceau. *n* = 4 per group. (**M**) *Fgf1* mRNA decay in H9C2 cells following the transcription inhibitor actinomycin D for the indicated time. *n* = 5 per group. Data shown as mean ± SEM. Two-way ANOVA with multiple comparisons test (**G**), unpaired *t* test (**H** and **L**), 1-way ANOVA with multiple comparisons test (**J**), and nonlinear fit with least squared regression and extra sum-of-squares *F* test (**M**) were used. *P < 0.05, **P < 0.01, ***P < 0.001, ****P < 0.0001.

To investigate the mechanistic targets contributing to the observed phenotypes, we first searched for Western diet–responsive proteins by performing mass spectrometry on mouse hearts after 2 weeks of control or Western diet (Figure 6A and Supplemental Table 3). We identified 445 proteins regulated by Western diet (Figure 6B). To narrow our search, we investigated overlapping protein/RNA pairs between the 445 differential proteins identified by mass spectrometry and the 1,633 METTL3-dependent/diet-responsive m6A-modified transcripts identified via Oxford Nanopore and discovered 60 targets of interest (Figure 6C). This key information informs on cardiac transcripts that are posttranscriptionally modified and altered in protein abundance following Western diet. We then looked more specifically into factors predicted to reach circulation and therefore with potential to affect systemic processes by comparing the 60 identified targets with the Uniprot database list of 873 canonically secreted proteins. This analysis highlighted FGF1, HDGF, and COL4A2 (Figure 6D), with COL4A2 being less likely to affect distant organs, since it is an extracellular matrix structural protein that does not typically circulate.

In parallel, we performed a protein array of canonical obesogenic factors using plasma taken from M3KO and control mice following 2 weeks of Western diet to discover which secreted proteins may be altered in circulation prior to phenotypic differences. Of all tested factors, FGF1 dramatically stood out as being regulated (Figure 6, E and F, and Supplemental Figure 1). Notably, we found that cardiac deletion of METTL3 blunted the Western diet–induced increase in circulating levels of FGF1 (Figure 6, E and F), which was verified by ELISA (Figure 6G). To investigate whether FGF1 could affect adipocyte homeostasis, we assessed cultured 3T3-L1 cell responsiveness to FGF1. We found that FGF1 was sufficient to decrease *Glut4* expression in these cells, where a trend in suppressing *Ucp1* expression was also observed (Figure 6H). This is interesting in relation to the fact that those markers were also markedly affected in M3KO mice, which presented with lower circulating FGF1 levels. Given these findings pointing at the importance of FGF1 in our model, we further investigated this factor. Using our Nanopore sequencing dataset we analyzed the location of the m6A site in *Fgf1* mRNA, and we found that, upon Western diet, *Fgf1* was modified only in the 3′ UTR (Figure 6I). To validate *Fgf1* as being m6A modified and thus a likely direct target of METTL3, we performed an m6A-immunoprecipitation (m6A-IP) and verified that *Fgf1* mRNA indeed contained m6A (Figure 6J). To verify the direct role of METTL3 in regulating FGF1 expression, we then adopted a simplified cardiomyoblast culture system. We verified protein downregulation of FGF1 upon acute METTL3 knockdown (Figure 6, K and L) as well as in M3KO mouse hearts (Supplemental Figure 2). Mechanistically, we found a more rapid decay of *Fgf1* transcripts when METTL3 was lost (Figure 6M). To understand which RNA binding protein could be responsible for this effect, we immunoprecipitated the YTH domain-containing family proteins, which are a family of m6A interactors, and found enrichment for *Fgf1* transcript binding in all 3 members of this family (Supplemental Figure 3). Collectively, our data hint at the importance of m6A formation in the 3′ UTR of *Fgf1* by METTL3 for the control of circulating FGF1 levels, suggesting regulation of factors implicated in diet-induced obesity.

### Mice overexpressing METTL3 in the heart exhibit exacerbated responses to Western diet.

**Figure 7:**
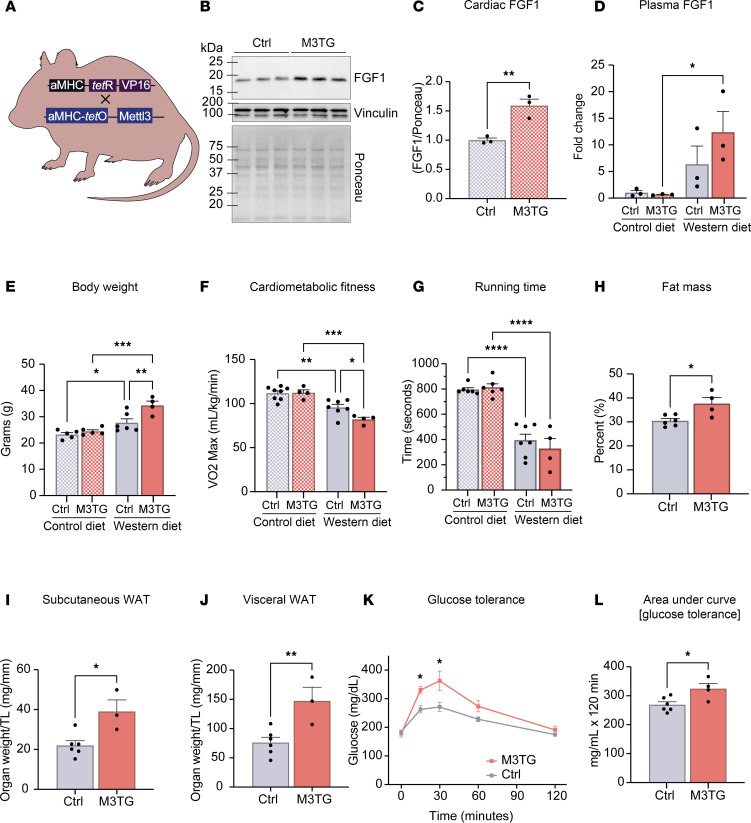
METTL3 overexpression exhibits the opposite phenotype from M3KO. (**A**) Schematic overview of the creation of METTL3 cardiomyocyte-specific overexpressing mice (M3TG). (**B** and **C**) Western blot and quantification of cardiac FGF1 expression in mice overexpressing METTL3 in cardiomyocytes (M3TG) compared with littermate controls normalized to total protein (Ponceau). *n* = 3 per group. (**D**) FGF1 ELISA on plasma isolated from mice after 2 weeks on control or Western diet. *n* = 3 per group. (**E**) Body weight after 12 weeks on control or Western diet. *n* = (5; 5; 6; 4) (CD Ctrl; CD M3TG; WD Ctrl; WD M3TG). (**F** and **G**) Maximal oxygen consumption (VO_2_ max) and total running time determined via a graded running protocol after 12 weeks on control or Western diet. *n* = (8; 4; 7; 4) (CD Ctrl; CD M3TG; WD Ctrl; WD M3TG). (**H**) Fat mass after 12 weeks of Western diet, measured via EchoMRI. *n* = (6; 4) (Ctrl; M3TG). (**I** and **J**) Subcutaneous and visceral WAT weights normalized to tibia length, after 12 weeks of Western diet. *n* = (6; 3) (Ctrl; M3TG). (**K** and **L**) GTT after 12 weeks on control or Western diet. *n* = (6; 4) (Ctrl; M3TG). Data shown as mean ± SEM. Unpaired *t* test (**C**, **H**–**J**, and **L**) and 2-way ANOVA with multiple comparisons tests (**D**–**G**) were used. *P < 0.05, **P < 0.01, ***P < 0.001, ****P < 0.0001. aMHC, α–myosin heavy chain.

We finally sought to investigate whether overexpression of METTL3 would cause metabolic defects. To test this, we used mice where METTL3 is specifically overexpressed in cardiomyocytes (16) (M3TG) (Figure 7A) and found that increasing METTL3 was sufficient to drive higher FGF1 expression in the heart (Figure 7, B and C). We also detected elevated levels of circulating FGF1 in plasma of METTL3-transgenic mice after 2 weeks of Western diet (Figure 7D). We then tested diet-induced obesity onset in these mice. After 12 weeks of Western diet, M3TG animals exhibited increased body weight (Figure 7E) and worsened cardiometabolic fitness (Figure 7F) compared with controls, which was independent from running time (Figure 7G). Consistently, M3TG mice had increased fat mass (Figure 7H) and white adipose tissue mass (Figure 7, I and J) compared with littermate controls. Overexpression of METTL3 in cardiomyocytes also led to exacerbated glucose intolerance by Western diet (Figure 7, K and L), further showing the systemic effects of alterations in cardiac METTL3. Together, our data highlight the connection between the METTL3/m6A pathway in cardiomyocytes, regulation of FGF1 levels, and modulation of systemic metabolism, overall affecting diet-induced obesity.

## Discussion

Interorgan crosstalk as a homeostatic mechanism for maintenance of organismal health is a growing field of interest. Although adipose tissue and skeletal muscle have garnered much attention as metabolic sensory and secretory organs, the cardiac secretome remains understudied (17). Many times, studies that seek to investigate the interplay between the heart and adipose tissue focus on how systemic metabolism can affect the heart, but few ask the converse question: Can the heart affect systemic metabolism? For those that do, a strong emphasis is typically placed on transcriptomic analyses, but these focuses can be problematic, as they fail to capture the complete effects on the proteome; transcripts that are unchanged in abundance, but could still be posttranscriptionally regulated, remain hidden.

In this study, we asked, “Is m6A central for the heart’s ability to sense and respond to metabolic stress?” and we discovered that the answer is “Yes, and it can coordinate the response of other organs as well.” We performed unbiased, direct RNA sequencing on cardiac tissue to identify high-fidelity, METTL3-dependent transcripts that are altered by Western diet. Transcripts encoding secreted factors revealed key m6A regulation with consequences on their ability to act as a heart-derived communication tool that coordinates metabolic interaction between organs. The shift in the location of the m6A site is also intriguing, and it might suggest a currently unknown regulatory mechanism that dictates differential sequence targeting by METTL3. Perhaps the most striking result of this study is the extent of the pathophysiological systemic effect that altering METTL3 levels specifically in cardiomyocytes had under Western diet stress. This is even more intriguing after verifying the absence of an overt cardiac phenotype in the tested conditions, therefore minimizing the chance that this finding is a consequence of maladaptive cardiac function.

Discovering that cardiomyocyte-specific alterations in METTL3 can have such dramatic effects on distant organs was astounding and perhaps raises more questions than it answers. Here we propose regulation of FGF1 as a contributor to the observed modulation of systemic metabolism, but we recognize the likelihood that multiple factors are at play to achieve such multiorgan effects. Indeed, METTL3 can target close to 20% of the transcriptome, and work from our group shows it also has an impact on the expression of intracellular proteins, including mitochondrial metabolism regulators (18). It is interesting to note that in addition to stress-dependent changes in the level of m6A on mRNA targets, location of the modification within a transcript can be an important regulatory mechanism, and it is interesting to see that in our dataset there is a global shift toward m6A at the 3′ UTR of cardiac transcripts following Western diet feeding. In general, the concept of the heart sensing a metabolic stressor and responding by altering RNA modifications with consequences on the entire organism advances our knowledge on interorgan crosstalks.

Interesting work on FGF1 has been conducted by Jonker and colleagues, where they find FGF1 is a target of PPAR-γ and is induced in white adipose tissue during high-fat diet (19, 20). The directions of the changes in FGF1 that we have identified match those Jonker et al. reported where lower levels of FGF1 in the METTL3-deficient mice correlate with protection from diet-induced obesity, and higher levels of FGF1 in mice overexpressing METTL3 lead to exacerbated responses to diet stress. Of particular note, Jonker et al. discovered multiple mRNA isoforms of *Fgf1*, with the isoform *Fgf1A* being the only variant that increases in response to high-fat diet stress. This group further shows that of all the tissues they investigated, which includes white adipose tissue, *Fgf1A* had the highest expression in cardiac tissue. It is important to note, however, that Jonker (20) and others (21, 22) suggest a protective role for FGF1, specifically on insulin sensitivity and glucose tolerance, whereas in our model mice with lower circulating FGF1 levels are protected from Western diet–driven maladaptations. There are key methodological differences between our study and others, which could help rectify these discrepancies. Namely, Jonker et al. used a global FGF1-KO model and a different mouse strain, whereas we are most likely only affecting cardiac derived FGF1 through modulation of cardiomyocyte METTL3 levels. Additionally, other studies deliver recombinant FGF1 subcutaneously to investigate endocrinization, which is yet another methodological difference (21). In vitro, it has been shown that treatment of preadipocytes with FGF1 can stimulate differentiation and maturation (23), which is similar to our findings of worsened adipocyte homeostasis following FGF1 treatment in 3T3-L1 cells. Despite the differences in experimental design, the diversity of results and conclusions between studies highlights the necessity for further investigation into the mechanistic role of FGF1, in particular the different isoforms. Our study adds on to these previous findings by contributing that m6A methylation may be a crucial regulator of *Fgf1* and that expression of FGF1 by the heart may serve an important purpose in systemic metabolism. Although not the focal point of their study, another group using m6A-IP sequencing identified differential methylation in the 3′ UTR of *Fgf1* in murine hippocampus following a psychological stress model (24), giving further confidence in our findings.

Although FGF1 does appear to play an important role, it is unrealistic to think that the entire METTL3-dependent differential phenotype is due to FGF1 alone. Without having a conditional FGF1 deletion model in hand, it is also not possible to know how much the heart contributes to total circulating FGF1 level in either healthy or pathological conditions. Another limitation of the study is that, considering the role of METTL3 and m6A in regulating mRNA stability and translation, this study focuses on these molecular aspects, and we cannot exclude that manipulation of METTL3 also leads to protein secretion problems. Furthermore, previous work from our group demonstrated a mitochondrial defect in METTL3-deficient hearts (18). Whether a change in cardiac substrate utilization could contribute to the obesity phenotype is unclear. Similarly, we cannot exclude that an alteration in cardiomyocytes’ mitochondrial function could lead to changes in circulating metabolites or lipids contributing to interorgan crosstalk. Nevertheless, proof-of-concept validation through multiple unbiased methods showing *Fgf1* as a METTL3 target is likely an important piece of a much bigger puzzle.

As the prevalence of obesity, metabolic syndrome, and cardiovascular disease continue to rise, improved understanding of the crosstalk between these diseases is necessary. If we hope to solve the challenges of multifactorial diseases, we need to study them in multifactorial conditions, rather than in isolation. While we recognize the complexity of interacting systems in animals and that much more work is needed to comprehensively understand the full extent of interorgan communication, our study investigates the previously unknown systemic effects of cardiac deletion of METTL3 and opens the door to understanding the role of the cardiac secretome in the background of diet-induced obesity.

## Methods

### Sex as a biological variable.

Both male and female mice were used in this study. Sex was considered as a biological variable and findings are expected to apply to both sexes (see Figure 2, E and F).

### Mouse line generation and handling.

Mouse models for METTL3 gain and loss of function were previously generated and characterized by our group (16). In brief, for overexpression of METTL3 specifically in cardiomyocytes, mice containing a METTL3 transgene driven by a doxycycline/tetracycline-responsive α-MHC promoter were crossed with mice expressing the tetracycline transactivator protein (tTA) driven by the α-MHC promoter. The resulting METTL3-overexpressing mice (M3TG) and controls (tTA) were generated in the FVB/N background. For cardiomyocyte-specific knockout of METTL3 (M3KO), mice expressing METTL3 with exon 4 flanked by flox sequences were crossed with mice expressing a cre recombinase transgene driven by the β-MHC promoter. M3KO mice and controls (Mettl3^fl/fl^) were generated in the C57BL/6J background. Three-month-old male and female mice were fed a Western diet (Envigo TD.88137, 42% kcal fat/42.7% kcal carbohydrate/15.2% kcal protein) or an ingredient-matched control diet (Envigo TD.08485, 13% kcal fat/67.9% kcal carbohydrate/19.1% kcal protein) for either 2 or 12 weeks. FVB/N and C57BL/6J mice were obtained from The Jackson Laboratory (strain 001800) and (strain 000664).

### Nanopore sequencing and bioinformatics.

Nanopore sequencing of mRNA was performed, and the resulting BAM files were subjected to analysis for the detection of m6A sites using the JACUSA2 software (25). High-fidelity targets were chosen for further analysis based on Nanopore score. Peak and modification site analyses were performed with RNAmod (26) by inputting .bed files produced by JACUSA2. Analysis was done using the general default settings supplied by RNAmod but changing the motif size to 4,5,6 to reflect canonical m6A motif size. Gene Ontology was performed using the clusterProfiler package (27) in R (Version 4.1.2), using Benjamini-Hochberg method to correct for FDR. Significance level was considered at FDR < 0.05.

### m6anet.

We predicted the site/residue-specific modification status using m6anet v2.0.1 (15). Briefly, we followed the instructions as outlined on https://m6anet.readthedocs.io/en/latest/ without retraining and using the EnsEMBL v102 mouse transcriptome as reference. The initial transcriptome-wide mapping was performed with minimap v2.22 (--MD --secondary=no -ax map-ont -k5).

### Western blotting.

Protein extracts from whole hearts were generated using RIPA buffer (150 mM NaCl, 1% NP-40, 0.5% sodium deoxycholate, 0.1% SDS, 25 mM Tris pH 7.4) supplemented with EDTA-free protease (Roche, 11873580001) and phosphatase (MilliporeSigma 524624, 524625) cocktails. Tissues were snap-frozen in liquid nitrogen and cryopulverized (Cole Parmer Tissue Pulverizer, 40355), then sonicated via UCD-500 Bioruptor XL for 10 minutes (30 seconds × 320 W, 30 seconds off). Samples were centrifuged (4°C × 21,130 rcf × 20 minutes) and quantified using Pierce BCA Protein Assay Kit (Thermo Fisher Scientific, 23225). Standard Western blotting analysis was performed with the following primary antibodies: METTL3 (Abcam, ab195352, 1:1,000), FGF1 (R&D Systems, Bio-Techne; AF4686, 1:1,000), vinculin (Novus Biologicals, Bio-Techne; NB600-1293, 1:1,000), and β-actin (Cell Signaling Technology, 3700, 1:1,000). Membranes were incubated with HRP-conjugated secondary antibodies for 90 minutes at room temperature (22°C) and imaged via ChemiDoc TOUCH Imaging System (Bio-Rad). Individual band intensity was quantified using ImageJ 1.53k, whereby intensity was normalized to the integrated density of total protein loaded, as detected by Ponceau S (Acid Red 112).

### Mass spectrometry–based proteomics analysis.

Samples were prepared to enrich for soluble proteins as previously described (28). Briefly, mouse hearts were minced, washed with PBS, and centrifuged (1 minute × 50*g*), and the supernatant was discarded. Tissue was resuspended in buffer (0.5 M NaCl, 10 mM Tris base pH 7.5), and samples were set on a shaker at 4°C overnight. The following day, proteinaceous supernatant was isolated and stored at –80°C. Protein concentration was quantified via Pierce BCA Protein Assay Kit. Protein quality was assessed via silver stain, and 100 μg of extracted protein was submitted to The Ohio State University Campus Chemical Instrument Center Mass Spectrometry and Proteomics Facility for label-free mass spectrometry analysis.

### Echocardiography.

Echocardiographic measurements were taken using a VisualSonics Vevo 3100 system and MS-550D transducer. The mice were lightly anesthetized (1.5% isoflurane), and the ejection fraction, fractional shortening, and ventricular chamber dimensions were determined in the M-mode using the parasternal short-axis view at the level of the papillary muscles. Measurements were calculated from the average of at least 3 consecutive cardiac cycles using the Vevo LAB program (VisualSonics). All echocardiographic measurements are reported in Supplemental Table 1.

### Histology.

Tissues were fixed in 10% neutral buffered formalin for 18 hours and processed for paraffin embedding. A Shandon Finesse 325 microtome (Thermo Fisher Scientific) was used to trim the surface of the paraffin blocks until all embedded tissues were exposed to the air. The blocks were then placed face down in ice water overnight to hydrate the tissue. Once hydrated, tissue blocks were cut in 5 μm sections. Paraffin sections were stained with Masson’s trichrome stain (Sigma-Aldrich) or H&E stain following the manufacturer’s protocol. For WGA staining, deparaffinized sections were subjected to antigen retrieval for 15 minutes in boiling sodium citrate buffer (10 mM sodium citrate pH 6.0, 0.05% Tween 20), rinsed in distilled water, incubated for 1 hour at room temperature (RT, 22°C) with blocking buffer (1% bovine growth serum in PBS), and incubated for 2 hours at RT with WGA, Alexa Fluor 488 conjugate (Invitrogen, W11261, 50 μg/mL). Slides were mounted with VECTASHIELD HardSet Antifade Mounting Medium (Vector Labs, H-1400-10) before imaging. Images were taken using an EVOS Cell Imaging System (Thermo Fisher Scientific). Images were quantified using ImageJ 1.53k.

### mRNA analysis by qPCR.

RNA was extracted using TRIzol (Ambion) and then reverse-transcribed using the High-Capacity cDNA Reverse Transcription Kit (Applied Biosystems). Selected gene expression differences were analyzed by qPCR using SsoAdvanced SYBR Green Supermix (Bio-Rad) in a 96-well format and the ΔΔCT method of analysis. Quantified mRNA levels were normalized to the housekeeping gene *Rpl7*, and expression is presented relative to control levels. The primers used were Rpl7 5′-TGGAACCATGGAGGCTGT-3′ and 5′-CACAGCGGGAACCTTTTTC-3′; Ucp1 5′-GCTTTGCCTCACTCAGGATTGG-3′ and 5′-CCAATGAACACTGCCACACCTC-3′; AdipoQ 5′-AACTTGTGCAGGTTGGATGG-3′ and 5′- GAGCGATACACATAAGCGGC-3′; Leptin 5′-TTCCTGTGGCTTTGGTCCTA-3′ and 5′-AGCACATTTTGGGAAGGCAG-3′; Prdm16 5′-GAACCAGGCATCCACTCGAA-3′ and 5′-CGTGTCCTCCTGTGACTTCC-3′; Glut4 5′-TGGTTCATTGTGGCAGAGCT-3′ and 5′-AGATCTGGTCAAACGTCCGG-3′; Pparα 5′-CAAAGACGGGATGCTGATCG-3′ and 5′-ATCCCCTCCTGCAACTTCTC-3′; Pparγ 5′-GAGGGCGATCTTGACAGGAA-3′ and 5′- TGTGATCTCTTGCACGGCTT-3′; Fasn 5′-CACAGTGCTCAAAGGACATGCC-3′ and 5′-CACCAGGTGTAGTGCCTTCCTC-3′; and Pgc1a 5′-GAATCAAGCCACTACAGACACCG-3′ and 5′-CATCCCTCTTGAGCCTTTCGTG-3′.

### Body composition.

An EchoMRI analyzer was used to determine the body composition of mice. Mice were first weighed and then carefully inserted into a plastic restraining tube to ensure that the mice remained still during measurement. The tube is then placed into the EchoMRI, run according to the manufacturer’s protocol, and subsequently calculates total lean mass and total fat mass. The fat and lean mass percentages are calculated by dividing the total fat/lean mass by the total body mass.

### Uniprot secretion analysis.

Secreted proteins were identified by searching Uniprot for proteins with a cellular component category of “Secreted” (“cc_scl_term:SL-0243”). The Uniprot settings were further adjusted to consider only mouse proteins that were reviewed by Swiss-Prot, had protein-level existence, and had an annotation score of 5. This was done to eliminate proteins that may be speculative or considered secretory because of similarity but were not confirmed.

### Exercise treadmill testing.

Exercise tests were conducted via Exer 3/6 Animal Treadmill (Columbus Instruments) using previously described methods (29). Following a period of acclimation, mice were subjected to an endurance or graded maximal exercise test. In brief, mice were placed on an enclosed treadmill at 0° incline, and the shock grid was activated. The treadmill speed (meters/min), duration (minutes), and grade (degrees) were then increased until exhaustion was reached with the following parameters: 0 m/min, 3 minutes, 0°; 6 m/min, 2 minutes, 0°; 9 m/min, 2 minutes, 5°; 12 m/min, 2 minutes, 10°; 15 m/min, 2 minutes, 15°; 18, 21, 23, 24 m/min, 1 minute, 15°; and +1 m/min, each minute thereafter. Exhaustion was defined as the point at which mice maintained continuous contact with the shock grid for ≥5 seconds, after which the treadmill and shock grid were ceased.

### GTT.

GTT was performed on mice fasted overnight (12 hours) to minimize variability in baseline glucose values. Prior to injection of glucose, mice were weighed and placed on a tail injection platform, to minimize handling stress, and a 26G needle was used to puncture the tail vein, forming a drop of blood. A glucose strip (Unistrip generic blood glucose test) was inserted into the glucometer (OneTouch Ultra2, LifeScan), and the drop of blood was placed onto the strip to measure glucose. Glucose (Sigma-Aldrich) was prepared as a 20% w/v stock solution in PBS and sterile filtered. The volume of glucose to be injected was calculated based on weight to reach a target concentration of 2 g of glucose per 1 kg of weight. Mice were injected intraperitoneally and placed back into their cage until the next glucose measurement. Glucose was measured 15, 30, 60, and 120 minutes following injection. Values for glucose were plotted against time to create a curve representing the response to glucose injection. GraphPad Prism software was used to calculate the AUC.

### ITT.

ITT was performed as described for the GTT but with the following changes. The mice were fasted for 2 hours, then injected with insulin, and glucose measurements were performed at 0, 15, 30, 45, and 60 minutes following injection. A 0.1 U/mL working solution of insulin was prepared from a 100 U/mL stock (HumulinR, Eli Lilly) in PBS and sterile filtered. The volume of insulin injected was based on weight, with a target concentration of 0.9 U insulin/1 kg weight.

### Metabolic cages.

Mice were placed in individual metabolic cages (Phenomaster, TSE Systems GmbH) for 7 days. Data on expired gases (VO_2_ and VCO_2_) and food and drink consumption were continuously collected. Individual mouse data from the last 5 days were averaged and used for the analysis. The respiratory exchange ratio was calculated as the VCO_2_/VO_2_. Energy balance was obtained by calculating the difference between food intake (kcal/h) and energy expenditure (kcal/h). Energy expenditure was calculated using indirect calorimetry according to the equation ([3.941 × VO_2_] + [1.106 × VCO_2_])/1,000. ANCOVA was performed using body mass as the covariate.

### Protein array and ELISA.

Circulating obesogenic factors were analyzed on plasma collected from mice following the manufacturer’s instructions for the Mouse Adipokine Array Kit (ARY013, R&D Systems, Bio-Techne) or FGF1 ELISA (ab223587, Abcam). Plasma was collected and isolated via cardiac puncture. Briefly, mice were anesthetized with isoflurane, and a 26G syringe was inserted through the skin to puncture the left ventricle. Blood was collected into the syringe and transferred to EDTA-containing tubes. Samples were centrifuged at 1,500*g* for 10 minutes at 4°C, and supernatant was transferred to a new tube.

### FGF1 treatment.

3T3-L1 cells (ATCC, CL-173) were differentiated as described (30). Briefly, cells were grown in expansion media (90% DMEM, 10% bovine calf serum). To induce differentiation, cells were transferred to DMEM with 10% FBS supplemented with 1 μM dexamethasone (Sigma-Aldrich), 0.5 mM 3-isobutyl-1-methylxanthine (Sigma-Aldrich), and 1 μg/mL insulin (Sigma-Aldrich) for 48 hours. Adipocytes were then maintained in DMEM with 10% FBS and 1 μg/mL insulin. For FGF1 treatment, cells were plated in expansion media and treated with 100 ng/mL FGF1 (PeproTech, 450-33A) or PBS for 72 hours, followed by the differentiation protocol. FGF1 or equal concentration of PBS was added to media for every step of differentiation. Concentration and timing were chosen based on published experimental designs (31).

### H9C2 and conditioned media transfer.

H9C2 cells (ATCC, CRL-1446) were grown in DMEM with 10% FBS and 1% penicillin/streptomycin until 70% confluence was reached. Knockdown of METTL3 was obtained by transfection of siRNA targeting Mettl3 (TriFECTa Mettl3 RNAi by Integrated DNA Technologies) or negative siRNA control (TriFECTa negative control DS NC1 RNAi by Integrated DNA Technologies). For conditioned media transfer, after 48 hours of knockdown, media were replaced with fresh media without siRNA. At 48 hours later, the media from siNC- or siM3-treated cells were transferred to differentiated 3T3-L1 cells for 24 hours.

### RNA-immunoprecipitations.

Fresh mouse heart tissues were excised, homogenized with handheld homogenizer, and sonicated in Buffer A (100 mM KCL, 5 mM MgCl_2_, 10 mM HEPES pH 7.0, 0.5% NP-40, 1 mM dithiothreitol, protease inhibitors). Sonicated samples were clarified by centrifugation at 4°C for 15 minutes. Pull-down was performed by incubating 2 mg of protein extract with 5 μg of antibody (anti-rabbit IgG, 12-370, EMD Millipore; anti-YTHDF1, 17479-1-AP, Proteintech; anti-YTHDF2, 24744-1-AP, Proteintech; anti-YTHDF3, 25537-1-AP, Proteintech) at 4°C for 4 hours, followed by the addition of 40 μL of protein A/G magnetic beads (88803, Pierce, Thermo Fisher Scientific) to the rotation overnight. The following day, samples were washed 5 times in Buffer B (50 mM Tris pH 7.4, 150 mM NaCl, 1 mM MgCl_2_, 0.05% NP-40), subjected to DNase I (AM2238, Invitrogen) digest at 37°C for 5 minutes, and then eluted in Buffer B, supplemented with 1 mg/mL Proteinase K (E195-5ML, VWR) and 0.1% SDS, at 55°C for 30 minutes. Following elution, RNA from input and immunoprecipitation samples was isolated using standard phenol-chloroform extraction followed by reverse transcription and qPCR with gene-specific primers.

### m6A-IP.

Total RNA was extracted from wild-type mouse heart tissues using TRIzol (Life Technologies). A total of 0.5 μg was reserved for input and subjected to reverse-transcription qPCR. A total of 10 μg of each purified RNA extract was incubated with 5 μg of m6A polyclonal antibody (Synaptic Systems) or control anti-rabbit IgG (MilliporeSigma, 12-370) in ice-cold IPP buffer (150 mM NaCl, 0.1% NP-40, 10 mM Tris-HCl at pH 7.4, and 200 U/mL RNase inhibitor) for 2 hours at 4°C. Following the 2-hour incubation, prewashed A/G magnetic beads were added into RNA-m6A IPs and subjected to incubation for an additional 2 hours at 4°C. The beads were then washed 5 times with 1 mL ice-cold IPP buffer. The IP complexes were digested with proteinase K (VWR, E195L) in the presence of 0.1% SDS at 55°C for 30 minutes, and the RNA was isolated using phenol-chloroform extraction followed by reverse transcription and qPCR with gene-specific primers.

### mRNA stability assay.

mRNA stability assays were performed in H9C2 rat cardiomyoblast cells transfected with either control nontargeting siRNA or a pool of 3 rat-specific siRNAs targeting *METTL3* gene (Integrated DNA Technologies). For RNA stability, experiments were performed 48 hours after siRNA transfection with Lipofectamine RNAiMax (Invitrogen; 13778150) using 10 μg/mL actinomycin D (A1410; Sigma-Aldrich) as a transcription inhibitor. Cells were treated with actinomycin D for 0, 0.25, 0.50, 1, 2, 4, or 8 hours (*n* = 5 wells per condition). RNA was extracted by TRIzol, and the *Fgf1* mRNA levels were measured by qPCR using the following rat primers: Fgf1 5′-TGGGAGGAAGGGCGGTAATC-3′ and 5′-TATCACTGCCACCTGCGTTC-3′; The best-fit values for mRNA decay rate (*k*) and half-life were calculated via nonlinear least squares regression curve fitting (1 phase decay).

### Statistics.

All data are presented as mean ± SEM, with dots indicating individual biological samples within a group. Statistical analysis between 2 groups was performed using an unpaired 2-tailed *t* test for normally distributed data with a *P* ≤ 0.05 considered significant. For groups of 3, a 1-way ANOVA followed by Tukey’s multiple-comparison test was performed, with statistical significance set at α = 0.05. For groups of 2 genotypes and 2 conditions, a 2-way ANOVA followed by Tukey’s multiple-comparison test was performed, with statistical significance set at α = 0.05. ANCOVA was performed to include body mass as the covariate in metabolic cage experiments. Bioinformatic statistics used default settings for each individual pipeline unless otherwise noted. Data analysis was performed using GraphPad Prism 9 (GraphPad Software).

### Study approval.

All presented experiments comply with the standards set forth by the Institutional Animal Care and Use Committee at The Ohio State and Brown Universities and the *Guide for the Care and Use of Laboratory Animals* published by the US NIH (National Academies Press, 2011). Animal use protocols were approved by the Institutional Animal Care and Use Committee and Institutional Biosafety Committee at The Ohio State University and Brown University.

### Data availability.

Data can be found in the Supporting Data Values file or obtained from the corresponding author upon request.

## Author contributions

CR and JZL performed all experiments and analysis not otherwise attributed. ISNDV and CD performed and analyzed Nanopore sequencing. JS and GAK performed and analyzed metabolic cage experiments. JMP performed and analyzed treadmill experiments. CR and FA wrote and edited the manuscript. FA conceived, supervised, and funded the project.

## Supplementary Material

Supplemental data

Unedited blot and gel images

Supporting data values

## Version 1 04/24/2025

In-Press Preview

## Version 2 06/09/2025

Electronic publication
