# Supplementary material for: The cardiac METTL3/m6A pathway regulates the systemic response to Western diet
Source: JCI Insight. 2025 Apr 24;10(11):e188414. doi: 10.1172/jci.insight.188414 (PMC12220958; doi:10.1172/jci.insight.188414)
Supplement: Supplemental data [file jciinsight-10-188414-s052.pdf]

Supplemental Figure 1

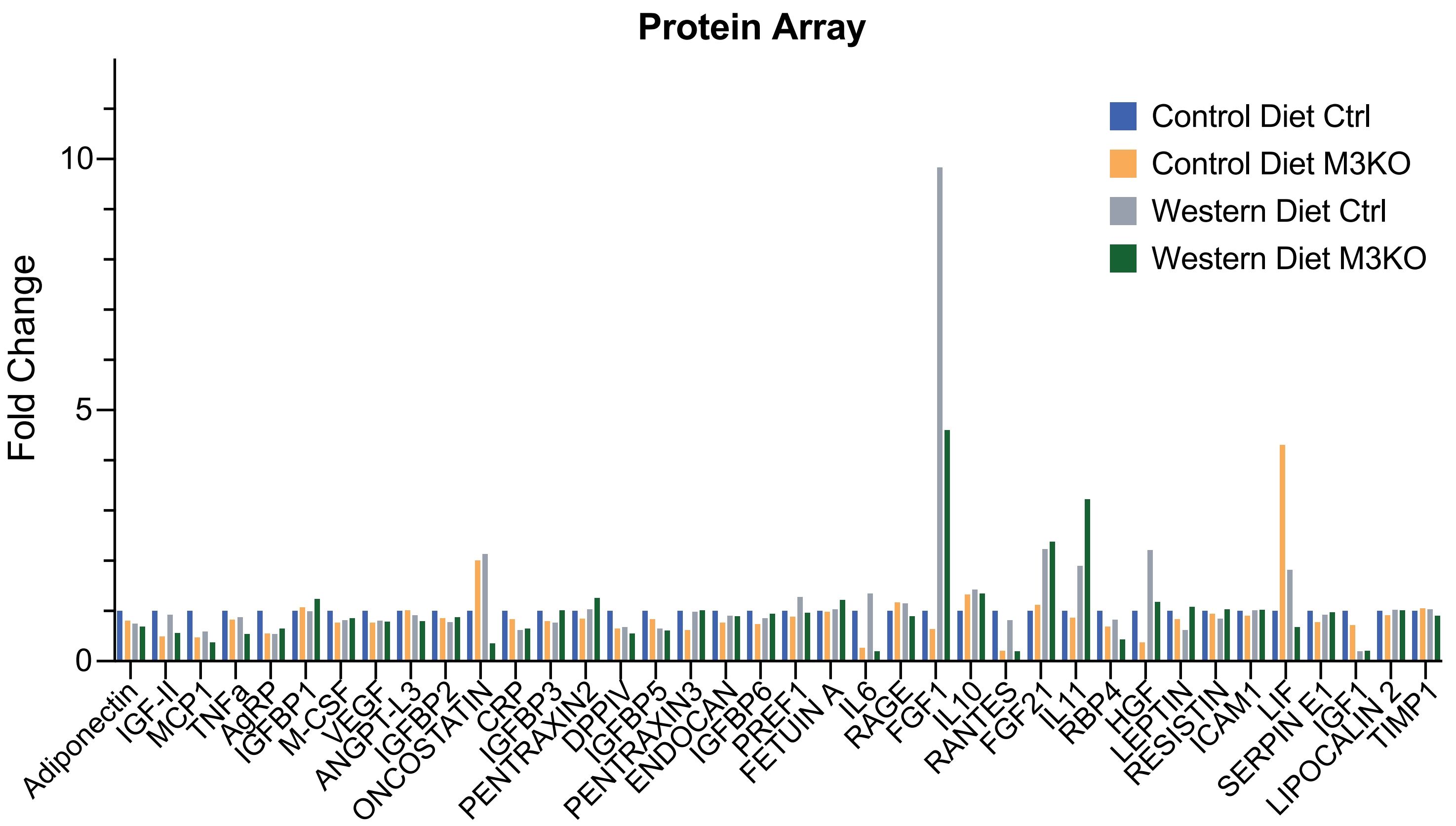

Supplemental Figure 1: Protein array on pooled plasma samples from mice. n=3 (control diet WT and M3KO) or n=4 (western diet WT and M3KO) mice.

Supplemental Figure 2

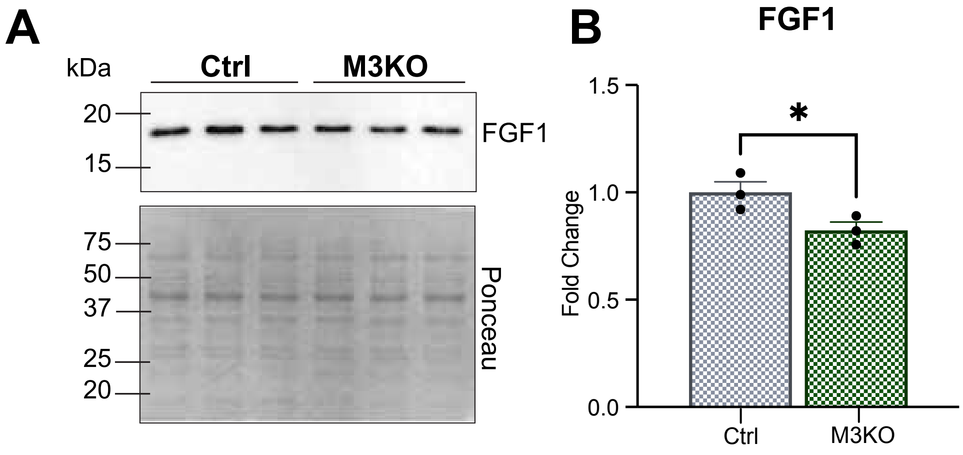

Supplemental Figure 2: Western Blot for FGF1 in Ctrl and M3KO mice. n=3 per group. Data normalized to Ponceau loading control. Unpaired t-test was used. *p<0.05.

Supplemental Figure 3

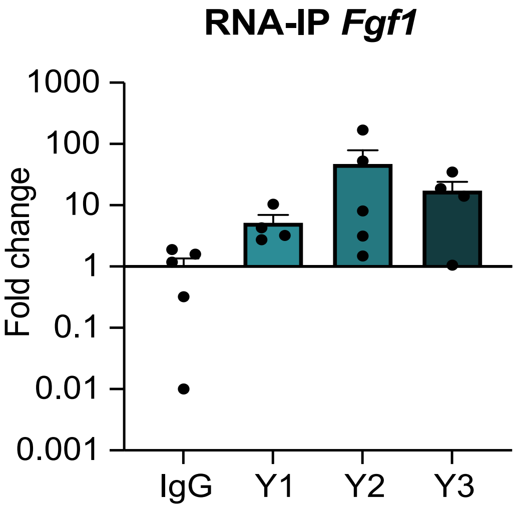

Supplemental Figure 3: RNA Immunoprecipitation (RNA-IP) for Immunoglobulin G (IgG) as control, YTHDF1 (Y1), YTHDF2 (Y2), and YTHDF3 (Y3). n=(5,4,5,4) for (IgG, Y1, Y2, Y3). Data normalized to IgG. One-way ANOVA with comparison to IgG was used.

Supplemental Table 1
Overlapping transcripts present in both conditions

Hspb7
Tpm1
Mb
Gm12191;Cnot6
Sdhb
Cox7a2
Myl2
Actc1
Cyc1
Slc25a4
Myl3
Atp5a1
Mdh1
Cox6a2
Hbb-bs
Atp2a2
Fabp3
Atp5g3
Gja1
Cox5a
Tnni3
Cox6b1
Pln
Decr1
Suds3
Nme2;Gm20390
Myh6
Atp5f1
Cryab
Hspb8
Cox6c
Csrp3
Gm7336
Atp5b
Myl12a;Myl12b
Stard7
Chchd10
Uqcrh
BC002163;Zbtb20
Mdh2
Tnnt2

AC141471.2;Ube2d2a
Flnc
Atp5j2
Acot13
Atp6v1c1
Fth1
Tpt1
Slc50a1
Ldhb
Ndufb9;Gm49356
Fkbp3
Hadh
Ncoa2
Ndufab1
Rps10;RPS10-NUDT3
Asb2
Ndufs4
Aco2
Pdk4
Fhl2
Ndufa7
Tmem126b
Ndufa4
Rps5
Aes
Hsp90b1
Des
Eef2
Atp5c1
Eif5
Ywhag
Ndufs2
Map1lc3a
Gpi1
Nnt;Nnt
Ndufa12
Uqcrfs1
Gm15459
Adsl
Atp5e
Ankrd1
Ttn
Pitpna
Gm13910
Oaz1

Cdip1
Skp1a
Sod2
Gm14303
Prdx1
Ldha
Mtx2
Pkm
Fis1
Gm10053
Cox7a1
Uqcr10
Arpc2
Ndufb3
Atp5g1
Etfa
Ndufb5
Ivns1abp
Acat1
Psmc2
Abcf2
Rpl4
Gm49369;Ctsd
Gnai2
Eno3
Aldoa
Ckm
Myoz2
Smyd1
Rpl8
Egln1
Fabp4
Rps15
Idh3b
Tnnc1
Slc25a3
Cs
Rpl9-ps6
Uqcrq
Lrrc10
Clu
Ckmt2
Gm12174
Ndufs8
Pcdh7

Gm15501
Hspa8
Gm10250
Ndufa6
Usmg5
Pkia
Lynx1
Got2
Ndrg2
Rhob
Chchd3
Ndufa5
Vegfb
Vdac2
Ndufb8
Chchd2;Phkg1
Tmod1
Timm23
Rgs5
Gm12338
Col4a1
Ndufv2
Gm10123
Prdx6
Myo18b
Rab35
Idh3a
Ndufb4
Suclg1
Ndufv1
Pgam2
Rps23-ps1
Uqcrc1
Coq8a
Cycs
Arhgdia
Etfb;Gm45233
Gpr27
Gsn
Hsp90ab1
Acsl1
Rps3a2
Sdhd
Hadha
Chid1

Stub1
Nampt
Fahd1
Ech1
Gm10175
Cd36
Tcp1
Lpl
Cfap77
Cdk9
Rpl7
0610012G03Rik
Tmem115
Crat
Rpl18
Lbh
Slc2a4
Hipk3
Hspb6
Vdac1
Gcsh
Atp5o
Hibadh
Clpx
Serinc1
Atp6v0e
Ppp1r3c
Gabarap
Gm12033
Tpt1-ps3
Actn2
Gm5559
Ndufa11
Srl
Atp1a1
Fh1
Gng5
Mpc1
Tubb4b
Idh2
Aqp1
Pink1
BC004004
Glrx
Slc41a3

Ndufa9
Oxct1
Sucla2
Cnbp
Afg3l2
Gm6204
Psma7
Pdhb
Ctsl
Ndufs6
Acaa2
Rrad
Ndrg4
Mfn2
Acadl
Mrps36
Copb2
Ctsb
Eif5a
Cdc37
Casq2
Pnpla2
Ucp3
C1qbp
Cystm1
Dazap2
Hadhb
Pdlim5
Acadvl
Sod1
Hikeshi
Mrpl41
Oat;Fgfr2
Ndufb10
Mybpc3
Dsp
Sdhc
Prdx2
Uqcrc2
Lmod2
Podn;Scp2
Tmbim6
Ndufs3
Gm13394;Cfap77
Rps3a1

Rpl6
Cox14
Slco3a1
Ogdh
Obscn
Trappc5
Chchd4
Vps35
Ybx1
Ndufb7
Ckb
Laptm4a
Hba-a2
Popdc2
Mul1
Jph2
Mrpl51
Tmem259
Ptp4a3
Rpl37rt
Atp5h
Cox7a2l
Gm9385
Gm15500
Txlnb
Polr2m
BC005537
Anxa6
Mpc2
Sdha
Txn2
Spryd7
Ndufb1-ps
Gtf2i
Ipo5
Hspd1
Acadm
Gm10073
Slc25a11
Tcap
Rpl35a-ps3
Surf4
Ppp2ca
Fam162a
Hbb-bt

Psmd7
Acacb
Fxyd1
Gm28661
Rps14
Adprhl1
Tmem38a
Rps11
Gm12537
Tnfrsf1a
Ldb3
Hbs1l
Fam174b
Psap
Polr2h
Rpl10-ps3
Ankrd23;Gm42417
Fxr1
Ndufs7
Sparcl1
Gm10709
Rps24
Etfdh
Tomm5
Trp53inp2
Atp5j
Eif4e
Eef1a2
Atp5d
Ndufc1
Hspb1
Park7
Mlf1
Slc38a2
Ndufv3
Ubl3
Gm9794
Mapkapk2
Lamp1
Atp5o;Atp5o
Timm17a
Hspa5
Car14
Gm6563
Fxn

Mt1
Nol7
Crip2
Sparc
Ano10
Cst3
Gm8730
Echs1
Fbxo40
Ccdc85b
Pdlim1
Maea
Spcs1
Coq10a
Gm15772
Jund
Prdx3
Tfam
Mrpl18
Gpx4-ps2
Tsc22d1
Rps6ka2
Pygm
Got1
Cish
Cfl2
Hmox2
AC132253.9
Rpl29
Ugp2
Ywhaq
Rps19-ps6;Tecpr2
Ddrgk1
Oaz1-ps;AC154200.1
Zfp106
Gm10221
Itm2b
Dbi
Lap3
Gm10076
Glrx3
Rps16-ps2
Trim63
B4gat1;AC124502.3
Tpi1

Psmc1
1700021F05Rik
Tecr
Eef1a1
Acyp2
Mccc1
Ndufb6
Phb2
D10Jhu81e
Psmb4
Spag7
Ccng1
Ndufs1
Rps2
Coro6
Acss1
Ppif
Tuba4a
Dnaja1
Sh3bgr
Fkbp4
Hba-a1
Ube2d3
Ndufa8
Pxmp2
Serinc3
Psmb7
Stom
BC003965
Rab12
Hagh
Col3a1
Entpd5
Ctnna1
Ank1
Asb8
Tspan3
Ghitm
Vegfa
Slc16a1
Ninj1
Dlst
Phyh
Cux1
Flot1

Gnas
Ackr3
Eif4g2
Ifngr1
Rhoa
Gm7536
Map2k2
Elob
Ddx5
Apobec2
Sdf2
Rps3
Mrps23
Gas6
Ostc
Ankrd40
Gsto1
Ecd
Tesk1
Pdhx
Nap1l4
Lmo7
Mrps34
Ephx2
Tmed10
Smim12
Nexn
Uba52;Kxd1
Odc1
Syngr2;Gm20708
Mad2l1bp
Kcng2
Eif1
Gm28048;Commd1
Ehd4
Cox7c
Pmpcb
Rps17
Dld
Psmb2
Fkbp1a
Pfkm
Art1
Anxa5
Nudt7

Higd2a
Rtraf
Cav1
Ly6a
Ctbp1
Npepl1
Smim10l1
Ybx3
Zfand5
Ralgapa1
Klhl24
Clic5
Eif3b
Myzap
Tufm
Ywhae
Fem1a
Cavin2
Coq9
Mgll
Ube2k
Hist1h2bc
Pygb
Eif3h
Creg1
Ubc
Tfg
Wnk1
Pkig
Gm10443
Ss18l2
Sar1a
Phb
Rilpl1
Oaz2
Pttg1
Trap1
Klf2
Pde4dip
Rit1
Mlf2
Mrfap1
Yipf7
Srf
Ehd1

Ube2r2
Ndufb2
Bag3
Uba3
Becn1
Lmod3
Aimp2
Timm50
Arpc5l
Zfp260
Glud1
Rps15a
Nadk2
Rtca
Ndufa10
Pdk2
Akr1b3
Hsdl2
Gm28437
Hk2
Adgrf5
Sgcg
Gm11273
Rhoc
Use1
Hspb2
Dynlrb1
Cpt2
Plekho1
B2m
Psmb5
Sirt2
Rplp2
Bsg
Agpat3
Cyb5a
Hint2
Mlycd
Wdr20
Mrpl12
Pla2g16
Fhod3
Pmpca
Arpc3
Ryr2

Ctsd
Il10rb
Saraf
Tm4sf1
Retreg1
Grsf1
Cox19
Mtfr1l
Ehbp1l1
Narfl
Mrpl30
Mbnl2
Iscu
Smpd1
Reep5
Abcb8
Hfe2
Vdac3
Map1lc3b;Gm20388
Cox6a1
Tuba1b;Gm49450
Rpsa
Tmem30a
Dhrs11
Rpl36
Cav3
Arf1
Gstm1
Phospho1;Zfp652
Emc6
Rnf187
Ak1
Dnajc4
Slc35a4
Vti1b
Calm2
Pa2g4
Msl1
Nfib
Nudt8;Gm49405
Ppdpf
Vapb
Cd59a
Hrc
Lyz2

Btf3l4
Hmgn2
Prpf19
Mrpl13
Rbp7
Gata4
Slc20a2
Gm10288
Usp13
Akt1
Prkcsh
Dcun1d2
Cog6
Fam69b
Twf2
Rpl41
Fitm2
Tsc22d4
Higd1a
Rpl15-ps3
Qk
Smim19
Rnf14
Lpin1
Gpx4
Eci2
Rps6-ps4
Prdx5
Enah
Cyb5r3
Rplp0
Aars
Gmpr
Eci1
Dnaja2
Uqcrb
Pdrg1
Tomm40
Cct6a
Rftn1
Gpx3
Mrps28
Mettl23
Usp24
Rpl19-ps11

Gm12671
Chmp2b
Gpihbp1
Fam220a;Fam220a
Rpl36-ps12;Sumf1
Rps19
Eif4g1
Tmem65
Maf1
Capns1
Isca2
Fndc5
Hopx
Nipsnap2
Dlat
Psmb1
Hist1h1c
Pnrc1
Trim55
Acads
Rbpms
Apoe
4930453N24Rik
Rpl28-ps1
Rps9
Ptgds
Mrpl55
Cd81
Csde1
Ptpn3
Steap3
Mtus2
Pebp1
Csdc2
1810058I24Rik
Jtb
Mpc1-ps
Tsfm
Mrps7
Fopnl
Nedd4
Psma2
Fitm1
Hspe1
Lman2l

Srsf2
Rab28
4933434E20Rik
Rpl35a-ps2
Mrps18a
Coa3
Btf3
Mrpl16
Rpl10a-ps1
Rpl11
Ptp4a2
2310011J03Rik
Mrpl15
Rnf10
Xbp1
Tango2
Prkar1a
Fbxo8
Inpp5a
Atxn10
Guk1
Msrb2
Emc2
Prkab1
Adamtsl5
Gm44502;Cpt1b
Vim
Ptpmt1
Tesc
Hbp1
P4ha1
Eno1
Pnpla8
Hspa9
Gpcpd1
Sptbn1
Map7d1
Tnni3k
Naca
Col4a2
Ddx39b
Uqcc3
Ablim1
Dmac2
AC165425.1;Fyco1

Gm2962;Fcrla
Nagk
H2-K1
Ly6e
Selenot
Hk1
Gyg
Scn5a
Ftl1
Hibch
Ppp2r2d
Igfbp7
Acta1
Gm49673;Rnaset2a
Ube2b;Gm26551
Vamp5
Emc3
Crip1
Atp6v0c
Ube2d1
Slc25a33
Slc9a3r2
Rack1
Cenpb;Spef1
Rheb
Hsp90aa1
Atp6v1d
Ndufa13;Yjefn3
Asph
Rbm38
Nsmce1
2410015M20Rik
Bola1
Dnpep
Atp1a2
Clasp1
Rpl36a-ps2;Ddah1
Dynll1
Ubb
Rpl23a
Rps21
Tmed1
Dele1
Nucb1
Ank

Gm11808
Fdx1
Gatad1
Slc25a20
Slc38a10
Hsd17b11
Pdss2
G3bp1
Tcp11l2
Hhatl
Palmd
Rab1b
Gm12715
Imp3
Commd3
Nedd8
Hcfc1r1
Smpd2
Mfsd5
Serf2;Hypk
Mfn1
Myom1;Gm26561
Aimp1
Mylk3
Nfe2l1
Wfs1
Rnf11
Emc4
Bag1
AC119982.1
Ddx50
Uqcc2
Lrrc14b
Sumo1
Eng
Zfand3
Tns1
Ogn
Trak1
Ptcd3
Sqstm1
Ptgr2
Selenow
Tmem14c
Ift81

Myom2
Capzb
Bnip3
Znrf1
Mbd3
Pgm1
Ppm1a
Kdm1a
Cpe
Mmadhc
Spr
Chd4
Mrps27
Gm21988;Rnasek
Nptn
Fsd2
Nudt19
Plin5
Hspg2
Ptges2
Gm49405;Doc2g
Mapk14
Mov10l1
Itgb1
Gnpat
Actg1
Bckdha
Ubr3
Slc25a34
Usp19
Rps6
Mrpl20
Xirp1;Cx3cr1
Sgca
Map2k3
Swi5
Poldip2
H1f0
Puf60
Cdh13
Ivd
Slc48a1
Rpl18a
Grhpr
Vcp

Slc40a1
H3f3a
Nr2f6
Ufc1
Psmb6
Gdi2
Gm13340
P4hb
Itgb5
Srsf5
Rpl19
Grpel1
Nrd1
Mrpl4
Eif4a2
Pfn2
Sf3b5
Pacsin2
Atp5l
Sf3b1
Immt
Ak3
Acad12
Zfp706
Ccni
Scp2-ps2
Minos1
Dcaf11
Rcan2
Gm4459;Cfap46
Mrpl27
Aplp2
Ubxn6
Lamtor4
Grb14
Rsrp1
Akr1a1
Tomm20
Ubl7
1810013L24Rik
Tysnd1
Antxr2
Pdf;Cog8
Frmd5
Nkiras1

Mrpl9
Lrrc39
Mcrip2
Kmt5a
Tusc2
Hspa4
1700020I14Rik
Mrps22
Ccn2
Oxa1l
Tgm2
Gadd45gip1
2310061I04Rik
Edf1
Tmem160
Gm42688;Mrpl53
Anapc5
Rps26-ps1
Kcmf1
Coq3
Rdm1
Gstp1
Tfpi
B230118H07Rik
Ppp1r12b
Emg1
Cct7
Aurkaip1
Tcn2
Mcl1
Eif4h
Rpl34-ps1
Rpsa-ps10
Eif3f
Chtop
Sord
Gm10925
Mprip
Eif3c
Arhgef10l
Ppm1b
Rpl34
Esrra
Hnrnpa2b1
Dgat2

Rassf3
Ppp1r14c
Sgcb
Snx3
Hnrnpab
Gm49708
Ipo11
Jpt1
Acta2
Matr3
Gm44386
Dmpk
Triap1
Lrpap1
Eny2
Polr2e
Rpl26
Cited4
Babam1
Tmem246
Tmem242
Mrpl54
Fam213b
Ddx54
Rplp1
Pttg1ip
Cdh2
Ist1
Slc25a12
Gm26712;Pyurf
Eif4a1
Cnih1
Lrp10
Vldlr
Hsbp1
Bves
Smim26
Mtch2
Chmp7
Rdh5;Bloc1s1
Taco1
Rpl36al;Gm49383
Smyd2
Itfg1
Cops2

Rab14
Eif4ebp1
Psmc3
Hnrnpk
Rer1
Kpnb1
Tomm6;Gm21981
Slc25a13
Prkaca
Inppl1
2610507B11Rik
Cab39
Fuom
Jak1
Mrpl50
Cfl1
Plin3
Ubl5
Srp14
Tuba1b
Aasdhppt
Spsb1
D8Ertd738e
Rpl36-ps3;Gm40271
Eif3j1
Chmp2a
Rapgef1
Nkx2-5
Tnfrsf12a
Zadh2
Drap1
Ier3
Plin4
Amd1
Fabp3-ps1
Sox18
Eef1d
Tmem134
Mrpl32
Snx5
Eif3i
Cluh
Serping1
Ndufa3
Pdia6

Hint1
Ppp1ca
Bdh1
Tacc2
Polr1d
Ndufs5
Fblim1
Cops6
Mob2
Serpinh1
Fdft1
Psme1
Psmd2
Vamp3;Camta1
Sept7
Nbr1
Mgrn1
Gm10039
Emilin2
C130080G10Rik;A530058N18Rik
Vps52
Fuca2
Dhrs7
Rab21
Iqschfp;Schip1
Dag1
Rab7
Mrpl44
Ptrh2
Pnkd
Rtn3
Cdc34
Tmbim4
Rhoq
Picalm
Mepce
Zfp970
March2
Ptms
Isca1
Me1
1810037I17Rik
Ccnd1
Ppa2
Gm20498;Synj2bp

Ltbr
Nppb
Rdx
Eef1g
Cops4
Gpx1
Mrpl46
Plec
Pcbp1
Rnf13
Ilk
Ppp3cb
Atxn2l
Pafah1b1
Gns
Cldn5
Rras2
Atcayos
Atp6v1g1
Mknk2
Glul
Acot2
Mrps2
Myadml2
Ube2n
Mrps35
Mut
Comt
Sh3glb1
Cmya5
Hnrnpu
Rnf7
Psma1
Nceh1
Trim54
Rock2
Sptssa
Gm20390;Nme1
Ctnnb1
Pank1
Gm8430
Pfkl
Htra1
Gchfr
Rpl13a;Gm45713

Prrc2a
2810025M15Rik
Ppp2r3a
Akap1
Lrrc2
1190007I07Rik
Dcaf8
Tcea3
Por
Sumo2
Herpud1
Tnfaip8
Ndufaf1
Arpc1a
Amfr
Gtf2h5
Neurl2
Gm49373;Gm38431;Fdx1l
Rpl3l
Tmem9b
Tm2d2
Hnrnpa0
Cyhr1
Trafd1
Gpam
Bnip2
Mff
Yipf3
Elp5
Fech
Bzw1
Hspb3
BC029722
Gpsm1
Txnip
Mrps18c;Gm43511
Ube2v1;Gm20431
Mrpl14
Sdr39u1
Mettl9
Vamp2
Dhrs7c
Kcnk3
Pim3
Nt5m

Trim72
Zfp651
Prox1
Crk
Dnajb4
Glrx5
Abhd11
Gstm5
Dnajb2
Vdac3-ps1
Cdc42
Stard3
Tuba8
Slain2
Doc2g
Pex11a
Auh
Scand1
Sqor
Neat1
Cisd1
Pacsin3
Srsf3
Slc38a3
Chpt1
Psme4
Dhrs4
Abcf1
Naa20
Rap1b
Abra
Gna12
Adipor1
Cap2
Tmem59
Asrgl1
Smim20
Dhrs3
Tfeb
Arpp19
Actr1b
Hdlbp
Snrpb
Kif1c
Myadm;Prkcg

Cdv3
Macf1
Coa5
Vps28
Lman1
Tmem222
Mcrip1
Asb14
Cds2
Mrps24
Grn
Hnrnpa1
Gm16286;Txnl4a
Paip2
Agpat2
Hand2
Phtf2
Gm28551;Asnsd1
Erp29
Gm2000
Efnb3
Stx12
Gm12251
Manf
Pard3
Prelid3b
2010111I01Rik
Eif4g3
Aldh2
Wdr83
Ypel3
Gm29253;Atg9a
Nck1
Chrac1
Ctdsp1
Rpl9
Hist1h4h
Mrpl28
Lims1
Dstn
Ankrd9
Abcc9
Tgoln1
Mat2b
Cuedc2

Selenop
Psma4
Ncor2
Wdr45b
Tmem223
Cct2
Tob2
Emc9
Prnp;Prn
Ran
Fbxl22
Tmem11
Pelo;Itga1;Gm49395
Rcsd1
Ndufaf4
Clic4
Plpp7
Cd300lg
Mrps17
Nudt3
Serbp1
Hnrnpf
Thoc7
Adhfe1
Ddx1
Atp1b1
Ramac
Osbpl2
Timm8b
Nob1
Zfp672
Pccb
Oxld1
Dnaja3
Perm1
Epn3
C1qb
Echdc3
Bod1
Tmem201
Tsn
Usp16
Smdt1
2810004N23Rik
Myl4

Clip4
Ktn1
Pofut2
Arfrp1
Cfap46
Adk
Plxnb2
Aamp
Gfm1
BC031181
Abracl
Phldb1
Rab2a
Ier5
Psmd14
Bcat2
Ulk1
Pcmt1
Trappc3
Cltb
Cse1l
Raf1
Gm10273
Tomm22
Epas1
Ces1d
Cmss1
Coq6
Ncor1
Pcmtd2
Acot7
Pef1
Rnpepl1
Ifngr2
Tprgl
Blvrb
Camk1
Apopt1
Riok3
Morf4l1
Rab5if
Tbc1d9b
Npm1
Wbp11
March5

Ociad1
Fam98a
Apoo-ps
Parm1
Mfge8
Cops5
Mrpl10
Svil
Gemin7
Abhd5
Nebl
Ntmt1
Slc25a39
Blcap
1110004F10Rik
Actr3
Rbm24
Tmem70
Tars2
Acad11
Spop
Ddx17
Btg2
Nudt4
Osbpl9
Afg1l
Pabpc4
Arcn1
Capza2
Ciapin1
Cdadc1
Acox1
Tmem143
Fbxl3
G0s2
Ptpa
Usp28
Hdhd2;Hdhd2
Pcbp2
Atpaf1
Qdpr
Cops8
Rab5c
Trappc6b
Timm10

Rnh1
Fam8a1
Rpl37a
Luc7l2
Med29
Mpv17
Rpl7a
Kif5b
Arl2
Mrpl43
Psmc4
Supt4a
Timm9
Snapc5
Mtfp1
Ywhab
Slc30a9
Spag9
Atxn2
Mrpl3
Rps16
Nfe2l2
Cmc2
Bckdk
Nae1
Btbd1
Ptcd2
Ube2l3
Uap1
Lrg1
Hdgf
Wtap
Polg
Rab11b
Vmp1
Il15
Wsb2
Dap3
Snu13
Thap12
Xirp2
Ctdnep1
Gm6136
Med17
Pkp2

Ppp6r3
Timm10b;Gm45799
Paip2b
Tmem182
Ppp2r1a
Atad3a
Szrd1
Nsa2
Lrrc24;C030006K11Rik
Zfand6
Dmac1
Sco2
Arf4
Commd6
Derl1
Dda1
Pfn1
Cul1
Epn1
Me3
Scarb2
Mrps34;Nme3
Dusp1
Ahnak
Gpr180
Cul3
St3gal5
Atp1b3
Etfrf1
Mylk4
Sirt5
Gcdh
Gm21969;Aldh4a1
Pex7
Gm28635;Tmx2
Rac1
Gm20388
Igf2r
Bmi1
Fbxo31
Csnk2a1
0610009B22Rik
Rabggtb
Dmwd
Rp9

Coq7
Slc35f6
Setd3
Tmem167
Mrpl45
Zmat5
Azin1
Tmem41a
Txndc9
Esd
Cmbl
Trak2
Sec13
Ddost
Gata6
Rtn2
Chmp4b
Mef2a
Stk39
Ssna1
Ctsa
Nrtn
Mrpl19
Sacm1l
Cox10
Cat
Dolk
Tmed4
Fbxw5
Gm20427;Slc39a7
Ginm1
Ppp2r2a
Preb
Usp47
Slc25a28
Mir7225;Pde4dip
Dynll2
Apbb1
Cd164
Rnf139;Gm49356
Perp
Uqcc1
Large1
Ahsa1
Srp68

Arhgap29
Psenen
Serpinb6a
Mat2a
Tmem60
Coq10b
Xpnpep1
Abcd3
Ets2
Arhgef7
Adh5
Derl2
Nt5c3
Dennd5a
Ttc4
Txndc5
Ube2f
Ifrd2
Mrps9
Kif1b
Pdia3
Dctn2
Ppp2r5c
Gde1
Ide
Vwa8
Trdn
Bad
Cav2
Plpbp
Itm2c
Ppic
Eif2b1
Dnajc8
Mkks;AL731706.1
Yap1
Adcy5
Fgf1
Alkbh7
Mcee
Capn2
Habp4
Akirin2
Efcab2
Dym

Mcat
Srsf6
Trappc2l;Gm20388
Gm7730
Khdrbs1
Nutf2-ps1
Suclg2
Prmt1
Psmd4
Rbm42;Gm21982
Calu
Gm43552;Smim14
Cebpb
Lrpprc
Psmc6
Tnip1
Flad1

Chromosomal Locations for m6A sites in Control Diet vs Western Diet Conditions

chrom	chromStart	chromEnd	name	strand
4	141425129	141425134	Hspb7	+
9	67029668	67029673	Tpm1	-
4	141424038	141424043	Hspb7	+
15	77015790	77015795	Mb	-
11	49678959	49678964	Gm12191;Cr	-
4	140966574	140966579	Sdhb	+
9	79755548	79755553	Cox7a2	-
4	141421765	141421770	Hspb7	+
5	122106762	122106767	Myl2	+
4	141424507	141424512	Hspb7	+
2	114050358	114050363	Actc1	-
15	77015620	77015625	Mb	-
15	76345812	76345817	Cyc1	+
8	46209339	46209344	Slc25a4	-
15	77017785	77017790	Mb	-
15	77015963	77015968	Mb	-
2	114050292	114050297	Actc1	-
9	67029688	67029693	Tpm1	-
2	114047295	114047300	Actc1	-
9	110766760	110766765	Myl3	+
18	77782319	77782324	Atp5a1	+
11	21557413	21557418	Mdh1	-
2	114050518	114050523	Actc1	-
8	46210888	46210893	Slc25a4	-
7	128205643	128205648	Cox6a2	-
7	103826635	103826640	Hbb-bs	-
5	122453552	122453557	Atp2a2	-
5	122105001	122105006	Myl2	+
4	130312374	130312379	Fabp3	+
2	73909292	73909297	Atp5g3	-
8	46209354	46209359	Slc25a4	-
2	73909215	73909220	Atp5g3	-
10	56968704	56968709	Gja1	-
15	77015628	77015633	Mb	-
2	114049245	114049250	Actc1	-
9	57531719	57531724	Cox5a	+
4	130315325	130315330	Fabp3	+
2	114050492	114050497	Actc1	-
4	130315202	130315207	Fabp3	+
5	122106774	122106779	Myl2	+
5	122102709	122102714	Myl2	+

7	4521891	4521896	Tnni3	-
15	77016020	77016025	Mb	-
15	77022503	77022508	Mb	-
7	103827476	103827481	Hbb-bs	-
3	96577928	96577933	Polr3gl	-
7	30624550	30624555	Cox6b1	-
9	67031059	67031064	Tpm1	-
8	46209270	46209275	Slc25a4	-
10	53344118	53344123	Pln	+
10	53345672	53345677	Pln	+
7	4518444	4518449	Tnni3	-
7	128205902	128205907	Cox6a2	-
10	53345153	53345158	Pln	+
8	46207597	46207602	Slc25a4	-
8	46207419	46207424	Slc25a4	-
15	77015614	77015619	Mb	-
15	77015835	77015840	Mb	-
7	128205714	128205719	Cox6a2	-
7	30624505	30624510	Cox6b1	-
5	122102742	122102747	Myl2	+
7	128205970	128205975	Cox6a2	-
7	4520493	4520498	Tnni3	-
4	15930951	15930956	Decr1	-
2	114050590	114050595	Actc1	-
9	57529043	57529048	Cox5a	+
9	67036100	67036105	Tpm1	-
2	73908590	73908595	Atp5g3	-
15	77017745	77017750	Mb	-
5	117092382	117092387	Suds3	-
11	93951938	93951943	Nme2;Gm20	-
14	54942363	54942368	Myh6	-
3	105954156	105954161	Atp5f1	-
5	122454000	122454005	Atp2a2	-
12	111961507	111961512	Atp5mpl	-
9	50756405	50756410	Cryab	+
5	116408977	116408982	Hspb8	-
2	114049562	114049567	Actc1	-
8	46208473	46208478	Slc25a4	-
11	21559223	21559228	Mdh1	-
2	114052010	114052015	Actc1	-
15	35931994	35931999	Cox6c	-
9	67031034	67031039	Tpm1	-
7	48835523	48835528	Csrp3	-
7	51747094	51747099	Gm7336	+
15	77015886	77015891	Mb	-

15	77017648	77017653	Mb	-
10	128086126	128086131	Atp5b	+
8	120674018	120674023	Cox4i1;Gm2(	+
7	48830490	48830495	Csrp3	-
17	70994374	70994379	Myl12a;Myl1:	-
2	127298802	127298807	Stard7	+
18	77782350	77782355	Atp5a1	+
4	130315242	130315247	Fabp3	+
8	46209030	46209035	Slc25a4	-
7	103827860	103827865	Hbb-bs	-
5	122453780	122453785	Atp2a2	-
7	103827521	103827526	Hbb-bs	-
17	87137492	87137497	Socs5	+
10	75937700	75937705	Chchd10	+
4	116067092	116067097	Uqcrh	-
7	103826579	103826584	Hbb-bs	-
18	67227850	67227855	Mppe1	-
4	10848845	10848850	Gm12918	+
16	42955812	42955817	BC002163;ZI	+
5	135786333	135786338	Mdh2	+
9	110769150	110769155	Myl3	+
1	135850808	135850813	Tnnt2	+
4	116074979	116074984	Uqcrh	-
18	35805813	35805818	AC141471.2;	+
9	67047855	67047860	Tpm1	-
6	29461472	29461477	Flnc	+
5	145183734	145183739	Atp5j2	-
7	4521010	4521015	Tnni3	-
13	24818029	24818034	Acot13	-
15	38686815	38686820	Atp6v1c1	+
18	77782569	77782574	Atp5a1	+
2	114049336	114049341	Actc1	-
19	9984813	9984818	Fth1	+
14	75846789	75846794	Tpt1	+
4	116069867	116069872	Uqcrh	-
3	89268494	89268499	Slc50a1	-
6	142494100	142494105	Ldhb	-
2	114047426	114047431	Actc1	-
10	53345478	53345483	Pln	+
15	58936313	58936318	Ndufb9;Gm4	+
4	130312437	130312442	Fabp3	+
14	70664563	70664568	Xpo7	-
9	67022973	67022978	Tpm1	-
6	142490434	142490439	Ldhb	-
12	65062687	65062692	Fkbp3	-

13	111426277	111426282	Gpbp1	-
19	9984666	9984671	Fth1	+
3	131242553	131242558	Hadh	-
2	114048275	114048280	Actc1	-
11	31549513	31549518	Ncoa2	+
4	140979177	140979182	Sdhb	+
5	117092291	117092296	Suds3	-
19	9984842	9984847	Fth1	+
4	116069989	116069994	Uqcrh	-
7	48832585	48832590	Csrp3	-
7	122101688	122101693	Ndufab1	-
9	67047876	67047881	Tpm1	-
17	27634196	27634201	Rps10;RPS1	-
12	103321601	103321606	Asb2	-
9	57532294	57532299	Cox5a	+
5	145183741	145183746	Atp5j2	-
13	114351462	114351467	Ndufs4	-
15	81914680	81914685	Aco2	+
5	135786234	135786239	Mdh2	+
6	5485196	5485201	Pdk4	-
1	43123208	43123213	Fhl2	-
8	46210821	46210826	Slc25a4	-
17	33829682	33829687	Ndufa7	+
7	90467741	90467746	Tmem126b	-
11	21559851	21559856	Mdh1	-
4	101466698	101466703	Ak4	+
8	46209488	46209493	Slc25a4	-
5	122101812	122101817	Myl2	+
14	75846703	75846708	Tpt1	+
10	80404206	80404211	Uqcr11	-
6	11900569	11900574	Ndufa4	-
9	57531677	57531682	Cox5a	+
7	12926630	12926635	Rps5	+
10	81565642	81565647	Aes	+
11	21557525	21557530	Mdh1	-
10	75937374	75937379	Chchd10	+
14	75846338	75846343	Tpt1	+
10	86731881	86731886	Hsp90b1	-
2	114047301	114047306	Actc1	-
9	79758518	79758523	Cox7a2	-
1	75367152	75367157	Des	+
1	43123150	43123155	Fhl2	-
10	75937707	75937712	Chchd10	+
15	77017773	77017778	Mb	-
10	81182435	81182440	Eef2	+

2	10056127	10056132	Atp5c1	-
12	111545854	111545859	Eif5	+
10	53344904	53344909	Pln	+
4	140971140	140971145	Sdhb	+
5	135908717	135908722	Ywhag	-
4	141424900	141424905	Hspb7	+
7	4519543	4519548	Tnni3	-
1	171234951	171234956	Ndufs2	-
4	130308812	130308817	Fabp3	+
5	122101019	122101024	Myl2	+
10	128088448	128088453	Atp5b	+
5	124004885	124004890	Vps37b	-
4	140966516	140966521	Sdhb	+
2	155278050	155278055	Map1lc3a	+
7	34204051	34204056	Gpi1	-
13	119366442	119366447	Nnt;Nnt	-
10	94220923	94220928	Ndufa12	+
4	41388736	41388741	Ubap1	+
4	130315312	130315317	Fabp3	+
13	30541201	30541206	Uqcrfs1	-
14	75848168	75848173	Tpt1	+
1	171234979	171234984	Ndufs2	-
17	25058320	25058325	Tmem204	-
5	5782405	5782410	Gm15459	-
15	81912246	81912251	Aco2	+
11	5896757	5896762	Myl7	-
18	77781860	77781865	Atp5a1	+
15	80952258	80952263	Adsl	+
5	122106790	122106795	Myl2	+
10	128088349	128088354	Atp5b	+
2	174462574	174462579	Atp5e	-
1	194959986	194959991	Cd34	+
15	58936267	58936272	Ndufb9;Gm4	+
3	105943738	105943743	Atp5f1	-
19	36112217	36112222	Ankrd1	-
5	135790270	135790275	Mdh2	+
2	76707062	76707067	Ttn	-
11	75599649	75599654	Pitpna	-
1	43128285	43128290	Fhl2	-
5	122454171	122454176	Atp2a2	-
2	108949241	108949246	Gm13910	+
10	80828336	80828341	Oaz1	+
10	75937696	75937701	Chchd10	+
16	4767208	4767213	Cdip1	-
5	122104899	122104904	Myl2	+

11	52243746	52243751	Skp1a	+
8	120673257	120673262	Cox4i1;Gm2(	+
17	13015090	13015095	Sod2	+
4	141425045	141425050	Hspb7	+
6	11905245	11905250	Ndufa4	-
5	122457615	122457620	Atp2a2	-
2	172509579	172509584	Gm14303	-
4	116691870	116691875	Prdx1	+
7	46855581	46855586	Ldha	+
5	122453944	122453949	Atp2a2	-
7	103827645	103827650	Hbb-bs	-
2	75191042	75191047	Mtx2	-
9	59678949	59678954	Pkm	+
5	136966060	136966065	Fis1	+
19	24875870	24875875	Gm10053	+
7	30185989	30185994	Cox7a1	+
11	4702145	4702150	Uqcr10	-
10	128086064	128086069	Atp5b	+
5	122453567	122453572	Atp2a2	-
1	135852031	135852036	Tnnt2	+
1	74267792	74267797	Arpc2	+
1	58591124	58591129	Ndufb3	+
15	77016031	77016036	Mb	-
11	96073146	96073151	Atp5g1	-
9	55486740	55486745	Etfa	-
2	73909909	73909914	Atp5g3	-
4	130313977	130313982	Fabp3	+
10	94220858	94220863	Ndufa12	+
3	32751074	32751079	Ndufb5	+
1	151361266	151361271	Ivns1abp	+
9	53587501	53587506	Acat1	-
5	21803725	21803730	Psmc2	+
3	105943859	105943864	Atp5f1	-
5	122453606	122453611	Atp2a2	-
5	24581534	24581539	Abcf2	-
9	110767974	110767979	Myl3	+
9	64174957	64174962	Rpl4	+
6	129186170	129186175	Clec2d	+
7	142376099	142376104	Gm49369;Ct	-
9	107614272	107614277	Gnai2	-
11	70662039	70662044	Eno3	+
10	80403047	80403052	Uqcr11	-
7	30617047	30617052	Cox6b1	-
4	141424993	141424998	Hspb7	+
7	126795429	126795434	Aldoa	-

7	19414309	19414314	Ckm	+
5	122453730	122453735	Atp2a2	-
10	53344784	53344789	Pln	+
7	128206295	128206300	Cox6a2	-
3	123013738	123013743	Myoz2	-
5	122454094	122454099	Atp2a2	-
6	71214050	71214055	Smyd1	-
10	53343769	53343774	Pln	+
15	76904549	76904554	Rpl8	+
15	35932043	35932048	Cox6c	-
1	171238543	171238548	Ndufs2	-
8	124908743	124908748	Egln1	-
3	10204546	10204551	Fabp4	-
10	80293686	80293691	Rps15	+
18	77782373	77782378	Atp5a1	+
11	31548476	31548481	Ncoa2	+
2	130279394	130279399	Idh3b	-
14	31211338	31211343	Tnnc1	+
8	120674034	120674039	Cox4i1;Gm2(	+
7	103827617	103827622	Hbb-bs	-
10	53345249	53345254	Pln	+
18	77782354	77782359	Atp5a1	+
6	11907364	11907369	Ndufa4	-
10	53345406	53345411	Pln	+
11	21557469	21557474	Mdh1	-
15	81914340	81914345	Aco2	+
10	91118810	91118815	Slc25a3	-
10	128362402	128362407	Cs	+
19	32466351	32466356	Rpl9-ps6	-
11	53429060	53429065	Uqcrq	-
10	117046458	117046463	Lrrc10	+
7	122096719	122096724	Ndufab1	-
14	65975723	65975728	Clu	+
6	142501440	142501445	Ldhb	-
13	91861813	91861818	Ckmt2	-
11	46727896	46727901	Gm12174	+
19	3908935	3908940	Ndufs8	-
15	77015522	77015527	Mb	-
5	5781566	5781571	Pcdh7	-
7	93179586	93179591	Gm15501	-
4	3834632	3834637	Rps20	-
9	40804608	40804613	Hspa8	+
15	77017757	77017762	Mb	-
19	3908925	3908930	Ndufs8	-
15	5120989	5120994	Gm10250	-

13	36204927	36204932	Fars2	+
9	67033889	67033894	Tpm1	-
13	30540727	30540732	Uqcrfs1	-
19	9982870	9982875	Fth1	+
15	82350323	82350328	Ndufa6	-
19	47083550	47083555	Usmg5	-
3	7444855	7444860	Pkia	+
13	119336217	119336222	Nnt;Nnt	-
15	74748378	74748383	Lynx1	-
6	142490405	142490410	Ldhb	-
8	95864922	95864927	Got2	-
14	51906184	51906189	Ndrg2	-
12	8498033	8498038	Rhob	-
10	80403130	80403135	Uqcr11	-
7	4519405	4519410	Tnni3	-
6	32792506	32792511	Chchd3	-
6	24518720	24518725	Ndufa5	-
19	36100195	36100200	Rpp30	+
19	6982661	6982666	Vegfb	-
7	103827552	103827557	Hbb-bs	-
14	21838530	21838535	Vdac2	+
19	44554973	44554978	Ndufb8	-
2	73909311	73909316	Atp5g3	-
5	122453702	122453707	Atp2a2	-
5	129881320	129881325	Chchd2;Phk(	-
8	46209202	46209207	Slc25a4	-
7	48832640	48832645	Csrp3	-
11	93949925	93949930	Nme2;Gm20	-
4	46078350	46078355	Tmod1	+
5	122102751	122102756	Myl2	+
14	32180524	32180529	Timm23	-
5	116408636	116408641	Hspb8	-
4	141423954	141423959	Hspb7	+
7	30624517	30624522	Cox6b1	-
1	169694292	169694297	Rgs5	+
11	75599966	75599971	Gm12338	-
14	75846231	75846236	Tpt1	+
2	114048229	114048234	Actc1	-
10	53343816	53343821	Pln	+
8	11208300	11208305	Col4a1	-
17	66079533	66079538	Ndufv2	-
13	24831390	24831395	Acot13	-
9	96896159	96896164	Gm10123	+
13	8972712	8972717	Gtpbp4	-
1	161243579	161243584	Prdx6	-

8	83435415	83435420	Scoc	-
5	112693295	112693300	Myo18b	-
5	115801668	115801673	Rab35	-
10	53344971	53344976	Pln	+
9	54604647	54604652	Idh3a	+
14	31208359	31208364	Tnnc1	+
16	37647679	37647684	Ndufb4	-
6	73275372	73275377	Suclg1	+
19	4007906	4007911	Ndufv1	-
11	5803462	5803467	Pgam2	-
3	37714811	37714816	Rps23-ps1	+
14	75846743	75846748	Tpt1	+
1	135846734	135846739	Tnnt2	+
9	108945396	108945401	Uqcrc1	+
1	180166788	180166793	Coq8a	-
17	66091921	66091926	Ndufv2	-
11	49678955	49678960	Gm12191;Cr	-
7	30185315	30185320	Cox7a1	+
7	126795416	126795421	Aldoa	-
6	50565122	50565127	Cycs	-
11	120579148	120579153	Arhgdia	-
10	53344191	53344196	Pln	+
1	43123348	43123353	Fhl2	-
19	44553700	44553705	Ndufb8	-
10	97517642	97517647	Dcn	+
6	83358315	83358320	Bola3	+
3	10204480	10204485	Fabp4	-
3	32746490	32746495	Ndufb5	+
11	96073631	96073636	Atp5g1	-
9	108942152	108942157	Uqcrc1	+
2	10056078	10056083	Atp5c1	-
11	21557504	21557509	Mdh1	-
7	43454534	43454539	Etfb;Gm4523	+
14	31211641	31211646	Tnnc1	+
10	75937400	75937405	Chchd10	+
6	99877750	99877755	Gpr27	-
2	35307799	35307804	Gsn	+
15	74748035	74748040	Lynx1	-
7	4521537	4521542	Tnni3	-
7	128206237	128206242	Cox6a2	-
8	46207415	46207420	Slc25a4	-
17	45569729	45569734	Hsp90ab1	-
10	95493704	95493709	Mrpl42	-
9	50756294	50756299	Cryab	+
19	9984834	9984839	Fth1	+

8	46535552	46535557	Acsl1	+
3	105942730	105942735	Atp5f1	-
3	10205330	10205335	Fabp4	-
14	88123035	88123040	Rps3a2	-
9	50596631	50596636	Sdhd	-
13	91858276	91858281	Ckmt2	-
10	91117074	91117079	Slc25a3	-
5	30119716	30119721	Hadha	-
7	141495996	141496001	Chid1	-
10	80403051	80403056	Uqcr11	-
5	24581499	24581504	Abcf2	-
17	25830766	25830771	Stub1	-
19	36115035	36115040	Ankrd1	-
11	21562884	21562889	Mdh1	-
12	32839193	32839198	Nampt	+
1	75362594	75362599	Des	+
12	112641318	112641323	Adssl1	+
17	24849732	24849737	Fahd1	-
14	54941965	54941970	Myh6	-
11	68943461	68943466	Arhgef15	-
10	53345834	53345839	Pln	+
9	50756559	50756564	Cryab	+
5	122458193	122458198	Atp2a2	-
15	78930045	78930050	Lgals1	+
7	28831442	28831447	Ech1	+
7	23947064	23947069	Gm10175	-
9	110769623	110769628	Myl3	+
5	17782692	17782697	Cd36	-
17	12924425	12924430	Tcp1	+
15	81914920	81914925	Aco2	+
7	126797411	126797416	Aldoa	-
2	10059518	10059523	Atp5c1	-
8	94860465	94860470	Polr2c	+
8	68906540	68906545	Lpl	+
9	108948563	108948568	Uqcrc1	+
3	60629479	60629484	Mbnl1	+
2	28933490	28933495	Cfap77	-
2	32707570	32707575	Cdk9	-
19	8707796	8707801	Slc3a2	-
1	16101316	16101321	Rpl7	-
7	43457690	43457695	Etfb;Gm4523	+
13	75645189	75645194	Gm4149	+
5	145183863	145183868	Atp5j2	-
16	31948010	31948015	0610012G03	-
9	107538209	107538214	Tmem115	+

2	30402525	30402530	Crat	-
5	122453885	122453890	Atp2a2	-
7	45719601	45719606	Rpl18	+
5	122458113	122458118	Atp2a2	-
17	72941526	72941531	Lbh	+
11	69943029	69943034	Slc2a4	-
2	104427119	104427124	Hipk3	-
4	148002048	148002053	Nppa	+
4	130315447	130315452	Fabp3	+
7	30555101	30555106	Hspb6	+
11	52388839	52388844	Vdac1	+
2	172509543	172509548	Gm14303	-
8	116982626	116982631	Gcsh	-
16	91928927	91928932	Atp5o	-
4	130314011	130314016	Fabp3	+
6	52546864	52546869	Hibadh	-
18	77781326	77781331	Atp5a1	+
9	65322784	65322789	Clpx	+
11	96073980	96073985	Atp5g1	-
6	11905227	11905232	Ndufa4	-
13	91865303	91865308	Ckmt2	-
10	97513668	97513673	Dcn	+
14	54946342	54946347	Myh6	-
18	77780238	77780243	Atp5a1	+
8	111952888	111952893	Gabarapl2	+
11	70662017	70662022	Eno3	+
8	46207590	46207595	Slc25a4	-
10	57516327	57516332	Serinc1	-
5	5782117	5782122	Gm15459	-
7	51747213	51747218	Gm7336	+
17	26699501	26699506	Atp6v0e	+
19	36732277	36732282	Ppp1r3c	-
6	99877706	99877711	Gpr27	-
7	43454567	43454572	Etfb;Gm4523	+
5	122457317	122457322	Atp2a2	-
7	103827897	103827902	Hbb-bs	-
8	95864205	95864210	Got2	-
11	69994629	69994634	Gabarap	+
10	53345200	53345205	Pln	+
7	19421281	19421286	Ckm	+
11	20335491	20335496	Gm12033	-
2	155819847	155819852	Eif6	-
6	99877940	99877945	Tpt1-ps3	-
13	12270830	12270835	Actn2	-
3	123013600	123013605	Myoz2	-

7	48835611	48835616	Csrp3	-
10	128084401	128084406	Atp5b	+
5	95862457	95862462	Gm5559	+
17	56721996	56722001	Ndufa11	+
13	30541062	30541067	Uqcrfs1	-
16	4480506	4480511	Srl	-
4	116069885	116069890	Uqcrh	-
6	24522748	24522753	Ndufa5	-
3	101579058	101579063	Atp1a1	-
13	91863254	91863259	Ckmt2	-
1	175601557	175601562	Fh1	-
11	101246500	101246505	Ramp2	+
3	146505340	146505345	Gng5	+
3	105943763	105943768	Atp5f1	-
7	103826661	103826666	Hbb-bs	-
17	8297489	8297494	Mpc1	+
9	78478601	78478606	Gm26377;Ee	-
2	25222725	25222730	Tubb4b	-
7	80115195	80115200	Idh2	-
6	55348535	55348540	Aqp1	+
4	138313448	138313453	Pink1	-
9	108947728	108947733	Uqcrc1	+
10	128090059	128090064	Atp5b	+
5	145187196	145187201	Atp5j2	-
17	29298655	29298660	BC004004	+
13	75849434	75849439	Glrx	+
6	90645857	90645862	Slc41a3	+
3	37714761	37714766	Rps23-ps1	+
3	131240984	131240989	Hadh	-
6	126836239	126836244	Ndufa9	-
15	4154948	4154953	Oxct1	+
7	30185983	30185988	Cox7a1	+
14	73590880	73590885	Sucla2	+
3	32751139	32751144	Ndufb5	+
7	30554815	30554820	Hspb6	+
7	30185282	30185287	Cox7a1	+
19	9984564	9984569	Fth1	+
6	87845524	87845529	Cnbp	-
18	67405114	67405119	Afg3l2	-
7	30624571	30624576	Cox6b1	-
3	53692679	53692684	Gm6204	-
2	180036968	180036973	Psma7	-
8	68906726	68906731	Lpl	+
8	46209396	46209401	Slc25a4	-
14	8169773	8169778	Pdhb	-

13	64364076	64364081	Ctsl	-
1	43153212	43153217	Fhl2	-
11	53429013	53429018	Uqcrq	-
13	73320036	73320041	Ndufs6	-
7	28829812	28829817	Ech1	+
13	30540540	30540545	Uqcrfs1	-
13	91859880	91859885	Ckmt2	-
18	74804064	74804069	Acaa2	+
10	53344150	53344155	Pln	+
8	104628401	104628406	Rrad	-
8	95713901	95713906	Ndrg4	+
4	147878732	147878737	Mfn2	-
13	91853400	91853405	Ckmt2	-
2	10056045	10056050	Atp5c1	-
9	108948961	108948966	Uqcrc1	+
7	103827452	103827457	Hbb-bs	-
11	21565852	21565857	Mdh1	-
1	66838319	66838324	Acadl	-
18	77779080	77779085	Atp5a1	+
13	100736199	100736204	Mrps36	-
7	4522405	4522410	Tnni3	-
7	4520523	4520528	Tnni3	-
9	98588234	98588239	Copb2	+
9	64177962	64177967	Rpl4	+
14	63142698	63142703	Ctsb	+
11	69917188	69917193	Eif5a	-
15	35932035	35932040	Cox6c	-
11	21557335	21557340	Mdh1	-
18	60812470	60812475	Cd74	+
6	11905223	11905228	Ndufa4	-
9	21139445	21139450	Cdc37	-
3	105956005	105956010	Atp5f1	-
3	102146393	102146398	Casq2	+
6	83194999	83195004	Dctn1	+
7	141459484	141459489	Pnpla2	+
7	100486717	100486722	Ucp3	+
11	70978042	70978047	C1qbp	-
5	122102715	122102720	Myl2	+
7	142376608	142376613	Gm49369;Ct	-
18	36402587	36402592	Cystm1	+
15	100620265	100620270	Dazap2	+
2	10068619	10068624	Atp5c1	-
1	58591130	58591135	Ndufb3	+
5	30184507	30184512	Hadhb	+
3	142302619	142302624	Pdlim5	-

9	67031462	67031467	Tpm1	-
11	70010725	70010730	Acadvl	-
3	103740003	103740008	Hipk1	-
17	70994682	70994687	Myl12a;Myl1:	-
16	90226282	90226287	Sod1	+
7	89923625	89923630	Hikeshi	-
11	75599781	75599786	Gm12338	-
19	3908877	3908882	Ndufs8	-
2	24974471	24974476	Mrpl41	-
7	132570074	132570079	Oat;Fgfr2	-
17	24722478	24722483	Ndufb10	-
2	91136446	91136451	Mybpc3	+
6	11900531	11900536	Ndufa4	-
2	91130820	91130825	Mybpc3	+
13	38198260	38198265	Dsp	+
5	30119779	30119784	Hadha	-
3	131248546	131248551	Hadh	-
15	81913714	81913719	Aco2	+
1	171129503	171129508	Sdhc	-
7	46855446	46855451	Ldha	+
8	84974051	84974056	Prdx2	+
5	30144162	30144167	Hadha	-
7	120650865	120650870	Uqcrc2	+
5	122103925	122103930	Myl2	+
6	24603713	24603718	Lmod2	+
2	76704753	76704758	Ttn	-
9	67028135	67028140	Tpm1	-
1	135852075	135852080	Tnnt2	+
7	19415022	19415027	Ckm	+
4	108044723	108044728	Podn;Scp2	-
15	99409868	99409873	Tmbim6	+
2	90894795	90894800	Ndufs3	-
2	28934206	28934211	Gm13394;Cf	-
10	94220700	94220705	Ndufa12	+
14	31211354	31211359	Tnnc1	+
4	3834656	3834661	Rps20	-
10	67256069	67256074	Jmjd1c	+
14	21845464	21845469	Vdac2	+
3	86138010	86138015	Rps3a1	-
5	121208423	121208428	Rpl6	+
15	99727891	99727896	Cox14	+
12	112641057	112641062	Adssl1	+
7	4519441	4519446	Tnni3	-
19	9984850	9984855	Fth1	+
7	74275639	74275644	Slco3a1	-

8	84970520	84970525	Prdx2	+
11	6355931	6355936	Ogdh	+
7	28972531	28972536	Eif3k	-
7	45719311	45719316	Rpl18	+
13	91853438	91853443	Ckmt2	-
15	81910487	81910492	Aco2	+
19	32466439	32466444	Rpl9-ps6	-
11	59012372	59012377	Obscn	-
8	3680401	3680406	Trappc5	+
18	24282226	24282231	Galnt1	+
2	76704144	76704149	Ttn	-
6	91464983	91464988	Chchd4	-
2	114052049	114052054	Actc1	-
11	21566710	21566715	Mdh1	-
2	114050434	114050439	Actc1	-
13	30540599	30540604	Uqcrfs1	-
3	101584811	101584816	Atp1a1	-
11	21559211	21559216	Mdh1	-
8	85260906	85260911	Vps35	-
4	119281581	119281586	Ybx1	-
8	83570884	83570889	Ndufb7	+
3	32746500	32746505	Ndufb5	+
12	111669378	111669383	Ckb	-
12	8938476	8938481	Laptm4a	+
11	32296518	32296523	Hba-a2	+
16	38377859	38377864	Popdc2	+
4	147873881	147873886	Mfn2	-
7	19421214	19421219	Ckm	+
7	19419480	19419485	Ckm	+
4	138439817	138439822	Mul1	+
2	163336833	163336838	Jph2	-
18	77782672	77782677	Atp5a1	+
6	125193394	125193399	Mrpl51	+
14	8166212	8166217	Pdhb	-
11	93949824	93949829	Nme2;Gm20	-
10	79977218	79977223	Tmem259	-
2	25222750	25222755	Tubb4b	-
1	66857496	66857501	Acadl	-
15	73751868	73751873	Ptp4a3	+
4	140976182	140976187	Sdhb	+
19	9984921	9984926	Fth1	+
8	120658079	120658084	Gm27021;Er	-
5	115103264	115103269	Rpl37rt	-
11	115417911	115417916	Atp5h	-
17	83502203	83502208	Cox7a2l	-

2	130565063	130565068	Mrps26	+
9	116040991	116040996	Gm9385	-
10	93861584	93861589	Metap2	-
7	114705915	114705920	Gm15500	-
11	115183659	115183664	Nat9	-
7	28832186	28832191	Ech1	+
15	76904961	76904966	Rpl8	+
10	17845261	17845266	Txlnb	+
5	122460698	122460703	Atp2a2	-
6	126832485	126832490	Ndufa9	-
5	145183798	145183803	Atp5j2	-
9	71483464	71483469	Polr2m	-
13	24813519	24813524	BC005537	+
11	54979163	54979168	Anxa6	-
13	114316926	114316931	Ndufs4	-
1	165478243	165478248	Mpc2	+
13	114288800	114288805	Ndufs4	-
13	74328304	74328309	Sdha	-
17	33838250	33838255	Ndufa7	+
15	77915721	77915726	Txn2	-
14	61534140	61534145	Spryd7	-
12	101969547	101969552	Ndufb1-ps	-
5	134238429	134238434	Gtf2i	-
7	141010587	141010592	Ifitm3	-
3	142303104	142303109	Pdlim5	-
14	121284366	121284371	Ipo5	-
1	55078252	55078257	Hspd1	-
3	153925807	153925812	Acadm	-
18	77775832	77775837	Atp5a1	+
16	37649019	37649024	Ndufb4	-
2	156008909	156008914	Ergic3	+
3	105951669	105951674	Atp5f1	-
9	22157964	22157969	Pigyl	+
8	106573028	106573033	Gm10073	-
7	28826060	28826065	Ech1	+
15	89427580	89427585	Chkb;Gm445	-
11	70644346	70644351	Slc25a11	-
11	75599685	75599690	Pitpna	-
9	50753278	50753283	Cryab	+
10	91116901	91116906	Slc25a3	-
11	98384604	98384609	Tcap	+
4	119293785	119293790	Ybx1	-
17	29137079	29137084	Rpl35a-ps3	-
18	77782852	77782857	Atp5a1	+
5	77343213	77343218	Polr2b	+

11	32296540	32296545	Hba-a2	+
2	26921709	26921714	Surf4	-
11	52122530	52122535	Ppp2ca	+
6	127087888	127087893	Tigar	-
16	36052292	36052297	Fam162a	-
4	130314043	130314048	Fabp3	+
1	165480912	165480917	Mpc2	+
7	103813871	103813876	Hbb-bt	-
8	107581247	107581252	Psmd7	-
14	31211657	31211662	Tnnc1	+
5	114250300	114250305	Acacb	+
7	4521354	4521359	Tnni3	-
7	31051725	31051730	Fxyd1	-
7	80094958	80094963	Idh2	-
17	35267304	35267309	H2-D1	+
19	9984655	9984660	Fth1	+
9	53587555	53587560	Acat1	-
9	108912818	108912823	Slc26a6	+
1	24615416	24615421	Gm28661	-
18	60777085	60777090	Rps14	+
8	13242520	13242525	Adprhl1	-
8	72585993	72585998	Tmem38a	+
7	45123507	45123512	Rps11	-
4	57371188	57371193	Gm12537	-
6	125362035	125362040	Tnfrsf1a	+
14	34561409	34561414	Ldb3	-
10	21368597	21368602	Hbs1l	+
7	73776813	73776818	Fam174b	+
10	60301870	60301875	Psap	+
1	16101855	16101860	Rpl7	-
18	84667580	84667585	Cndp2	-
3	123006532	123006537	Myoz2	-
7	19416803	19416808	Ckm	+
16	20722173	20722178	Polr2h	+
12	101969524	101969529	Ndufb1-ps	-
7	80096052	80096057	Idh2	-
9	50344319	50344324	Rpl10-ps3	-
1	171129529	171129534	Sdhc	-
1	171237207	171237212	Ndufs2	-
18	77780297	77780302	Atp5a1	+
1	36530409	36530414	Ankrd23;Gm	-
14	75846378	75846383	Tpt1	+
3	34069313	34069318	Fxr1	+
17	66080843	66080848	Ndufv2	-
14	51905462	51905467	Ndrg2	-

11	70662462	70662467	Eno3	+
10	53345804	53345809	Pln	+
9	67049029	67049034	Tpm1	-
2	28933575	28933580	Cfap77	-
13	91858263	91858268	Ckmt2	-
3	86139039	86139044	Rps3a1	-
6	73269359	73269364	Suclg1	+
10	80254855	80254860	Ndufs7	+
4	140972904	140972909	Sdhb	+
1	24615412	24615417	Gm28661	-
15	76345549	76345554	Cyc1	+
5	104079497	104079502	Sparcl1	-
7	51747631	51747636	Gm7336	+
9	7752236	7752241	Gm10709	+
7	126796245	126796250	Aldoa	-
14	24492098	24492103	Rps24	+
6	5483729	5483734	Pdk4	-
3	79605634	79605639	Etfdh	-
4	130314020	130314025	Fabp3	+
6	142490337	142490342	Ldhb	-
9	59665293	59665298	Pkm	+
4	45105473	45105478	Tomm5	-
2	155389681	155389686	Trp53inp2	+
10	53345014	53345019	Pln	+
15	78929751	78929756	Lgals1	+
5	92392791	92392796	Art3	+
16	84827951	84827956	Atp5j	-
12	32850861	32850866	Nampt	+
3	138546407	138546412	Eif4e	+
8	11198843	11198848	Col4a1	-
7	30626058	30626063	Cox6b1	-
2	181155388	181155393	Eef1a2	-
14	54946941	54946946	Myh6	-
6	99877734	99877739	Gpr27	-
8	46209213	46209218	Slc25a4	-
10	80145281	80145286	Atp5d	+
19	44550275	44550280	Ndufb8;Gm2	-
8	46534596	46534601	Acsl1	+
9	57531713	57531718	Cox5a	+
3	51408178	51408183	Ndufc1	-
10	128090025	128090030	Atp5b	+
19	9984785	9984790	Fth1	+
11	70658234	70658239	Eno3	+
2	35307538	35307543	Gsn	+
7	19247692	19247697	Opa3	+

13	91859299	91859304	Ckmt2	-
7	142376439	142376444	Gm49369;Ct	-
13	111433586	111433591	Gpbp1	-
5	135888106	135888111	Hspb1	+
4	150897312	150897317	Park7	-
4	130312447	130312452	Fabp3	+
3	67394040	67394045	Mlf1	+
7	103827881	103827886	Hbb-bs	-
15	96687573	96687578	Slc38a2	-
4	140979140	140979145	Sdhb	+
7	51747706	51747711	Gm7336	+
19	9984191	9984196	Fth1	+
1	165481084	165481089	Mpc2	+
17	31531155	31531160	Ndufv3	+
9	57532322	57532327	Cox5a	+
5	148504989	148504994	Ubl3	-
6	66875588	66875593	Gm9794	-
13	91863310	91863315	Ckmt2	-
14	21840495	21840500	Vdac2	+
1	131054008	131054013	Mapkapk2	-
8	13174767	13174772	Lamp1	+
6	142490474	142490479	Ldhb	-
17	70996850	70996855	Myl12a;Myl1:	-
13	91865202	91865207	Ckmt2	-
18	77779131	77779136	Atp5a1	+
16	91926897	91926902	Atp5o;Atp5o	-
3	79604082	79604087	Etfdh	-
1	135301881	135301886	Timm17a	-
11	93949857	93949862	Nme2;Gm20	-
2	34775212	34775217	Hspa5	+
3	95904341	95904346	Car14	-
19	23675923	23675928	Gm6563	+
7	4521091	4521096	Tnni3	-
8	13167333	13167338	Lamp1	+
7	48830483	48830488	Csrp3	-
19	24261937	24261942	Fxn	-
2	114049571	114049576	Actc1	-
8	94180274	94180279	Mt1	+
1	165461352	165461357	Mpc2	+
2	10064272	10064277	Atp5c1	-
3	153925863	153925868	Acadm	-
14	31210672	31210677	Tnnc1	+
13	43401409	43401414	Nol7	+
12	113144018	113144023	Crip2	+
5	95862111	95862116	Gm5559	+

9	108947454	108947459	Uqcrc1	+
17	29282216	29282221	BC004004	+
18	77778472	77778477	Atp5a1	+
3	67394009	67394014	Mlf1	+
11	55394657	55394662	Sparc	-
10	53345449	53345454	Pln	+
17	12922164	12922169	Tcp1	+
7	103813443	103813448	Hbb-bt	-
11	70661011	70661016	Eno3	+
2	91135186	91135191	Mybpc3	+
6	126844796	126844801	Ndufa9	-
11	5803481	5803486	Pgam2	-
9	122176370	122176375	Ano10	-
2	148871795	148871800	Cst3	-
13	30540507	30540512	Uqcrfs1	-
5	135786291	135786296	Mdh2	+
18	36742511	36742516	Ndufa2	-
8	102865302	102865307	Gm8730	-
7	103813854	103813859	Hbb-bt	-
3	95947266	95947271	Anp32e	+
9	67028073	67028078	Tpm1	-
6	50565537	50565542	Cycs	-
10	128088345	128088350	Atp5b	+
7	140105776	140105781	Echs1	-
16	36963627	36963632	Fbxo40	-
12	10395347	10395352	Rdh14	+
19	5456590	5456595	Ccdc85b	-
1	171238336	171238341	Ndufs2	-
14	75847310	75847315	Tpt1	+
9	110767992	110767997	Myl3	+
19	9984287	9984292	Fth1	+
15	76906146	76906151	Rpl8	+
11	20335766	20335771	Gm12033	-
18	77781856	77781861	Atp5a1	+
19	40292371	40292376	Pdlim1	-
19	6985367	6985372	Vegfb	-
5	33372744	33372749	Maea	+
9	120495265	120495270	Eif1b	+
11	21557435	21557440	Mdh1	-
11	55394998	55395003	Sparc	-
14	31000098	31000103	Spcs1	-
8	95865102	95865107	Got2	-
10	53345139	53345144	Pln	+
10	128365035	128365040	Coq10a	-
5	3236456	3236461	Gm15772	+

8	70700159	70700164	Jund	+
9	40803281	40803286	Hspa8	+
6	24518810	24518815	Ndufa5	-
9	67031982	67031987	Tpm1	-
19	60867571	60867576	Prdx3	-
12	113145063	113145068	Crip2	+
10	71228293	71228298	Tfam	-
11	31549197	31549202	Ncoa2	+
2	73909869	73909874	Atp5g3	-
18	74798461	74798466	Acaa2	+
5	115103338	115103343	Rpl37rt	-
13	74323079	74323084	Sdha	-
1	131054034	131054039	Mapkapk2	-
17	12911636	12911641	Mrpl18	-
7	132557977	132557982	Oat;Fgfr2	-
2	25573638	25573643	Mamdc4;Php	-
10	45875890	45875895	Gpx4-ps2	+
11	93952855	93952860	Nme2;Gm20	-
14	76506582	76506587	Tsc22d1	+
11	55395128	55395133	Sparc	-
14	31209805	31209810	Tnnc1	+
11	21562945	21562950	Mdh1	-
14	77826384	77826389	Dnajc15	-
11	6106060	6106065	Nudcd3	-
7	43452354	43452359	Etfb;Gm452	+
17	7302618	7302623	Rps6ka2	+
1	24615424	24615429	Gm28661	-
5	129887208	129887213	Chchd2;Phk	-
19	6398106	6398111	Pygm	+
2	90894801	90894806	Ndufs3	-
19	43504540	43504545	Got1	-
7	93179347	93179352	Gm15501	-
7	126796863	126796868	Aldoa	-
6	50565494	50565499	Cycs	-
18	77782689	77782694	Atp5a1	+
4	116066993	116066998	Uqcrh	-
19	4008381	4008386	Ndufv1	-
9	107301517	107301522	Cish	+
12	54861189	54861194	Cfl2	-
11	75599737	75599742	Pitpna	-
2	39004123	39004128	Rpl35	-
16	4764733	4764738	Hmox2	+
5	145128003	145128008	Arpc1b	+
6	24522692	24522697	Ndufa5	-
19	9829803	9829808	AC132253.9	-

8	84970149	84970154	Prdx2	+
9	106431478	106431483	Rpl29	+
9	40804193	40804198	Hspa8	+
5	76967285	76967290	Paics	+
11	21323222	21323227	Ugp2	-
3	102146449	102146454	Casq2	+
12	103321403	103321408	Asb2	-
12	21390935	21390940	Ywhaq	-
12	110898491	110898496	Rps19-ps6;T	+
1	55078062	55078067	Hspd1	-
11	70661433	70661438	Eno3	+
15	5120706	5120711	Gm10250	-
19	7215354	7215359	Cox8a	-
2	130658603	130658608	Ddrgk1	-
17	17345962	17345967	Oaz1-ps;AC	-
2	120506879	120506884	Zfp106	-
11	46728497	46728502	Gm12174	+
4	141425139	141425144	Hspb7	+
9	67047850	67047855	Tpm1	-
3	101576463	101576468	Atp1a1	-
5	24581840	24581845	Gm10221	-
1	58591106	58591111	Ndufb3	+
11	52383869	52383874	Vdac1	+
10	53345887	53345892	Pln	+
14	73364616	73364621	Itm2b	-
4	141424344	141424349	Hspb7	+
6	50565209	50565214	Cycs	-
10	4403198	4403203	Rmnd1	-
5	122459491	122459496	Atp2a2	-
17	24724262	24724267	Ndufb10	-
15	35937280	35937285	Cox6c	-
1	120116427	120116432	Dbi	-
11	21571773	21571778	Mdh1	-
5	45504836	45504841	Lap3	+
19	44552810	44552815	Ndufb8;Gm2	-
14	105682127	105682132	Gm10076	+
19	43500034	43500039	Got1	-
7	137467984	137467989	Glrx3	+
5	129128463	129128468	Rps16-ps2	-
7	126795405	126795410	Aldoa	-
5	30180364	30180369	Hadhb	+
4	134329390	134329395	Trim63	+
12	65062681	65062686	Fkbp3	-
19	5040609	5040614	B4gat1;AC12	+
7	122093673	122093678	Ndufab1	-

1	36693268	36693273	Cox5b	+
15	81914321	81914326	Aco2	+
14	54943203	54943208	Myh6	-
6	124812416	124812421	Tpi1	-
11	98384556	98384561	Tcap	+
12	100121091	100121096	Psmc1	+
10	43533085	43533090	1700021F05I	-
13	74322410	74322415	Sdha	-
8	83572409	83572414	Tecr	-
13	12280460	12280465	Actn2	-
19	6982726	6982731	Vegfb	-
11	70661965	70661970	Eno3	+
8	46208439	46208444	Slc25a4	-
14	65979852	65979857	Clu	+
10	60295056	60295061	Psap	+
14	51905295	51905300	Ndrg2	-
14	73362916	73362921	Itm2b	-
9	78480341	78480346	Eef1a1	-
13	114351476	114351481	Ndufs4	-
5	122454073	122454078	Atp2a2	-
10	53345060	53345065	Pln	+
17	66091899	66091904	Ndufv2	-
6	32792675	32792680	Chchd3	-
11	30506366	30506371	Acyp2	-
5	129887260	129887265	Chchd2;Phk(	-
3	35959997	35960002	Mccc1	-
6	11905254	11905259	Ndufa4	-
18	36753525	36753530	Ik	+
15	76344921	76344926	Cyc1	+
2	28934087	28934092	Gm13394;Cf	-
1	24615056	24615061	Gm28661	-
12	54179595	54179600	Egln3	-
4	45105464	45105469	Tomm5	-
17	70994403	70994408	Myl12a;Myl1:	-
7	30563601	30563606	U2af1l4;Gm4	+
4	40270757	40270762	Ndufb6	-
8	68906775	68906780	Lpl	+
6	124716742	124716747	Phb2	+
10	78162576	78162581	D10Jhu81e	-
5	30920950	30920955	Emilin1	+
3	94886631	94886636	Psmb4	-
10	91116886	91116891	Slc25a3	-
11	70664864	70664869	Spag7	-
11	40748679	40748684	Ccng1	-
7	80095106	80095111	Idh2	-

9	55464798	55464803	Etfa	-
11	70661105	70661110	Eno3	+
1	63147164	63147169	Ndufs1	-
5	33027371	33027376	Ywhah	+
7	28831857	28831862	Ech1	+
16	38352694	38352699	Cox17;Gm2	+
4	130315409	130315414	Fabp3	+
17	24721143	24721148	Rps2	+
17	70996160	70996165	Myl12a;Myl1	-
4	91851548	91851553	Gm12669	-
10	94220916	94220921	Ndufa12	+
5	30119452	30119457	Hadha	-
11	77468950	77468955	Coro6	+
14	34561495	34561500	Ldb3	-
2	30172902	30172907	Endog	+
15	81914658	81914663	Aco2	+
9	108947890	108947895	Uqcrc1	+
19	59322664	59322669	Rps12-ps3	+
2	150618971	150618976	Acss1	-
14	25700012	25700017	Ppif	+
7	30555133	30555138	Hspb6	+
4	46172003	46172008	Ncbp1	+
11	115418437	115418442	Atp5h	-
4	15919886	15919891	Decr1	-
16	31253734	31253739	Ppp1r2	-
10	29699016	29699021	Gm10275	-
1	75215565	75215570	Tuba4a	-
9	55488853	55488858	Etfa	-
17	70994670	70994675	Myl12a;Myl1	-
19	46543516	46543521	Arl3	-
3	86141294	86141299	Rps3a1	-
10	128359229	128359234	Cs	+
10	81565523	81565528	Aes	+
6	126827574	126827579	Ndufa9	-
4	40732781	40732786	Dnaja1	+
9	67027983	67027988	Tpm1	-
5	122104841	122104846	Myl2	+
16	96228774	96228779	Sh3bgr	+
6	128430801	128430806	Fkbp4	-
11	6355600	6355605	Ogdh	+
11	32283723	32283728	Hba-a1	+
5	122461579	122461584	Atp2a2	-
2	28933777	28933782	Gm13394;Cf	-
5	122457307	122457312	Atp2a2	-
17	45568977	45568982	Hsp90ab1	-

2	10059632	10059637	Atp5c1	-
11	100409053	100409058	P3h4	-
7	66050303	66050308	Pcsk6;Gm45	+
7	103813891	103813896	Hbb-bt	-
8	13173927	13173932	Lamp1	+
6	136875732	136875737	Mgp	-
10	91118873	91118878	Slc25a3	-
6	124811018	124811023	Tpi1	-
10	91116941	91116946	Slc25a3	-
3	135466407	135466412	Ube2d3	+
2	36036400	36036405	Ndufa8	-
5	110277674	110277679	Pxmp2	-
2	163625482	163625487	Serinc3	-
14	21843924	21843929	Vdac2	+
10	128912180	128912185	Cd63	+
3	105943051	105943056	Atp5f1	-
9	67031495	67031500	Tpm1	-
2	38640184	38640189	Psmb7	-
2	35314206	35314211	Stom	-
5	129881202	129881207	Chchd2;Phk	-
5	129882551	129882556	Chchd2;Phk	-
17	25184995	25185000	BC003965	+
17	66495433	66495438	Rab12	-
2	127298883	127298888	Stard7	+
17	24864233	24864238	Hagh	+
1	45348657	45348662	Col3a1	+
1	55077962	55077967	Hspd1	-
12	84376547	84376552	Entpd5	-
10	80145288	80145293	Atp5d	+
7	43456497	43456502	Etfb;Gm4523	+
11	58994348	58994353	Obscn	-
7	88530505	88530510	Rps13-ps2	-
2	108950007	108950012	Gm13910	+
2	76704426	76704431	Ttn	-
10	128085440	128085445	Atp5b	+
18	35254458	35254463	Ctnna1	+
8	23148435	23148440	Ank1	+
19	5426490	5426495	AI837181	+
15	98135665	98135670	Asb8	-
9	53582043	53582048	Acat1	-
10	53344895	53344900	Pln	+
1	43123455	43123460	Fhl2	-
9	56136178	56136183	Tspan3	-
10	81182294	81182299	Eef2	+
19	44552770	44552775	Ndufb8;Gm2	-

14	37120994	37120999	Ghitm	-
17	46017926	46017931	Vegfa	-
3	104656977	104656982	Slc16a1	+
13	49196039	49196044	Ninj1	+
1	43123120	43123125	Fhl2	-
8	83571584	83571589	Ndufb7	+
12	85133732	85133737	Dlst	+
2	4938350	4938355	Phyh	+
3	142304368	142304373	Pdlim5	-
5	136248244	136248249	Cux1	-
18	74793326	74793331	Acaa2	+
17	35832551	35832556	Flot1	+
10	81182179	81182184	Eef2	+
17	24882739	24882744	Nubp2	-
8	94863637	94863642	Polr2c	+
14	73365840	73365845	Itm2b	-
5	122453877	122453882	Atp2a2	-
2	35304612	35304617	Gsn	+
5	104079316	104079321	Sparcl1	-
18	74804053	74804058	Acaa2	+
4	116693830	116693835	Prdx1	+
14	34561490	34561495	Ldb3	-
2	174345511	174345516	Gnas	+
9	79758502	79758507	Cox7a2	-
5	144255379	144255384	Bri3	+
12	76773292	76773297	Churc1;Fntb	+
8	46535713	46535718	Acsl1	+
15	76904614	76904619	Rpl8	+
3	32747768	32747773	Ndufb5	+
19	59322705	59322710	Rps12-ps3	+
7	19414984	19414989	Ckm	+
17	45392036	45392041	Cdc5l	-
11	70661401	70661406	Eno3	+
16	84828496	84828501	Atp5j	-
16	84831497	84831502	Atp5j	-
1	90215228	90215233	Ackr3	+
10	53344039	53344044	Pln	+
7	111075336	111075341	Eif4g2	-
10	19609781	19609786	Ifngr1	+
9	108337114	108337119	Rhoa	+
3	24333295	24333300	Gm7536	+
8	102865782	102865787	Gm8730	-
8	46207366	46207371	Slc25a4	-
15	77915589	77915594	Txn2	-
1	171239366	171239371	Ndufs2	-

10	81123680	81123685	Map2k2	+
11	70011721	70011726	Acadvl	-
17	23824840	23824845	Elob	-
5	5781802	5781807	Gm15459	-
11	106781616	106781621	Ddx5	-
17	48419496	48419501	Apobec2	-
11	78254916	78254921	Sdf2	+
7	99479926	99479931	Rps3	-
11	88211207	88211212	Mrps23	+
8	13465689	13465694	Gas6	-
3	130696119	130696124	Ostc	-
11	94339649	94339654	Ankrd40	+
19	47864727	47864732	Gsto1	+
13	74322827	74322832	Sdha	-
1	151364196	151364201	Ivns1abp	+
10	80294011	80294016	Rps15	+
11	70010207	70010212	Acadvl	-
4	140966568	140966573	Sdhb	+
2	4922875	4922880	Phyh	+
18	77779096	77779101	Atp5a1	+
5	116409204	116409209	Hspb8	-
7	103827877	103827882	Hbb-bs	-
3	86138493	86138498	Rps3a1	-
14	20320857	20320862	Ecd	-
19	9982901	9982906	Fth1	+
4	140977475	140977480	Sdhb	+
3	60629196	60629201	Mbnl1	+
4	43447551	43447556	Tesk1	+
5	30121497	30121502	Hadha	-
2	103021359	103021364	Pdhx	-
18	77780994	77780999	Atp5a1	+
2	181148720	181148725	Eef1a2	-
19	8794327	8794332	Polr2g	-
7	143514057	143514062	Nap1l4	-
14	101934539	101934544	Lmo7	+
6	83358220	83358225	Bola3	+
17	72941181	72941186	Lbh	+
17	24895666	24895671	Mrps34	+
11	40748602	40748607	Ccng1	-
11	5803028	5803033	Pgam2	-
5	145184533	145184538	Atp5j2	-
8	120674001	120674006	Cox4i1;Gm2(	+
14	66084941	66084946	Ephx2	-
1	75367322	75367327	Des	+
5	17813052	17813057	Cd36	-

1	63163707	63163712	Ndufs1	-
10	128086052	128086057	Atp5b	+
19	9984646	9984651	Fth1	+
12	85343226	85343231	Tmed10	-
19	43499892	43499897	Got1	-
2	76704953	76704958	Ttn	-
6	32792531	32792536	Chchd3	-
14	25700237	25700242	Ppif	+
1	165481031	165481036	Mpc2	+
4	127247125	127247130	Smim12	+
10	45875391	45875396	Gpx4-ps2	+
15	82350296	82350301	Ndufa6	-
3	79618379	79618384	Etfdh	-
1	171246118	171246123	Ndufs2	-
9	110767968	110767973	Myl3	+
14	29027735	29027740	Lrtm1	+
14	105682003	105682008	Gm10076	+
17	83502414	83502419	Cox7a2l	-
7	25721594	25721599	Hnrnpul1	-
3	152237446	152237451	Nexn	-
5	108433768	108433773	Atp5k	-
7	137459503	137459508	Glrx3	+
15	4154774	4154779	Oxct1	+
15	5121105	5121110	Gm10250	-
10	80256776	80256781	Ndufs7	+
6	128313916	128313921	Rpl18-ps2	+
15	82351060	82351065	Ndufa6	-
15	81910415	81910420	Aco2	+
1	135850735	135850740	Tnnt2	+
8	70509339	70509344	Uba52;Kxd1	-
19	9982756	9982761	Fth1	+
14	25700075	25700080	Ppif	+
12	17550705	17550710	Odc1	+
11	117813534	117813539	Syngr2;Gm2	+
17	46147902	46147907	Mad2l1bp	-
7	46851039	46851044	Ldha	+
18	80296223	80296228	Kcng2	-
11	100321832	100321837	Eif1	+
11	22956523	22956528	Gm28048;Cc	-
2	120090485	120090490	Ehd4	-
12	113144059	113144064	Crip2	+
1	75215618	75215623	Tuba4a	-
3	85653880	85653885	Gatb	+
9	50596604	50596609	Sdhd	-
7	126796875	126796880	Aldoa	-

1	24613157	24613162	Gm28438	-
13	74322289	74322294	Sdha	-
14	73362672	73362677	Itm2b	-
9	59679353	59679358	Pkm	+
18	61259595	61259600	Rps2-ps10	-
13	86045844	86045849	Cox7c	-
11	53428966	53428971	Uqcrq	-
5	21738885	21738890	Pmpcb	+
17	31531191	31531196	Ndufv3	+
9	78470723	78470728	Mto1	+
8	68906916	68906921	Lpl	+
15	80260783	80260788	Rps19bp1	-
2	181508173	181508178	Tpd52l2	+
15	81915019	81915024	Aco2	+
4	116067031	116067036	Uqcrh	-
9	110769110	110769115	Myl3	+
1	95499149	95499154	Gm15427	+
7	81342786	81342791	Rps17	-
17	73884019	73884024	Xdh	-
15	73757027	73757032	Ptp4a3	+
12	31331945	31331950	Dld	-
4	126687056	126687061	Psmb2	+
8	70305664	70305669	Cope	+
19	9984944	9984949	Fth1	+
5	31622551	31622556	Mrpl33;Gm4	+
6	83351326	83351331	Bola3	+
2	151561222	151561227	Fkbp1a	+
15	76344873	76344878	Cyc1	+
5	104087089	104087094	Sparcl1	-
15	98125035	98125040	Pfkm	+
11	70010243	70010248	Acadvl	-
7	102111062	102111067	Art1	+
17	46018128	46018133	Vegfa	-
14	27403619	27403624	Arhgef3	+
3	36449069	36449074	Anxa5	-
8	114151751	114151756	Nudt7	+
10	80255995	80256000	Ndufs7	+
13	54590981	54590986	Higd2a	+
3	101576289	101576294	Atp1a1	-
6	5485374	5485379	Pdk4	-
14	19814572	19814577	Rtraf	-
3	67397850	67397855	Mlf1	+
6	17340814	17340819	Cav1	+
13	91853478	91853483	Ckmt2	-
12	65062466	65062471	Fkbp3	-

2	73908512	73908517	Atp5g3	-
5	17782919	17782924	Cd36	-
11	59012817	59012822	Obscn	-
15	74995378	74995383	Ly6a	-
5	33248392	33248397	Ctbp1	-
2	174122152	174122157	Npepl1	+
3	131233739	131233744	Hadh	-
6	133106384	133106389	Smim10l1	+
5	74206355	74206360	Scfd2	-
1	66837028	66837033	Acadl	-
7	103813512	103813517	Hbb-bt	-
6	131367813	131367818	Ybx3	-
9	57531769	57531774	Cox5a	+
14	75846321	75846326	Tpt1	+
2	35307814	35307819	Gsn	+
9	64175199	64175204	Rpl4	+
19	21281638	21281643	Zfand5	+
12	56373476	56373481	Ralgapa1	-
16	20128768	20128773	Klhl24	+
17	44279994	44279999	Clic5	+
13	17989609	17989614	Gm48778;Ya	-
6	125193386	125193391	Mrpl51	+
2	126709287	126709292	Usp50	-
13	30540719	30540724	Uqcrfs1	-
1	120119920	120119925	Dbi	-
5	140442955	140442960	Eif3b	+
10	128090038	128090043	Atp5b	+
12	103325174	103325179	Asb2	-
11	6355631	6355636	Ogdh	+
6	73276787	73276792	Suclg1	+
16	38377985	38377990	Popdc2	+
10	78162512	78162517	D10Jhu81e	-
11	21557499	21557504	Mdh1	-
1	43153225	43153230	Fhl2	-
13	114316947	114316952	Ndufs4	-
11	98384203	98384208	Tcap	+
4	15929796	15929801	Decr1	-
4	152332387	152332392	Rpl22	+
7	120649396	120649401	Uqcrc2	+
1	143741653	143741658	Glrx2	+
9	55464854	55464859	Etfa	-
9	71548724	71548729	Myzap	-
1	45348600	45348605	Col3a1	+
7	126489874	126489879	Tufm	+
11	75765269	75765274	Ywhae	+

14	76507373	76507378	Tsc22d1	+
5	17797037	17797042	Cd36	-
17	56259267	56259272	Fem1a	+
15	99409447	99409452	Tmbim6	+
7	12926660	12926665	Rps5	+
18	74806102	74806107	Acaa2	+
1	51302616	51302621	Cavin2	+
4	48672858	48672863	Cavin4	+
8	120674076	120674081	Cox4i1;Gm2(	+
5	136966144	136966149	Fis1	+
3	36453495	36453500	Anxa5	-
8	94854397	94854402	Coq9	+
18	36404172	36404177	Pfdn1	-
6	88827655	88827660	Mgll	+
11	31549235	31549240	Ncoa2	+
1	63150082	63150087	Ndufs1	-
5	65595077	65595082	Ube2k	+
13	23692182	23692187	Hist1h2bc	+
19	59322495	59322500	Rps12-ps3	+
7	46855310	46855315	Ldha	+
2	150831161	150831166	Pygb	+
3	152237316	152237321	Nexn	-
2	25222639	25222644	Tubb4b	-
9	40802849	40802854	Hspa8	+
5	24581545	24581550	Abcf2	-
15	51797651	51797656	Eif3h	-
14	63143132	63143137	Ctsb	+
15	77015648	77015653	Mb	-
11	54981714	54981719	Anxa6	-
2	76704768	76704773	Ttn	-
18	74806015	74806020	Acaa2	+
3	79604048	79604053	Etfdh	-
17	66083477	66083482	Ndufv2	-
12	111961471	111961476	Atp5mpl	-
15	76344486	76344491	Cyc1	+
4	15918973	15918978	Decr1	-
1	75215779	75215784	Tuba4a	-
1	165770711	165770716	Creg1	+
10	81565653	81565658	Aes	+
2	174461131	174461136	Atp5e	-
4	57951864	57951869	Txn1	-
2	127297382	127297387	Stard7	+
3	94886191	94886196	Psmb4	-
6	133106491	133106496	Smim10l1	+
17	29277860	29277865	BC004004	+

11	100321920	100321925	Eif1	+
7	12926439	12926444	Rps5	+
10	53344276	53344281	Pln	+
18	77775849	77775854	Atp5a1	+
7	120659074	120659079	Uqcrc2	+
15	81913275	81913280	Aco2	+
3	79603967	79603972	Etfdh	-
5	125388006	125388011	Ubc	-
16	56701077	56701082	Tfg	-
6	119924116	119924121	Wnk1	-
14	54948303	54948308	Myh6	-
1	63143786	63143791	Ndufs1	-
2	163725897	163725902	Pkig	+
14	24491946	24491951	Rps24	+
17	24883062	24883067	Nubp2	-
6	86334032	86334037	Gm10443	+
19	3909209	3909214	Ndufs8	-
7	45122856	45122861	Rps11	-
1	151363385	151363390	Ivns1abp	+
9	121712756	121712761	Ss18l2	+
5	21743410	21743415	Pmpcb	+
3	105942921	105942926	Atp5f1	-
17	66083487	66083492	Ndufv2	-
1	36693203	36693208	Cox5b	+
4	116692898	116692903	Prdx1	+
1	165478265	165478270	Mpc2	+
3	102146254	102146259	Casq2	+
13	74339079	74339084	Sdha	-
7	88530729	88530734	Rps13-ps2	-
14	75846691	75846696	Tpt1	+
3	24333302	24333307	Gm7536	+
5	135790183	135790188	Mdh2	+
1	175604030	175604035	Fh1	-
10	61692659	61692664	Sar1a	+
17	31521601	31521606	Ndufv3	+
10	75937432	75937437	Chchd10	+
14	76507203	76507208	Tsc22d1	+
11	95678095	95678100	Phb	+
2	29776484	29776489	Trub2	-
7	19416783	19416788	Ckm	+
2	34775989	34775994	Hspa5	+
10	128089001	128089006	Atp5b	+
1	66841636	66841641	Acadl	-
5	124493697	124493702	Rilpl1	-
9	65690020	65690025	Oaz2	+

11	43424796	43424801	Pttg1	-
17	24722446	24722451	Ndufb10	-
6	66875592	66875597	Gm9794	-
10	91116969	91116974	Slc25a3	-
16	4055846	4055851	Trap1	-
1	24612867	24612872	Gm28438	-
14	60780029	60780034	C1qtnf9	+
8	72321006	72321011	Klf2	+
7	141471283	141471288	Cd151	+
10	128086095	128086100	Atp5b	+
10	128083380	128083385	Atp5b	+
9	64178480	64178485	Rpl4	+
10	45875474	45875479	Gpx4-ps2	+
3	97690663	97690668	Pde4dip	-
3	88729513	88729518	Rit1	+
6	124936106	124936111	Mlf2	+
17	24720717	24720722	Rps2	+
4	141424248	141424253	Hspb7	+
14	31211405	31211410	Tnnc1	+
3	105954024	105954029	Atp5f1	-
17	24722421	24722426	Ndufb10	-
7	30624563	30624568	Cox6b1	-
1	175601490	175601495	Fh1	-
5	36795382	36795387	Mrfap1	-
14	76506716	76506721	Tsc22d1	+
12	113144423	113144428	Crip2	+
10	128085549	128085554	Atp5b	+
1	135306110	135306115	Timm17a	-
9	79755432	79755437	Cox7a2	-
5	69516903	69516908	Yipf7	-
9	40803080	40803085	Hspa8	+
7	140112446	140112451	Echs1	-
13	74322339	74322344	Sdha	-
14	31211616	31211621	Tnnc1	+
17	46548993	46548998	Srf	-
11	69916913	69916918	Eif5a	-
7	28830235	28830240	Ech1	+
4	40270744	40270749	Ndufb6	-
8	70510128	70510133	Uba52;Kxd1	-
13	91855796	91855801	Ckmt2	-
19	6298885	6298890	Ehd1	+
18	77774104	77774109	Atp5a1	+
4	41190982	41190987	Ube2r2	+
19	9984215	9984220	Fth1	+
1	36530324	36530329	Ankrd23;Gm	-

7	43452808	43452813	Etfb;Gm4523	+
6	39598346	39598351	Ndufb2	+
10	128362067	128362072	Cs	+
7	128546493	128546498	Bag3	+
7	122093661	122093666	Ndufab1	-
3	90614237	90614242	S100a6	+
7	143513657	143513662	Nap1l4	-
2	174461086	174461091	Atp5e	-
1	45347177	45347182	Col3a1	+
1	75367376	75367381	Des	+
6	97185713	97185718	Uba3	-
11	75765051	75765056	Ywhae	+
7	141191184	141191189	Hras	-
4	148001783	148001788	Nppa	+
17	45569627	45569632	Hsp90ab1	-
11	101288863	101288868	Becn1	-
6	32792821	32792826	Chchd3	-
17	26699505	26699510	Atp6v0e	+
6	97248240	97248245	Lmod3	-
7	103813636	103813641	Hbb-bt	-
3	79609953	79609958	Etfdh	-
5	69517030	69517035	Yipf7	-
5	143902729	143902734	Aimp2	-
7	28832178	28832183	Ech1	+
6	140746956	140746961	Rpl38-ps2	-
8	83572025	83572030	Tecr	-
2	28934652	28934657	Cfap77	-
12	85128361	85128366	Dlst	+
7	28305752	28305757	Timm50	-
5	69517060	69517065	Yipf7	-
13	54591021	54591026	Higd2a	+
13	12278795	12278800	Actn2	-
2	39015178	39015183	Arpc5l	+
5	74198475	74198480	Rasl11b	+
11	4702029	4702034	Uqcr10	-
18	77782585	77782590	Atp5a1	+
13	73319956	73319961	Ndufs6	-
10	78162693	78162698	D10Jhu81e	-
7	19411121	19411126	Ckm	+
4	116067003	116067008	Uqcrh	-
10	128361704	128361709	Cs	+
15	81911943	81911948	Aco2	+
7	30107409	30107414	Zfp260	+
9	78478837	78478842	Eef1a1	-
4	140973764	140973769	Sdhb	+

5	30119639	30119644	Hadha	-
19	44555043	44555048	Ndufb8	-
5	30174840	30174845	Hadhb	+
7	30624584	30624589	Cox6b1	-
9	106429991	106429996	Rpl29	+
9	106429973	106429978	Rpl29	+
14	34344754	34344759	Glud1	+
11	116105927	116105932	Trim47	-
5	3236468	3236473	Gm15772	+
1	135848048	135848053	Tnnt2	+
5	125386872	125386877	Ubc	-
17	29326970	29326975	Pi16	+
7	118115124	118115129	Rps15a	-
15	9109167	9109172	Nadk2	+
11	107497639	107497644	Psmd12	+
2	181149921	181149926	Eef1a2	-
3	116494123	116494128	Rtca	-
3	102145734	102145739	Casq2	+
1	92439773	92439778	Ndufa10	-
11	95027204	95027209	Pdk2	-
15	90764280	90764285	Rpl31-ps8	-
14	76507129	76507134	Tsc22d1	+
6	34309967	34309972	Akr1b3	-
9	59679142	59679147	Pkm	+
4	59612751	59612756	Hsdl2	+
1	24613459	24613464	Gm28437	-
3	60628967	60628972	Mbnl1	+
6	82725369	82725374	Hk2	-
7	122090902	122090907	Ndufab1	-
2	114049190	114049195	Actc1	-
8	70509526	70509531	Uba52;Kxd1	-
5	135889071	135889076	Hspb1	+
3	79603899	79603904	Etfdh	-
17	43456406	43456411	Adgrf5	+
14	54944513	54944518	Myh6	-
1	135851721	135851726	Tnnt2	+
2	172509522	172509527	Gm14303	-
14	61221600	61221605	Sgcg	-
1	160199620	160199625	Mrps14	+
6	142490386	142490391	Ldhb	-
14	54944459	54944464	Myh6	-
5	5782609	5782614	Gm15459	-
13	21501334	21501339	Gm11273	-
3	104794040	104794045	Rhoc	+
5	122501796	122501801	Atp2a2	-

10	61429121	61429126	Eif4ebp2	-
8	71369293	71369298	Use1	+
6	92174939	92174944	Mrps25	-
9	50751397	50751402	Hspb2	-
2	152737198	152737203	Id1	+
2	155250066	155250071	Dynlrb1	+
4	107904234	107904239	Cpt2	-
9	40803161	40803166	Hspa8	+
1	135852212	135852217	Tnnt2	+
3	97693964	97693969	Pde4dip	-
6	39599401	39599406	Ndufb2	+
3	95988981	95988986	Plekho1	-
10	117046162	117046167	Lrrc10	+
8	119957422	119957427	Usp10;Gm2(	+
2	122150944	122150949	B2m	+
7	19421482	19421487	Ckm	+
14	54614226	54614231	Psmb5	-
14	75848205	75848210	Tpt1	+
7	28788442	28788447	Sirt2	+
2	71247849	71247854	Dync1i2	+
19	24280460	24280465	Fxn	-
8	70508304	70508309	Uba52;Kxd1	-
17	66089233	66089238	Ndufv2	-
7	141447988	141447993	Rplp2	+
10	79711938	79711943	Bsg	+
11	49678721	49678726	Gm12191;Cr	-
11	58998005	58998010	Obscn	-
3	146505409	146505414	Gng5	+
3	32751433	32751438	Ndufb5	+
10	78269223	78269228	Agpat3	-
16	36044004	36044009	Fam162a	-
2	36036509	36036514	Ndufa8	-
8	104628081	104628086	Rrad	-
18	84879507	84879512	Cyb5a	+
4	141424545	141424550	Hspb7	+
8	94854417	94854422	Coq9	+
4	43663998	43664003	Hint2	+
8	119410709	119410714	Mlycd	+
1	165480957	165480962	Mpc2	+
16	95982138	95982143	Psmg1	-
7	114705995	114706000	Gm15500	-
12	110858108	110858113	Wdr20	-
11	120488120	120488125	Mrpl12	+
19	7574984	7574989	Pla2g16	+
5	21757004	21757009	Pmpcb	+

1	75215932	75215937	Tuba4a	-
18	25112554	25112559	Fhod3	+
1	135304457	135304462	Timm17a	-
5	30134120	30134125	Hadha	-
2	26396980	26396985	Pmpca	+
14	20703162	20703167	Chchd1	+
8	68906825	68906830	Lpl	+
9	40805164	40805169	Hspa8	+
11	52388550	52388555	Vdac1	+
2	36039767	36039772	Ndufa8	-
10	97513689	97513694	Dcn	+
5	122405971	122405976	Arpc3	+
13	11553319	11553324	Ryr2	-
8	106573228	106573233	Gm10073	-
7	142385581	142385586	Ctsd	-
5	116408917	116408922	Hspb8	-
7	34204127	34204132	Gpi1	-
6	50565271	50565276	Cycs	-
11	75599828	75599833	Gm12338	-
14	120947396	120947401	Ipo5	+
2	112467272	112467277	Emc7	+
16	91425203	91425208	Il10rb	+
8	34170594	34170599	Saraf	+
1	66838306	66838311	Acadl	-
3	57287536	57287541	Tm4sf1	-
15	25972709	25972714	Retreg1	+
5	88659784	88659789	Grsf1	-
5	139338056	139338061	Cox19	-
9	78478795	78478800	Eef1a1	-
8	95870786	95870791	Got2	-
19	45792577	45792582	Kcnip2	-
11	21557484	21557489	Mdh1	-
9	40804556	40804561	Hspa8	+
7	31051695	31051700	Fxyd1	-
17	46773209	46773214	2310039H08	+
5	35652248	35652253	Htra3	-
17	56618352	56618357	Lonp1	-
16	84831419	84831424	Atp5j	-
7	103813875	103813880	Hbb-bt	-
7	27306615	27306620	Ltbp4	-
3	53692565	53692570	Gm6204	-
2	76704025	76704030	Ttn	-
4	15918749	15918754	Decr1	-
4	98355518	98355523	Tm2d1	-
16	44162277	44162282	Naa50	+

14	63142336	63142341	Ctsb	+
10	128088955	128088960	Atp5b	+
4	134525656	134525661	Mtfr1l	-
19	5716154	5716159	Ehbp1l1	-
16	84831356	84831361	Atp5j	-
16	90220906	90220911	Sod1	+
17	25782388	25782393	Narfl	+
12	85343160	85343165	Tmed10	-
7	80099062	80099067	Idh2	-
10	81565613	81565618	Aes	+
10	128088340	128088345	Atp5b	+
7	80095174	80095179	Idh2	-
1	37898302	37898307	Mrpl30	+
12	111293281	111293286	Cdc42bpb	-
7	46855064	46855069	Ldha	+
14	120431440	120431445	Mbnl2	+
7	100485913	100485918	Ucp3	+
5	113777866	113777871	Iscu	+
10	53345189	53345194	Pln	+
7	105557772	105557777	Smpd1	+
4	46115240	46115245	Tmod1	+
18	34347059	34347064	Reep5	-
13	30541284	30541289	Uqcrfs1	-
6	32792524	32792529	Chchd3	-
16	37649015	37649020	Ndufb4	-
5	24409707	24409712	Abcb8	+
2	150831372	150831377	Pygb	+
3	96529011	96529016	Hfe2	+
4	130315372	130315377	Fabp3	+
19	21281468	21281473	Zfand5	+
6	115954970	115954975	Plxnd1	-
9	123118961	123118966	Exosc7	+
8	22588782	22588787	Vdac3	-
3	93564361	93564366	S100a10	+
4	130308830	130308835	Fabp3	+
2	14044783	14044788	Hacd1	-
2	130281667	130281672	Idh3b	-
11	31549379	31549384	Ncoa2	+
4	116074923	116074928	Uqcrh	-
8	121596889	121596894	Map1lc3b;Gr	+
7	99479979	99479984	Rps3	-
5	115345807	115345812	Cox6a1	-
19	60867594	60867599	Prdx3	-
5	76967009	76967014	Paics	+
8	121598000	121598005	Map1lc3b;Gr	+

15	98932321	98932326	Tuba1b;Gm4	-
14	75846309	75846314	Tpt1	+
9	120130251	120130256	Rpsa	+
11	6427486	6427491	H2afv	-
9	79769715	79769720	Tmem30a	-
6	87843434	87843439	Cnbp	-
11	84821178	84821183	Dhrs11	-
7	46855196	46855201	Ldha	+
11	97711140	97711145	Psmb3	+
17	56614214	56614219	Rpl36	+
5	92392941	92392946	Art3	+
7	111074725	111074730	Eif4g2	-
5	5782198	5782203	Gm15459	-
3	79604099	79604104	Etfdh	-
6	112472824	112472829	Cav3	+
9	53581800	53581805	Acat1	-
1	24613279	24613284	Gm28437	-
15	58939348	58939353	Ndufb9	+
11	59212156	59212161	Arf1	-
10	128084036	128084041	Atp5b	+
3	108014823	108014828	Gstm1	-
6	32803985	32803990	Chchd3	-
11	95831716	95831721	Phospho1;Zf	+
3	90512011	90512016	S100a1	-
7	36118655	36118660	Rpl17-ps9	+
11	73176040	73176045	Emc6	-
11	21564114	21564119	Mdh1	-
6	34310947	34310952	Akr1b3	-
11	17233757	17233762	Wdr92	+
18	77778816	77778821	Atp5a1	+
11	21557559	21557564	Mdh1	-
7	114705704	114705709	Gm15500	-
4	40279261	40279266	Ndufb6	-
10	91116819	91116824	Slc25a3	-
11	58932439	58932444	Rnf187	-
11	115415860	115415865	Atp5h	-
2	32633839	32633844	Ak1	+
11	53430775	53430780	Uqcrq	-
10	79888832	79888837	Gm19810	+
9	50753168	50753173	Cryab	+
19	6989494	6989499	Dnajc4	-
18	36682679	36682684	Slc35a4	+
3	88169349	88169354	Mef2d	+
14	8172900	8172905	Pdhb	-
12	79156447	79156452	Vti1b	-

3	106574653	106574658	Dram2	+
1	135851709	135851714	Tnnt2	+
17	66083405	66083410	Ndufv2	-
17	87435105	87435110	Calm2	-
9	54601159	54601164	Idh3a	+
7	120645234	120645239	Uqcrc2	+
19	36112695	36112700	Ankrd1	-
13	12270837	12270842	Actn2	-
10	128558932	128558937	Pa2g4	-
13	91859902	91859907	Ckmt2	-
5	104065096	104065101	Nudt9	+
5	137606903	137606908	Pcolce	-
11	98800862	98800867	Msl1	+
3	116494116	116494121	Rtca	-
14	73366501	73366506	Itm2b	-
14	34344578	34344583	Glud1	+
1	24612549	24612554	Gm10222	-
8	102865515	102865520	Gm8730	-
7	30555236	30555241	Hspb6	+
13	91853569	91853574	Ckmt2	-
10	97513679	97513684	Dcn	+
14	75846212	75846217	Tpt1	+
17	35832528	35832533	Flot1	+
4	82290991	82290996	Nfib	-
12	103321593	103321598	Asb2	-
10	57811409	57811414	Smpdl3a	+
19	4001842	4001847	Nudt8;Gm49	+
7	28832226	28832231	Ech1	+
8	4255630	4255635	Snapc2	+
5	122454288	122454293	Atp2a2	-
14	54941938	54941943	Myh6	-
13	12304315	12304320	Actn2	-
3	123013770	123013775	Myoz2	-
8	70534991	70534996	Fkbp8	+
2	181188456	181188461	Ppdpf	+
7	80095135	80095140	Idh2	-
17	33952517	33952522	Rps18	-
14	65981495	65981500	Clu	+
2	173778948	173778953	Vapb	+
1	55080292	55080297	Hspd1	-
11	80139963	80139968	Tefm	-
2	104114032	104114037	Cd59a	+
6	97233076	97233081	Arl6ip5	+
4	126055474	126055479	Mrps15	+
5	30176946	30176951	Hadhb	+

7	45338698	45338703	Hrc	+
10	117278158	117278163	Lyz2	-
12	111669582	111669587	Ckb	-
9	40803847	40803852	Hspa8	+
2	28933843	28933848	Gm13394;Cf	-
11	120488203	120488208	Mrpl12	+
9	106431487	106431492	Rpl29	+
8	94844844	94844849	Coq9	+
14	65980981	65980986	Clu	+
15	102326202	102326207	Pfdn5	+
4	108816807	108816812	Btf3l4	-
6	32792264	32792269	Chchd3	-
2	122151038	122151043	B2m	+
14	73365834	73365839	Itm2b	-
9	40804186	40804191	Hspa8	+
17	83502653	83502658	Cox7a2l	-
18	67404785	67404790	Afg3l2	-
2	181147702	181147707	Eef1a2	-
12	111961438	111961443	Atp5mpl	-
4	133965264	133965269	Hmgn2	-
8	23148507	23148512	Ank1	+
19	10899025	10899030	Prpf19	+
7	27305188	27305193	Ltbp4	-
15	55534279	55534284	Mrpl13	-
17	45568419	45568424	Hsp90ab1	-
3	142303221	142303226	Pdlim5	-
4	149453381	149453386	Rbp7	-
14	63199844	63199849	Gata4	-
8	22569524	22569529	Slc20a2	+
3	146838959	146838964	Gm10288	-
5	129881267	129881272	Chchd2;Phk(	-
12	85134044	85134049	Dlst	+
19	47085978	47085983	Usmg5	-
10	53344420	53344425	Pln	+
1	135301913	135301918	Timm17a	-
3	32935228	32935233	Usp13	+
12	112654252	112654257	Akt1	-
9	22013953	22013958	Prkcsh	+
8	13257672	13257677	Dcun1d2	-
4	118410988	118410993	Med8	+
3	52982275	52982280	Cog6	-
1	167309308	167309313	Tmco1	+
11	32283701	32283706	Hba-a1	+
10	29698937	29698942	Gm10275	-
2	26636307	26636312	Fam69b	+

2	29349887	29349892	Med27	+
5	114250454	114250459	Acacb	+
2	10063328	10063333	Atp5c1	-
7	111071192	111071197	Eif4g2	-
19	6982675	6982680	Vegfb	-
9	106215008	106215013	Twf2	+
10	128548218	128548223	Rpl41	-
18	60778496	60778501	Rps14	+
2	163467165	163467170	Fitm2	-
5	137759667	137759672	Tsc22d4	+
7	4794093	4794098	Rpl28	+
9	121849551	121849556	Higd1a	-
7	99479917	99479922	Rps3	-
10	60302273	60302278	Psap	+
1	171238817	171238822	Ndufs2	-
9	22185779	22185784	Rpl15-ps3	-
17	10203357	10203362	Qk	-
10	53345264	53345269	Pln	+
4	116066978	116066983	Uqcrh	-
8	22463127	22463132	Smim19	-
18	38317451	38317456	Rnf14	+
12	16536448	16536453	Lpin1	-
5	121208949	121208954	Rpl6	+
10	80056263	80056268	Gpx4	+
13	34977846	34977851	Eci2	-
8	34168474	34168479	Saraf	+
12	31331976	31331981	Dld	-
10	128362019	128362024	Cs	+
5	5782255	5782260	Gm15459	-
19	7215254	7215259	Cox8a	-
2	75191332	75191337	Rps6-ps4	-
19	6906747	6906752	Prdx5	-
7	51747800	51747805	Gm7336	+
7	43454528	43454533	Etfb;Gm4523	+
10	81181360	81181365	Eef2	+
8	84835316	84835321	Rad23a	-
1	151364191	151364196	Ivns1abp	+
7	34140895	34140900	Uba2	-
1	181903423	181903428	Enah	-
17	66080819	66080824	Ndufv2	-
5	5781573	5781578	Pcdh7	-
7	46855101	46855106	Ldha	+
9	110769130	110769135	Myl3	+
17	24896077	24896082	Mrps34	+
13	55597187	55597192	Tmed9	+

7	28830217	28830222	Ech1	+
2	35290356	35290361	Gsn	+
19	7197910	7197915	Macrod1	+
7	100485632	100485637	Ucp3	+
6	142494149	142494154	Ldhb	-
14	31000088	31000093	Spcs1	-
7	34202390	34202395	Gpi1	-
2	30174010	30174015	Endog	+
15	83153875	83153880	Cyb5r3	-
19	43499771	43499776	Got1	-
3	90511243	90511248	S100a1	-
5	115561445	115561450	Rplp0	+
2	36044447	36044452	Ndufa8	-
7	30555227	30555232	Hspb6	+
7	4520445	4520450	Tnni3	-
16	37647684	37647689	Ndufb4	-
8	111139491	111139496	Aars	+
13	45546195	45546200	Gmpr	+
14	73581704	73581709	Sucla2	+
11	40748844	40748849	Ccng1	-
5	137759474	137759479	Tsc22d4	+
17	24439054	24439059	Eci1	+
5	17785835	17785840	Cd36	-
7	102107232	102107237	Art1	+
7	93179626	93179631	Gm15501	-
3	10206014	10206019	Fabp4	-
17	35267088	35267093	H2-D1	+
2	4938518	4938523	Phyh	+
13	75645079	75645084	Mir682;Gm4	+
11	70978772	70978777	C1qbp	-
17	24896025	24896030	Mrps34	+
11	120488447	120488452	Mrpl12	+
8	72586797	72586802	Tmem38a	+
8	85539240	85539245	Dnaja2	-
13	66901454	66901459	Uqcrb	-
3	142302757	142302762	Pdlim5	-
13	30541015	30541020	Uqcrfs1	-
3	105954161	105954166	Atp5f1	-
2	153010275	153010280	Pdrg1	-
2	153009256	153009261	Pdrg1	-
2	130283723	130283728	Idh3b	-
7	19701691	19701696	Tomm40	-
2	90898493	90898498	Ndufs3	-
5	122461624	122461629	Atp2a2	-
7	45337306	45337311	Hrc	+

4	48673395	48673400	Cavin4	+
14	75846718	75846723	Tpt1	+
9	54603285	54603290	Idh3a	+
5	129794503	129794508	Cct6a	+
4	34771325	34771330	Smim8	-
19	24876179	24876184	Gm10053	+
9	40803386	40803391	Hspa8	+
11	31548353	31548358	Ncoa2	+
17	49993749	49993754	Rftn1	-
1	36531343	36531348	Ankrd23;Gm	-
6	124935936	124935941	Mlf2	+
15	76345685	76345690	Cyc1	+
7	43452396	43452401	Etfb;Gm4523	+
10	97507787	97507792	Dcn	+
11	54910006	54910011	Gpx3	+
4	116699452	116699457	Prdx1	+
3	8802277	8802282	Mrps28	-
1	66854654	66854659	Acadl	-
8	71369256	71369261	Use1	+
9	67031089	67031094	Tpm1	-
3	146838883	146838888	Gm10288	-
11	116849097	116849102	Mettl23	+
14	61221456	61221461	Sgcg	-
6	128430248	128430253	Fkbp4	-
9	54604260	54604265	Idh3a	+
4	106440963	106440968	Usp24	+
11	88210740	88210745	Mrps23	+
8	19493279	19493284	Rpl19-ps11	+
7	30185331	30185336	Cox7a1	+
4	91852114	91852119	Gm12671	-
11	40748951	40748956	Ccng1	-
16	65539466	65539471	Chmp2b	-
2	30403601	30403606	Crat	-
8	95864661	95864666	Got2	-
2	10059069	10059074	Atp5c1	-
15	75597120	75597125	Gpihbp1	+
8	23148305	23148310	Ank1	+
1	24615367	24615372	Gm28661	-
3	153930347	153930352	Acadm	-
7	28825970	28825975	Ech1	+
1	75216487	75216492	Tuba4a	-
19	56321768	56321773	Nrap	-
12	100206968	100206973	Calm1	+
12	113144407	113144412	Crip2	+
13	73319967	73319972	Ndufs6	-

7	24972219	24972224	Rabac1	-
5	143564057	143564062	Fam220a;Fa	+
6	108140283	108140288	Rpl36-ps12;S	-
6	11900559	11900564	Ndufa4	-
7	24888453	24888458	Rps19	+
11	120488154	120488159	Mrpl12	+
16	20692763	20692768	Eif4g1	+
15	58783460	58783465	Tmem65	-
15	76353915	76353920	Maf1	+
7	30187678	30187683	Capns1	-
15	75045591	75045596	Ly6c1	-
1	189277606	189277611	Pdcd5-ps;Kc	-
9	50522039	50522044	Pts	-
13	66901449	66901454	Uqcrb	-
15	98129751	98129756	Pfkm	+
15	80078010	80078015	Rpl3	-
9	71479380	71479385	Polr2m	-
1	172197317	172197322	Pea15a	-
10	128088973	128088978	Atp5b	+
2	148872848	148872853	Cst3	-
12	84774882	84774887	Isca2	+
16	20176561	20176566	Klhl24	-
3	94944552	94944557	Selenbp1	+
17	24439112	24439117	Eci1	+
13	91863246	91863251	Ckmt2	-
4	43663961	43663966	Hint2	+
6	28426369	28426374	Arf5	+
11	70661123	70661128	Eno3	+
17	45567950	45567955	Hsp90ab1	-
6	50564922	50564927	Cycs	-
19	6906723	6906728	Prdx5	-
7	73776858	73776863	Fam174b	+
17	34934929	34934934	Neu1	+
1	24614955	24614960	Gm28661	-
14	64123072	64123077	Msra	-
4	129143412	129143417	Fndc5	+
8	71367984	71367989	Use1	+
5	77087706	77087711	Hopx	-
10	78171265	78171270	Pwp2	-
9	64178225	64178230	Rpl4	+
13	11554141	11554146	Ryr2	-
15	73757159	73757164	Ptp4a3	+
5	129758185	129758190	Nipsnap2	+
9	50635830	50635835	Dlat	-
5	77095009	77095014	Hopx	-

6	133106001	133106006	Smim10l1	+
9	57531667	57531672	Cox5a	+
17	15494415	15494420	Psmb1	-
13	23739583	23739588	Hist1h1c	+
14	20694772	20694777	Sec24c	+
10	97518029	97518034	Dcn	+
16	36043928	36043933	Fam162a	-
11	70010411	70010416	Acadvl	-
2	4938359	4938364	Phyh	+
4	33246272	33246277	Pnrc1	-
3	19691758	19691763	Trim55	+
5	122454276	122454281	Atp2a2	-
5	115110836	115110841	Acads	-
9	116040770	116040775	Gm9385	-
5	115345762	115345767	Cox6a1	-
14	120944405	120944410	Ipo5	+
8	33866333	33866338	Rbpms	-
2	181149707	181149712	Eef1a2	-
16	36043915	36043920	Fam162a	-
11	20335458	20335463	Gm12033	-
7	4522371	4522376	Tnni3	-
9	122176340	122176345	Ano10	-
6	108665511	108665516	Bhlhe40	+
19	43503021	43503026	Got1	-
6	82725957	82725962	Hk2	-
13	114288658	114288663	Ndufs4	-
7	80099107	80099112	Idh2	-
7	19696471	19696476	Apoe	-
5	115103294	115103299	Rpl37rt	-
12	110898480	110898485	Rps19-ps6;T	+
10	78162480	78162485	D10Jhu81e	-
16	64766818	64766823	4930453N24	-
7	31051949	31051954	Fxyd1	-
7	80097839	80097844	Idh2	-
14	8170359	8170364	Pdhb	-
14	34576994	34576999	Ldb3	-
14	61221492	61221497	Sgcg	-
2	121452623	121452628	Serf2	+
1	128038803	128038808	Rpl28-ps1	+
10	53344195	53344200	Pln	+
13	91859940	91859945	Ckmt2	-
7	3706695	3706700	Rps9	+
6	39592694	39592699	Gm42420;Nc	+
10	45875936	45875941	Gpx4-ps2	+
17	56721382	56721387	Ndufa11	+

6	6558412	6558417	Sem1	-
6	136875750	136875755	Mgp	-
10	91117284	91117289	Slc25a3	-
8	46534715	46534720	Acsl1	+
3	37714916	37714921	Rps23-ps1	+
6	135023420	135023425	Ddx47	+
4	136144908	136144913	Id3	+
6	142498531	142498536	Ldhb	-
11	40748780	40748785	Ccng1	-
7	30185137	30185142	Cox7a1	+
2	90902931	90902936	Ndufs3	-
5	139338171	139338176	Cox19	-
18	61259564	61259569	Rps2-ps10	-
10	60302344	60302349	Psap	+
7	24862335	24862340	Gm9844	+
2	25466825	25466830	Ptgds	-
10	91116803	91116808	Slc25a3	-
5	122460081	122460086	Atp2a2	-
15	99404483	99404488	Tmbim6	+
11	32284339	32284344	Hba-a1	+
10	79711504	79711509	Bsg	+
12	65066729	65066734	Fkbp3	-
17	24720568	24720573	Rps2	+
3	79603884	79603889	Etfdh	-
11	69943370	69943375	Slc2a4	-
14	51905681	51905686	Ndrg2	-
18	77779211	77779216	Atp5a1	+
19	24876092	24876097	Gm10053	+
11	59204627	59204632	Mrpl55	+
5	30184556	30184561	Hadhb	+
11	106781999	106782004	Ddx5	-
2	73909875	73909880	Atp5g3	-
17	33952290	33952295	Rps18	-
7	143067569	143067574	Cd81	+
9	40805150	40805155	Hspa8	+
3	103057597	103057602	Csde1	+
16	4482684	4482689	Srl	-
2	30402364	30402369	Crat	-
4	57191251	57191256	Ptpn3	-
3	36452220	36452225	Anxa5	-
9	59679055	59679060	Pkm	+
3	24333201	24333206	Gm7536	+
10	79710383	79710388	Bsg	+
7	140105929	140105934	Echs1	-
9	79769455	79769460	Tmem30a	-

7	48830533	48830538	Csrp3	-
9	50635962	50635967	Dlat	-
11	20334894	20334899	Steap3	-
4	116074961	116074966	Uqcrh	-
12	112498197	112498202	A530016L24I	+
11	55049921	55049926	Ccdc69	-
5	148315108	148315113	Mtus2	+
5	117283333	117283338	Pebp1	-
17	24721824	24721829	Rps2	+
15	99727689	99727694	Cox14	+
11	70662024	70662029	Eno3	+
15	81950731	81950736	Csdc2	+
11	69916880	69916885	Eif5a	-
7	46851988	46851993	Ldha	+
13	91853498	91853503	Ckmt2	-
9	108946942	108946947	Uqcrc1	+
8	33782805	33782810	Rbpms	-
9	120572835	120572840	Rpl14	+
2	153009231	153009236	Pdrg1	-
5	30178699	30178704	Hadhb	+
3	153936314	153936319	Acadm	-
11	70661487	70661492	Eno3	+
2	25224008	25224013	Tubb4b	-
6	35260453	35260458	1810058I24F	+
3	90235644	90235649	Jtb	+
11	88211155	88211160	Mrps23	+
12	110858662	110858667	Mpc1-ps	-
4	138313787	138313792	Pink1	-
10	127022654	127022659	Tsfm	-
10	128086104	128086109	Atp5b	+
1	151364153	151364158	Ivns1abp	+
16	4480345	4480350	Srl	-
16	4480735	4480740	Srl	-
2	130279428	130279433	Idh3b	-
3	142302762	142302767	Pdlim5	-
1	120067768	120067773	Tmem37	-
11	115607177	115607182	Mrps7	+
8	70700133	70700138	Jund	+
14	88123514	88123519	Rps3a2	-
6	17340459	17340464	Cav1	+
16	14299689	14299694	Fopnl	-
9	72749189	72749194	Nedd4	+
7	80095676	80095681	Idh2	-
7	102239189	102239194	Rhog	-
12	31331829	31331834	Dld	-

17	66079445	66079450	Ndufv2	-
13	14625249	14625254	Psma2	+
7	140900246	140900251	Cox8b	-
3	146839206	146839211	Gm10288	-
8	95713907	95713912	Ndrg4	+
5	108433325	108433330	Atp5k	-
9	78478630	78478635	Gm26377;Ee	-
1	171235054	171235059	Ndufs2	-
5	34631462	34631467	Add1	+
11	6349894	6349899	Ogdh	+
14	55576920	55576925	Fitm1	+
5	116408652	116408657	Hspb8	-
1	55091230	55091235	Hspe1	+
15	98951611	98951616	Tuba1a;Gm4	-
1	36423583	36423588	Lman2l	-
10	77616387	77616392	Sumo3;Gm4	+
10	91116947	91116952	Slc25a3	-
14	75845442	75845447	Tpt1	+
15	102523505	102523510	Tarbp2	+
18	61259184	61259189	Rps2-ps10	-
8	124914047	124914052	Egln1	-
2	38640178	38640183	Psmb7	-
1	180167145	180167150	Coq8a	-
11	116850909	116850914	Srsf2	-
10	81182126	81182131	Eef2	+
19	6398057	6398062	Pygm	+
5	41625688	41625693	Rab28	-
14	75848161	75848166	Tpt1	+
4	140972952	140972957	Sdhb	+
8	83566898	83566903	Ndufb7	+
10	88743755	88743760	Arl1	+
1	75215791	75215796	Tuba4a	-
16	84828474	84828479	Atp5j	-
9	78478510	78478515	Eef1a1	-
10	128362346	128362351	Cs	+
3	90061962	90061967	4933434E20	+
10	60293452	60293457	Psap	+
8	124910544	124910549	Egln1	-
1	181242119	181242124	Rpl35a-ps2	-
10	81182069	81182074	Eef2	+
17	46122770	46122775	Mrps18a	+
7	30555174	30555179	Hspb6	+
15	35932057	35932062	Cox6c	-
11	101278762	101278767	Coa3	-
14	34561519	34561524	Ldb3	-

18	60809058	60809063	Cd74	+
8	70868800	70868805	Ccdc124	-
9	57528994	57528999	Cox5a	+
2	181150960	181150965	Eef1a2	-
17	34284434	34284439	H2-Aa	-
2	84958372	84958377	Slc43a3	+
2	148872882	148872887	Cst3	-
13	98310086	98310091	Btf3	-
9	120128466	120128471	Rpsa	+
19	11772945	11772950	Mrpl16	+
1	64994174	64994179	Rpl10a-ps1	-
11	52388639	52388644	Vdac1	+
4	136051231	136051236	Rpl11	-
4	129849902	129849907	Ptp4a2	+
5	45520444	45520449	Med28	+
7	30185993	30185998	Cox7a1	+
10	80318767	80318772	2310011J03I	-
1	4776678	4776683	Mrpl15	-
7	25228922	25228927	Gsk3a	-
6	135382181	135382186	Emp1	+
5	115242497	115242502	Rnf10	-
6	124934704	124934709	Mlf2	+
9	50653637	50653642	Dlat	-
1	63147396	63147401	Ndufs1	-
7	29179159	29179164	Psmd8	-
4	6395126	6395131	Sdcbp	+
1	55089123	55089128	Hspe1	+
10	78162602	78162607	D10Jhu81e	-
11	5525650	5525655	Xbp1	+
1	151363617	151363622	Ivns1abp	+
4	140977486	140977491	Sdhb	+
8	111994961	111994966	Kars	-
14	73362946	73362951	Itm2b	-
16	18301377	18301382	Tango2	-
10	80406753	80406758	Uqcr11	-
11	109669074	109669079	Prkar1a	+
4	46115832	46115837	Tmod1	+
8	83572749	83572754	Tecr	-
1	133610223	133610228	Snrpe	-
7	141458880	141458885	Pnpla2	+
4	15986588	15986593	Nbn	+
14	63143076	63143081	Ctsb	+
4	47312704	47312709	Col15a1	+
13	91863305	91863310	Ckmt2	-
16	36049937	36049942	Fam162a	-

4	116692885	116692890	Prdx1	+
7	142376452	142376457	Gm49369;Ct	-
8	56591849	56591854	Fbxo8	+
13	23692279	23692284	Hist1h2bc	+
7	139558953	139558958	Inpp5a	+
15	85438133	85438138	Atxn10	+
8	11199629	11199634	Col4a1	-
1	171129895	171129900	Sdhc	-
11	59185037	59185042	Guk1	-
13	38198377	38198382	Dsp	+
16	92310806	92310811	Smim11	+
6	115805644	115805649	Rpl32	-
3	102146414	102146419	Casq2	+
2	19394510	19394515	Msrb2	+
10	56969019	56969024	Gja1	-
11	21557364	21557369	Mdh1	-
14	31210153	31210158	Tnnc1	+
12	113144464	113144469	Crip2	+
11	20334979	20334984	Steap3	-
6	142492551	142492556	Ldhb	-
13	86045771	86045776	Cox7c	-
3	90511291	90511296	S100a1	-
1	133603983	133603988	Snrpe	-
15	81913125	81913130	Aco2	+
12	110858636	110858641	Mpc1-ps	-
15	43511765	43511770	Emc2	+
11	55394571	55394576	Sparc	-
5	116014173	116014178	Prkab1	-
10	80341409	80341414	Adamtsl5	-
3	146838903	146838908	Gm10288	-
15	55540155	55540160	Mrpl13	-
5	129757508	129757513	Nipsnap2	+
15	89417871	89417876	Gm44502;Cp	-
2	13582656	13582661	Vim	+
2	90911159	90911164	Ptpmt1	-
3	142304303	142304308	Pdlim5	-
19	6392960	6392965	Pygm	+
5	118056559	118056564	Tesc	+
13	91859271	91859276	Ckmt2	-
9	122947875	122947880	1110059G10	-
9	54603352	54603357	Idh3a	+
12	31927289	31927294	Hbp1	-
12	75631251	75631256	Wdr89	-
14	61232406	61232411	Sgcg	-
6	142565758	142565763	Kcnj8	-

5	95862540	95862545	Gm5559	+
3	90614338	90614343	S100a6	+
5	17785877	17785882	Cd36	-
7	28829901	28829906	Ech1	+
7	45719275	45719280	Rpl18	+
17	13015095	13015100	Sod2	+
3	153922547	153922552	Acadm	-
12	55398941	55398946	Psma6	+
15	73809571	73809576	Ndufb4c;Mro	-
10	59371836	59371841	P4ha1	+
4	136896100	136896105	C1qa	-
11	55395002	55395007	Sparc	-
16	14190171	14190176	Nde1	+
12	110858245	110858250	Wdr20	-
10	95324267	95324272	Gm48882	+
2	172509478	172509483	Rtf2	-
14	54617903	54617908	Psmb5	-
5	115345771	115345776	Cox6a1	-
11	70644613	70644618	Slc25a11	-
4	150248560	150248565	Eno1	+
9	67027935	67027940	Tpm1	-
8	11198673	11198678	Col4a1	-
12	100121073	100121078	Psmc1	+
11	115415794	115415799	Atp5h	-
19	47864600	47864605	Gsto1	+
2	39004141	39004146	Rpl35	-
13	24820718	24820723	Acot13	-
17	14404249	14404254	Smoc2	+
9	121712673	121712678	Ss18l2	+
3	54390629	54390634	Postn	+
12	44311559	44311564	Pnpla8	+
15	79761618	79761623	Dnal4	-
3	97689887	97689892	Pde4dip	-
2	28933669	28933674	Gm13394;Cf	-
18	34938154	34938159	Hspa9	-
1	165480972	165480977	Mpc2	+
8	83723138	83723143	Ddx39	+
14	31000128	31000133	Spcs1	-
17	56259473	56259478	Fem1a	+
4	126709555	126709560	Psmb2	+
5	95862618	95862623	Gm5559	+
7	45461715	45461720	Bax	-
2	132529194	132529199	Gpcpd1	-
11	52383918	52383923	Vdac1	+
15	77017709	77017714	Mb	-

11	6596368	6596373	Ccm2	+
15	76905880	76905885	Rpl8	+
11	5715885	5715890	Urgcp	-
3	123034193	123034198	Myoz2	-
2	30405391	30405396	Crat	-
6	39599420	39599425	Ndufb2	+
7	128546834	128546839	Bag3	+
7	30185964	30185969	Cox7a1	+
16	38373934	38373939	Popdc2	+
13	73618406	73618411	Clptm1l	+
11	30109031	30109036	Sptbn1	-
15	73757195	73757200	Ptp4a3	+
1	86543409	86543414	Pde6d	-
3	79618896	79618901	Etfdh	-
5	125386118	125386123	Ubc	-
13	111593332	111593337	Gpbp1	-
5	114420985	114420990	Ube3b	+
3	129888909	129888914	Pla2g12a	+
4	126232738	126232743	Map7d1	-
12	111669522	111669527	Ckb	-
7	45719953	45719958	Rpl18	+
14	66084966	66084971	Ephx2	-
3	154786521	154786526	Tnni3k	-
11	20063089	20063094	Actr2	-
10	128048521	128048526	Naca	+
8	11445028	11445033	Col4a2	+
17	35253613	35253618	Ddx39b	+
6	17339743	17339748	Cav1	+
19	8880249	8880254	Uqcc3	-
8	102865269	102865274	Gm8730	-
17	25830734	25830739	Stub1	-
4	57943882	57943887	Txn1	-
12	85343488	85343493	Tmed10	-
7	46850930	46850935	Ldha	+
19	6908072	6908077	Prdx5	-
6	125361912	125361917	Tnfrsf1a	+
8	23148392	23148397	Ank1	+
19	57034240	57034245	Ablim1	-
8	122424892	122424897	Cyba	-
9	55454636	55454641	Etfa	-
11	115418490	115418495	Atp5h	-
11	54908631	54908636	Gpx3	+
8	84971612	84971617	Prdx2	+
1	43123084	43123089	Fhl2	-
6	50565102	50565107	Cycs	-

7	25624844	25624849	Dmac2	+
7	80100817	80100822	Idh2	-
9	123790130	123790135	AC165425.1;	-
1	66831452	66831457	Acadl	-
1	170925382	170925387	Gm2962;Fcr	-
15	35931979	35931984	Cox6c	-
9	123789539	123789544	AC165425.1;	-
6	83802513	83802518	Nagk	+
17	8297613	8297618	Mpc1	+
9	59679279	59679284	Pkm	+
17	33997040	33997045	H2-K1	-
15	74959001	74959006	Ly6e	+
3	58592962	58592967	Selenot	+
10	62269756	62269761	Hk1	-
3	20122256	20122261	Gyg	-
11	40749118	40749123	Ccng1	-
3	142304377	142304382	Pdlim5	-
2	114051991	114051996	Actc1	-
15	81911910	81911915	Aco2	+
9	119485450	119485455	Scn5a	-
5	5781934	5781939	Gm15459	-
16	90225167	90225172	Sod1	+
1	165478235	165478240	Mpc2	+
16	38377952	38377957	Popdc2	+
1	51302471	51302476	Cavin2	+
9	79758552	79758557	Cox7a2	-
8	104631232	104631237	Rrad	-
16	36044054	36044059	Fam162a	-
7	45458539	45458544	Ftl1	-
1	161244315	161244320	Prdx6	-
1	52920658	52920663	Hibch	+
10	117278017	117278022	Lyz2	-
7	138882694	138882699	Ppp2r2d	+
6	126827583	126827588	Ndufa9	-
2	153014051	153014056	Pdrg1	-
9	65689218	65689223	Oaz2	+
5	77349265	77349270	Igfbp7	-
17	66079538	66079543	Ndufv2	-
19	57033479	57033484	Ablim1	-
10	128085468	128085473	Atp5b	+
8	33782877	33782882	Rbpms	-
11	96047329	96047334	Snf8	+
14	75848177	75848182	Tpt1	+
8	123891802	123891807	Acta1	-
17	8128831	8128836	Gm49673;Rr	-

11	70662380	70662385	Eno3	+
9	50597159	50597164	Sdhd	-
8	94853245	94853250	Coq9	+
11	51997884	51997889	Ube2b;Gm26	-
7	43457619	43457624	Etfb;Gm4523	+
6	72369175	72369180	Vamp5	-
6	113515099	113515104	Emc3	-
12	113153824	113153829	Crip1	+
4	136145127	136145132	Id3	+
17	56011888	56011893	Mpnd	+
19	32465932	32465937	Rpl9-ps6	-
5	90229018	90229023	Ankrd17	-
10	128211300	128211305	Spryd4	-
9	89699835	89699840	Tmed3	-
5	124493515	124493520	Rilpl1	-
18	36742527	36742532	Ndufa2	-
17	24169400	24169405	Atp6v0c	-
19	6386033	6386038	Pygm	+
1	58591118	58591123	Ndufb3	+
3	79605042	79605047	Etfdh	-
8	85539129	85539134	Dnaja2	-
10	71255608	71255613	Ube2d1	-
9	50636202	50636207	Dlat	-
10	111496351	111496356	Nap1l1	+
4	149744538	149744543	Slc25a33	-
11	115607060	115607065	Mrps7	+
5	122460793	122460798	Atp2a2	-
17	24639370	24639375	Slc9a3r2	-
14	73595615	73595620	Sucla2	+
11	48800546	48800551	Rack1	+
14	8171524	8171529	Pdhb	-
2	131177596	131177601	Cenpb;Spef1	-
15	98131676	98131681	Pfkm	+
9	108337146	108337151	Rhoa	+
5	30184571	30184576	Hadhb	+
11	100361675	100361680	Eif1	-
4	148000802	148000807	Nppa	+
18	25133096	25133101	Fhod3	+
5	24817759	24817764	Rheb	-
15	51787965	51787970	Eif3h	-
13	74327273	74327278	Sdha	-
6	119923999	119924004	Wnk1	-
12	110691182	110691187	Hsp90aa1	-
12	78843420	78843425	Atp6v1d	-
9	50344898	50344903	Rpl10-ps3	-

14	54943221	54943226	Myh6	-
5	24803580	24803585	Rheb	-
8	69894384	69894389	Ndufa13;Yjef	-
9	79759668	79759673	Cox7a2	-
5	125387969	125387974	Ubc	-
10	80826821	80826826	Oaz1	+
3	135458863	135458868	Ube2d3	+
17	13015344	13015349	Sod2	+
4	9621542	9621547	Asph	-
6	99878217	99878222	Tpt1-ps3	-
19	44552834	44552839	Ndufb8	-
2	148871930	148871935	Cst3	-
7	24885500	24885505	Rps19	+
10	80403014	80403019	Uqcr11	-
2	173034650	173034655	Rbm38	+
11	115416869	115416874	Atp5h	-
1	165474959	165474964	Mpc2	+
2	35307511	35307516	Gsn	+
7	125467684	125467689	Nsmce1	-
11	6355997	6356002	Ogdh	+
17	56608640	56608645	2410015M20	-
3	96196877	96196882	Bola1	-
1	75312011	75312016	Dnpep	-
7	19411117	19411122	Ckm	+
1	172272960	172272965	Atp1a2	-
7	3704653	3704658	Rps9	+
10	56390004	56390009	Gja1	+
1	118608854	118608859	Clasp1	+
6	115807818	115807823	Rpl32	-
11	84825495	84825500	Dhrs11	-
3	145879200	145879205	Rpl36a-ps2;[	+
7	4519463	4519468	Tnni3	-
11	70644419	70644424	Slc25a11	-
17	45569615	45569620	Hsp90ab1	-
9	50596509	50596514	Sdhd	-
13	66902731	66902736	Uqcrb	-
5	115298646	115298651	Dynll1	-
1	93509219	93509224	Sept2;Sept2	+
7	101911527	101911532	Lamtor1	+
2	114050402	114050407	Actc1	-
7	88530791	88530796	Rps13-ps2	-
14	76507175	76507180	Tsc22d1	+
17	83502680	83502685	Cox7a2l	-
8	46535880	46535885	Acsl1	+
4	116070012	116070017	Uqcrh	-

11	62552176	62552181	Ubb	+
14	77840207	77840212	Dnajc15	-
11	78181470	78181475	Rpl23a	-
11	20335247	20335252	Gm12033	-
4	59617892	59617897	Hsdl2	+
2	180257817	180257822	Rps21	+
9	21507770	21507775	Tmed1	-
13	75644938	75644943	Gm4149	+
11	54909784	54909789	Gpx3	+
18	38262428	38262433	Dele1	+
1	161241474	161241479	Prdx6	-
17	12924469	12924474	Tcp1	+
9	122176203	122176208	Ano10	-
5	122467735	122467740	Atp2a2	-
4	103366225	103366230	Oma1	+
7	132557947	132557952	Oat;Fgfr2	-
7	111073869	111073874	Eif4g2	-
2	74591442	74591447	Mrpl23-ps1	-
7	45493823	45493828	Nucb1	-
5	21757081	21757086	Pmpcb	+
18	34937899	34937904	Hspa9	-
13	66901484	66901489	Uqcrb	-
1	135309153	135309158	Timm17a	-
15	27594117	27594122	Ank	+
4	3973442	3973447	Gm11808	-
11	31548602	31548607	Ncoa2	+
3	97690341	97690346	Pde4dip	-
19	32465893	32465898	Rpl9-ps6	-
19	47864544	47864549	Gsto1	+
15	98127743	98127748	Pfkm	+
7	141191042	141191047	Hras	-
9	67047813	67047818	Tpm1	-
19	9984559	9984564	Fth1	+
10	81179629	81179634	Eef2	+
5	129128287	129128292	Rps16-ps2	-
5	117283425	117283430	Pebp1	-
14	34561462	34561467	Ldb3	-
10	17845506	17845511	Txlnb	+
5	129882535	129882540	Chchd2;Phk[illegible]	-
9	51957178	51957183	Fdx1	-
12	113145464	113145469	Crip2	+
5	143563651	143563656	Fam220a;Fa	+
5	3641446	3641451	Gatad1	-
15	4154083	4154088	Oxct1	+
9	108683925	108683930	Slc25a20	+

16	84954839	84954844	App	-
7	19415003	19415008	Ckm	+
1	51302318	51302323	Cavin2	+
17	29301427	29301432	BC004004	+
8	69901607	69901612	Ndufa13;Yjef	-
13	114316984	114316989	Ndufs4	-
2	76704744	76704749	Ttn	-
10	80406696	80406701	Uqcr11	-
10	81566067	81566072	Aes	+
11	120104551	120104556	Slc38a10	-
17	24721649	24721654	Rps2	+
2	108950230	108950235	Gm13910	+
6	99878011	99878016	Tpt1-ps3	-
11	70010865	70010870	Acadvl	-
2	174345499	174345504	Gnas	+
3	86139143	86139148	Rps3a1	-
18	38464705	38464710	Ndfip1	+
5	103990026	103990031	Hsd17b11	-
10	43440272	43440277	Pdss2	+
11	55499944	55499949	G3bp1	+
11	70010291	70010296	Acadvl	-
9	122187538	122187543	Ano10	-
10	84608470	84608475	Tcp11l2	+
9	121784097	121784102	Hhatl	-
16	20692461	20692466	Eif4g1	+
10	128047773	128047778	Naca	+
9	108947655	108947660	Uqcrc1	+
2	174341682	174341687	Gnas	+
7	30617017	30617022	Cox6b1	-
3	116924212	116924217	Palmd	-
5	125386568	125386573	Ubc	-
19	5099946	5099951	Rab1b	-
4	103564727	103564732	Gm12715	+
15	81910495	81910500	Aco2	+
9	107255303	107255308	Mapkapk3	-
9	56938298	56938303	Imp3	+
15	81910463	81910468	Aco2	+
2	25222579	25222584	Tubb4b	-
2	36189379	36189384	Mrrf	+
2	18675987	18675992	Commd3	+
17	70996856	70996861	Myl12a;Myl1:	-
14	55662521	55662526	Nedd8	-
6	71214330	71214335	Smyd1	-
6	137751009	137751014	Strap	+
17	23675135	23675140	Hcfc1r1	+

10	41488985	41488990	Smpd2	-
1	55078428	55078433	Hspd1	-
8	94854436	94854441	Coq9	+
16	84827885	84827890	Atp5j	-
10	53345116	53345121	Pln	+
14	37121589	37121594	Ghitm	-
9	72749683	72749688	Nedd4	+
17	33996841	33996846	H2-K1	-
5	125454698	125454703	Bri3bp	+
15	102281002	102281007	Mfsd5	+
6	52742820	52742825	Tax1bp1	+
2	121458267	121458272	Serf2;Hypk	+
17	56259702	56259707	Fem1a	+
11	95680621	95680626	Phb	+
2	74870456	74870461	Mtx2	+
2	25222823	25222828	Tubb4b	-
3	32577365	32577370	Mfn1	+
10	81565726	81565731	Aes	+
7	144579960	144579965	Fadd	-
10	80055934	80055939	Gpx4	+
12	8285126	8285131	Ldah	+
17	71126104	71126109	Myom1;Gm2	+
17	45568236	45568241	Hsp90ab1	-
1	63143819	63143824	Ndufs1	-
3	132660553	132660558	Aimp1	-
8	22580483	22580488	Vdac3	-
18	34349656	34349661	Reep5	-
6	133106476	133106481	Smim10l1	+
2	10059651	10059656	Atp5c1	-
17	8297520	8297525	Mpc1	+
15	27594075	27594080	Ank	+
11	49678815	49678820	Gm12191;Cr	-
11	54983313	54983318	Anxa6	-
8	95864917	95864922	Got2	-
3	53692418	53692423	Gm6204	-
11	54909507	54909512	Gpx3	+
8	85324972	85324977	Mylk3	-
5	30180381	30180386	Hadhb	+
11	96075016	96075021	Atp5g1	-
15	4155265	4155270	Oxct1	+
1	63143636	63143641	Ndufs1	-
1	95498952	95498957	Gm15427	+
6	55348159	55348164	Aqp1	+
10	80828997	80829002	Oaz1	+
15	43511779	43511784	Emc2	+

2	174463967	174463972	Atp5e	-
11	59013354	59013359	Obscn	-
3	53692560	53692565	Gm6204	-
2	130281871	130281876	Idh3b	-
11	96818707	96818712	Nfe2l1	-
13	86045818	86045823	Cox7c	-
5	36966220	36966225	Wfs1	-
17	29137310	29137315	Rpl35a-ps3	-
3	145879035	145879040	Rpl36a-ps2;[	+
13	38198436	38198441	Dsp	+
10	81566091	81566096	Aes	+
2	104999811	104999816	Eif3m	-
4	109452665	109452670	Rnf11	-
2	112367489	112367494	Emc4	-
1	165770756	165770761	Creg1	+
4	40936521	40936526	Bag1	-
16	38362615	38362620	Popdc2	+
10	57516210	57516215	Serinc1	-
7	45123481	45123486	Rps11	-
10	61692997	61693002	Sar1a	+
9	22092810	22092815	Gm6581	+
17	56017097	56017102	Sh3gl1	-
19	27260907	27260912	AC119982.1	-
1	165480905	165480910	Mpc2	+
10	62621450	62621455	Ddx50	-
16	90226301	90226306	Sod1	+
7	132558158	132558163	Oat;Fgfr2	-
17	27133814	27133819	Uqcc2	-
16	38363086	38363091	Popdc2	+
8	68906064	68906069	Lpl	+
8	85324385	85324390	Mylk3	-
1	24615228	24615233	Gm28661	-
10	79711778	79711783	Bsg	+
7	31116727	31116732	Scn1b	-
13	74360179	74360184	Lrrc14b	-
6	29461452	29461457	Flnc	+
9	108944717	108944722	Uqcrc1	+
5	129881305	129881310	Chchd2;Phk(	-
10	80254919	80254924	Ndufs7	+
5	5782573	5782578	Gm15459	-
5	29766819	29766824	Dnajb6	+
11	58995529	58995534	Obscn	-
1	59639498	59639503	Sumo1	-
8	46535313	46535318	Acsl1	+
19	9984573	9984578	Fth1	+

2	32681751	32681756	Eng	+
9	106431470	106431475	Rpl29	+
9	110769106	110769111	Myl3	+
4	126706125	126706130	Psmb2	+
17	30209370	30209375	Zfand3	+
18	61259744	61259749	Rps2-ps10	-
10	53345466	53345471	Pln	+
19	6392714	6392719	Pygm	+
2	73908702	73908707	Atp5g3	-
3	79605785	79605790	Etfdh	-
1	73910869	73910874	Tns1	-
13	49624296	49624301	Ogn	+
9	121455826	121455831	Trak1	+
6	71881034	71881039	Ptcd3	-
11	50202442	50202447	Sqstm1	-
12	84314871	84314876	Ptgr2	+
8	34170201	34170206	Saraf	+
2	36036454	36036459	Ndufa8	-
7	126490498	126490503	Tufm	+
7	88530648	88530653	Rps13-ps2	-
5	5781852	5781857	Gm15459	-
15	98123190	98123195	Pfkm	+
5	123578721	123578726	Clip1	-
12	8498402	8498407	Rhob	-
10	128361530	128361535	Cs	+
9	78480610	78480615	Eef1a1	-
9	59679042	59679047	Pkm	+
6	24605522	24605527	Lmod2	+
4	57370833	57370838	Ptpn3	-
2	26592572	26592577	Egfl7;Gm205	+
1	55091234	55091239	Hspe1	+
5	122106254	122106259	Myl2	+
11	69686855	69686860	Tnfsfm13;Tn	-
7	15917484	15917489	Selenow	-
12	102394511	102394516	Lgmn	-
13	41022422	41022427	Tmem14c	+
5	122550685	122550690	Ift81	-
2	25285278	25285283	Anapc2	+
13	75645147	75645152	Gm4149	+
17	24436206	24436211	Eci1	+
17	66087432	66087437	Ndufv2	-
8	15132872	15132877	Myom2	+
13	12280469	12280474	Actn2	-
4	139291374	139291379	Capzb	+
12	103321535	103321540	Asb2	-

4	141425077	141425082	Hspb7	+
7	118109155	118109160	Rps15a	-
5	95862742	95862747	Gm5559	+
15	98519150	98519155	Kansl2	-
3	131242584	131242589	Hadh	-
9	50344391	50344396	Rpl10-ps3	-
1	171238315	171238320	Ndufs2	-
4	46115623	46115628	Tmod1	+
14	20694447	20694452	Sec24c	+
11	59185297	59185302	Guk1	-
7	138894658	138894663	Bnip3	-
13	91853444	91853449	Ckmt2	-
14	21837859	21837864	Vdac2	+
19	5427179	5427184	AI837181	+
17	46649987	46649992	Mrpl2;Gm26	+
18	76930211	76930216	Ier3ip1;Hdhd	+
11	94291260	94291265	Luc7l3	-
7	45122538	45122543	Rps11	-
8	111622597	111622602	Znrf1	+
12	111670238	111670243	Ckb	-
5	30119840	30119845	Hadha	-
10	80392637	80392642	Mbd3	-
7	100485873	100485878	Ucp3	+
3	102145796	102145801	Casq2	+
7	46851925	46851930	Ldha	+
7	140898955	140898960	Cox8b	-
11	5803175	5803180	Pgam2	-
2	148875232	148875237	Cst3	-
1	66830993	66830998	Acadl	-
4	99986861	99986866	Pgm1	+
4	91851654	91851659	Gm12669	-
1	165481105	165481110	Mpc2	+
8	123892278	123892283	Acta1	-
4	59618159	59618164	Hsdl2	+
13	91865264	91865269	Ckmt2	-
5	136982651	136982656	Znhit1	-
2	120506987	120506992	Zfp106	-
8	68906343	68906348	Lpl	+
7	103813543	103813548	Hbb-bt	-
2	132750645	132750650	Shld1	+
12	72794255	72794260	Ppm1a	+
7	4518429	4518434	Tnni3	-
4	136550654	136550659	Kdm1a	-
1	24613131	24613136	Gm28438	-
11	20336015	20336020	Gm12033	-

8	64594966	64594971	Cpe	-
2	38588140	38588145	Psmb7	-
11	6616591	6616596	Tbrg4	-
2	50291321	50291326	Mmadhc	-
6	85137068	85137073	Spr	-
12	72101014	72101019	Jkamp	+
4	46115311	46115316	Tmod1	+
5	77094972	77094977	Hopx	-
12	85133751	85133756	Dlst	+
1	16647960	16647965	Eloc	-
2	90894707	90894712	Ndufs3	-
10	78162227	78162232	D10Jhu81e	-
10	97507798	97507803	Dcn	+
9	66065603	66065608	Ppib	+
6	125129931	125129936	Chd4	+
13	99415373	99415378	Mrps27	+
14	21838567	21838572	Vdac2	+
7	28826217	28826222	Ech1	+
4	59617905	59617910	Hsdl2	+
2	130281309	130281314	Idh3b	-
7	19414319	19414324	Ckm	+
3	60629686	60629691	Mbnl1	+
3	102146222	102146227	Casq2	+
12	54860960	54860965	Cfl2	-
14	65981398	65981403	Clu	+
14	51905514	51905519	Ndrg2	-
7	28832011	28832016	Ech1	+
17	46928611	46928616	Ubr2	-
5	122454013	122454018	Atp2a2	-
9	22076416	22076421	Ecsit	-
3	123026184	123026189	Myoz2	-
11	70238271	70238276	Gm21988;Rr	-
13	11553553	11553558	Ryr2	-
3	142302787	142302792	Pdlim5	-
4	46115009	46115014	Tmod1	+
13	36205011	36205016	Fars2	+
1	16102517	16102522	Rpl7	-
17	13017654	13017659	Sod2	+
14	34579278	34579283	Ldb3	-
11	76986487	76986492	Blmh	+
7	141402031	141402036	Taldo1	+
9	58652442	58652447	Nptn	+
7	81534715	81534720	Fsd2	-
10	81180230	81180235	Eef2	+
15	6654038	6654043	Gm7666;Fyb	+

7	19414172	19414177	Ckm	+
3	142302498	142302503	Pdlim5	-
7	35547865	35547870	Nudt19	-
11	93949971	93949976	Nme2;Gm20	-
9	7752094	7752099	Gm10709	+
17	56112141	56112146	Plin5	-
4	137570006	137570011	Hspg2	+
12	103321317	103321322	Asb2	-
1	75366759	75366764	Des	+
5	35652269	35652274	Htra3	-
6	142494130	142494135	Ldhb	-
7	80098157	80098162	Idh2	-
8	34170678	34170683	Saraf	+
13	24818036	24818041	Acot13	-
8	68906531	68906536	Lpl	+
14	29973369	29973374	Selenok	+
2	32402470	32402475	Ptges2	+
5	115561400	115561405	Rplp0	+
11	88211362	88211367	Mrps23	+
11	100887061	100887066	Stat3	-
1	75216370	75216375	Tuba4a	-
5	122489267	122489272	Atp2a2	-
9	55461681	55461686	Etfa	-
3	146503276	146503281	Gng5	+
3	152237500	152237505	Nexn	-
11	70646134	70646139	Slc25a11	-
16	18301205	18301210	Tango2	-
19	4004555	4004560	Gm49405;Dc	+
2	152185566	152185571	Rps15a-ps7	-
3	79603927	79603932	Etfdh	-
7	12923010	12923015	Rps5	+
7	30728443	30728448	Tmem147	-
5	135786308	135786313	Mdh2	+
17	28748210	28748215	Mapk14	+
6	83487027	83487032	Dguok	-
15	89055026	89055031	Mov10l1	+
9	107583793	107583798	Naa80;Hyal3	+
2	181153073	181153078	Eef1a2	-
13	24818121	24818126	Acot13	-
19	24876099	24876104	Gm10053	+
14	73363116	73363121	Itm2b	-
8	128722870	128722875	Itgb1	+
4	15932957	15932962	Decr1	-
8	124889365	124889370	Gnpat	+
8	11198998	11199003	Col4a1	-

15	74995318	74995323	Ly6a	-
12	65073849	65073854	Fkbp3	-
14	24492039	24492044	Rps24	+
11	120347680	120347685	Actg1	-
5	129884012	129884017	Chchd2;Phk(	-
17	29328812	29328817	Pi16	+
1	151363821	151363826	Ivns1abp	+
11	70644797	70644802	Slc25a11	-
2	173984562	173984567	Atp5k-ps2	-
7	28832028	28832033	Ech1	+
18	60776453	60776458	Rps14	+
7	25630039	25630044	Bckdha	-
2	120507738	120507743	Zfp106	-
4	139280528	139280533	Capzb	+
10	80406707	80406712	Uqcr11	-
14	24495832	24495837	Rps24	+
5	125387936	125387941	Ubc	-
3	105954107	105954112	Atp5f1	-
2	173034228	173034233	Rbm38	+
7	12925545	12925550	Rps5	+
2	70023842	70023847	Ubr3	+
15	76347341	76347346	Sharpin	-
11	4702033	4702038	Uqcr10	-
17	71126544	71126549	Myom1;Gm2	+
10	53344920	53344925	Pln	+
10	53344742	53344747	Pln	+
5	129757752	129757757	Nipsnap2	+
14	61534231	61534236	Spryd7	-
18	70617859	70617864	Mbd2	+
9	71505135	71505140	Myzap	-
4	141619265	141619270	Slc25a34	-
13	119335743	119335748	Nnt;Nnt	-
9	64177348	64177353	Rpl4	+
7	80100865	80100870	Idh2	-
3	102145198	102145203	Casq2	+
11	62552395	62552400	Ubb	+
4	10848829	10848834	Gm12918	+
11	6356586	6356591	Ogdh	+
9	108502041	108502046	Usp19	+
19	10905239	10905244	Prpf19	+
2	76704435	76704440	Ttn	-
6	38539191	38539196	Fmc1	+
1	24615122	24615127	Gm28661	-
6	124811288	124811293	Tpi1	-
9	56145825	56145830	Tspan3	-

4	87104971	87104976	Rps6	+
1	64994483	64994488	Rpl10a-ps1	-
10	81182073	81182078	Eef2	+
14	54948129	54948134	Myh6	-
4	155808784	155808789	Mrpl20	+
8	88343643	88343648	Brd7	-
10	80828604	80828609	Oaz1	+
17	46647515	46647520	Mrpl2;Gm26	+
4	45105382	45105387	Tomm5	-
18	61259406	61259411	Rps2-ps10	-
9	50636238	50636243	Dlat	-
9	120014576	120014581	Xirp1;Cx3cr1	-
9	20894361	20894366	Eif3g	-
14	54944480	54944485	Myh6	-
11	94962884	94962889	Sgca	-
14	105682015	105682020	Gm10076	+
7	25630387	25630392	Bckdha	-
7	13035680	13035685	Ube2m	-
7	24888942	24888947	Rps19	+
11	70011071	70011076	Acadvl	-
17	35116302	35116307	Csnk2b	-
4	141424029	141424034	Hspb7	+
8	4255645	4255650	Snapc2	+
10	78164889	78164894	D10Jhu81e	-
6	17340651	17340656	Cav1	+
11	60952248	60952253	Map2k3	+
2	32279068	32279073	Swi5	-
3	107985625	107985630	Gstm2	-
18	67405090	67405095	Afg3l2	-
5	108433816	108433821	Atp5k	-
8	123103925	123103930	Rpl13;Gm20	+
13	55319980	55319985	Rab24	-
11	78522534	78522539	Poldip2	+
9	50596498	50596503	Sdhd	-
11	118301300	118301305	Timp2	-
15	79029400	79029405	H1f0	+
4	147888635	147888640	Mfn2	-
15	76070305	76070310	Puf60	-
2	26008928	26008933	Ubac1	-
8	95864336	95864341	Got2	-
11	70661419	70661424	Eno3	+
6	55348221	55348226	Aqp1	+
8	119324042	119324047	Cdh13	+
2	118880776	118880781	Ivd	+
4	141424129	141424134	Hspb7	+

13	21501237	21501242	Gm11273	-
8	46535112	46535117	Acsl1	+
15	97791623	97791628	Slc48a1	+
2	35296163	35296168	Gsn	+
6	55347645	55347650	Aqp1	+
17	24438963	24438968	Eci1	+
8	70895940	70895945	Rpl18a	-
4	44990409	44990414	Grhpr	+
14	63142660	63142665	Ctsb	+
14	63142978	63142983	Ctsb	+
1	52901237	52901242	Hibch	+
6	13870267	13870272	2610001J05I	-
2	114049644	114049649	Actc1	-
4	42980551	42980556	Vcp	-
13	114351491	114351496	Ndufs4	-
1	45908499	45908504	Slc40a1	-
1	180802804	180802809	H3f3a	-
17	33838170	33838175	Ndufa7	+
13	74331352	74331357	Sdha	-
6	124716776	124716781	Phb2	+
5	118054885	118054890	Tesc	+
9	69487583	69487588	Anxa2	+
8	94180199	94180204	Mt1	+
5	125386012	125386017	Ubc	-
2	38640173	38640178	Psmb7	-
5	21757019	21757024	Pmpcb	+
2	75191418	75191423	Rps6-ps4	-
5	135789214	135789219	Mdh2	+
8	71374529	71374534	Nr2f6	-
19	3908956	3908961	Ndufs8	-
8	70508441	70508446	Uba52;Kxd1	-
1	171290188	171290193	Ufc1	-
11	120488611	120488616	Mrpl12	+
11	52388436	52388441	Vdac1	+
1	140086207	140086212	Cfh	-
4	141424480	141424485	Hspb7	+
11	70526313	70526318	Psmb6	+
10	128084433	128084438	Atp5b	+
4	107904028	107904033	Cpt2	-
7	73776049	73776054	Fam174b	+
8	83583295	83583300	D830024N08	+
1	24615010	24615015	Gm28661	-
7	142376686	142376691	Gm49369;Ct	-
17	48419503	48419508	Apobec2	-
7	25229909	25229914	Gsk3a	-

1	178321840	178321845	Cox20	+
6	87845308	87845313	Cnbp	-
15	73809809	73809814	Ndufb4c;Mro	-
11	69917200	69917205	Eif5a	-
1	43131738	43131743	Fhl2	-
8	68906870	68906875	Lpl	+
9	78479665	78479670	Eef1a1	-
9	22185760	22185765	Rpl15-ps3	-
13	3565604	3565609	Gdi2	+
2	22588380	22588385	Gm13340	-
1	140086268	140086273	Cfh	-
3	108012409	108012414	Gstm1	-
7	13035337	13035342	Ube2m	-
13	119335837	119335842	Nnt;Nnt	-
6	124811098	124811103	Tpi1	-
10	81561268	81561273	Aes	+
18	61259133	61259138	Rps2-ps10	-
9	79755475	79755480	Cox7a2	-
17	15476040	15476045	Psmb1	-
13	74327036	74327041	Sdha	-
17	83502144	83502149	Cox7a2l	-
7	25228658	25228663	Gsk3a	-
11	120560392	120560397	P4hb	-
16	33948812	33948817	Itgb5	+
12	80950306	80950311	Srsf5	+
2	120507101	120507106	Zfp106	-
11	98029503	98029508	Rpl19	+
5	36469468	36469473	Grpel1	+
11	103066027	103066032	Nmt1;Gm266	+
4	109060166	109060171	Nrd1	+
4	57943861	57943866	Txn1	-
9	21008731	21008736	Mrpl4	+
13	74339651	74339656	Sdha	-
13	34978670	34978675	Eci2	-
3	153922593	153922598	Acadm	-
8	121597531	121597536	Map1lc3b;Gr	+
16	23113819	23113824	Eif4a2	+
17	34284417	34284422	H2-Aa	-
14	34561567	34561572	Ldb3	-
11	84821056	84821061	Dhrs11	-
17	17345889	17345894	Oaz1-ps;AC	-
7	120643115	120643120	Uqcrc2	+
5	117092088	117092093	Suds3	-
9	59670611	59670616	Pkm	+
3	58102865	58102870	Pfn2	-

4	140971195	140971200	Sdhb	+
10	13008861	13008866	Sf3b5	+
5	115561170	115561175	Rplp0	+
15	83376154	83376159	Pacsin2	-
7	140112468	140112473	Echs1	-
17	56613935	56613940	Rpl36	+
12	111963309	111963314	Atp5mpl	-
9	44914677	44914682	Atp5l	-
1	171241037	171241042	Ndufs2	-
5	115345661	115345666	Cox6a1	-
9	53581946	53581951	Acat1	-
1	120227865	120227870	Steap3	-
1	54987264	54987269	Sf3b1	-
6	71874487	71874492	Immt	+
2	181150990	181150995	Eef1a2	-
19	29021956	29021961	Ak3	-
16	35313670	35313675	Sec22a	-
5	121598391	121598396	Acad12	-
15	36999964	36999969	Zfp706	-
3	102146010	102146015	Casq2	+
5	93182120	93182125	Ccni	-
6	99877916	99877921	Tpt1-ps3	-
9	123307141	123307146	Scp2-ps2	-
8	13219074	13219079	Lamp1	-
4	139103988	139103993	Minos1	-
12	54180343	54180348	Egln3	-
14	55569280	55569285	Dcaf11	+
1	135848087	135848092	Tnnt2	+
2	148871983	148871988	Cst3	-
7	122090938	122090943	Ndufab1	-
2	74876689	74876694	Mtx2	+
4	139103919	139103924	Minos1	-
2	151511066	151511071	Nsfl1c	+
17	44038567	44038572	Rcan2	+
2	163725458	163725463	Pkig	+
7	139660021	139660026	Gm4459;Cfa	-
17	34718869	34718874	Tnxb	+
11	94659802	94659807	Mrpl27	+
9	31149962	31149967	Aplp2	-
2	90898680	90898685	Ndufs3	-
14	52075105	52075110	Hnrnpc	-
15	35938145	35938150	Cox6c	-
17	56070918	56070923	Ubxn6	-
8	124910444	124910449	Egln1	-
19	43500087	43500092	Got1	-

15	79030019	79030024	H1f0	+
3	131234166	131234171	Hadh	-
7	4519559	4519564	Tnni3	-
13	66901496	66901501	Uqcrb	-
7	73776650	73776655	Fam174b	+
5	138259329	138259334	Lamtor4	+
3	101579648	101579653	Atp1a1	-
14	73364564	73364569	Itm2b	-
9	116040688	116040693	Gm9385	-
5	129753244	129753249	Nipsnap2	+
11	98384781	98384786	Tcap	+
2	64912614	64912619	Grb14	-
6	17340463	17340468	Cav1	+
14	31210576	31210581	Tnnc1	+
8	69894507	69894512	Ndufa13;Yjef	-
18	77781346	77781351	Atp5a1	+
9	78209290	78209295	Gsta4	+
1	171241008	171241013	Ndufs2	-
7	51747664	51747669	Gm7336	+
5	122457518	122457523	Atp2a2	-
1	38996566	38996571	Pdcl3	+
7	111072112	111072117	Eif4g2	-
5	136982251	136982256	Znhit1	-
4	134927213	134927218	Rsrp1	+
1	66830918	66830923	Acadl	-
8	22579000	22579005	Vdac3	-
17	31521671	31521676	Ndufv3	+
4	116639060	116639065	Akr1a1	-
8	126945773	126945778	Tomm20	-
17	71126585	71126590	Myom1;Gm2	+
9	57929744	57929749	Ubl7	+
7	114705544	114705549	Gm15500	-
7	45720786	45720791	Rpl18	+
2	180258377	180258382	Rps21	+
17	87435650	87435655	Calm2	-
7	127015267	127015272	Pagr1a;Gm4	-
10	75937661	75937666	Chchd10	+
18	61730545	61730550	Afap1l1	-
13	100736015	100736020	Mrps36	-
12	113145284	113145289	Crip2	+
1	86530043	86530048	Ptma	+
9	120574110	120574115	Rpl14	+
7	25631346	25631351	Bckdha	-
11	52000354	52000359	Ube2b;Gm2(	-
2	76706922	76706927	Ttn	-

8	95864728	95864733	Got2	-
2	163336699	163336704	Jph2	-
16	8851147	8851152	1810013L24I	+
10	61702590	61702595	Tysnd1	+
11	88211345	88211350	Mrps23	+
11	97711199	97711204	Psmb3	+
5	104087137	104087142	Sparcl1	-
1	43123530	43123535	Fhl2	-
6	146599067	146599072	Fgfr1op2	+
10	29698916	29698921	Gm10275	-
9	44913351	44913356	Atp5l	-
12	31333519	31333524	Dld	-
19	44552840	44552845	Ndufb8	-
13	111593386	111593391	Gpbp1	-
5	97885094	97885099	Antxr2	-
10	128359284	128359289	Cs	+
10	17845486	17845491	Txlnb	+
9	35215549	35215554	Srpr	+
6	5483481	5483486	Pdk4	-
11	23003169	23003174	Cct4	+
1	24615167	24615172	Gm28661	-
7	35547520	35547525	Nudt19	-
10	80341102	80341107	Adamtsl5	-
8	107046670	107046675	Pdf;Cog8	-
6	72369108	72369113	Vamp5	-
5	24409720	24409725	Abcb8	+
5	77087365	77087370	Hopx	-
14	66094789	66094794	Ephx2	-
8	94854796	94854801	Coq9	+
6	87843553	87843558	Cnbp	-
12	51609311	51609316	Scfd1	-
3	90488480	90488485	Snapin	-
7	73776056	73776061	Fam174b	+
7	128206013	128206018	Cox6a2	-
7	25630301	25630306	Bckdha	-
4	147875100	147875105	Mfn2	-
4	15929792	15929797	Decr1	-
5	77349403	77349408	Igfbp7	-
14	101932586	101932591	Lmo7	+
14	21838469	21838474	Vdac2	+
15	73809689	73809694	Ndufb4c;Mro	-
2	121548835	121548840	Frmd5	-
5	77087370	77087375	Hopx	-
9	78478826	78478831	Eef1a1	-
14	18280073	18280078	Nkiras1	+

9	59670572	59670577	Pkm	+
14	29018385	29018390	Lrtm1	+
14	101934669	101934674	Lmo7	+
5	3641379	3641384	Gatad1	-
11	59012712	59012717	Obscn	-
3	51407349	51407354	Ndufc1	-
18	61259444	61259449	Rps2-ps10	-
10	45875484	45875489	Gpx4-ps2	+
10	79711597	79711602	Bsg	+
3	94448000	94448005	Mrpl9	+
3	116581311	116581316	Lrrc39	+
9	119484308	119484313	Scn5a	-
17	25863778	25863783	Mcrip2	-
5	124462237	124462242	Kmt5a	+
9	71479338	71479343	Polr2m	-
9	107565560	107565565	Tusc2	+
10	128351225	128351230	Cs	+
15	90764248	90764253	Nadk2	-
10	80145763	80145768	Atp5d	+
1	92439633	92439638	Ndufa10	-
12	79156333	79156338	Vti1b	-
11	53261619	53261624	Hspa4	-
5	33076223	33076228	Rpl35a-ps5	+
12	112632728	112632733	Adssl1	+
2	119607102	119607107	1700020I14F	+
19	44555010	44555015	Ndufb8	-
3	103058098	103058103	Csde1	+
11	20335610	20335615	Gm12033	-
9	65690191	65690196	Oaz2	+
2	76704740	76704745	Ttn	-
3	86138566	86138571	Rps3a1	-
2	11408910	11408915	A530058N18	-
1	24613048	24613053	Gm28438	-
4	126047118	126047123	Mrps15	+
9	98588869	98588874	Mrps22	-
4	116692921	116692926	Prdx1	+
4	82290716	82290721	Nfib	-
1	171129573	171129578	Sdhc	-
9	64174964	64174969	Rpl4	+
8	95865236	95865241	Got2	-
15	5121024	5121029	Gm10250	-
2	28934497	28934502	Gm13394;Cf	-
3	90512035	90512040	S100a1	-
14	21837870	21837875	Vdac2	+
10	24598141	24598146	Ccn2	+

3	101590679	101590684	Atp1a1	-
7	30227690	30227695	Tbcb	-
5	76967043	76967048	Paics	+
10	128361946	128361951	Cs	+
14	54368474	54368479	Oxa1l	+
7	30553327	30553332	Hspb6	+
9	78478466	78478471	Eef1a1	-
2	158116971	158116976	Tgm2	-
10	127022579	127022584	Tsfm	-
8	84834392	84834397	Gadd45gip1	+
17	35895803	35895808	2310061I04F	-
10	128085155	128085160	Atp5b	+
10	62271626	62271631	Hk1	-
7	45122907	45122912	Rps11	-
10	95490370	95490375	Mrpl42	-
8	70531189	70531194	Fkbp8	+
7	24969804	24969809	Rabac1	-
2	25557968	25557973	Edf1	+
10	81181244	81181249	Eef2	+
7	19415414	19415419	Ckm	+
15	58782888	58782893	Tmem65	-
7	46855618	46855623	Ldha	+
7	4518318	4518323	Tnni3	-
5	35652494	35652499	Htra3	-
7	16455424	16455429	Tmem160	+
17	26213416	26213421	Fam234a	-
15	69093286	69093291	Khdrbs3	+
18	34946563	34946568	Hspa9	-
19	4008765	4008770	Ndufv1	-
10	60302286	60302291	Psap	+
7	142376081	142376086	Gm49369;Ct	-
15	4091181	4091186	Oxct1	+
7	27306702	27306707	Ltbp4	-
11	69396196	69396201	Naa38	+
1	152371784	152371789	Tsen15	-
17	15476153	15476158	Psmb1	-
11	70645854	70645859	Slc25a11	-
2	163336480	163336485	Jph2	-
11	40748606	40748611	Ccng1	-
1	24615277	24615282	Gm28661	-
5	125386173	125386178	Ubc	-
5	23844044	23844049	Tomm7	-
6	42358295	42358300	Zyx	+
7	19151391	19151396	Snrpd2	+
17	46125659	46125664	Mrps18a	+

15	82329753	82329758	Naga	-
1	55081660	55081665	Hspd1	-
3	137379903	137379908	Emcn	+
8	33782841	33782846	Rbpms	-
19	57034091	57034096	Ablim1	-
5	17785844	17785849	Cd36	-
10	80829199	80829204	Oaz1	+
6	83109600	83109605	Gm42688;Mr	+
2	34775133	34775138	Hspa5	+
5	129845925	129845930	Cct6a	+
11	23002375	23002380	Cct4	+
11	115184515	115184520	Nat9	-
4	141422519	141422524	Hspb7	+
2	64912792	64912797	Grb14	-
7	142385466	142385471	Ctsd	-
5	122821141	122821146	Anapc5	-
5	107904880	107904885	Rpl5	+
19	10629184	10629189	Ddb1	+
15	98934389	98934394	Tuba1b;Gm4	-
12	31334687	31334692	Dld	-
14	20704255	20704260	Chchd1	+
19	6908055	6908060	Prdx5	-
2	181148816	181148821	Eef1a2	-
8	107439182	107439187	Rps26-ps1	-
4	91851920	91851925	Gm12671	-
5	129758097	129758102	Nipsnap2	+
6	57689778	57689783	Pyurf	-
19	44553669	44553674	Ndufb8	-
6	72842810	72842815	Kcmf1	-
4	119278311	119278316	Ybx1	-
10	68525699	68525704	Cabcoco1	-
14	21838435	21838440	Vdac2	+
12	78884820	78884825	Eif2s1	+
10	80253758	80253763	Ndufs7	+
6	124810750	124810755	Tpi1	-
9	55512069	55512074	Etfa	-
3	123006238	123006243	Myoz2	-
6	124936007	124936012	Mlf2	+
11	31549296	31549301	Ncoa2	+
2	35304321	35304326	Gsn	+
4	21910593	21910598	Coq3	+
3	105959187	105959192	Atp5f1	-
4	119282848	119282853	Ybx1	-
9	56058609	56058614	Rcn2	+
5	115103376	115103381	Rpl37rt	-

9	40804688	40804693	Hspa8	+
11	101630252	101630257	Rdm1	+
7	73776249	73776254	Fam174b	+
16	31948198	31948203	0610012G03	-
13	74328315	74328320	Sdha	-
19	4036725	4036730	Gstp1	-
5	95862230	95862235	Gm5559	+
2	84433169	84433174	Tfpi	-
3	153922664	153922669	Acadm	-
12	112641271	112641276	Adssl1	+
9	21142226	21142231	Cdc37	-
4	136896317	136896322	C1qa	-
2	101562199	101562204	B230118H07	-
1	134755371	134755376	Ppp1r12b	-
10	79711906	79711911	Bsg	+
9	40803450	40803455	Hspa8	+
17	6997947	6997952	Rnaset2b;Gr	+
3	24333342	24333347	Gm7536	+
6	124705197	124705202	Emg1	-
6	32892350	32892355	Chchd3	-
6	85467580	85467585	Cct7	+
8	83570868	83570873	Ndufb7	+
4	155833028	155833033	Aurkaip1	+
14	25700114	25700119	Ppif	+
12	31331807	31331812	Dld	-
5	121205812	121205817	Rpl6	+
1	92463094	92463099	Ndufa10	-
13	44922115	44922120	Dtnbp1	-
6	73263964	73263969	Suclg1	+
4	40270826	40270831	Ndufb6	-
11	98384548	98384553	Tcap	+
7	103812599	103812604	Hbb-bt	-
11	3919304	3919309	Tcn2	-
10	128084466	128084471	Atp5b	+
8	120672776	120672781	Cox4i1;Gm2	+
3	95660928	95660933	Mcl1	+
5	134619942	134619947	Eif4h	-
6	71124762	71124767	Rpl34-ps1	+
5	115560964	115560969	Rplp0	+
18	35254367	35254372	Ctnna1	+
7	19415041	19415046	Ckm	+
3	150072873	150072878	Rpsa-ps10	-
7	108940910	108940915	Eif3f	+
11	6356533	6356538	Ogdh	+
13	54590928	54590933	Higd2a	+

6	90660579	90660584	Iqsec1	-
9	44410002	44410007	Rps25	+
7	34202427	34202432	Gpi1	-
18	25133325	25133330	Fhod3	+
19	4008598	4008603	Ndufv1	-
2	155276538	155276543	Map1lc3a	+
19	10626607	10626612	Ddb1	+
9	51948585	51948590	Fdx1	-
3	90499637	90499642	Chtop	-
8	46535510	46535515	Acsl1	+
2	122264965	122264970	Sord	+
5	125387997	125388002	Ubc	-
1	4776460	4776465	Mrpl15	-
1	24615144	24615149	Gm28661	-
1	90215214	90215219	Ackr3	+
10	80828914	80828919	Oaz1	+
1	24614665	24614670	Gm10925	-
4	156009916	156009921	Sdf4	+
17	56115218	56115223	Plin5	-
11	59771614	59771619	Mprip	+
1	75367459	75367464	Des	+
11	53430729	53430734	Uqcrq	-
17	66079491	66079496	Ndufv2	-
4	150245314	150245319	Eno1	+
7	126547203	126547208	Eif3c	-
4	140514707	140514712	Arhgef10l	-
11	95831981	95831986	Phospho1;Zf	+
19	6398304	6398309	Pygm	+
10	128361375	128361380	Cs	+
9	54603723	54603728	Idh3a	+
8	34170628	34170633	Saraf	+
11	40748696	40748701	Ccng1	-
14	51905976	51905981	Ndrg2	-
8	104629590	104629595	Rrad	-
3	94944480	94944485	Selenbp1	+
7	99482350	99482355	Rps3	-
11	31548623	31548628	Ncoa2	+
2	174345249	174345254	Gnas	+
3	150073380	150073385	Rpsa-ps10	-
8	124910513	124910518	Egln1	-
17	85063368	85063373	Ppm1b	-
5	110829110	110829115	Hscb	-
10	79711650	79711655	Bsg	+
12	55418410	55418415	Psma6	+
6	24604137	24604142	Lmod2	+

10	78162146	78162151	D10Jhu81e	-
6	125193346	125193351	Mrpl51	+
14	8166227	8166232	Pdhb	-
3	130729057	130729062	Rpl34	-
19	6911231	6911236	Esrra	-
17	29137322	29137327	Rpl35a-ps3	-
14	34527240	34527245	Ldb3	-
2	163469694	163469699	Fitm2	-
6	51464151	51464156	Hnrnpa2b1	-
3	95989014	95989019	Plekho1	-
7	99154096	99154101	Dgat2	-
15	76354143	76354148	Maf1	+
13	11553454	11553459	Ryr2	-
10	121410454	121410459	Rassf3	-
1	167330393	167330398	Tmco1	+
5	5782957	5782962	Gm15459	-
17	12911509	12911514	Mrpl18	-
1	175606147	175606152	Fh1	-
10	3463697	3463702	Ppp1r14c	+
11	58998349	58998354	Obscn	-
5	73633826	73633831	Sgcb	-
10	42534842	42534847	Snx3	+
3	97690147	97690152	Pde4dip	-
11	51601421	51601426	Hnrnpab	-
16	90341823	90341828	Gm49708	-
12	103333462	103333467	Asb2	-
11	23002352	23002357	Cct4	+
19	5426676	5426681	AI837181	+
9	69491587	69491592	Anxa2	+
11	120346686	120346691	Actg1	-
13	107414081	107414086	Ipo11	-
12	28594748	28594753	Colec11	-
12	112654405	112654410	Akt1	-
11	120561153	120561158	P4hb	-
10	78162091	78162096	D10Jhu81e	-
2	76705956	76705961	Ttn	-
6	71124622	71124627	Rpl34-ps1	+
3	94884918	94884923	Psmb4	-
11	21353380	21353385	Ugp2	-
11	115497477	115497482	Jpt1	-
19	34244933	34244938	Acta2	-
6	34306591	34306596	Akr1b3	-
18	35590632	35590637	Matr3	+
7	141459271	141459276	Pnpla2	+
1	66924507	66924512	Myl1	-

6	86513916	86513921	Gm44386	-
2	76705191	76705196	Ttn	-
9	50596427	50596432	Sdhd	-
7	30187441	30187446	Capns1	-
15	75045410	75045415	Ly6c1	-
10	53345976	53345981	Pln	+
17	8297587	8297592	Mpc1	+
7	19088023	19088028	Dmpk	+
8	71374432	71374437	Nr2f6	-
11	53430667	53430672	Uqcrq	-
8	68906836	68906841	Lpl	+
7	19087671	19087676	Dmpk	+
5	112715413	112715418	Myo18b	-
19	24875728	24875733	Gm10053	+
5	115343057	115343062	Triap1	+
4	116306841	116306846	Mast2	-
5	35093076	35093081	Lrpap1	-
7	126795248	126795253	Aldoa	-
17	29137138	29137143	Rpl35a-ps3	-
11	70661171	70661176	Eno3	+
15	44429600	44429605	Eny2	+
2	35302590	35302595	Gsn	+
2	34776011	34776016	Hspa5	+
5	110830950	110830955	Hscb	-
7	80098814	80098819	Idh2	-
8	68905792	68905797	Lpl	+
18	80295632	80295637	Kcng2	-
6	39598296	39598301	Ndufb2	+
16	37834774	37834779	Fstl1	+
9	50637583	50637588	Dlat	-
7	120637945	120637950	Uqcrc2	+
7	29178973	29178978	Psmd8	-
14	55564509	55564514	Dcaf11	+
14	51906170	51906175	Ndrg2	-
17	83504169	83504174	Cox7a2l	-
3	19692045	19692050	Trim55	+
10	80036097	80036102	Polr2e	-
1	118609179	118609184	Clasp1	+
14	51911480	51911485	Ndrg2	-
5	129881355	129881360	Chchd2;Phk(	-
8	104629621	104629626	Rrad	-
5	143564291	143564296	Fam220a;Fa	+
12	31331588	31331593	Dld	-
11	68904493	68904498	Rpl26	+
5	121208488	121208493	Rpl6	+

9	107614919	107614924	Gnai2	-
17	56260004	56260009	Fem1a	+
4	150621667	150621672	Rere	+
12	80634489	80634494	Erh	-
1	191783770	191783775	Lpgat1	+
4	120667365	120667370	Cited4	+
2	180038123	180038128	Psma7	-
19	12767270	12767275	Zfp91	-
1	55078246	55078251	Hspd1	-
9	53589196	53589201	Acat1	-
14	55569517	55569522	Dcaf11	+
19	4009953	4009958	Ndufv1	-
6	52546901	52546906	Hibadh	-
11	50200377	50200382	Sqstm1	-
16	23113631	23113636	Eif4a2	+
7	30185347	30185352	Cox7a1	+
17	12924496	12924501	Tcp1	+
8	124889291	124889296	Gnpat	+
11	5801770	5801775	Pgam2	-
14	25699976	25699981	Ppif	+
5	118056398	118056403	Tesc	+
11	70645182	70645187	Slc25a11	-
15	76354151	76354156	Maf1	+
6	50564899	50564904	Cycs	-
2	108949971	108949976	Gm13910	+
7	80100850	80100855	Idh2	-
3	105955905	105955910	Atp5f1	-
7	105555795	105555800	Smpd1	+
6	86334070	86334075	Gm10443	+
15	81895252	81895257	Aco2	+
8	124908683	124908688	Egln1	-
12	110858381	110858386	Wdr20	-
13	74322313	74322318	Sdha	-
10	81387947	81387952	Dohh;Gm485	+
6	124810995	124811000	Tpi1	-
2	25222966	25222971	Tubb4b	-
18	40205589	40205594	Yipf5	-
14	75847290	75847295	Tpt1	+
4	59618153	59618158	Hsdl2	+
13	23739743	23739748	Hist1h1c	+
15	51787955	51787960	Eif3h	-
9	55485304	55485309	Etfa	-
2	154587890	154587895	Pxmp4	-
8	71404504	71404509	Babam1	+
1	66831358	66831363	Acadl	-

5	5782879	5782884	Gm15459	-
4	141422527	141422532	Hspb7	+
4	49586076	49586081	Tmem246	-
17	5411252	5411257	Tmem242	-
7	19414244	19414249	Ckm	+
11	55395465	55395470	Sparc	-
19	10903654	10903659	Prpf19	+
16	36044077	36044082	Fam162a	-
10	81264952	81264957	Mrpl54	-
2	76705065	76705070	Ttn	-
14	63143112	63143117	Ctsb	+
9	53582511	53582516	Acat1	-
7	19414273	19414278	Ckm	+
3	79605065	79605070	Etfdh	-
10	94199985	94199990	Ndufa12	+
6	126834446	126834451	Ndufa9	-
1	171129639	171129644	Sdhc	-
4	154896475	154896480	Fam213b	-
17	45568514	45568519	Hsp90ab1	-
8	70508296	70508301	Uba52;Kxd1	-
1	180167297	180167302	Coq8a	-
4	140979099	140979104	Sdhb	+
1	36530278	36530283	Ankrd23;Gm	-
14	63143019	63143024	Ctsb	+
5	120627434	120627439	Ddx54	+
9	108337135	108337140	Rhoa	+
13	24818085	24818090	Acot13	-
2	76704310	76704315	Ttn	-
17	48423002	48423007	Apobec2	-
9	121785146	121785151	Hhatl	-
9	107538013	107538018	Tmem115	+
5	139249708	139249713	Sun1	+
17	35832766	35832771	Flot1	+
9	61913325	61913330	Rplp1	-
15	5120762	5120767	Gm10250	-
4	108044662	108044667	Podn;Scp2	-
5	115559845	115559850	Rplp0	+
6	32792561	32792566	Chchd3	-
11	32296869	32296874	Hba-a2	+
7	105809929	105809934	Mrpl17	-
17	29332685	29332690	Mtch1	-
15	4053821	4053826	Oxct1	+
19	7215182	7215187	Cox8a	-
10	81566118	81566123	Aes	+
1	55080178	55080183	Hspd1	-

10	77598056	77598061	Pttg1ip	+
5	24581489	24581494	Abcf2	-
18	80295678	80295683	Kcng2	-
7	118114804	118114809	Rps15a	-
8	84842603	84842608	Calr	-
1	84724468	84724473	Trip12	-
9	44913316	44913321	Atp5l	-
3	153922777	153922782	Acadm	-
18	16589818	16589823	Cdh2	-
8	109672251	109672256	Ist1	-
2	71324058	71324063	Slc25a12	-
1	171239341	171239346	Ndufs2	-
2	150830631	150830636	Pygb	+
4	138313422	138313427	Pink1	-
8	94854727	94854732	Coq9	+
6	57689627	57689632	Gm26712;Py	-
5	135790195	135790200	Mdh2	+
11	69667213	69667218	Eif4a1	-
2	25620442	25620447	Tmem141	-
19	6982558	6982563	Vegfb	-
15	76344942	76344947	Cyc1	+
8	107045513	107045518	Vps4a	+
7	99482592	99482597	Rps3	-
6	135382150	135382155	Emp1	+
3	86141306	86141311	Rps3a1	-
14	46776232	46776237	Cnih1	-
5	31198608	31198613	Snx17	+
9	96895971	96895976	Gm10123	+
11	59205885	59205890	Mrpl55	+
9	21008706	21008711	Mrpl4	+
10	34009070	34009075	Rwdd1	-
6	87843201	87843206	Cnbp	-
14	54469724	54469729	Lrp10	+
14	60779430	60779435	C1qtnf9	+
14	55576743	55576748	Fitm1	+
5	30184479	30184484	Hadhb	+
19	27254021	27254026	Vldlr	+
6	66875634	66875639	Gm9794	-
6	55348102	55348107	Aqp1	+
16	4939581	4939586	Nudt16l1	+
4	149744515	149744520	Slc25a33	-
1	118608917	118608922	Clasp1	+
11	32284371	32284376	Hba-a1	+
14	76507363	76507368	Tsc22d1	+
15	82350290	82350295	Ndufa6	-

8	119345666	119345671	Hsbp1	+
10	56968671	56968676	Gja1	-
7	25629961	25629966	Bckdha	-
1	86543086	86543091	Pde6d	-
9	53582087	53582092	Acat1	-
14	54948297	54948302	Myh6	-
6	135023463	135023468	Ddx47	+
14	73595849	73595854	Sucla2	+
8	70010542	70010547	Gatad2a	-
17	8134996	8135001	Gm49673;Rr	-
11	94328315	94328320	Ankrd40	+
6	52556447	52556452	Hibadh	-
9	40803764	40803769	Hspa8	+
2	103022071	103022076	Pdhx	-
3	75517197	75517202	Pdcd10	-
4	9621860	9621865	Asph	-
12	84377223	84377228	Entpd5	-
8	95864631	95864636	Got2	-
9	20663455	20663460	Pin1	+
19	9984270	9984275	Fth1	+
2	36036623	36036628	Ndufa8	-
9	75345319	75345324	Gnb5	+
19	40292574	40292579	Pdlim1	-
10	45347817	45347822	Bves	+
7	43452830	43452835	Etfb;Gm4523	+
2	91135831	91135836	Mybpc3	+
11	68973732	68973737	Rangrf	-
9	109078241	109078246	Tma7	-
15	55548202	55548207	Mrpl13	-
2	144595227	144595232	Smim26	+
2	90859559	90859564	Mtch2	+
17	13020017	13020022	Sod2	+
3	97695082	97695087	Pde4dip	-
18	74795237	74795242	Acaa2	+
14	69717052	69717057	Chmp7	-
2	14040904	14040909	Hacd1	-
9	89699297	89699302	Tmed3	-
15	37943546	37943551	Gm3362;Rrn	-
1	75215103	75215108	Tuba4a	-
2	156123884	156123889	Nfs1;Gm280	-
3	101582794	101582799	Atp1a1	-
6	125193453	125193458	Mrpl51	+
3	102146425	102146430	Casq2	+
10	128920085	128920090	Rdh5;Bloc1s	-
12	83541614	83541619	Dcaf4	+

11	106073429	106073434	Taco1	+
11	4704252	4704257	Uqcr10	-
13	34994070	34994075	Eci2	-
1	55078285	55078290	Hspd1	-
19	44555350	44555355	Ndufb8	-
1	171238831	171238836	Ndufs2	-
7	102106719	102106724	Art1	+
8	119348229	119348234	Hsbp1	+
14	54947069	54947074	Myh6	-
14	73552871	73552876	Sucla2	+
19	43500477	43500482	Got1	-
5	115345834	115345839	Cox6a1	-
2	13580118	13580123	Vim	+
5	30184396	30184401	Hadhb	+
11	118303703	118303708	Timp2	-
11	119285159	119285164	Gaa	+
2	75191187	75191192	Rps6-ps4	-
6	24522719	24522724	Ndufa5	-
2	14026885	14026890	Hacd1	-
4	139291107	139291112	Capzb	+
12	69183064	69183069	Rpl36al;Gm4	-
1	135768883	135768888	Phlda3	+
3	64014364	64014369	Gmps	+
8	22577104	22577109	Vdac3	-
6	32792804	32792809	Chchd3	-
5	65409039	65409044	Lias	+
14	31211381	31211386	Tnnc1	+
3	132660533	132660538	Aimp1	-
6	112472790	112472795	Cav3	+
1	171234902	171234907	Ndufs2	-
5	122460858	122460863	Atp2a2	-
5	125454743	125454748	Bri3bp	+
2	163725806	163725811	Pkig	+
1	189880783	189880788	Smyd2	-
3	130729233	130729238	Rpl34	-
17	70997952	70997957	Myl12a;Myl1:	-
19	7215223	7215228	Cox8a	-
18	60777065	60777070	Rps14	+
3	102145396	102145401	Casq2	+
3	142303178	142303183	Pdlim5	-
5	115111915	115111920	Acads	-
2	181515261	181515266	Tpd52l2	+
3	32748510	32748515	Ndufb5	+
19	6298595	6298600	Ehd1	+
11	21557423	21557428	Mdh1	-

11	70645794	70645799	Slc25a11	-
8	85718821	85718826	Itfg1	-
19	40243437	40243442	Pdlim1	-
1	135850708	135850713	Tnnt2	+
2	125830820	125830825	Cops2	-
6	71214691	71214696	Smyd1	-
3	153922606	153922611	Acadm	-
1	160199535	160199540	Mrps14	+
4	42980390	42980395	Vcp	-
17	24722211	24722216	Ndufb10	-
2	35181111	35181116	Rab14	-
7	25641741	25641746	Bckdha	-
8	128732600	128732605	Itgb1	+
8	23141189	23141194	Ank1	+
6	39596576	39596581	Gm42420;Nc	+
17	35060377	35060382	Ddah2	+
13	24831323	24831328	Acot13	-
8	27260500	27260505	Eif4ebp1	+
2	91056683	91056688	Psmc3	+
5	124493103	124493108	Rilpl1	-
7	99479331	99479336	Rps3	-
4	126232467	126232472	Map7d1	-
15	73809565	73809570	Ndufb4c;Mro	-
13	58391782	58391787	Hnrnpk	-
11	106073494	106073499	Taco1	+
5	30184461	30184466	Hadhb	+
9	22185748	22185753	Rpl15-ps3	-
4	155074703	155074708	Rer1	-
5	122469600	122469605	Atp2a2	-
7	30554774	30554779	Hspb6	+
8	70504369	70504374	Rex1bd	-
3	105943784	105943789	Atp5f1	-
3	97690110	97690115	Pde4dip	-
17	48419523	48419528	Apobec2	-
3	10205251	10205256	Fabp4	-
11	97161947	97161952	Kpnb1	-
10	81264786	81264791	Mrpl54	-
17	47687947	47687952	Tomm6;Gm2	-
11	58932457	58932462	Rnf187	-
11	87583991	87583996	Sept4	+
7	142377425	142377430	Gm49369;Ct	-
3	102145663	102145668	Casq2	+
2	32279026	32279031	Swi5	-
6	32792886	32792891	Chchd3	-
11	48805543	48805548	Rack1	+

6	6041612	6041617	Slc25a13	-
14	8169402	8169407	Pdhb	-
13	49194942	49194947	Ninj1	+
3	10016252	10016257	Fabp5	+
3	108012616	108012621	Gstm1	-
17	47686953	47686958	Tomm6;Gm2	-
6	115807136	115807141	Rpl32	-
8	78505319	78505324	Rbmxl1	-
8	83995338	83995343	Prkaca	+
6	50565006	50565011	Cycs	-
15	4153779	4153784	Oxct1	+
2	30405396	30405401	Crat	-
10	75771425	75771430	Ddt	-
7	101822878	101822883	Inppl1	-
5	122461886	122461891	Atp2a2	-
11	78290014	78290019	2610507B11	+
11	31548930	31548935	Ncoa2	+
1	85850418	85850423	Cab39	+
11	48801660	48801665	Rack1	+
7	140099606	140099611	Fuom	-
4	101153238	101153243	Jak1	-
16	18301164	18301169	Tango2	-
6	71124659	71124664	Rpl34-ps1	+
16	90341916	90341921	Gm49708	-
8	46534823	46534828	Acsl1	+
14	50915856	50915861	Osgep	-
4	49514238	49514243	Mrpl50	-
9	53583488	53583493	Acat1	-
2	174345735	174345740	Gnas	+
7	28788261	28788266	Sirt2	+
13	24817954	24817959	Acot13	-
19	5493974	5493979	Cfl1	+
2	25621669	25621674	Tmem141	-
6	87843598	87843603	Cnbp	-
8	83570977	83570982	Ndufb7	+
17	56279746	56279751	Plin3	-
7	45459777	45459782	Ftl1	-
5	77351977	77351982	Igfbp7	-
2	163337354	163337359	Jph2	-
1	92439858	92439863	Ndufa10	-
3	144597324	144597329	Selenof	+
9	71478857	71478862	Polr2m	-
17	12924317	12924322	Tcp1	+
8	116982534	116982539	Gcsh	-
11	68943274	68943279	Arhgef15	-

5	124493625	124493630	Rilpl1	-
19	59322437	59322442	Rps12-ps3	+
7	19414237	19414242	Ckm	+
9	20645636	20645641	Ubl5	+
17	24433226	24433231	Eci1	+
5	17781913	17781918	Cd36	-
17	33824664	33824669	Ndufa7	+
4	19606133	19606138	Rmdn1;Gm1	+
4	116693834	116693839	Prdx1	+
2	118475930	118475935	Srp14	-
3	138556129	138556134	Eif4e	+
8	46207231	46207236	Slc25a4	-
17	33996247	33996252	H2-K1	-
15	98931664	98931669	Tuba1b	-
9	102634087	102634092	Anapc13	+
9	4296724	4296729	Aasdhppt	-
18	77777564	77777569	Atp5a1	+
5	88659923	88659928	Grsf1	-
10	84613719	84613724	Tcp11l2	+
8	85336425	85336430	Mylk3	-
15	98127731	98127736	Pfkm	+
8	71535804	71535809	Bst2	-
15	10447097	10447102	Dnajc21	-
4	149896322	149896327	Spsb1	-
15	25973432	25973437	Retreg1	+
5	30176891	30176896	Hadhb	+
5	92409282	92409287	Art3	+
7	51747523	51747528	Gm7336	+
2	125830696	125830701	Cops2	-
8	84246561	84246566	D8Ertd738e	-
11	6356250	6356255	Ogdh	+
13	119374741	119374746	Nnt;Nnt	-
11	70661039	70661044	Eno3	+
12	12912093	12912098	Rpl36-ps3;G	+
1	169695042	169695047	Rgs5	+
8	70895700	70895705	Rpl18a	-
2	122052458	122052463	Eif3j1	+
7	13032311	13032316	Chmp2a	-
14	51910375	51910380	Ndrg2	-
19	5493523	5493528	Cfl1	+
2	29739985	29739990	Rapgef1	+
14	55576340	55576345	Fitm1	+
7	19091819	19091824	Dmpk	+
8	107262935	107262940	Rps18-ps3	-
7	132557966	132557971	Oat;Fgfr2	-

2	14035909	14035914	Hacd1	-
8	84705647	84705652	Nfix	-
14	51908355	51908360	Ndrg2	-
4	116641052	116641057	Akr1a1	-
17	26839098	26839103	Nkx2-5	-
17	23675746	23675751	Tnfrsf12a	-
16	90341941	90341946	Gm49708	-
18	84097205	84097210	Zadh2	+
3	152237863	152237868	Nexn	-
11	51997848	51997853	Ube2b;Gm26	-
3	90511226	90511231	S100a1	-
19	5423844	5423849	Drap1	-
11	84828790	84828795	Dhrs11	-
7	13032793	13032798	Chmp2a	-
7	30554986	30554991	Hspb6	+
17	35822468	35822473	Ier3	+
10	81181979	81181984	Eef2	+
18	34345129	34345134	Reep5	-
17	56614453	56614458	Lonp1	-
19	56323946	56323951	Nrap	-
2	73908450	73908455	Atp5g3	-
7	99479428	99479433	Rps3	-
17	56101616	56101621	Plin4	-
1	64994321	64994326	Rpl10a-ps1	-
3	123034229	123034234	Myoz2	-
13	12270846	12270851	Actn2	-
3	108014778	108014783	Gstm1	-
1	175614744	175614749	Fh1	-
5	115110787	115110792	Acads	-
11	59012327	59012332	Obscn	-
12	112654566	112654571	Akt1	-
9	89702836	89702841	Tmed3	-
2	130281264	130281269	Idh3b	-
19	6390950	6390955	Pygm	+
10	40287851	40287856	Amd1	-
2	174345283	174345288	Gnas	+
10	86732079	86732084	Fabp3-ps1	-
2	181670349	181670354	Sox18	-
10	56390015	56390020	Gja1	+
7	35643706	35643711	Pdcd5	-
15	75896391	75896396	Eef1d	-
19	4131566	4131571	Tmem134	+
1	118609166	118609171	Clasp1	+
10	17845278	17845283	Txlnb	+
6	116652126	116652131	Depp1	+

1	180169449	180169454	Coq8a	-
13	73320056	73320061	Ndufs6	-
10	117045427	117045432	Lrrc10	+
13	14610584	14610589	Mrpl32	-
6	136872484	136872489	Mgp	-
2	122150924	122150929	B2m	+
11	43424740	43424745	Pttg1	-
11	6616562	6616567	Tbrg4	-
19	6390414	6390419	Pygm	+
4	132530805	132530810	Atpif1	-
9	50596843	50596848	Sdhd	-
17	25185194	25185199	BC003965	+
2	144250851	144250856	Snx5	-
4	129593343	129593348	Eif3i	-
11	74669626	74669631	Cluh	+
17	56017038	56017043	Sh3gl1	-
5	21796609	21796614	Psmc2	+
19	5427120	5427125	AI837181	+
12	113144470	113144475	Crip2	+
2	84765783	84765788	Serping1	-
17	46647463	46647468	Mrpl2;Gm26‹	+
11	119293597	119293602	Eif4a3	-
19	6398149	6398154	Pygm	+
6	88842696	88842701	Podxl2	-
11	31548880	31548885	Ncoa2	+
7	3620070	3620075	Ndufa3	+
7	111071290	111071295	Eif4g2	-
1	75367227	75367232	Des	+
3	20125343	20125348	Gyg	-
12	17284235	17284240	Pdia6	+
17	5411115	5411120	Tmem242	-
9	50636047	50636052	Dlat	-
11	70012188	70012193	Acadvl	-
12	103321443	103321448	Asb2	-
19	37295356	37295361	Rpl10-ps6;Id	-
15	79146131	79146136	Polr2f	+
17	46017248	46017253	Vegfa	-
14	51905983	51905988	Ndrg2	-
11	54870334	54870339	Hint1	+
1	172274557	172274562	Atp1a2	-
9	106682036	106682041	Tex264	-
19	4194599	4194604	Ppp1ca	+
16	31456871	31456876	Bdh1	+
9	53582117	53582122	Acat1	-
15	99409582	99409587	Tmbim6	+

10	80392877	80392882	Mbd3	-
6	52546495	52546500	Hibadh	-
8	107580787	107580792	Psmd7	-
4	141424568	141424573	Hspb7	+
7	130764534	130764539	Tacc2	+
3	153922727	153922732	Acadm	-
11	96047348	96047353	Snf8	+
14	19812371	19812376	Rtraf	-
7	141010516	141010521	Ifitm3	-
5	147078648	147078653	Polr1d	+
2	74869410	74869415	Mtx2	+
1	135850731	135850736	Tnnt2	+
5	22612835	22612840	Rpl17-ps5	-
3	90614326	90614331	S100a6	+
17	33952473	33952478	Rps18	-
6	50565098	50565103	Cycs	-
4	123718126	123718131	Ndufs5	-
14	29027727	29027732	Lrtm1	+
4	141577109	141577114	Fblim1	-
2	36036373	36036378	Ndufa8	-
10	53344513	53344518	Pln	+
5	138163233	138163238	Cops6	+
8	70535092	70535097	Fkbp8	+
7	142376857	142376862	Gm49369;Ct	-
5	135786318	135786323	Mdh2	+
1	64994012	64994017	Rpl10a-ps1	-
13	11553469	11553474	Ryr2	-
7	141458598	141458603	Pnpla2	+
2	121551157	121551162	Frmd5	-
13	13512070	13512075	Nid1	+
5	115296594	115296599	Coq5	+
1	164823806	164823811	Dpt	+
11	76986694	76986699	Blmh	+
7	142009027	142009032	Mob2	-
7	99345692	99345697	Serpinh1	-
11	70658649	70658654	Eno3	+
10	80054743	80054748	Gpx4	+
14	63146680	63146685	Fdft1	-
6	55347242	55347247	Aqp1	+
9	106215026	106215031	Twf2	+
4	116699261	116699266	Prdx1	+
10	128361427	128361432	Cs	+
14	55581499	55581504	Psme1	+
3	24333541	24333546	Gm7536	+
16	20662096	20662101	Psmd2	+

4	151048277	151048282	Vamp3;Camt	-
17	31531122	31531127	Ndufv3	+
2	25620412	25620417	Tmem141	-
9	25307662	25307667	Sept7	+
9	102586729	102586734	Cep63	-
17	33952087	33952092	Rps18	-
5	122444118	122444123	Anapc7	+
3	135463027	135463032	Ube2d3	+
6	124810959	124810964	Tpi1	-
11	101581558	101581563	Nbr1	+
11	96073650	96073655	Atp5g1	-
13	91859304	91859309	Ckmt2	-
11	48803885	48803890	Rack1	+
8	121597625	121597630	Map1lc3b;Gr	+
16	4044079	4044084	Trap1	-
8	119410803	119410808	Mlycd	+
7	46850193	46850198	Ldha	+
13	86046687	86046692	Cox7c	-
3	142303163	142303168	Pdlim5	-
3	138556086	138556091	Eif4e	+
19	47857942	47857947	Gsto1	+
15	75045052	75045057	Ly6c1	-
1	52905406	52905411	Hibch	+
19	21276448	21276453	Zfand5	+
4	48672981	48672986	Cavin4	+
16	4937974	4937979	Mgrn1	+
10	128490999	128491004	Myl6	-
2	32633296	32633301	Ak1	+
11	100361939	100361944	Gm10039	-
10	128362248	128362253	Cs	+
10	80143877	80143882	Atp5d	+
5	115802032	115802037	Gm13841	-
5	24803637	24803642	Rheb	-
17	71252350	71252355	Emilin2	-
17	13010572	13010577	Sod2	+
2	76705715	76705720	Ttn	-
2	114062021	114062026	C130080G1(	+
17	24164008	24164013	Atp6v0c	-
19	11047833	11047838	AW112010	-
8	15133332	15133337	Myom2	+
15	81904852	81904857	Aco2	+
16	91925388	91925393	Atp5o;Atp5o	-
12	100123281	100123286	Psmc1	+
17	33966361	33966366	Vps52	+
17	48419537	48419542	Apobec2	-

10	13515545	13515550	Fuca2	+
18	60776515	60776520	Rps14	+
5	36471267	36471272	Grpel1	+
10	45369679	45369684	Bves	+
4	134927026	134927031	Rsrp1	+
19	57033642	57033647	Ablim1	-
12	72652248	72652253	Dhrs7	-
9	108684377	108684382	Slc25a20	+
8	123891830	123891835	Acta1	-
10	80056196	80056201	Gpx4	+
14	37121557	37121562	Ghitm	-
15	83376873	83376878	Pacsin2	-
18	60812302	60812307	Cd74	+
10	115287525	115287530	Rab21	-
7	43454539	43454544	Etfb;Gm4523	+
6	73260513	73260518	Suclg1	+
3	68626347	68626352	Iqschfp;Schip	+
11	101418917	101418922	Ptges3l;Gm2	-
7	24862594	24862599	Gm9844	+
17	24722655	24722660	Ndufb10	-
9	108205262	108205267	Dag1	-
6	87999404	87999409	Rab7	-
13	66997440	66997445	Ptdss1	+
4	141421794	141421799	Hspb7	+
1	79778037	79778042	Mrpl44	+
9	40803107	40803112	Hspa8	+
8	119956941	119956946	Usp10;Gm2(	+
14	51905331	51905336	Ndrg2	-
8	72181030	72181035	Rab8a	+
17	17345457	17345462	Oaz1-ps;AC	-
6	48701848	48701853	Gimap6	-
3	105942741	105942746	Atp5f1	-
11	86689976	86689981	Ptrh2	+
4	42980450	42980455	Vcp	-
2	152185324	152185329	Rps15a-ps7	-
3	123013650	123013655	Myoz2	-
18	74805926	74805931	Acaa2	+
11	93952887	93952892	Nme2;Gm20	-
11	62552714	62552719	Ubb	+
1	74287316	74287321	Pnkd	+
19	7426743	7426748	Rtn3	-
8	72153031	72153036	Tpm4	+
7	127893185	127893190	Gm21974;Vk	-
8	124636456	124636461	2310022B05l	-
7	105809854	105809859	Mrpl17	-

10	79688056	79688061	Cdc34	+
2	157522034	157522039	Gm14279	-
2	90853063	90853068	Mtch2	+
10	120215630	120215635	Tmbim4	+
8	95713277	95713282	Ndrg4	+
19	24875913	24875918	Gm10053	+
7	12922974	12922979	Rps5	+
4	116990311	116990316	Urod	-
7	30554471	30554476	Hspb6	+
9	55485311	55485316	Etfa	-
9	53581743	53581748	Acat1	-
7	34202874	34202879	Gpi1	-
1	43153155	43153160	Fhl2	-
11	69667570	69667575	Eif4a1	-
9	22186069	22186074	Rpl15-ps3	-
10	116485376	116485381	Cnot2	-
17	86998462	86998467	Rhoq	+
5	104065168	104065173	Nudt9	+
19	6982619	6982624	Vegfb	-
17	56259495	56259500	Fem1a	+
19	5493838	5493843	Cfl1	+
9	40804274	40804279	Hspa8	+
11	115607138	115607143	Mrps7	+
4	40732962	40732967	Dnaja1	+
1	171241105	171241110	Ndufs2	-
4	123127175	123127180	Ppie	-
8	128732343	128732348	Itgb1	+
5	114250295	114250300	Acacb	+
14	19812354	19812359	Rtraf	-
2	91130899	91130904	Mybpc3	+
13	23740027	23740032	Hist1h1c	+
7	90208145	90208150	Picalm	+
11	95671455	95671460	Phb;Gm2683	+
4	40946252	40946257	Bag1	-
5	21748879	21748884	Pmpcb	+
8	23148344	23148349	Ank1	+
8	85350367	85350372	Mylk3	-
14	24491783	24491788	Rps24	+
7	141471206	141471211	Cd151	+
14	73363089	73363094	Itm2b	-
11	109668589	109668594	Prkar1a	+
2	163725928	163725933	Pkig	+
10	81559568	81559573	Aes	+
11	107494686	107494691	Psmd12	+
7	25228727	25228732	Gsk3a	-

9	66066335	66066340	Ppib	+
5	137782057	137782062	Mepce	-
2	177478392	177478397	Zfp970	+
6	116174165	116174170	Gm8203	+
17	33709960	33709965	March2	-
2	76707281	76707286	Ttn	-
6	124913977	124913982	Ptms	-
19	6393866	6393871	Pygm	+
4	62394809	62394814	Cdc26	-
13	59755984	59755989	Isca1	-
6	88000141	88000146	Rab7	-
9	86581509	86581514	Me1	-
3	122925601	122925606	1810037I17F	+
19	42034128	42034133	Ubtd1	+
7	144930907	144930912	Ccnd1	-
3	133330460	133330465	Ppa2	+
9	59679217	59679222	Pkm	+
12	81499415	81499420	Gm20498;Sy	-
11	119288905	119288910	Eif4a3	-
9	57532392	57532397	Cox5a	+
8	13146655	13146660	Cul4a	+
19	5456721	5456726	Ccdc85b	-
14	14112294	14112299	Psmd6	-
3	86141733	86141738	Rps3a1	-
7	111072256	111072261	Eif4g2	-
9	55454744	55454749	Etfa	-
8	78486074	78486079	Gm2225	-
10	97517998	97518003	Dcn	+
11	16504765	16504770	Sec61g	-
6	125306889	125306894	Ltbr	-
14	120431042	120431047	Mbnl2	+
4	147987173	147987178	Nppb	+
9	52088504	52088509	Rdx	+
19	8978049	8978054	Eef1g	+
5	100547586	100547591	Cops4	+
18	35253962	35253967	Ctnna1	+
14	54614370	54614375	Psmb5	-
5	108121691	108121696	Tmed5	-
17	31531185	31531190	Ndufv3	+
9	108339513	108339518	Gpx1	+
4	126048654	126048659	Mrps15	+
7	78775587	78775592	Mrpl46	-
5	17798257	17798262	Cd36	-
15	76171185	76171190	Plec	-
10	128323400	128323405	Cnpy2	+

10	76709117	76709122	Col6a1	-
5	100804386	100804391	Mrps18c	+
8	85082036	85082041	Wdr83os	+
11	40750365	40750370	Ccng1	-
11	69994811	69994816	Gabarap	+
12	65066642	65066647	Fkbp3	-
8	23148930	23148935	Ank1	+
6	86524820	86524825	Pcbp1	-
3	57834714	57834719	Rnf13	+
8	121597761	121597766	Map1lc3b;Gr	+
7	36118505	36118510	Rpl17-ps9	+
5	8067556	8067561	Sri	+
2	30173939	30173944	Endog	+
11	120484736	120484741	Mrpl12	+
7	128546517	128546522	Bag3	+
13	74323074	74323079	Sdha	-
11	21323248	21323253	Ugp2	-
7	105742734	105742739	Ilk	+
4	116699325	116699330	Prdx1	+
3	103058105	103058110	Csde1	+
11	120605587	120605592	Anapc11	+
12	103323869	103323874	Asb2	-
18	60777825	60777830	Rps14	+
13	91865252	91865257	Ckmt2	-
14	20501481	20501486	Ppp3cb	-
7	126492084	126492089	Atxn2l	-
11	74674362	74674367	Pafah1b1	-
10	121397105	121397110	Gns	+
2	76704123	76704128	Ttn	-
16	18777102	18777107	Cldn5	+
7	25705020	25705025	Tgfb1	+
7	114047722	114047727	Rras2	-
10	81208900	81208905	Atcayos	+
1	63147131	63147136	Ndufs1	-
1	24614716	24614721	Gm10925	-
6	137751433	137751438	Strap	+
4	63550237	63550242	Atp6v1g1	+
11	115646747	115646752	Grb2	-
13	23740089	23740094	Hist1h1c	+
17	56258803	56258808	Fem1a	+
6	51463487	51463492	Hnrnpa2b1	-
10	80665411	80665416	Mknk2	-
6	142495727	142495732	Ldhb	-
12	103321670	103321675	Asb2	-
1	153909298	153909303	Glul	+

15	81907517	81907522	Aco2	+
12	83993641	83993646	Acot2	+
8	94844880	94844885	Coq9	+
9	108947903	108947908	Uqcrc1	+
5	5781838	5781843	Gm15459	-
2	28470914	28470919	Mrps2	+
11	120646234	120646239	Myadml2	-
7	141471441	141471446	Cd151	+
17	43456161	43456166	Adgrf5	+
11	69653010	69653015	Fxr2	+
9	121454921	121454926	Trak1	+
1	175614840	175614845	Fh1	-
6	52766377	52766382	Tax1bp1	+
6	97248005	97248010	Lmod3	-
13	86045727	86045732	Cox7c	-
10	81177744	81177749	Eef2	+
15	100621632	100621637	Smagp	-
11	96817706	96817711	Nfe2l1	-
8	123242815	123242820	Vps9d1	-
11	94291304	94291309	Luc7l3	-
14	20455473	20455478	Anxa7	-
2	127284170	127284175	Stard7	+
10	13507401	13507406	Fuca2	+
14	8166340	8166345	Pdhb	-
10	21368580	21368585	Hbs1l	+
4	117155622	117155627	Rps8	-
11	59212570	59212575	Arf1	-
10	95542740	95542745	Ube2n	+
13	24820784	24820789	Acot13	-
11	88210980	88210985	Mrps23	+
5	95862126	95862131	Gm5559	+
11	68973796	68973801	Rangrf	-
3	10204357	10204362	Fabp4	-
6	147070802	147070807	Mrps35	+
17	40961216	40961221	Mut	+
9	50596838	50596843	Sdhd	-
10	91119699	91119704	Slc25a3	-
1	175612182	175612187	Fh1	-
15	81913679	81913684	Aco2	+
10	79711756	79711761	Bsg	+
11	95026391	95026396	Pdk2	-
9	56058340	56058345	Rcn2	+
1	169695476	169695481	Rgs5	+
9	22185950	22185955	Rpl15-ps3	-
3	90614231	90614236	S100a6	+

10	117278005	117278010	Lyz2	-
1	175606085	175606090	Fh1	-
6	73276709	73276714	Suclg1	+
2	120091003	120091008	Ehd4	-
2	91134568	91134573	Mybpc3	+
16	18407587	18407592	Comt	-
3	144697443	144697448	Sh3glb1	-
5	30673415	30673420	Cenpa	+
7	140108131	140108136	Echs1	-
13	93050165	93050170	Cmya5	-
17	56111922	56111927	Plin5	-
9	67023294	67023299	Tpm1	-
12	79160527	79160532	Vti1b	-
8	124948319	124948324	Egln1	-
14	66086972	66086977	Ephx2	-
14	54268057	54268062	Abhd4	+
5	129758010	129758015	Nipsnap2	+
4	46115127	46115132	Tmod1	+
7	30554527	30554532	Hspb6	+
11	40749017	40749022	Ccng1	-
2	38640194	38640199	Psmb7	-
12	84314181	84314186	Ptgr2	+
7	3617390	3617395	Ndufa3	+
1	178330172	178330177	Hnrnpu	-
3	150072743	150072748	Rpsa-ps10	-
8	11449195	11449200	Col4a2	+
7	45458045	45458050	Ftl1	-
18	74798373	74798378	Acaa2	+
12	113145002	113145007	Crip2	+
17	24639786	24639791	Slc9a3r2	-
13	30541037	30541042	Uqcrfs1	-
1	180166825	180166830	Coq8a	-
1	36530404	36530409	Ankrd23;Gm	-
5	129748005	129748010	Nipsnap2	+
7	62476628	62476633	Atp5l-ps1	+
4	127247461	127247466	Smim12	+
10	29698827	29698832	Gm10275	-
9	96471267	96471272	Rnf7	-
2	4938536	4938541	Phyh	+
5	36795065	36795070	Mrfap1	-
9	22092874	22092879	Gm6581	+
6	71214422	71214427	Smyd1	-
5	122453516	122453521	Atp2a2	-
1	118608573	118608578	Clasp1	+
7	45160042	45160047	Pih1d1	+

5	129757954	129757959	Nipsnap2	+
19	4194563	4194568	Ppp1ca	+
18	74805983	74805988	Acaa2	+
14	55576406	55576411	Fitm1	+
4	119279049	119279054	Ybx1	-
8	70895987	70895992	Rpl18a	-
14	21033604	21033609	Vcl	+
7	114267150	114267155	Psma1	-
18	35254027	35254032	Ctnna1	+
9	106215163	106215168	Twf2	+
2	163469753	163469758	Fitm2	-
9	64177976	64177981	Rpl4	+
12	54179665	54179670	Egln3	-
12	100123109	100123114	Psmc1	+
9	50636436	50636441	Dlat	-
15	74748155	74748160	Lynx1	-
9	64176984	64176989	Rpl4	+
3	27244354	27244359	Nceh1	+
19	5493573	5493578	Cfl1	+
14	51908088	51908093	Ndrg2	-
5	31137556	31137561	Trim54	+
7	43452799	43452804	Etfb;Gm4523	+
12	16985425	16985430	Rock2	+
10	17845561	17845566	Txlnb	+
10	79688308	79688313	Cdc34	+
9	65689323	65689328	Oaz2	+
2	112363155	112363160	Emc4	-
9	20646718	20646723	Ubl5	+
8	68906571	68906576	Lpl	+
12	54645521	54645526	Sptssa	-
16	37836227	37836232	Fstl1	+
5	135790093	135790098	Mdh2	+
11	94339707	94339712	Ankrd40	+
5	104087055	104087060	Sparcl1	-
15	77915285	77915290	Txn2	-
2	76704835	76704840	Ttn	-
1	135847979	135847984	Tnnt2	+
1	151363781	151363786	Ivns1abp	+
4	138242986	138242991	Hp1bp3	+
11	100362117	100362122	Gm10039	-
2	84767298	84767303	Serping1	-
11	93959296	93959301	Gm20390;Nr	-
4	147873778	147873783	Mfn2	-
3	101579790	101579795	Atp1a1	-
7	126786052	126786057	Ppp4c	-

17	24438994	24438999	Eci1	+
11	96854615	96854620	Copz2	+
9	120960395	120960400	Ctnnb1	+
11	54866471	54866476	Hint1	+
5	138259077	138259082	Lamtor4	+
11	115415837	115415842	Atp5h	-
3	144691272	144691277	Sh3glb1	-
17	46147874	46147879	Mad2l1bp	-
9	40803002	40803007	Hspa8	+
2	34776430	34776435	Hspa5	+
19	34811157	34811162	Pank1	-
6	122457339	122457344	Gm8430	+
4	134937295	134937300	Syf2	+
13	23739953	23739958	Hist1h1c	+
19	44553679	44553684	Ndufb8	-
10	77990694	77990699	Pfkl	-
10	111496594	111496599	Nap1l1	+
4	154896551	154896556	Fam213b	-
9	123156825	123156830	Clec3b	+
1	169695698	169695703	Rgs5	+
5	100655104	100655109	Coq2	-
9	114757096	114757101	Cmtm7	-
11	106782613	106782618	Ddx5	-
10	128048277	128048282	Naca	+
1	161241343	161241348	Prdx6	-
1	36531106	36531111	Ankrd23;Gm	-
10	128626086	128626091	Rps26	-
2	32402551	32402556	Ptges2	+
3	101579814	101579819	Atp1a1	-
10	128492082	128492087	Myl6	-
7	78775392	78775397	Mrpl46	-
7	130981676	130981681	Htra1	+
14	120946812	120946817	Ipo5	+
2	130284384	130284389	Idh3b	-
5	88659887	88659892	Grsf1	-
6	71215745	71215750	Smyd1	-
11	54870479	54870484	Hint1	+
9	50756523	50756528	Cryab	+
13	74323121	74323126	Sdha	-
7	126696202	126696207	Bola2	+
1	171129411	171129416	Sdhc	-
4	141425281	141425286	Hspb7	+
3	90514965	90514970	S100a13	+
15	96687955	96687960	Slc38a2	-
10	29698749	29698754	Gm10275	-

11	115431127	115431132	Kctd2	+
12	35145375	35145380	Snx13	+
2	119172197	119172202	Gchfr	+
3	108676904	108676909	Clcc1	+
8	126934912	126934917	Tomm20	-
15	5120657	5120662	Gm10250	-
11	94951741	94951746	Col1a1	+
7	45125955	45125960	Rpl13a;Gm4!	-
17	35149243	35149248	Prrc2a	-
2	74876508	74876513	Mtx2	+
6	72369090	72369095	Vamp5	-
17	46928732	46928737	Ubr2	-
12	8498040	8498045	Rhob	-
1	157412892	157412897	2810025M15	+
7	16809754	16809759	Fkrp	-
15	27593858	27593863	Ank	+
9	101105787	101105792	Ppp2r3a	-
3	8802239	8802244	Mrps28	-
17	15476017	15476022	Psmb1	-
11	88830837	88830842	Akap1	-
9	110983845	110983850	Lrrc2	+
10	82620161	82620166	1190007I07F	-
12	8938641	8938646	Laptm4a	+
1	172195219	172195224	Dcaf8	+
15	35937242	35937247	Cox6c	-
1	151361575	151361580	Ivns1abp	+
14	55663821	55663826	Nedd8	-
2	76706826	76706831	Ttn	-
7	30555221	30555226	Hspb6	+
3	94886483	94886488	Psmb4	-
4	137569921	137569926	Hspg2	+
3	58592264	58592269	Selenot	+
14	37121050	37121055	Ghitm	-
4	136255158	136255163	Tcea3	+
4	55390631	55390636	Rad23b	+
10	79888862	79888867	Gm19810	+
3	86141698	86141703	Rps3a1	-
1	180167197	180167202	Coq8a	-
9	44920639	44920644	Atp5l	-
17	56259784	56259789	Fem1a	+
6	29470255	29470260	Atp6v1f	+
2	108950701	108950706	Gm13910	+
5	145183713	145183718	Atp5j2	-
14	60780087	60780092	C1qtnf9	+
10	97506594	97506599	Dcn	+

11	51624536	51624541	Rmnd5b	-
11	62552404	62552409	Ubb	+
11	6355643	6355648	Ogdh	+
3	146505496	146505501	Gng5	+
9	59668665	59668670	Pkm	+
13	24818056	24818061	Acot13	-
14	60903580	60903585	Mipep	+
5	135735046	135735051	Por	+
5	138163924	138163929	Cops6	+
11	6356405	6356410	Ogdh	+
10	62270031	62270036	Hk1	-
2	163726064	163726069	Pkig	+
11	101247646	101247651	Ramp2	+
9	59679049	59679054	Pkm	+
8	33783202	33783207	Rbpms	-
13	46749440	46749445	Kif13a	-
14	105682143	105682148	Gm10076	+
19	43500007	43500012	Got1	-
1	63148421	63148426	Ndufs1	-
6	97184102	97184107	Uba3	-
6	124739309	124739314	Grcc10;Gm4	-
15	76070344	76070349	Puf60	-
6	115954957	115954962	Plxnd1	-
9	57544863	57544868	Mpi	-
7	34192890	34192895	Pdcd2l	-
5	30921109	30921114	Emilin1	+
11	115523337	115523342	Sumo2	-
4	149744602	149744607	Slc25a33	-
3	123006545	123006550	Myoz2	-
9	120128578	120128583	Rpsa	+
4	44987243	44987248	Grhpr	+
4	129143631	129143636	Fndc5	+
2	114050376	114050381	Actc1	-
13	91859201	91859206	Ckmt2	-
3	153922524	153922529	Acadm	-
13	12282612	12282617	Actn2	-
8	94394905	94394910	Herpud1	+
1	43141796	43141801	Fhl2	-
4	9624369	9624374	Asph	-
18	50090644	50090649	Tnfaip8	+
7	48839433	48839438	Csrp3	-
1	36531629	36531634	Ankrd23;Gm	-
4	15917253	15917258	Decr1	-
3	34077479	34077484	Dnajc19	-
7	43457764	43457769	Etfb;Gm4523	+

17	33996412	33996417	H2-K1	-
5	23839797	23839802	Tomm7	-
10	62269554	62269559	Hk1	-
14	34567535	34567540	Ldb3	-
2	156567860	156567865	Aar2	+
15	58936414	58936419	Ndufb9;Gm4	+
4	9576197	9576202	Asph	-
11	58994765	58994770	Obscn	-
15	73751185	73751190	Ptp4a3	+
5	31622377	31622382	Mrpl33;Gm4;	+
2	119655659	119655664	Ndufaf1	-
6	99878198	99878203	Tpt1-ps3	-
11	75764957	75764962	Ywhae	+
11	58997187	58997192	Obscn	-
17	24720442	24720447	Rps2	+
6	50564860	50564865	Cycs	-
5	145108688	145108693	Arpc1a	+
7	80096029	80096034	Idh2	-
2	50280072	50280077	Mmadhc	-
19	27253923	27253928	Vldlr	+
8	93972093	93972098	Amfr	-
5	31616393	31616398	Mrpl33;Gm4;	+
19	32466093	32466098	Rpl9-ps6	-
7	24885481	24885486	Rps19	+
8	104628178	104628183	Rrad	-
7	78792910	78792915	Mrps11	+
19	7197730	7197735	Macrod1	+
17	6084543	6084548	Gtf2h5	+
2	164830888	164830893	Neurl2	-
7	105557945	105557950	Smpd1	+
11	5897326	5897331	Myl7	-
2	108949469	108949474	Gm13910	+
9	21067639	21067644	Gm49373;Gı	-
3	97702286	97702291	Pde4dip	-
17	24731031	24731036	Rpl3l	+
7	109736833	109736838	Tmem9b	-
6	124714854	124714859	Phb2	+
14	31211387	31211392	Tnnc1	+
8	25023072	25023077	Tm2d2	+
6	71880922	71880927	Ptcd3	-
12	31331857	31331862	Dld	-
3	57287680	57287685	Tm4sf1	-
11	4702173	4702178	Uqcr10	-
17	83501954	83501959	Cox7a2l	-
2	91134825	91134830	Mybpc3	+

10	71228425	71228430	Tfam	-
5	31511242	31511247	Gpn1	+
8	124889352	124889357	Gnpat	+
13	58127253	58127258	Hnrnpa0	-
15	76645936	76645941	Cyhr1	-
6	124740623	124740628	Grcc10;Gm4	-
12	28635434	28635439	Rps7	-
4	147874728	147874733	Mfn2	-
5	121373354	121373359	Trafd1	-
8	85538315	85538320	Dnaja2	-
4	116651425	116651430	Akr1a1	-
19	24876199	24876204	Gm10053	+
2	91135814	91135819	Mybpc3	+
10	128353189	128353194	Cs	+
19	55068067	55068072	Gpam	-
12	8498280	8498285	Rhob	-
1	75216319	75216324	Tuba4a	-
15	96687796	96687801	Slc38a2	-
2	127436314	127436319	Fahd2a	-
16	64766231	64766236	4930453N24	-
4	119278014	119278019	Ybx1	-
11	52245093	52245098	Skp1a	+
7	93179417	93179422	Gm15501	-
4	147874535	147874540	Mfn2	-
9	70003601	70003606	Bnip2	+
2	19394283	19394288	Msrb2	+
8	22578068	22578073	Vdac3	-
16	37836355	37836360	Fstl1	+
1	82747080	82747085	Mff	+
18	80295909	80295914	Kcng2	-
11	70011006	70011011	Acadvl	-
17	27122791	27122796	Uqcc2	-
15	58939314	58939319	Ndufb9	+
17	46251890	46251895	Yipf3	+
14	54944726	54944731	Myh6	-
8	124883356	124883361	Gnpat	+
8	85327247	85327252	Mylk3	-
11	69968436	69968441	Elp5	-
1	43141868	43141873	Fhl2	-
9	122176061	122176066	Ano10	-
18	64462195	64462200	Fech	-
1	171238363	171238368	Ndufs2	-
12	31331759	31331764	Dld	-
6	54985672	54985677	Ggct	-
2	35303270	35303275	Gsn	+

15	89086270	89086275	Trabd	+
19	3911056	3911061	Ndufs8	-
9	54596058	54596063	Idh3a	+
11	93949942	93949947	Nme2;Gm20	-
10	128560140	128560145	Pa2g4	-
16	90226201	90226206	Sod1	+
10	13515016	13515021	Fuca2	+
12	8498096	8498101	Rhob	-
1	58406365	58406370	Bzw1	+
15	55534294	55534299	Mrpl13	-
1	120116394	120116399	Dbi	-
9	22185726	22185731	Rpl15-ps3	-
5	30122689	30122694	Hadha	-
9	50756414	50756419	Cryab	+
7	66049815	66049820	Pcsk6;Gm45	+
1	180179126	180179131	Coq8a	-
13	113663121	113663126	Hspb3	-
3	152237258	152237263	Nexn	-
9	54604548	54604553	Idh3a	+
2	155817919	155817924	BC029722	-
5	5782205	5782210	Gm15459	-
5	138178713	138178718	Taf6	-
2	26347120	26347125	Gpsm1	+
7	102110922	102110927	Art1	+
15	81913176	81913181	Aco2	+
2	13582501	13582506	Vim	+
3	131242572	131242577	Hadh	-
8	95877839	95877844	Got2	-
3	96561477	96561482	Txnip	+
17	39846540	39846545	Tns1	+
5	112689015	112689020	Myo18b	-
5	100799606	100799611	Mrps18c;Gm	+
11	109669284	109669289	Prkar1a	+
6	112459855	112459860	Cav3	+
3	32568294	32568299	Mfn1	+
18	36667849	36667854	Sra1	-
6	51462849	51462854	Hnrnpa2b1	-
11	54909811	54909816	Gpx3	+
10	79711517	79711522	Bsg	+
17	83504165	83504170	Cox7a2l	-
2	151560810	151560815	Fkbp1a	+
5	124493595	124493600	Rilpl1	-
15	79151399	79151404	Polr2f	+
6	124935380	124935385	Mlf2	+
1	24614484	24614489	Gm10925	-

6	124811205	124811210	Tpi1	-
2	167608246	167608251	Ube2v1;Gm2	-
11	119293667	119293672	Eif4a3	-
11	51985995	51986000	Ube2b;Gm26	-
9	54603462	54603467	Idh3a	+
1	106771544	106771549	Vps4b	-
4	136895989	136895994	C1qa	-
11	69667704	69667709	Eif4a1	-
17	8145568	8145573	Gm49673;Rr	-
17	45698401	45698406	Mrpl14	+
19	29020955	29020960	Ak3	-
11	70011379	70011384	Acadvl	-
2	30403562	30403567	Crat	-
14	55897429	55897434	Sdr39u1	-
17	24736036	24736041	Rpl3l	+
18	34345446	34345451	Reep5	-
8	4523788	4523793	Cers4	+
2	181149897	181149902	Eef1a2	-
1	24612920	24612925	Gm28438	-
9	40804634	40804639	Hspa8	+
5	115561391	115561396	Rplp0	+
5	122466847	122466852	Atp2a2	-
7	121076333	121076338	Mettl9	+
19	40292549	40292554	Pdlim1	-
15	77927717	77927722	Txn2	-
13	44931036	44931041	Dtnbp1	-
7	43457739	43457744	Etfb;Gm4523	+
18	74806031	74806036	Acaa2	+
11	69089186	69089191	Vamp2	+
7	15949244	15949249	Ehd2	-
5	30168544	30168549	Hadhb	+
9	116040907	116040912	Gm9385	-
5	135790245	135790250	Mdh2	+
11	67811656	67811661	Dhrs7c	+
6	6560414	6560419	Sem1	-
14	66084743	66084748	Ephx2	-
10	62497838	62497843	Srgn	-
5	30624296	30624301	Kcnk3	+
7	46855344	46855349	Ldha	+
1	175606170	175606175	Fh1	-
7	30554652	30554657	Hspb6	+
1	37897822	37897827	Mrpl30	+
7	48839551	48839556	Csrp3	-
10	62455610	62455615	Vps26a	-
7	105742698	105742703	Ilk	+

5	52154185	52154190	Dhx15	-
2	150831461	150831466	Pygb	+
15	88865069	88865074	Pim3	+
18	77781072	77781077	Atp5a1	+
8	104639888	104639893	Ciao2b	-
13	45532363	45532368	Gmpr	+
13	119335671	119335676	Nnt;Nnt	-
17	26838960	26838965	Nkx2-5	-
5	125388012	125388017	Ubc	-
15	102635271	102635276	Rpl39-ps	+
15	81889416	81889421	Aco2	+
6	83109716	83109721	Gm42688;Mr	+
11	59876460	59876465	Nt5m	+
18	77778887	77778892	Atp5a1	+
3	101581151	101581156	Atp1a1	-
8	70513527	70513532	Kxd1	-
7	128011010	128011015	Trim72	+
10	95480953	95480958	Mrpl42	-
9	121767909	121767914	Zfp651	+
1	190118373	190118378	Prox1	-
18	35713284	35713289	Ecscr	-
10	128920639	128920644	Rdh5;Bloc1s	-
9	20663374	20663379	Pin1	+
1	75362617	75362622	Des	+
3	36464035	36464040	Anxa5	-
5	145147922	145147927	Bud31	+
4	130312393	130312398	Fabp3	+
2	35295029	35295034	Gsn	+
3	142303958	142303963	Pdlim5	-
2	32707813	32707818	Cdk9	-
5	115284599	115284604	Coq5	+
11	75705431	75705436	Crk	+
8	72222612	72222617	Fam32a	+
7	30185335	30185340	Cox7a1	+
17	24639811	24639816	Slc9a3r2	-
4	15919956	15919961	Decr1	-
10	128363466	128363471	Coq10a	-
3	152186602	152186607	Dnajb4	-
1	66838389	66838394	Acadl	-
12	105040431	105040436	Glrx5	+
10	60294639	60294644	Psap	+
2	25560576	25560581	Edf1	+
19	4194804	4194809	Ppp1ca	+
6	113366699	113366704	Tada3	-
16	31947884	31947889	0610012G03	-

5	135012047	135012052	Abhd11	+
7	30554913	30554918	Hspb6	+
7	97407767	97407772	Ndufc2	+
11	116537865	116537870	Ube2o	-
7	3620154	3620159	Ndufa3	+
11	94334662	94334667	Ankrd40	+
17	24426901	24426906	Eci1	+
11	120346382	120346387	Actg1	-
12	54860892	54860897	Cfl2	-
7	30564568	30564573	U2af1l4;Gm4	+
8	124908893	124908898	Egln1	-
8	72586675	72586680	Tmem38a	+
14	61215217	61215222	Sacs	+
2	152737102	152737107	Id1	+
11	116079063	116079068	Wbp2	-
3	135465538	135465543	Ube2d3	+
12	36092062	36092067	Bzw2	-
3	107898206	107898211	Gstm5	+
19	43500215	43500220	Got1	-
1	75363538	75363543	Des	+
1	66831430	66831435	Acadl	-
3	133370444	133370449	Ppa2	+
2	34775800	34775805	Hspa5	+
2	22587412	22587417	Gm13341	-
1	33746367	33746372	Bag2	-
5	115345788	115345793	Cox6a1	-
13	21501129	21501134	Gm11273	-
14	54953441	54953446	Myh6	-
9	120130335	120130340	Rpsa	+
2	174345553	174345558	Gnas	+
19	8834053	8834058	Hnrnpul2;AC	+
15	80968949	80968954	Adsl	+
11	53261720	53261725	Hspa4	-
1	75240919	75240924	Dnajb2	+
9	50644032	50644037	Dlat	-
9	56937970	56937975	Imp3	+
4	126149660	126149665	Eva1b	+
5	115801642	115801647	Rab35	-
6	50564868	50564873	Cycs	-
14	29974505	29974510	Selenok	+
3	90062303	90062308	4933434E20I	+
7	23947034	23947039	Gm10175	-
13	18031535	18031540	Vdac3-ps1	-
10	17827864	17827869	Txlnb	+
2	150618620	150618625	Acss1	-

4	137328812	137328817	Cdc42	-
19	46531205	46531210	Arl3	-
3	107985667	107985672	Gstm2	-
17	24733992	24733997	Rpl3l	+
11	98380867	98380872	Stard3	+
10	86732037	86732042	Fabp3-ps1	-
14	101934631	101934636	Lmo7	+
6	115955275	115955280	Plxnd1	-
4	149150314	149150319	Pgd	-
14	32180396	32180401	Timm23	-
10	128086159	128086164	Atp5b	+
11	84964802	84964807	Car4	+
5	137061666	137061671	Serpine1	-
17	24896086	24896091	Mrps34	+
6	121223389	121223394	Tuba8	+
13	62836945	62836950	Fbp2	-
7	102107252	102107257	Art1	+
5	72978201	72978206	Slain2	+
8	83573413	83573418	Tecr	-
19	4006249	4006254	Doc2g	+
7	79736116	79736121	Pex11a	-
6	66875652	66875657	Gm9794	-
4	136145112	136145117	Id3	+
11	76220967	76220972	Glod4	-
4	133965170	133965175	Hmgn2	-
18	64457615	64457620	Fech	-
4	42980409	42980414	Vcp	-
13	52887265	52887270	Auh	-
2	148872852	148872857	Cst3	-
10	21999400	21999405	Sgk1	+
13	59755910	59755915	Isca1	-
2	156312200	156312205	Scand1	-
9	110966477	110966482	Lrrc2	+
15	11990969	11990974	Sub1	-
9	50753380	50753385	Cryab	+
8	72320987	72320992	Klf2	+
2	122809415	122809420	Sqor	+
11	22899909	22899914	Gm28048;Cc	-
4	49585559	49585564	Tmem246	-
6	99877883	99877888	Tpt1-ps3	-
19	5843563	5843568	Neat1	-
12	78843209	78843214	Atp6v1d	-
10	71330873	71330878	Cisd1	-
8	104628183	104628188	Rrad	-
19	24875984	24875989	Gm10053	+

17	24164726	24164731	Atp6v0c	-
2	150831035	150831040	Pygb	+
7	114705228	114705233	Gm15500	-
2	91264365	91264370	Pacsin3	+
17	29041698	29041703	Srsf3	+
11	97206593	97206598	Npepps	-
14	73593710	73593715	Sucla2	+
6	142565892	142565897	Kcnj8	-
2	74876429	74876434	Mtx2	+
9	107651733	107651738	Slc38a3	-
10	88474853	88474858	Chpt1	-
11	30878479	30878484	Psme4	+
10	13515367	13515372	Fuca2	+
16	92392163	92392168	Rcan1	-
4	3834997	3835002	Rps20	-
5	22612649	22612654	Rpl17-ps5	-
8	22569409	22569414	Slc20a2	+
10	128492349	128492354	Myl6	-
14	55478852	55478857	Dhrs4	+
2	30402108	30402113	Crat	-
6	97232660	97232665	Arl6ip5	+
3	32744872	32744877	Ndufb5	+
1	23996211	23996216	Sdhaf4	-
9	56136345	56136350	Tspan3	-
17	35956983	35956988	Abcf1	-
2	35307670	35307675	Gsn	+
2	145908610	145908615	Naa20	+
7	27188897	27188902	Snrpa	-
10	117814594	117814599	Rap1b	-
1	171241128	171241133	Ndufs2	-
10	40288914	40288919	Amd1	-
15	41866253	41866258	Abra	-
5	140758662	140758667	Gna12	-
17	12915719	12915724	Mrpl18	-
7	128546861	128546866	Bag3	+
19	36115474	36115479	Ankrd1	-
5	30865934	30865939	Mapre3	+
17	70994746	70994751	Myl12a;Myl1:	-
11	93949878	93949883	Nme2;Gm20	-
9	54595260	54595265	Idh3a	+
12	78885247	78885252	Eif2s1	+
1	134431240	134431245	Adipor1	+
14	34567500	34567505	Ldb3	-
1	75367683	75367688	Des	+
16	14192794	14192799	Nde1	+

13	46648587	46648592	Cap2	+
18	43476320	43476325	Eif3j2	-
5	31622578	31622583	Mrpl33;Gm4:	+
12	10394758	10394763	Rdh14	+
14	54944469	54944474	Myh6	-
19	5843686	5843691	Neat1	-
11	70646101	70646106	Slc25a11	-
6	35261554	35261559	1810058I24F	+
2	35307726	35307731	Gsn	+
13	74328355	74328360	Sdha	-
4	107200846	107200851	Tmem59	+
19	9116615	9116620	Asrgl1	-
16	18301015	18301020	Tango2	-
19	9985032	9985037	Fth1	+
5	53278155	53278160	Smim20	+
4	144927211	144927216	Dhrs3	+
16	18316696	18316701	Tango2	-
7	80265307	80265312	Ngrn	+
3	131245212	131245217	Hadh	-
5	77087205	77087210	Hopx	-
5	30176967	30176972	Hadhb	+
9	96895966	96895971	Gm10123	+
13	64363893	64363898	Ctsl	-
14	65980918	65980923	Clu	+
2	155389096	155389101	Trp53inp2	+
8	33863023	33863028	Rbpms	-
16	33948946	33948951	Itgb5	+
1	169694303	169694308	Rgs5	+
12	36015593	36015598	Tspan13	-
1	51301907	51301912	Cavin2	+
14	34577085	34577090	Ldb3	-
16	33948745	33948750	Itgb5	+
17	46128509	46128514	Mrps18a	+
11	100321024	100321029	Eif1	+
7	43456581	43456586	Etfb;Gm4523	+
7	112906712	112906717	Tead1	+
17	47792047	47792052	Tfeb	+
19	6986389	6986394	Vegfb	-
8	33834361	33834366	Rbpms	-
2	155389257	155389262	Trp53inp2	+
12	8499166	8499171	Rhob	-
7	142380766	142380771	Gm49369;Ct	-
7	143514552	143514557	Nap1l4	-
7	111072637	111072642	Eif4g2	-
11	17266954	17266959	C1d	+

10	53345122	53345127	Pln	+
4	119282378	119282383	Ybx1	-
14	51908638	51908643	Ndrg2	-
18	9995637	9995642	Usp14	-
6	24518742	24518747	Ndufa5	-
15	51842601	51842606	Eif3h	-
3	10016278	10016283	Fabp5	+
9	75052248	75052253	Arpp19	+
4	107907394	107907399	Cpt2	-
19	5730482	5730487	Sssca1;Sssc	-
11	106262618	106262623	Psmc5	+
1	36699350	36699355	Actr1b	-
19	40292500	40292505	Pdlim1	-
1	151364029	151364034	Ivns1abp	+
1	93408478	93408483	Hdlbp	-
11	76247376	76247381	Mrm3	+
9	54596065	54596070	Idh3a	+
10	127190529	127190534	Dtx3	-
17	24896132	24896137	Mrps34	+
19	43768338	43768343	Cutc	+
1	118609202	118609207	Clasp1	+
9	37223091	37223096	Tmem218	+
2	130177036	130177041	Snrpb	-
5	5781856	5781861	Gm15459	-
11	109669349	109669354	Prkar1a	+
3	51408194	51408199	Ndufc1	-
1	75216209	75216214	Tuba4a	-
3	86139083	86139088	Rps3a1	-
3	142302752	142302757	Pdlim5	-
17	6436244	6436249	Dynlt1b;Gm2	+
7	19152601	19152606	Snrpd2	+
9	40803914	40803919	Hspa8	+
11	70729370	70729375	Kif1c	+
10	60302537	60302542	Psap	+
10	81123727	81123732	Map2k2	+
7	3298918	3298923	Myadm;Prkc	+
9	103353125	103353130	Cdv3	-
3	90061693	90061698	4933434E20I	+
10	80340063	80340068	Adamtsl5	-
4	123350220	123350225	Macf1	-
6	5483598	5483603	Pdk4	-
5	30624410	30624415	Kcnk3	+
6	86525075	86525080	Pcbp1	-
11	120347150	120347155	Actg1	-
5	33247771	33247776	Ctbp1	-

3	101576279	101576284	Atp1a1	-
6	73263998	73264003	Suclg1	+
5	148314937	148314942	Mtus2	+
8	11514605	11514610	Cars2	-
4	141425233	141425238	Hspb7	+
1	37417240	37417245	Coa5	-
4	150621845	150621850	Rere	+
3	101576564	101576569	Atp1a1	-
14	54942408	54942413	Myh6	-
8	13245484	13245489	Adprhl1	-
2	93695759	93695764	Ext2	-
6	113616385	113616390	Brk1	+
5	97885766	97885771	Antxr2	-
15	76622249	76622254	Vps28	-
11	73176966	73176971	Emc6	-
7	120637695	120637700	Uqcrc2	+
18	65981080	65981085	Lman1	-
2	156311892	156311897	Scand1	-
9	50754525	50754530	Cryab	+
6	99877863	99877868	Tpt1-ps3	-
13	54551895	54551900	4833439L19I	-
2	155389712	155389717	Trp53inp2	+
14	51906014	51906019	Ndrg2	-
4	133266635	133266640	Tmem222	-
11	120543312	120543317	Mcrip1	-
2	32634934	32634939	Ak1	+
1	171129520	171129525	Sdhc	-
1	169694994	169694999	Rgs5	+
9	78478969	78478974	Eef1a1	-
9	71479196	71479201	Polr2m	-
9	50751551	50751556	Hspb2	-
11	59012605	59012610	Obscn	-
4	116067338	116067343	Gm12854	+
9	50753465	50753470	Cryab	+
11	32284052	32284057	Hba-a1	+
8	3622406	3622411	Pet100	+
14	26914189	26914194	Asb14	+
12	55489600	55489605	Nfkbia	-
2	130279324	130279329	Idh3b	-
10	86731964	86731969	Hsp90b1	-
2	35295014	35295019	Gsn	+
2	132311629	132311634	Cds2	+
10	79711259	79711264	Bsg	+
3	108012693	108012698	Gstm1	-
3	60629030	60629035	Mbnl1	+

1	52920547	52920552	Hibch	+
11	5704529	5704534	Mrps24	-
11	120488647	120488652	Mrpl12	+
13	91865350	91865355	Ckmt2	-
11	107495721	107495726	Psmd12	+
10	80392757	80392762	Mbd3	-
15	96687419	96687424	Slc38a2	-
19	10905066	10905071	Prpf19	+
4	57370981	57370986	Gm12537	-
11	102436673	102436678	Grn	+
2	180085505	180085510	Mtg2	+
8	71369179	71369184	Use1	+
5	137529449	137529454	Gnb2;Epo	-
7	143066713	143066718	Cd81	+
17	34200741	34200746	Psmb8	+
7	141191055	141191060	Hras	-
13	86044857	86044862	Cox7c	-
4	148001239	148001244	Nppa	+
7	126980367	126980372	Cdipt	+
5	23839806	23839811	Tomm7	-
5	30174821	30174826	Hadhb	+
9	55486730	55486735	Etfa	-
15	103244414	103244419	Hnrnpa1	+
18	80211677	80211682	Gm16286;Tx	+
2	19394830	19394835	Msrb2	+
8	26000979	26000984	Fnta	-
6	32792794	32792799	Chchd3	-
8	95864414	95864419	Got2	-
14	69717192	69717197	Chmp7	-
19	3911066	3911071	Ndufs8	-
19	5426794	5426799	AI837181	+
18	61259205	61259210	Rps2-ps10	-
14	20500068	20500073	Ppp3cb	-
1	171241049	171241054	Ndufs2	-
7	25630023	25630028	Bckdha	-
17	35267098	35267103	H2-D1	+
18	35616998	35617003	Paip2	+
2	26593332	26593337	Agpat2	-
2	121450005	121450010	Serf2	+
19	43507347	43507352	Got1	-
2	91136453	91136458	Mybpc3	+
8	33783775	33783780	Rbpms	-
9	40804334	40804339	Hspa8	+
11	31549527	31549532	Ncoa2	+
18	60812525	60812530	Cd74	+

8	57324103	57324108	Hand2	+
8	83566868	83566873	Ndufb7	+
19	6398076	6398081	Pygm	+
14	30999839	30999844	Spcs1	-
16	30602222	30602227	Fam43a	+
11	115410804	115410809	Mrpl58	+
8	8681412	8681417	Arglu1	-
3	86141298	86141303	Rps3a1	-
14	60903431	60903436	Mipep	+
2	127298523	127298528	Stard7	+
1	118608824	118608829	Clasp1	+
3	10205995	10206000	Fabp4	-
18	36742548	36742553	Ndufa2	-
5	20758799	20758804	Phtf2	-
1	53344654	53344659	Gm28551;As	-
11	97777806	97777811	Rpl23	-
5	121445301	121445306	Erp29	-
2	25222972	25222977	Tubb4b	-
10	79675726	79675731	Tpgs1	+
16	92392181	92392186	Rcan1	-
18	35590516	35590521	Matr3	+
4	119278264	119278269	Ybx1	-
12	8285105	8285110	Ldah	+
16	4764877	4764882	Hmox2	+
15	77927825	77927830	Txn2	-
1	84724712	84724717	Trip12	-
14	54429693	54429698	Mrpl52	+
10	62497806	62497811	Srgn	-
6	121225913	121225918	Tuba8	+
2	156123924	156123929	Nfs1;Gm280	-
1	72633029	72633034	Smarcal1	+
19	10905101	10905106	Prpf19	+
19	6995453	6995458	Nudt22	-
7	45127211	45127216	Rpl13a;Gm4	-
1	156366476	156366481	Gm2000	+
11	69554670	69554675	Efnb3	-
4	10848867	10848872	Gm12918	+
4	132855008	132855013	Stx12	-
11	58392766	58392771	Gm12251	-
11	93959409	93959414	Gm20390;Nr	-
2	144250998	144251003	Snx5	-
4	10848712	10848717	Gm12918	+
14	60780380	60780385	C1qtnf9	+
7	126797494	126797499	Aldoa	-
7	30623512	30623517	Cox6b1	-

16	20283007	20283012	Parl	-
9	106889049	106889054	Manf	-
6	83109135	83109140	Gm42688;Mı	+
2	125831948	125831953	Cops2	-
2	26591667	26591672	Egfl7;Gm205	+
7	127257178	127257183	Dctpp1	-
8	128722581	128722586	Itgb1	+
1	66838342	66838347	Acadl	-
11	100362028	100362033	Gm10039	-
8	128505388	128505393	Pard3	+
2	174465344	174465349	Prelid3b	-
16	20722070	20722075	Polr2h	+
17	27562703	27562708	Hmga1	+
18	60778501	60778506	Rps14	+
5	24581676	24581681	Gm10221	-
10	81118165	81118170	Map2k2	+
12	69183106	69183111	Rpl36al;Gm4	-
11	62552432	62552437	Ubb	+
7	126796251	126796256	Aldoa	-
5	77351954	77351959	Igfbp7	-
8	104629826	104629831	Rrad	-
5	115561358	115561363	Rplp0	+
3	85915858	85915863	Gm9790	+
7	59985980	59985985	Gm38393;Sr	-
13	63302832	63302837	2010111I01F	+
8	84842338	84842343	Calr	-
2	145903310	145903315	Naa20	+
7	46855058	46855063	Ldha	+
4	138206934	138206939	Eif4g3	+
11	40748616	40748621	Ccng1	-
8	13165935	13165940	Lamp1	+
4	144919838	144919843	Dhrs3	+
13	34986094	34986099	Eci2	-
2	10063582	10063587	Atp5c1	-
5	121576017	121576022	Aldh2	-
19	4001909	4001914	Nudt8;Gm49	+
15	83376613	83376618	Pacsin2	-
1	36534099	36534104	Ankrd23;Gm	-
15	75895890	75895895	Eef1d	-
8	123403428	123403433	Tcf25;Gm20	+
8	85080211	85080216	Wdr83	-
11	55394774	55394779	Sparc	-
7	126780263	126780268	Ypel3	+
14	63142682	63142687	Ctsb	+
6	48691969	48691974	Gimap4	+

6	121223229	121223234	Tuba8	+
1	75181738	75181743	Gm29253;At(	-
9	100495455	100495460	Nck1	-
15	76904652	76904657	Rpl8	+
13	34990350	34990355	Eci2	-
10	57516372	57516377	Serinc1	-
2	120089640	120089645	Ehd4	-
13	91865343	91865348	Ckmt2	-
18	34941904	34941909	Hspa9	-
5	17782854	17782859	Cd36	-
13	49623816	49623821	Ogn	+
7	59985154	59985159	Gm38393;Sr	-
5	17814773	17814778	Cd36	-
3	152186503	152186508	Dnajb4	-
8	72152704	72152709	Tpm4	+
15	43266138	43266143	Eif3e	-
15	73093893	73093898	Chrac1	+
11	5898450	5898455	Myl7	-
7	114706042	114706047	Gm15500	-
4	46115841	46115846	Tmod1	+
5	117092843	117092848	Suds3	-
5	122100991	122100996	Myl2	+
3	96528209	96528214	Hfe2	+
19	4002074	4002079	Nudt8;Gm49	+
3	105967617	105967622	Wdr77;Gm42	+
15	41866209	41866214	Abra	-
17	66667479	66667484	Ptprm	-
5	122453824	122453829	Atp2a2	-
1	74396153	74396158	Ctdsp1	+
11	120346743	120346748	Actg1;Gm23	-
2	34776410	34776415	Hspa5	+
12	85133095	85133100	Dlst	+
5	115801784	115801789	Gm13841	-
19	6982520	6982525	Vegfb	-
16	4481545	4481550	Srl	-
10	86731797	86731802	Hsp90b1	-
17	29137113	29137118	Rpl35a-ps3	-
9	40804652	40804657	Hspa8	+
4	99964679	99964684	Pgm1	+
15	75045458	75045463	Ly6c1	-
5	65390407	65390412	Rpl9	-
6	4540601	4540606	Col1a2	+
1	161244218	161244223	Prdx6	-
2	84773466	84773471	Serping1	-
7	139578593	139578598	Inpp5a	+

13	23531449	23531454	Hist1h4h	+
15	102331379	102331384	Pfdn5	+
14	77840179	77840184	Dnajc15	-
7	80094927	80094932	Idh2	-
8	34090456	34090461	Dctn6	-
3	135460065	135460070	Ube2d3	+
6	17341004	17341009	Cav1	+
19	4193138	4193143	Ppp1ca	+
5	77087039	77087044	Hopx	-
14	34561855	34561860	Ldb3	-
13	59755426	59755431	Isca1	-
17	26126368	26126373	Mrpl28	+
2	25223950	25223955	Tubb4b	-
4	15919007	15919012	Decr1	-
6	87845278	87845283	Cnbp	-
17	53866655	53866660	Des	-
17	33952545	33952550	Rps18	-
6	121226549	121226554	Tuba8	+
7	44550777	44550782	Nr1h2	-
2	4936098	4936103	Phyh	+
6	131379456	131379461	Ybx3	-
1	169695387	169695392	Rgs5	+
7	28974650	28974655	Eif3k	-
10	58423149	58423154	Lims1	+
17	46168822	46168827	Gtpbp2	+
2	26409870	26409875	Sec16a	-
14	61221313	61221318	Sgcg	-
8	11198824	11198829	Col4a1	-
11	21329913	21329918	Ugp2	-
2	10068728	10068733	Atp5c1	-
6	72369306	72369311	Vamp5	-
10	97517597	97517602	Dcn	+
2	32671472	32671477	Eng	+
9	96471679	96471684	Rnf7	-
6	90645876	90645881	Slc41a3	+
8	19493375	19493380	Rpl19-ps11	+
17	5440208	5440213	Tmem242	-
14	73364683	73364688	Itm2b	-
2	143942618	143942623	Dstn	+
12	110977087	110977092	Ankrd9	-
6	142588401	142588406	Abcc9	-
11	88210990	88210995	Mrps23	+
10	128912472	128912477	Cd63	+
8	95713830	95713835	Ndrg4	+
1	57407139	57407144	Maip1	+

11	115523463	115523468	Sumo2	-
14	8166788	8166793	Pdhb	-
12	8938636	8938641	Laptm4a	+
3	108266465	108266470	Psma5	+
17	24722383	24722388	Ndufb10	-
1	135848006	135848011	Tnnt2	+
9	55486761	55486766	Etfa	-
6	66875534	66875539	Gm9794	-
2	25998592	25998597	Ubac1	-
15	76354083	76354088	Maf1	+
2	156124194	156124199	Nfs1;Gm280	-
6	136634487	136634492	Plbd1	-
7	48835497	48835502	Csrp3	-
2	120090744	120090749	Ehd4	-
10	81179602	81179607	Eef2	+
7	45638857	45638862	Rasip1	+
6	24604425	24604430	Lmod2	+
1	175609687	175609692	Fh1	-
12	105040437	105040442	Glrx5	+
3	105942863	105942868	Atp5f1	-
7	45720364	45720369	Rpl18	+
12	72794108	72794113	Ppm1a	+
13	119707824	119707829	Hmgcs1	+
5	116408887	116408892	Hspb8	-
6	72608454	72608459	Tgoln1	-
11	40680748	40680753	Mat2b	-
7	45461840	45461845	Bax	-
9	108949558	108949563	Uqcrc1	+
8	11449108	11449113	Col4a2	+
19	32466361	32466366	Rpl9-ps6	-
7	45123213	45123218	Rps11	-
4	46106922	46106927	Tmod1	+
8	107046327	107046332	Pdf;Cog8	-
7	19149881	19149886	Snrpd2	+
12	105040374	105040379	Glrx5	+
9	106212340	106212345	Twf2	+
7	46850893	46850898	Ldha	+
14	63142304	63142309	Ctsb	+
9	78209298	78209303	Gsta4	+
17	23674618	23674623	Hcfc1r1	+
1	95498918	95498923	Gm15427	+
19	46331976	46331981	Cuedc2	-
19	36112468	36112473	Ankrd1	-
5	122461900	122461905	Atp2a2	-
2	122747398	122747403	Bloc1s6	+

12	31329450	31329455	Lamb1	+
7	99158303	99158308	Dgat2	-
8	19493590	19493595	Rpl19-ps11	+
17	47791963	47791968	Tfeb	+
11	68972579	68972584	Rangrf	-
18	60776946	60776951	Rps14	+
10	78162139	78162144	D10Jhu81e	-
10	128548968	128548973	Rpl41	-
9	4302543	4302548	Aasdhppt	-
7	46855027	46855032	Ldha	+
1	171219243	171219248	Tomm40l	-
15	3275608	3275613	Selenop	+
12	96922741	96922746	Gm6863	-
10	93861179	93861184	Metap2	-
4	129143642	129143647	Fndc5	+
9	54957807	54957812	Psma4	+
2	174122600	174122605	Npepl1	+
5	21747075	21747080	Pmpcb	+
3	131233449	131233454	Hadh	-
17	46683027	46683032	Mea1;Gm26	+
7	30321684	30321689	Sdhaf1	-
10	75532316	75532321	Snrpd3	+
5	125017686	125017691	Ncor2	-
6	71874904	71874909	Immt	+
11	121327757	121327762	Wdr45b	-
10	13515151	13515156	Fuca2	+
19	8772280	8772285	Tmem223	+
10	117051126	117051131	Cct2	-
13	38198201	38198206	Dsp	+
15	81848646	81848651	Tob2	-
13	74332109	74332114	Sdha	-
1	135301648	135301653	Timm17a	-
6	71214397	71214402	Smyd1	-
4	116691806	116691811	Prdx1	+
11	31545927	31545932	Ncoa2	+
6	87999590	87999595	Rab7	-
14	55581660	55581665	Emc9	-
5	143562938	143562943	Fam220a;Fa	+
2	32233692	32233697	Prrc2b	+
4	109961880	109961885	Faf1	+
2	131938186	131938191	Prnp;Prn	+
12	55412287	55412292	Psma6	+
17	70996747	70996752	Myl12a;Myl1	-
5	129022505	129022510	Ran	+
7	81342827	81342832	Rps17	-

9	66511843	66511848	Fbxl22	-
1	59639791	59639796	Sumo1	-
4	41190910	41190915	Ube2r2	+
9	21008658	21008663	Mrpl4	+
4	119279086	119279091	Ybx1	-
11	40750751	40750756	Ccng1	-
1	156366259	156366264	Gm2000	+
8	104639898	104639903	Ciao2b	-
11	60865022	60865027	Tmem11	-
1	135306192	135306197	Timm17a	-
9	55488925	55488930	Etfa	-
13	73320295	73320300	Ndufs6	-
14	88123483	88123488	Rps3a2	-
9	108945374	108945379	Uqcrc1	+
2	34775900	34775905	Hspa5	+
1	43123377	43123382	Fhl2	-
7	126547006	126547011	Eif3c	-
2	163336411	163336416	Jph2	-
1	66841675	66841680	Acadl	-
8	95714596	95714601	Ndrg4	+
13	115088759	115088764	Pelo;Itga1;Gı	-
9	50634999	50635004	Dlat	-
10	53343949	53343954	Pln	+
2	120090357	120090362	Ehd4	-
18	80295735	80295740	Kcng2	-
4	3973138	3973143	Gm11808	-
11	48806071	48806076	Rack1	+
2	174345507	174345512	Gnas	+
7	126799062	126799067	Aldoa	-
2	155277165	155277170	Map1lc3a	+
16	18069428	18069433	Dgcr6	+
2	76708237	76708242	Ttn	-
17	24742437	24742442	Msrb1	+
1	165650111	165650116	Rcsd1	-
4	141424970	141424975	Hspb7	+
15	74995025	74995030	Ly6a	-
4	24903231	24903236	Ndufaf4	+
3	95662413	95662418	Mcl1	+
19	55067716	55067721	Gpam	-
6	121226367	121226372	Tuba8	+
6	122457116	122457121	Gm8430	+
2	90894836	90894841	Ndufs3	-
6	48833838	48833843	Tmem176b	-
9	50751364	50751369	Hspb2	-
5	5782498	5782503	Gm15459	-

10	79688123	79688128	Cdc34	+
7	125467715	125467720	Nsmce1	-
4	43654239	43654244	Hint2	-
2	146889542	146889547	Kiz	+
19	43499936	43499941	Got1	-
1	151361002	151361007	Ivns1abp	+
19	4007530	4007535	Ndufv1	-
14	34561453	34561458	Ldb3	-
2	151561410	151561415	Fkbp1a	+
19	32465923	32465928	Rpl9-ps6	-
10	29346868	29346873	Rnf146	-
4	135214217	135214222	Clic4	-
17	34284361	34284366	H2-Aa	-
4	137320001	137320006	Cdc42	-
10	61673843	61673848	Ppa1	+
15	36999208	36999213	Zfp706	-
2	32110547	32110552	Plpp7	+
17	45570280	45570285	Hsp90ab1	-
8	15132673	15132678	Myom2	+
4	119279126	119279131	Ybx1	-
4	129593561	129593566	Eif3i	-
13	59756617	59756622	Isca1	-
19	41917999	41918004	Pgam1	+
7	34205880	34205885	Gpi1	-
11	48803916	48803921	Rack1	+
11	102043209	102043214	Cd300lg	+
3	152237321	152237326	Nexn	-
5	129715748	129715753	Mrps17	+
6	117845675	117845680	Zfp637	+
4	141424016	141424021	Hspb7	+
5	129758290	129758295	Nipsnap2	+
8	94395208	94395213	Herpud1	+
17	27579959	27579964	Nudt3	-
5	33076257	33076262	Rpl35a-ps5	+
12	55407497	55407502	Psma6	+
3	10204639	10204644	Fabp4	-
5	100655036	100655041	Coq2	-
1	164824087	164824092	Dpt	+
6	67283956	67283961	Serbp1	+
15	98132089	98132094	Pfkm	+
12	87444011	87444016	Slirp	+
3	144705551	144705556	Sh3glb1	-
1	189277648	189277653	Pdcd5-ps;Kc	-
11	70661410	70661415	Eno3	+
9	90075887	90075892	Ctsh	+

6	113067921	113067926	Thumpd3	+
6	117925315	117925320	Hnrnpf	+
10	128327096	128327101	Cnpy2	+
11	54869861	54869866	Hint1	+
17	35831009	35831014	Flot1	+
19	8977359	8977364	Eef1g	+
5	104085727	104085732	Sparcl1	-
11	98384831	98384836	Tcap	+
17	8134910	8134915	Gm49673;Rr	-
11	75764705	75764710	Ywhae	+
5	121132013	121132018	Ptpn11	-
14	13949318	13949323	Thoc7	-
15	76071248	76071253	Puf60	-
1	9576373	9576378	Adhfe1	+
10	79710704	79710709	Bsg	+
17	23824973	23824978	Elob	-
9	101105623	101105628	Ppp2r3a	-
7	34204041	34204046	Gpi1	-
19	3908940	3908945	Ndufs8	-
8	70757251	70757256	Rab3a	+
5	124515552	124515557	Rilpl1	-
13	11554276	11554281	Ryr2	-
15	81914295	81914300	Aco2	+
2	150618674	150618679	Acss1	-
15	103140221	103140226	Gm49477	+
12	13219317	13219322	Ddx1	-
17	25795277	25795282	AC134908.4;	-
12	65062555	65062560	Fkbp3	-
15	89419902	89419907	Gm44502;Cp	-
1	135301792	135301797	Timm17a	-
15	25971886	25971891	Retreg1	+
7	127848821	127848826	Stx4a	+
12	72794045	72794050	Ppm1a	+
3	94448203	94448208	Mrpl9	+
2	91125990	91125995	Mybpc3	+
1	164438340	164438345	Atp1b1	-
10	94200012	94200017	Ndufa12	+
15	76904899	76904904	Rpl8	+
3	152237478	152237483	Nexn	-
11	21322059	21322064	Ugp2	-
11	58934215	58934220	Rnf187	-
11	40748636	40748641	Ccng1	-
9	108339504	108339509	Gpx1	+
11	115645726	115645731	Grb2	-
1	172506774	172506779	Tagln2	+

2	32963819	32963824	Rpl12	+
13	3556965	3556970	Gdi2	+
10	79820873	79820878	Palm	+
5	5782852	5782857	Gm15459	-
2	35304651	35304656	Gsn	+
6	34304010	34304015	Akr1b3	-
1	156678740	156678745	Fam20b	-
5	104085809	104085814	Sparcl1	-
13	55320208	55320213	Rab24	-
17	24742705	24742710	Msrb1	+
1	45348719	45348724	Col3a1	+
9	118900420	118900425	Itga9	+
7	24885161	24885166	Rps19	+
11	117813586	117813591	Syngr2;Gm2	+
12	8936719	8936724	Laptm4a	+
14	50915767	50915772	Osgep	-
10	117280754	117280759	Lyz2	-
13	12290683	12290688	Actn2	-
6	32792772	32792777	Chchd3	-
2	122151136	122151141	B2m	+
9	31149974	31149979	Aplp2	-
17	46018391	46018396	Vegfa	-
5	17782404	17782409	Cd36	-
8	94853116	94853121	Coq9	+
3	105954177	105954182	Atp5f1	-
8	92853026	92853031	Mmp2	+
7	81768980	81768985	Ramac	+
2	180162452	180162457	Osbpl2	+
9	108945379	108945384	Uqcrc1	+
8	84970333	84970338	Prdx2	+
7	28829878	28829883	Ech1	+
4	139103860	139103865	Minos1	-
11	58994424	58994429	Obscn	-
1	180811867	180811872	H3f3a	-
5	31203082	31203087	Ppm1g	-
4	117154796	117154801	Rps8	-
1	63147182	63147187	Ndufs1	-
8	8666790	8666795	Arglu1	-
13	23704367	23704372	Hfe	-
3	88309178	88309183	Cct3	+
19	4008126	4008131	Ndufv1	-
16	91931529	91931534	Atp5o	-
15	98131694	98131699	Pfkm	+
16	33948803	33948808	Itgb5	+
9	50605129	50605134	Timm8b	+

1	66830942	66830947	Acadl	-
14	51905428	51905433	Ndrg2	-
19	27249702	27249707	Vldlr	+
3	123026214	123026219	Myoz2	-
6	137750748	137750753	Strap	+
5	5781559	5781564	Pcdh7	-
2	153767207	153767212	Mapre1	+
10	128359169	128359174	Cs	+
2	173034078	173034083	Rbm38	+
18	67405017	67405022	Afg3l2	-
2	157522046	157522051	Gm14279	-
7	30186950	30186955	Capns1	-
3	32934269	32934274	Usp13	+
7	30946300	30946305	Usf2	-
10	13515399	13515404	Fuca2	+
8	107412568	107412573	Nob1	-
17	46649933	46649938	Mrpl2;Gm26	+
11	95680524	95680529	Phb	+
11	115645857	115645862	Grb2	-
11	95026288	95026293	Pdk2	-
11	5801806	5801811	Pgam2	-
10	57516928	57516933	Serinc1	-
10	53345584	53345589	Pln	+
10	80258212	80258217	Gamt	-
2	34623424	34623429	Mapkap1	+
7	45720782	45720787	Rpl18	+
14	88122990	88122995	Gm9800	-
12	72792810	72792815	Ppm1a	+
6	50564510	50564515	Cycs	-
19	34248468	34248473	Acta2	-
2	104114564	104114569	Cd59a	+
10	77598356	77598361	Pttg1ip	+
8	102865698	102865703	Gm8730	-
8	104628299	104628304	Rrad	-
11	58315407	58315412	Zfp672	-
10	75937739	75937744	Chchd10	+
10	85941945	85941950	Rtcb	-
4	116699300	116699305	Prdx1	+
8	68906127	68906132	Lpl	+
9	100982103	100982108	Pccb	-
5	125454849	125454854	Bri3bp	+
3	90231717	90231722	Jtb	+
4	141577049	141577054	Fblim1	-
13	21501242	21501247	Gm11273	-
5	95862681	95862686	Gm5559	+

11	58998483	58998488	Obscn	-
4	107904269	107904274	Cpt2	-
7	114267175	114267180	Psma1	-
19	40249694	40249699	Pdlim1	-
2	104115122	104115127	Cd59a	+
11	120457953	120457958	Oxld1	-
19	3908995	3909000	Ndufs8	-
11	58995058	58995063	Obscn	-
5	117282852	117282857	Pebp1	-
12	8922193	8922198	Laptm4a	+
8	106573002	106573007	Gm10073	-
3	142303325	142303330	Pdlim5	-
1	180166760	180166765	Coq8a	-
11	6172919	6172924	Rps15a-ps6;	-
19	60864563	60864568	Prdx3	-
16	4707269	4707274	Dnaja3	+
14	55563761	55563766	Dcaf11	+
1	53347348	53347353	Gm28551;As	-
8	109671933	109671938	Ist1	-
8	85539350	85539355	Dnaja2	-
8	102865033	102865038	Gm8730	-
9	110983237	110983242	Lrrc2	+
11	60952574	60952579	Map2k3	+
7	105480898	105480903	Cavin3	-
5	135785931	135785936	Mdh2	+
12	103325438	103325443	Asb2	-
3	20122361	20122366	Gyg	-
4	147874829	147874834	Mfn2	-
8	24438428	24438433	Tcim	-
10	17845624	17845629	Txlnb	+
17	35253071	35253076	Ddx39b	+
9	108339954	108339959	Gpx1	+
4	43663971	43663976	Hint2	+
10	60302411	60302416	Psap	+
3	37405098	37405103	Nudt6	-
14	46776204	46776209	Cnih1	-
10	62616239	62616244	Ddx50	-
8	57323942	57323947	Hand2	+
4	114961711	114961716	Cmpk1	-
1	120226905	120226910	Steap3	-
9	108944732	108944737	Uqcrc1	+
8	119348187	119348192	Hsbp1	+
4	107903995	107904000	Cpt2	-
3	105942700	105942705	Atp5f1	-
13	74359789	74359794	Lrrc14b	-

4	156218112	156218117	Perm1	+
15	5120729	5120734	Gm10250	-
10	57516620	57516625	Serinc1	-
3	88321516	88321521	Cct3	+
11	94491042	94491047	Epn3	-
13	23692224	23692229	Hist1h2bc	+
6	82725662	82725667	Hk2	-
4	46115826	46115831	Tmod1	+
4	136880199	136880204	C1qb	-
18	77779929	77779934	Atp5a1	+
17	23674046	23674051	Hcfc1r1	+
8	70870035	70870040	Ccdc124	-
17	33709874	33709879	March2	-
8	19493212	19493217	Rpl19-ps11;(	+
5	30119543	30119548	Hadha	-
13	14623652	14623657	Psma2	+
16	24530909	24530914	Lpp	+
10	53344304	53344309	Pln	+
4	108044383	108044388	Podn;Scp2	-
7	111074378	111074383	Eif4g2	-
5	92393136	92393141	Art3	+
10	29698906	29698911	Gm10275	-
2	6189543	6189548	Echdc3	-
9	107614849	107614854	Gnai2	-
2	181153005	181153010	Eef1a2	-
3	138555802	138555807	Eif4e	+
2	150831575	150831580	Pygb	+
4	137568338	137568343	Hspg2	+
4	107200662	107200667	Tmem59	+
6	91272141	91272146	Fbln2	+
17	29327166	29327171	Pi16	+
5	114250538	114250543	Acacb	+
3	131242542	131242547	Hadh	-
2	76706845	76706850	Ttn	-
9	107301884	107301889	Cish	+
4	119278256	119278261	Ybx1	-
2	4938688	4938693	Phyh	+
8	72151790	72151795	Tpm4	+
11	31665710	31665715	Bod1	-
7	30555053	30555058	Hspb6	+
5	134238095	134238100	Gtf2i	-
5	73633855	73633860	Sgcb	-
2	122147780	122147785	B2m	+
7	99482305	99482310	Rps3	-
5	140443026	140443031	Eif3b	+

16	36035671	36035676	Kpna1	+
6	67284068	67284073	Serbp1	+
7	102110999	102111004	Art1	+
4	108044789	108044794	Podn;Scp2	-
9	22072405	22072410	Ecsit	-
8	94854510	94854515	Coq9	+
8	94854475	94854480	Coq9	+
13	75645100	75645105	Mir682;Gm4	+
17	25831605	25831610	Stub1	-
19	8978100	8978105	Eef1g	+
16	4040035	4040040	Trap1	-
11	6349427	6349432	Ogdh	+
9	108303148	108303153	Tcta	-
5	148504705	148504710	Ubl3	-
11	115415845	115415850	Atp5h	-
11	68821610	68821615	Ndel1	-
16	4706845	4706850	Dnaja3	+
12	110898214	110898219	Rps19-ps6;T	+
9	53582125	53582130	Acat1	-
4	149725139	149725144	Tmem201	-
1	118300686	118300691	Tsn	-
9	65329894	65329899	Clpx	+
1	55078343	55078348	Hspd1	-
6	24604638	24604643	Lmod2	+
19	29021967	29021972	Ak3	-
2	148872833	148872838	Cst3	-
7	80095811	80095816	Idh2	-
14	37121678	37121683	Ghitm	-
17	15490252	15490257	Psmb1	-
10	86732163	86732168	Fabp3-ps1	-
9	108205008	108205013	Dag1	-
1	37898159	37898164	Mrpl30	+
1	52159655	52159660	Stat1	+
10	13507376	13507381	Fuca2	+
11	96861100	96861105	Copz2	+
11	102196640	102196645	Hdac5	-
9	22113472	22113477	Elof1	-
1	95498847	95498852	Gm15427	+
7	51747246	51747251	Gm7336	+
16	87479568	87479573	Usp16	+
16	29228346	29228351	Hrasls	+
8	85355322	85355327	Mylk3	-
19	27253831	27253836	Vldlr	+
15	82348034	82348039	Smdt1	+
14	34561472	34561477	Ldb3	-

8	124839782	124839787	2810004N23	-
5	129128163	129128168	Rps16-ps2	-
8	94854391	94854396	Coq9	+
14	32189213	32189218	Timm23	-
2	91136111	91136116	Mybpc3	+
2	121457662	121457667	Serf2;Hypk	+
11	104584550	104584555	Myl4	+
9	79769249	79769254	Tmem30a	-
9	40804967	40804972	Hspa8	+
7	45095162	45095167	Fcgrt	-
2	18675940	18675945	Commd3	+
6	142494139	142494144	Ldhb	-
17	70994440	70994445	Myl12a;Myl1:	-
4	141424386	141424391	Hspb7	+
11	62552617	62552622	Ubb	+
17	71857833	71857838	Clip4	+
7	46850274	46850279	Ldha	+
14	25694393	25694398	Ppif	+
11	55500147	55500152	G3bp1	+
14	47736209	47736214	Ktn1	+
17	8297535	8297540	Mpc1	+
5	122469703	122469708	Atp2a2	-
14	32096594	32096599	Oxnad1	+
2	38588088	38588093	Psmb7	-
17	56259753	56259758	Fem1a	+
1	120113504	120113509	Dbi	-
12	70984578	70984583	Psma3	+
9	50344521	50344526	Rpl10-ps3	-
6	119925482	119925487	Wnk1	-
9	69491617	69491622	Anxa2	+
11	96817993	96817998	Nfe2l1	-
10	77269020	77269025	Pofut2	+
6	37806943	37806948	Atp6v0c-ps2	+
4	117272015	117272020	Rnf220	-
5	97885459	97885464	Antxr2	-
8	70699827	70699832	Jund	+
15	58784767	58784772	Tmem65	-
7	80095805	80095810	Idh2	-
4	129143674	129143679	Fndc5	+
13	108052683	108052688	Ndufaf2	-
16	96228629	96228634	Sh3bgr	+
9	22185569	22185574	Pigyl	-
12	79156471	79156476	Vti1b	-
8	107045118	107045123	Vps4a	+
17	78920218	78920223	Cebpzos	+

2	181359394	181359399	Arfrp1	-
6	99878120	99878125	Tpt1-ps3	-
19	9113153	9113158	Asrgl1	-
12	111827890	111827895	Zfyve21	+
17	35267204	35267209	H2-D1	+
10	76596328	76596333	Col6a2	-
2	174346019	174346024	Gnas	+
15	7207441	7207446	Egflam	-
6	124705539	124705544	Emg1	-
11	119288473	119288478	Eif4a3	-
11	106683607	106683612	Pecam1	-
6	66875528	66875533	Gm9794	-
2	32634736	32634741	Ak1	+
12	111785496	111785501	Klc1	+
11	40750043	40750048	Ccng1	-
8	95865212	95865217	Got2	-
7	139659651	139659656	Cfap46	-
11	40750084	40750089	Ccng1	-
15	81912330	81912335	Aco2	+
10	128361304	128361309	Cs	+
2	180257992	180257997	Rps21	+
14	21448054	21448059	Adk	+
7	99157018	99157023	Dgat2	-
1	135845595	135845600	Tnnt2	+
15	89155878	89155883	Plxnb2	-
11	95680362	95680367	Phb	+
11	59012392	59012397	Obscn	-
14	120943876	120943881	Ipo5	+
11	120347130	120347135	Actg1	-
1	75217314	75217319	Tuba4a	-
7	28972555	28972560	Eif3k	-
14	8166347	8166352	Pdhb	-
4	3938990	3938995	Chchd7	+
16	90341603	90341608	Gm49708	-
5	129846299	129846304	Cct6a	+
2	122265182	122265187	Sord	+
4	15922487	15922492	Decr1	-
4	150897351	150897356	Park7	-
17	35053119	35053124	Clic1	+
11	55394981	55394986	Sparc	-
14	51910782	51910787	Ndrg2	-
6	148181526	148181531	Ergic2	-
12	31332030	31332035	Dld	-
2	143943083	143943088	Dstn	+
17	35895848	35895853	2310061I04F	-

2	36044356	36044361	Ndufa8	-
17	5411183	5411188	Tmem242	-
1	164823856	164823861	Dpt	+
2	34774369	34774374	Hspa5	+
6	124810725	124810730	Tpi1	-
10	43524542	43524547	Gm9803;Gm	+
15	89055062	89055067	Mov10l1	+
11	69397559	69397564	Tmem88	-
4	152327536	152327541	Rpl22	+
1	74280066	74280071	Aamp	-
3	67474847	67474852	Gfm1	+
11	62525012	62525017	Cenpv	-
1	43196663	43196668	Fhl2	-
15	43527620	43527625	Emc2	+
6	29461571	29461576	Flnc	+
3	123034938	123034943	Myoz2	-
7	73776044	73776049	Fam174b	+
3	150072846	150072851	Rpsa-ps10	-
1	92439700	92439705	Ndufa10	-
18	75009379	75009384	BC031181	+
13	58394135	58394140	Hnrnpk	-
4	107200498	107200503	Tmem59	+
1	172273040	172273045	Atp1a2	-
10	127517591	127517596	Shmt2	-
7	122096633	122096638	Ndufab1	-
7	73776474	73776479	Fam174b	+
18	70625852	70625857	Mbd2	+
1	165481060	165481065	Mpc2	+
15	100621885	100621890	Smagp	-
15	55534334	55534339	Mrpl13	-
9	123307547	123307552	Scp2-ps2	-
7	102111009	102111014	Art1	+
12	80950238	80950243	Srsf5	+
7	138891935	138891940	Bnip3	-
8	92850218	92850223	Mmp2	+
1	131055019	131055024	Mapkapk2	-
1	86530456	86530461	Ptma	+
19	5842660	5842665	Neat1	-
5	124345961	124345966	Cdk2ap1	-
17	33996449	33996454	H2-K1	-
4	49586125	49586130	Tmem246	-
10	18011320	18011325	Abracl	-
4	136890576	136890581	C1qc	-
19	4000711	4000716	Nudt8;Gm49	+
9	44686604	44686609	Phldb1	-

2	84809948	84809953	Ube2l6	+
4	137320100	137320105	Cdc42	-
2	121548581	121548586	Frmd5	-
3	107983952	107983957	Gstm2	-
7	19093706	19093711	Dmpk	+
8	124889654	124889659	Gnpat	+
1	66831173	66831178	Acadl	-
17	56607753	56607758	2410015M20	-
9	54599605	54599610	Idh3a	+
11	52122327	52122332	Ppp2ca	+
3	102145747	102145752	Casq2	+
5	104065084	104065089	Nudt9	+
13	30540529	30540534	Uqcrfs1	-
5	115295922	115295927	Coq5	+
15	80110640	80110645	Syngr1	+
2	101562191	101562196	B230118H07	-
5	69516697	69516702	Yipf7	-
7	126490657	126490662	Tufm	+
13	98316266	98316271	Btf3	-
2	76705264	76705269	Ttn	-
15	100620612	100620617	Dazap2	+
2	76705144	76705149	Ttn	-
1	156366384	156366389	Gm2000	+
4	8561209	8561214	Rab2a	+
10	128548184	128548189	Rpl41	-
2	32963573	32963578	Rpl12	+
11	6350643	6350648	Ogdh	+
9	116040847	116040852	Gm9385	-
14	60779907	60779912	C1qtnf9	+
3	127536849	127536854	Larp7	-
1	155097863	155097868	Ier5	-
4	116641899	116641904	Akr1a1	-
11	5704395	5704400	Mrps24	-
11	52122069	52122074	Ppp2ca	+
4	24903413	24903418	Ndufaf4	+
5	24593731	24593736	Smarcd3	-
2	61800152	61800157	Psmd14	+
3	108012453	108012458	Gstm1	-
10	127660625	127660630	Stat6	+
17	23827513	23827518	Elob	-
15	98931548	98931553	Tuba1b	-
7	45587726	45587731	Bcat2	+
14	76507231	76507236	Tsc22d1	+
5	129746355	129746360	Nipsnap2	+
7	122093652	122093657	Ndufab1	-

3	144691224	144691229	Sh3glb1	-
12	86162413	86162418	Ift43	+
2	112467208	112467213	Emc7	+
2	173034198	173034203	Rbm38	+
2	32634254	32634259	Ak1	+
9	50604009	50604014	Timm8b	+
16	38378063	38378068	Popdc2	+
17	45568496	45568501	Hsp90ab1	-
4	91852251	91852256	Gm12671	-
19	24876203	24876208	Gm10053	+
7	24888928	24888933	Rps19	+
5	110784748	110784753	Ulk1	-
10	7663236	7663241	Pcmt1	-
7	126796664	126796669	Aldoa	-
1	75245484	75245489	Dnajb2	+
16	4482797	4482802	Srl	-
4	126275345	126275350	Trappc3	+
17	28062344	28062349	Anks1	+
15	79028729	79028734	H1f0	+
4	155074717	155074722	Rer1	-
16	94364557	94364562	Pigp	-
11	69999473	69999478	Phf23	+
2	130281709	130281714	Idh3b	-
11	101419044	101419049	Ptges3l;Gm2	-
5	117282914	117282919	Pebp1	-
8	83570929	83570934	Ndufb7	+
4	138321001	138321006	Pink1	-
13	54593641	54593646	Cltb	-
17	27752412	27752417	D17Wsu92e	-
2	172380612	172380617	Cstf1	+
12	54179711	54179716	Egln3	-
12	110691079	110691084	Hsp90aa1	-
6	126840634	126840639	Ndufa9	-
11	115649839	115649844	Grb2	-
7	100485859	100485864	Ucp3	+
5	115560895	115560900	Rplp0	+
10	81180050	81180055	Eef2	+
2	166945894	166945899	Cse1l	+
6	115618934	115618939	Raf1	-
1	171238296	171238301	Ndufs2	-
12	111546550	111546555	Eif5	+
10	59396537	59396542	Gm10273	+
17	66083473	66083478	Ndufv2	-
15	76345538	76345543	Cyc1	+
11	116078820	116078825	Wbp2	-

6	71874818	71874823	Immt	+
2	76704884	76704889	Ttn	-
3	51408199	51408204	Ndufc1	-
18	36402559	36402564	Cystm1	+
13	111593366	111593371	Gpbp1	-
10	128548239	128548244	Rpl41	-
15	79672321	79672326	Tomm22	+
2	32402557	32402562	Ptges2	+
19	27261050	27261055	AC119982.1	-
2	74876309	74876314	Mtx2	+
12	55489893	55489898	Nfkbia	-
6	73270994	73270999	Suclg1	+
9	56136007	56136012	Tspan3	-
7	51747812	51747817	Gm7336	+
17	86832263	86832268	Epas1	+
6	71214426	71214431	Smyd1	-
1	156366264	156366269	Gm2000	+
14	21832929	21832934	Vdac2	+
9	122176453	122176458	Ano10	-
4	127311644	127311649	Gja4	-
5	115801633	115801638	Rab35	-
7	13035897	13035902	Ube2m	-
5	122469385	122469390	Atp2a2	-
9	40802779	40802784	Hspa8	+
17	70994613	70994618	Myl12a;Myl1:	-
2	122265259	122265264	Sord	+
9	53581939	53581944	Acat1	-
7	28832133	28832138	Ech1	+
10	91123973	91123978	Slc25a3	-
18	80212417	80212422	Gm16286;Tx	+
2	121457245	121457250	Serf2;Hypk	+
13	75645025	75645030	Gm4149	+
8	93166176	93166181	Ces1d	-
16	57391466	57391471	Cmss1	-
10	81559637	81559642	Aes	+
4	129849865	129849870	Ptp4a2	+
12	84372526	84372531	Coq6	+
11	62317743	62317748	Ncor1	-
7	25625082	25625087	Dmac2	+
9	55487448	55487453	Etfa	-
12	111961445	111961450	Atp5mpl	-
11	5780296	5780301	Ube2d-ps	+
9	120572779	120572784	Rpl14	+
11	120456731	120456736	Oxld1	-
12	85132743	85132748	Dlst	+

2	181857054	181857059	Pcmtd2	+
4	3973414	3973419	Gm11808	-
9	55488900	55488905	Etfa	-
9	56136074	56136079	Tspan3	-
10	62494737	62494742	Srgn	-
5	134238186	134238191	Gtf2i	-
13	111593459	111593464	Gpbp1	-
17	65582685	65582690	Vapa	-
17	25782105	25782110	Narfl	+
19	7427279	7427284	Rtn3	-
11	100361628	100361633	Eif1	-
3	116581238	116581243	Lrrc39	+
8	69895251	69895256	Ndufa13;Yjef	-
4	152271725	152271730	Acot7	+
19	8969439	8969444	Eef1g	+
4	130127901	130127906	Pef1	+
15	102281015	102281020	Mfsd5	+
6	136872631	136872636	Mgp	-
3	102142219	102142224	Casq2	+
5	124461969	124461974	Kmt5a	+
1	92919868	92919873	Rnpepl1	+
7	99479877	99479882	Rps3	-
4	155074445	155074450	Rer1	-
6	125460513	125460518	Cd9	-
16	91562994	91562999	Ifngr2	+
9	44348300	44348305	Vps11	-
5	75933195	75933200	Kdr	-
4	154157909	154157914	Tprgl	-
11	40679349	40679354	Mat2b	-
11	70978082	70978087	C1qbp	-
14	65975693	65975698	Clu	+
17	74492490	74492495	Yipf4	+
16	4480280	4480285	Srl	-
18	50091502	50091507	Tnfaip8	+
4	154897384	154897389	Fam213b	-
7	27459626	27459631	Blvrb	+
17	87433846	87433851	Calm2	-
6	113340380	113340385	Camk1	-
11	51985735	51985740	Ube2b;Gm2(	-
12	111724363	111724368	Apopt1	+
18	12156205	12156210	Riok3	+
6	57689308	57689313	Pyurf	-
7	17004801	17004806	Ppp5c	-
13	45507680	45507685	Gmpr	+
8	70868806	70868811	Ccdc124	-

17	24722399	24722404	Ndufb10	-
8	72586417	72586422	Tmem38a	+
6	133106036	133106041	Smim10l1	+
2	104427466	104427471	Hipk3	-
4	139105027	139105032	Minos1	-
8	71368549	71368554	Use1	+
9	90096545	90096550	Morf4l1	-
5	116409353	116409358	Hspb8	-
2	156863197	156863202	Rab5if	+
10	79685340	79685345	Cdc34	+
9	54601068	54601073	Idh3a	+
12	85132688	85132693	Dlst	+
9	71504953	71504958	Myzap	-
7	45458236	45458241	Ftl1	-
11	50171788	50171793	Tbc1d9b	+
11	33156034	33156039	Npm1	-
11	58995038	58995043	Obscn	-
11	58995361	58995366	Obscn	-
12	110858764	110858769	Mpc1-ps	-
10	57802548	57802553	Smpdl3a	+
5	129757307	129757312	Nipsnap2	+
2	30402541	30402546	Crat	-
16	92112010	92112015	Mrps6;Gm49	+
5	24581862	24581867	Gm10221	-
16	4480955	4480960	Srl	-
3	93564496	93564501	S100a10	+
7	102111049	102111054	Art1	+
9	122176328	122176333	Ano10	-
16	23613588	23613593	Rtp4	+
5	121567817	121567822	Aldh2	-
2	32631074	32631079	Ak1	+
8	123893215	123893220	Acta1	-
9	96896183	96896188	Gm10123	+
17	8297626	8297631	Mpc1	+
13	74407087	74407092	Mir692-3;Ftl1	+
18	67405381	67405386	Afg3l2	-
7	19420228	19420233	Ckm	+
8	121597693	121597698	Map1lc3b;Gr	+
10	128089125	128089130	Atp5b	+
14	73591128	73591133	Sucla2	+
18	34938164	34938169	Hspa9	-
18	61130602	61130607	Csf1r	+
9	108303985	108303990	Tcta	-
6	5484963	5484968	Pdk4	-
6	136813916	136813921	Wbp11	-

7	141459802	141459807	Pnpla2	+
17	44037033	44037038	Rcan2	+
17	45571462	45571467	Hsp90ab1	-
17	13013589	13013594	Sod2	+
9	77239560	77239565	Mlip	-
19	37221662	37221667	March5	+
1	171240955	171240960	Ndufs2	-
19	5423784	5423789	Drap1	-
12	87449846	87449851	Slirp	+
5	73313635	73313640	Ociad1	+
5	21803690	21803695	Psmc2	+
10	117051102	117051107	Cct2	-
4	147873999	147874004	Mfn2	-
9	54604087	54604092	Idh3a	+
7	141429939	141429944	Slc25a22	-
17	75537325	75537330	Fam98a	-
18	67429018	67429023	Afg3l2	-
13	107414433	107414438	Apoo-ps	-
16	37836202	37836207	Fstl1	+
5	91626043	91626048	Parm1	+
12	113153656	113153661	Crip1	+
7	79142901	79142906	Mfge8	-
16	4480606	4480611	Srl	-
1	10027142	10027147	Cops5	-
2	108950624	108950629	Gm13910	+
9	37413632	37413637	Robo4	+
15	100620078	100620083	Dazap2	+
19	6389245	6389250	Pygm	+
17	27634211	27634216	Rps10;RPS1	-
15	43250156	43250161	Eif3e	-
1	172279680	172279685	Atp1a2	-
11	97048572	97048577	Mrpl10	+
7	30554699	30554704	Hspb6	+
8	121596769	121596774	Map1lc3b;Gr	+
9	21354694	21354699	Slc44a2	+
6	86525235	86525240	Pcbp1	-
8	84246539	84246544	D8Ertd738e	-
2	181670387	181670392	Sox18	-
3	75517442	75517447	Pdcd10	-
18	5118157	5118162	Svil	+
8	25022654	25022659	Tm2d2	+
3	146839303	146839308	Gm10288	-
4	82291090	82291095	Nfib	-
7	45883302	45883307	Kdelr1	+
7	78780465	78780470	Mrpl46	-

5	92414534	92414539	Art3	+
8	94850526	94850531	Coq9	+
11	60864593	60864598	Tmem11	-
7	141470817	141470822	Cd151	+
13	58127208	58127213	Hnrnpa0	-
11	95671435	95671440	Phb;Gm2683	+
7	19565304	19565309	Gemin7	-
1	161241405	161241410	Prdx6	-
13	100741172	100741177	Mrps36	-
19	32466542	32466547	Rpl9-ps6	-
14	63199100	63199105	Gata4	-
11	69942704	69942709	Slc2a4	-
3	107283896	107283901	Lamtor5	+
9	122381467	122381472	Abhd5	+
11	69397392	69397397	Tmem88	-
11	93949833	93949838	Nme2;Gm20	-
10	53342031	53342036	Pln	+
3	79603949	79603954	Etfdh	-
8	85539121	85539126	Dnaja2	-
2	174341673	174341678	Gnas	+
2	17344314	17344319	Nebl	-
8	70535111	70535116	Fkbp8	+
11	55395369	55395374	Sparc	-
1	165478253	165478258	Mpc2	+
2	30822838	30822843	Ntmt1	+
7	19152680	19152685	Snrpd2	+
3	88321576	88321581	Cct3	+
13	119336156	119336161	Nnt;Nnt	-
18	80295928	80295933	Kcng2	-
5	142903146	142903151	Actb	-
19	4193043	4193048	Ppp1ca	+
11	100321492	100321497	Eif1	+
7	142540666	142540671	Mrpl23	+
5	21748828	21748833	Pmpcb	+
9	59672023	59672028	Pkm	+
5	134620179	134620184	Eif4h	-
6	124714068	124714073	Phb2	+
4	130316215	130316220	Zcchc17	-
11	5780327	5780332	Ube2d-ps	+
16	64766203	64766208	4930453N24	-
11	102403830	102403835	Slc25a39	-
2	130172654	130172659	Snrpb	-
12	55414438	55414443	Psma6	+
15	81848346	81848351	Tob2	-
11	17203685	17203690	Pno1	-

11	95675226	95675231	Phb	+
7	140853868	140853873	Bet1l;Gm457	-
4	108044781	108044786	Podn;Scp2	-
1	64994393	64994398	Rpl10a-ps1	-
10	128363518	128363523	Coq10a	-
2	120507448	120507453	Zfp106	-
13	119336301	119336306	Nnt;Nnt	-
15	43252205	43252210	Eif3e	-
19	5844023	5844028	Neat1	-
11	101278246	101278251	Coa3	-
12	54860345	54860350	Cfl2	-
2	157556446	157556451	Blcap	-
18	35617057	35617062	Paip2	+
8	119410502	119410507	Mlycd	+
18	70625779	70625784	Mbd2	+
15	84314651	84314656	Parvb	+
7	116104905	116104910	1110004F10I	+
13	58127183	58127188	Hnrnpa0	-
16	20543892	20543897	Ap2m1	+
2	156873119	156873124	Rab5if	+
10	77598700	77598705	Pttg1ip	+
19	6911752	6911757	Esrra	-
8	93166155	93166160	Ces1d	-
5	22612871	22612876	Rpl17-ps5	-
6	125460555	125460560	Cd9	-
1	125393066	125393071	Actr3	-
13	46430886	46430891	Rbm24	+
2	91059297	91059302	Psmc3	+
4	136896595	136896600	C1qa	-
5	73313495	73313500	Ociad1	+
6	71816009	71816014	Mrpl35	-
16	23113252	23113257	Eif4a2	+
5	116408947	116408952	Hspb8	-
13	44922268	44922273	Dtnbp1	-
2	30173689	30173694	Endog	+
19	9829689	9829694	AC132253.9	-
1	63147322	63147327	Ndufs1	-
7	141470853	141470858	Cd151	+
19	10903043	10903048	Prpf19	+
11	62552660	62552665	Ubb	+
6	85134175	85134180	Spr	-
1	16677037	16677042	Tmem70	+
15	81849000	81849005	Tob2	-
3	95740262	95740267	Tars2	-
9	109058530	109058535	Trex1;Atrip	-

7	99154566	99154571	Dgat2	-
6	133106233	133106238	Smim10l1	+
2	156873246	156873251	Rab5if	+
5	122456416	122456421	Atp2a2	-
1	92460412	92460417	Ndufa10	-
4	117154708	117154713	Rps8	-
3	79622850	79622855	Etfdh	-
15	76344886	76344891	Cyc1	+
11	60952007	60952012	Map2k3	+
10	80828627	80828632	Oaz1	+
2	76705158	76705163	Ttn	-
11	6596660	6596665	Ccm2	+
11	52385675	52385680	Vdac1	+
11	100320758	100320763	Eif1	+
9	104127248	104127253	Acad11	+
11	120543221	120543226	Mcrip1	-
3	153930352	153930357	Acadm	-
3	90512060	90512065	S100a1	-
13	119357591	119357596	Nnt;Nnt	-
11	95491956	95491961	Spop	+
16	14300049	14300054	Fopnl	-
17	25782637	25782642	Narfl	+
3	88729488	88729493	Rit1	+
6	90645809	90645814	Slc41a3	+
5	5781759	5781764	Gm15459	-
8	11198714	11198719	Col4a1	-
15	99409756	99409761	Tmbim6	+
6	87850977	87850982	Cnbp	-
12	54860504	54860509	Cfl2	-
5	21742785	21742790	Pmpcb	+
7	140111702	140111707	Echs1	-
16	4481914	4481919	Srl	-
12	84313954	84313959	Ptgr2	+
19	46377225	46377230	Actr1a	-
18	70622687	70622692	Mbd2	+
15	79527970	79527975	Ddx17	-
2	121548629	121548634	Frmd5	-
11	120347334	120347339	Actg1	-
15	58782867	58782872	Tmem65	-
1	16677778	16677783	Tmem70	+
11	5896729	5896734	Myl7	-
17	25790967	25790972	Fam173a;AC	-
5	111388214	111388219	Pitpnb	+
1	134075696	134075701	Btg2	-
4	138314017	138314022	Pink1	-

10	78427466	78427471	Cstb	+
5	117286234	117286239	Pebp1	-
17	47687264	47687269	Tomm6;Gm2	-
18	34938648	34938653	Hspa9	-
6	34304183	34304188	Akr1b3	-
5	122458137	122458142	Atp2a2	-
4	155957399	155957404	Ube2j2	+
19	43502959	43502964	Got1	-
1	75216214	75216219	Tuba4a	-
18	64504983	64504988	Nars	-
10	57811669	57811674	Smpdl3a	+
11	95680476	95680481	Phb	+
10	80293794	80293799	Rps15	+
8	128732427	128732432	Itgb1	+
5	17828977	17828982	Cd36	-
1	180810155	180810160	H3f3a	-
10	95547888	95547893	Nudt4	-
7	15917469	15917474	Selenow	-
2	84765726	84765731	Serping1	-
2	91134193	91134198	Mybpc3	+
3	53692585	53692590	Gm6204	-
12	72656434	72656439	Dhrs7	-
2	84770206	84770211	Serping1	-
3	51407400	51407405	Ndufc1	-
11	32297188	32297193	Hba-a2	+
2	50280147	50280152	Mmadhc	-
18	41875672	41875677	Prelid2	-
17	27122710	27122715	Uqcc2	-
19	5492618	5492623	Cfl1	+
1	95498812	95498817	Gm15427	+
2	174330390	174330395	Gnas	+
16	20125335	20125340	Klhl24	+
6	57689525	57689530	Gm26712;Py	-
5	88660244	88660249	Grsf1	-
13	66916645	66916650	Mterf3	-
10	128361923	128361928	Cs	+
5	23961403	23961408	Fam126a	-
11	29547692	29547697	Rps27a	-
1	135850207	135850212	Tnnt2	+
17	29702523	29702528	Cmtr1;Gm28	+
12	31333879	31333884	Dld	-
7	12923017	12923022	Rps5	+
16	84955332	84955337	App	-
17	36949967	36949972	Ppp1r11	-
2	145916066	145916071	Naa20	+

18	35616647	35616652	Paip2	+
14	21840484	21840489	Vdac2	+
3	123111563	123111568	Synpo2	-
11	78541440	78541445	Ift20	+
7	44855225	44855230	Akt1s1	+
4	109063450	109063455	Osbpl9	-
1	16643318	16643323	Eloc	-
10	42313612	42313617	Afg1l	-
5	145191520	145191525	Atp5j2	-
7	19697018	19697023	Apoe	-
11	70238280	70238285	Gm21988;Rr	-
18	50091349	50091354	Tnfaip8	+
4	123298578	123298583	Pabpc4	+
6	50564544	50564549	Cycs	-
8	72321047	72321052	Klf2	+
9	44742388	44742393	Arcn1	-
7	97406953	97406958	Ndufc2	+
10	60300212	60300217	Psap	+
17	24439294	24439299	Eci1	+
13	86044865	86044870	Cox7c	-
4	24901767	24901772	Ndufaf4	+
6	17665619	17665624	Capza2	+
18	76939634	76939639	Ier3ip1;Hdhd	+
8	94828330	94828335	Ciapin1	-
2	18675865	18675870	Commd3	+
10	60302462	60302467	Psap	+
11	116646652	116646657	Cygb	-
7	126546943	126546948	Eif3c	-
14	59564444	59564449	Cdadc1	-
9	120131036	120131041	Rpsa	+
11	84821026	84821031	Dhrs11	-
1	36692497	36692502	Cox5b	+
7	25625361	25625366	Dmac2	+
12	16825894	16825899	E2f6	+
6	35261255	35261260	1810058I24F	+
5	115298619	115298624	Dynll1	-
17	33838130	33838135	Ndufa7	+
2	4935976	4935981	Phyh	+
16	4045451	4045456	Trap1	-
3	108426816	108426821	Sars	-
9	44408717	44408722	Rps25	+
9	120013975	120013980	Xirp1;Cx3cr1	-
11	48802365	48802370	Rack1	+
2	90902403	90902408	Ndufs3	-
11	116172264	116172269	Acox1	-

9	53583560	53583565	Acat1	-
7	45916897	45916902	Tmem143	+
7	25365675	25365680	Megf8	+
11	116172018	116172023	Acox1	-
2	152737121	152737126	Id1	+
9	78479448	78479453	Eef1a1	-
1	194960919	194960924	Cd34	+
3	108012655	108012660	Gstm1	-
2	30402042	30402047	Crat	-
14	21838453	21838458	Vdac2	+
6	142565518	142565523	Kcnj8	-
11	84821099	84821104	Dhrs11	-
19	6909827	6909832	Prdx5	-
5	115110714	115110719	Acads	-
10	14655714	14655719	Vta1	-
4	136049983	136049988	Rpl11	-
18	35616411	35616416	Paip2	+
5	73310340	73310345	Ociad1	+
5	122471220	122471225	Atp2a2	-
5	92392966	92392971	Art3	+
11	115798594	115798599	Tmem94	+
7	130764336	130764341	Tacc2	+
3	145879086	145879091	Rpl36a-ps2;[	+
4	154160610	154160615	Tprgl	-
10	42534746	42534751	Snx3	+
7	30553420	30553425	Hspb6	+
15	51786688	51786693	Eif3h	-
7	99153812	99153817	Dgat2	-
3	32737156	32737161	Ndufb5	+
3	103057151	103057156	Csde1	+
7	105555404	105555409	Smpd1	+
15	74749257	74749262	Lynx1	-
10	42534776	42534781	Snx3	+
14	103081166	103081171	Fbxl3	-
6	31141939	31141944	Lncpint	-
11	94491018	94491023	Epn3	-
14	76504807	76504812	Tsc22d1	+
17	8297310	8297315	Mpc1	+
1	193272451	193272456	G0s2	-
11	104584523	104584528	Myl4	+
10	95490245	95490250	Mrpl42	-
18	25133069	25133074	Fhod3	+
9	75345363	75345368	Gnb5	+
3	107283985	107283990	Lamtor5	+
5	3236544	3236549	Gm15772	+

7	44864658	44864663	Ptov1	-
2	150831733	150831738	Pygb	+
5	122323954	122323959	Pptc7	+
4	130316605	130316610	Zcchc17	-
2	30447064	30447069	Ptpa	+
10	80341048	80341053	Adamtsl5	-
5	115343102	115343107	Triap1	+
9	49042339	49042344	Usp28	+
1	125595199	125595204	Slc35f5	+
9	108947417	108947422	Uqcrc1	+
8	85325059	85325064	Mylk3	-
11	115608298	115608303	Mif4gd	-
11	94656530	94656535	Mrpl27	+
10	95490296	95490301	Mrpl42	-
5	134703215	134703220	Eln	-
11	95680487	95680492	Phb	+
5	92414472	92414477	Art3	+
7	19415011	19415016	Ckm	+
4	156220364	156220369	Perm1	+
17	29041443	29041448	Srsf3	+
5	117282906	117282911	Pebp1	-
16	18302761	18302766	Tango2	-
10	128920061	128920066	Rdh5;Bloc1s	-
2	91130830	91130835	Mybpc3	+
13	24813737	24813742	BC005537	+
18	76970747	76970752	Hdhd2;Hdhd2	+
15	41866013	41866018	Abra	-
9	44410174	44410179	Rps25	+
11	21321774	21321779	Ugp2	-
15	102473329	102473334	Pcbp2	+
2	150818851	150818856	Pygb	+
16	38374240	38374245	Popdc2	+
2	148875303	148875308	Cst3	-
2	155277705	155277710	Map1lc3a	+
9	44913268	44913273	Atp5l	-
4	115811090	115811095	Atpaf1	+
9	40802582	40802587	Hspa8	+
3	101581829	101581834	Atp1a1	-
17	46147907	46147912	Mad2l1bp	-
1	134424834	134424839	Adipor1	+
7	126797506	126797511	Aldoa	-
19	8890629	8890634	Lbhd1;18100	+
17	27630434	27630439	Rps10;RPS1	-
11	3917328	3917333	Tcn2	-
10	24188558	24188563	Stx7	+

7	66074411	66074416	Snrpa1	+
7	3706668	3706673	Rps9	+
17	72941828	72941833	Lbh	+
7	127257091	127257096	Dctpp1	-
5	8966434	8966439	Crot	-
18	67405268	67405273	Afg3l2	-
7	27158751	27158756	Egln2	-
10	91118180	91118185	Slc25a3	-
4	155808696	155808701	Mrpl20	+
5	67056820	67056825	Limch1	+
4	91852557	91852562	Gm12671	-
15	43510865	43510870	Emc2	+
5	45437862	45437867	Qdpr	-
10	91116639	91116644	Slc25a3	-
18	36667433	36667438	Sra1	-
9	120130308	120130313	Rpsa	+
8	71537144	71537149	Bst2	-
6	50564445	50564450	Cycs	-
1	128038672	128038677	Rpl28-ps1	+
1	180179016	180179021	Coq8a	-
14	120431436	120431441	Mbnl2	+
8	124880209	124880214	Gnpat	+
8	83572309	83572314	Tecr	-
15	89086931	89086936	Trabd	+
2	30822670	30822675	Ntmt1	+
7	28831247	28831252	Ech1	+
14	55570004	55570009	Dcaf11	+
11	70645171	70645176	Slc25a11	-
7	28831278	28831283	Ech1	+
1	131055061	131055066	Mapkapk2	-
17	57204269	57204274	C3	-
5	77351996	77352001	Igfbp7	-
11	94340167	94340172	Ankrd40	+
13	73960760	73960765	Brd9	+
2	104427577	104427582	Hipk3	-
2	163725717	163725722	Pkig	+
10	17845090	17845095	Txlnb	+
7	132557756	132557761	Oat;Fgfr2	-
3	7445060	7445065	Pkia	+
11	67815027	67815032	Dhrs7c	+
10	80828788	80828793	Oaz1	+
1	90612616	90612621	Cops8	+
4	59618502	59618507	Hsdl2	+
4	107200696	107200701	Tmem59	+
11	120456640	120456645	Oxld1	-

7	100486309	100486314	Ucp3	+
18	38251372	38251377	Dele1	+
8	80764557	80764562	Gab1	-
5	29766973	29766978	Dnajb6	+
6	145963819	145963824	Sspn	+
9	50596687	50596692	Sdhd	-
11	100715911	100715916	Rab5c	-
12	59043268	59043273	Trappc6b	-
19	43507392	43507397	Got1	-
2	84829870	84829875	Timm10	+
10	76709620	76709625	Col6a1	-
13	30540999	30541004	Uqcrfs1	-
5	122323246	122323251	Pptc7	+
6	52546700	52546705	Hibadh	-
6	142565864	142565869	Kcnj8	-
17	87137455	87137460	Socs5	+
14	120947051	120947056	Ipo5	+
9	44408774	44408779	Rps25	+
14	21838594	21838599	Vdac2	+
6	73256214	73256219	Suclg1	+
15	73809575	73809580	Ndufb4c;Mrc	-
14	63142434	63142439	Ctsb	+
7	141160725	141160730	Rnh1	-
9	54603797	54603802	Idh3a	+
10	80394613	80394618	Mbd3	-
9	106239709	106239714	Alas1	-
11	49678938	49678943	Gm12191;Cr	-
10	81564711	81564716	Aes	+
6	35261476	35261481	1810058I24F	+
3	96528581	96528586	Hfe2	+
4	147910196	147910201	Plod1	-
2	150830652	150830657	Pygb	+
7	128546405	128546410	Bag3	+
8	94857591	94857596	Polr2c	+
9	50634961	50634966	Dlat	-
14	120946172	120946177	Ipo5	+
19	10903432	10903437	Prpf19	+
2	180039457	180039462	Psma7	-
3	142302777	142302782	Pdlim5	-
13	46677888	46677893	Fam8a1	+
11	70527287	70527292	Psmb6	+
12	100207165	100207170	Calm1	+
1	175612316	175612321	Fh1	-
11	98800430	98800435	Msl1	+
7	114274450	114274455	Psma1	-

8	123103903	123103908	Rpl13;Gm20:	+
1	72711711	72711716	Rpl37a	+
6	38687704	38687709	Luc7l2	-
3	10015538	10015543	Fabp5	+
3	144597065	144597070	Selenof	+
10	128044760	128044765	Naca	+
1	131054354	131054359	Mapkapk2	-
11	95027917	95027922	Pdk2	-
3	95989143	95989148	Plekho1	-
17	24437437	24437442	Eci1	+
7	126489350	126489355	Tufm	+
12	32850737	32850742	Nampt	+
12	103321661	103321666	Asb2	-
19	32124095	32124100	Sgms1	-
2	119607157	119607162	1700020I14F	+
7	142376542	142376547	Gm49369;Ct	-
7	28391102	28391107	Med29	-
5	116013641	116013646	Prkab1	-
8	95870897	95870902	Got2	-
17	66080875	66080880	Ndufv2	-
14	37121026	37121031	Ghitm	-
3	86141267	86141272	Rps3a1	-
1	181903453	181903458	Enah	-
5	31141531	31141536	Mpv17	-
17	86833157	86833162	Epas1	+
1	171235317	171235322	Ndufs2	-
2	26911420	26911425	Rpl7a	+
5	33742398	33742403	Letm1	-
16	31254435	31254440	Ppp1r2	-
7	126780077	126780082	Ypel3	+
11	5524878	5524883	Xbp1	+
11	70238240	70238245	Gm21988;Rr	-
16	90225153	90225158	Sod1	+
1	63149986	63149991	Ndufs1	-
8	83571708	83571713	Tecr	-
1	38051794	38051799	Eif5b	+
13	32978309	32978314	Serpinb6b	+
18	76971043	76971048	Hdhd2;Hdhd:	+
9	108204891	108204896	Dag1	-
7	16327470	16327475	Sae1	-
7	132560818	132560823	Oat;Fgfr2	-
4	126232365	126232370	Map7d1	-
11	115607252	115607257	Mrps7	+
9	22092637	22092642	Gm6581	+
3	32737196	32737201	Ndufb5	+

1	180168786	180168791	Coq8a	-
1	92641934	92641939	Cops9	-
14	73579390	73579395	Sucla2	+
12	110858134	110858139	Wdr20	-
3	97694066	97694071	Pde4dip	-
15	37000561	37000566	Zfp706	-
13	58392078	58392083	Hnrnpk	-
3	7444916	7444921	Pkia	+
18	6201586	6201591	Kif5b	-
6	128313382	128313387	Rpl18-ps2	+
13	107414232	107414237	Apoo-ps	-
19	6136013	6136018	Arl2	-
5	45511124	45511129	Lap3	+
17	26213623	26213628	Fam234a	-
5	115345901	115345906	Cox6a1	-
4	147910459	147910464	Plod1	-
2	174343282	174343287	Gnas	+
1	91362275	91362280	Klhl30	+
1	24615727	24615732	Gm29216	-
19	45005577	45005582	Mrpl43	-
2	118880764	118880769	Ivd	+
7	24972563	24972568	Rabac1	-
11	31548246	31548251	Ncoa2	+
1	24613839	24613844	Gm28437	-
7	19697043	19697048	Apoe	-
10	128048504	128048509	Naca	+
4	139103964	139103969	Minos1	-
17	78378430	78378435	Fez2	-
11	80136963	80136968	Tefm	-
1	55444226	55444231	Gm6644	-
15	4101817	4101822	Oxct1	+
17	8141627	8141632	Gm49673;Rr	-
19	5424198	5424203	Drap1	-
7	28042720	28042725	Psmc4	-
6	86516077	86516082	Gm44386	-
2	25561966	25561971	Edf1	+
16	84831367	84831372	Atp5j	-
7	45338188	45338193	Hrc	+
13	98313274	98313279	Btf3	-
10	80395182	80395187	Mbd3	-
8	83435535	83435540	Scoc	-
7	73776837	73776842	Fam174b	+
11	87743574	87743579	Supt4a	+
14	34561744	34561749	Ldb3	-
2	163466527	163466532	Fitm2	-

7	44813217	44813222	Atf5	-
17	13010626	13010631	Sod2	+
7	108941634	108941639	Eif3f	+
17	26213071	26213076	Fam234a	-
7	102110980	102110985	Art1	+
1	90612732	90612737	Cops8	+
10	84614315	84614320	Tcp11l2	+
4	120757556	120757561	Nfyc	-
2	39015346	39015351	Arpc5l	+
15	75045532	75045537	Ly6c1	-
12	71123530	71123535	Timm9	-
9	64182298	64182303	Snapc5	+
13	93041048	93041053	Cmya5	-
11	4092288	4092293	Mtfp1	-
12	103330402	103330407	Asb2	-
2	164018003	164018008	Ywhab	+
15	58939477	58939482	Ndufb9	+
7	29174334	29174339	Psmd8	-
2	153619215	153619220	Commd7	-
12	8936006	8936011	Laptm4a	+
10	43525240	43525245	1700021F05I	-
17	33838186	33838191	Ndufa7	+
1	74397086	74397091	Ctdsp1	+
2	148872911	148872916	Cst3	-
1	75366816	75366821	Des	+
19	56740755	56740760	Adrb1	-
12	85342958	85342963	Tmed10	-
2	158117246	158117251	Tgm2	-
8	72152873	72152878	Tpm4	+
6	133106159	133106164	Smim10l1	+
4	109062130	109062135	Osbpl9	-
19	43503010	43503015	Got1	-
3	75517246	75517251	Pdcd10	-
2	36039752	36039757	Ndufa8	-
3	152237090	152237095	Nexn	-
10	128048512	128048517	Naca	+
15	73751846	73751851	Ptp4a3	+
8	104641349	104641354	Ciao2b	-
8	83571897	83571902	Tecr	-
14	55636361	55636366	Gm49378;Tr	-
5	115342991	115342996	Triap1	+
14	77852934	77852939	Dnajc15	-
3	90514284	90514289	S100a1	-
8	34090609	34090614	Dctn6	-
1	131053751	131053756	Mapkapk2	-

5	77087580	77087585	Hopx	-
3	97690563	97690568	Pde4dip	-
2	72407034	72407039	Map3k20	+
7	13033989	13033994	Chmp2a	-
19	27253643	27253648	Vldlr	+
17	33838283	33838288	Ndufa7	+
9	44408708	44408713	Rps25	+
15	79029415	79029420	H1f0	+
1	118299340	118299345	Tsn	-
3	105942938	105942943	Atp5f1	-
15	76645806	76645811	Cyhr1	-
7	45459676	45459681	Ftl1	-
7	30192190	30192195	Capns1	-
15	89156278	89156283	Plxnb2	-
5	67355564	67355569	Slc30a9	+
12	110691459	110691464	Hsp90aa1	-
15	75597915	75597920	Gpihbp1	+
11	51623218	51623223	Nhp2	+
3	153922562	153922567	Acadm	-
12	103442370	103442375	Ifi27l2a	-
11	120605315	120605320	Anapc11	+
17	56259771	56259776	Fem1a	+
15	98932555	98932560	Tuba1b;Gm4	-
9	123790503	123790508	AC165425.1;	-
5	121205563	121205568	Rpl6	+
18	67225644	67225649	Mppe1	-
10	127067142	127067147	Cdk4	+
11	21333727	21333732	Ugp2	-
3	20122392	20122397	Gyg	-
16	20663382	20663387	Psmd2	+
12	110898336	110898341	Rps19-ps6;T	+
7	19701472	19701477	Tomm40	-
3	130696393	130696398	Ostc	-
16	24392794	24392799	Lppos	-
6	71881041	71881046	Ptcd3	-
8	22782736	22782741	Plat	+
5	116014182	116014187	Prkab1	-
7	111072622	111072627	Eif4g2	-
19	45792436	45792441	Kcnip2	-
3	152242761	152242766	Nexn	-
11	94125633	94125638	Spag9	+
1	51302790	51302795	Cavin2	+
5	121814764	121814769	Atxn2	+
17	35061537	35061542	Ddah2	+
9	21008626	21008631	Mrpl4	+

13	114288826	114288831	Ndufs4	-
11	23003184	23003189	Cct4	+
6	125462347	125462352	Cd9	-
9	108944159	108944164	Uqcrc1	+
9	105054553	105054558	Mrpl3	+
15	74958788	74958793	Ly6e	+
1	180802771	180802776	H3f3a	-
7	28352563	28352568	Rps16	+
19	10900727	10900732	Prpf19	+
15	55534234	55534239	Mrpl13	-
5	115242488	115242493	Rnf10	-
10	127064653	127064658	Cdk4	+
7	122096655	122096660	Ndufab1	-
15	76904407	76904412	Rpl8	+
11	120578276	120578281	Arhgdia	-
11	96818678	96818683	Nfe2l1	-
4	136890433	136890438	C1qc	-
10	79675978	79675983	Tpgs1	+
12	76773325	76773330	Churc1;Fntb	+
10	60300853	60300858	Psap	+
10	76595978	76595983	Col6a2	-
5	53278098	53278103	Smim20	+
12	84756987	84756992	Npc2	-
2	125832170	125832175	Cops2	-
2	75676214	75676219	Nfe2l2	-
8	116889841	116889846	Cmc2	-
9	78474002	78474007	Mto1	+
15	6654059	6654064	Gm7666;Fyb	+
15	51786774	51786779	Eif3h	-
12	55418386	55418391	Psma6	+
16	18307973	18307978	Tango2	-
5	23839679	23839684	Tomm7	-
16	18410723	18410728	Comt	-
11	58996856	58996861	Obscn	-
4	135216139	135216144	Clic4	-
7	142376209	142376214	Gm49369;Ct	-
2	90894715	90894720	Ndufs3	-
13	43401518	43401523	Nol7	+
19	43500410	43500415	Got1	-
9	65689534	65689539	Oaz2	+
2	157556669	157556674	Blcap	-
7	127908558	127908563	Bckdk	+
8	104516145	104516150	Nae1	-
11	6296990	6296995	Ogdh	+
6	4008114	4008119	Gng11	+

3	90512016	90512021	S100a1	-
10	93861474	93861479	Metap2	-
5	69516898	69516903	Yipf7	-
16	92099637	92099642	Mrps6;Gm49	+
9	102594537	102594542	Cep63	-
7	81792271	81792276	Btbd1	-
2	91130914	91130919	Mybpc3	+
13	99320204	99320209	Ptcd2	-
7	34204141	34204146	Gpi1	-
19	4008485	4008490	Ndufv1	-
7	79134059	79134064	Mfge8	-
6	24604227	24604232	Lmod2	+
1	9942704	9942709	Snhg6	-
7	44471413	44471418	Josd2	+
18	74795198	74795203	Acaa2	+
9	108490417	108490422	Lamb2	+
10	81115043	81115048	Map2k2	+
13	99319860	99319865	Ptcd2	-
1	161241308	161241313	Prdx6	-
9	107300836	107300841	Cish	+
5	30624986	30624991	Kcnk3	+
5	121208919	121208924	Rpl6	+
3	130728816	130728821	Rpl34	-
5	95862474	95862479	Gm5559	+
17	25080354	25080359	Tmem204	-
11	120347249	120347254	Actg1	-
9	107300487	107300492	Cish	+
16	17154050	17154055	Ube2l3	-
9	122176007	122176012	Ano10	-
17	71112022	71112027	Myom1;Gm2	+
2	130281035	130281040	Idh3b	-
5	31137610	31137615	Trim54	+
4	33246012	33246017	Pnrc1	-
6	71875134	71875139	Immt	+
5	121205655	121205660	Rpl6	+
6	35261444	35261449	1810058I24R	+
11	93949915	93949920	Nme2;Gm20	-
8	71624802	71624807	Colgalt1	+
17	12913896	12913901	Mrpl18	-
7	139579326	139579331	Inpp5a	+
7	28788093	28788098	Sirt2	+
5	124493297	124493302	Rilpl1	-
1	170142031	170142036	Uap1	-
2	84453809	84453814	Tfpi	-
14	66084383	66084388	Ephx2	-

2	163469113	163469118	Fitm2	-
8	94854315	94854320	Coq9	+
1	180168581	180168586	Coq8a	-
4	116307388	116307393	Mast2	-
6	136640226	136640231	Plbd1	-
11	68902300	68902305	Rpl26	+
17	29041958	29041963	Srsf3	+
7	44867408	44867413	Ptov1	-
15	99402059	99402064	Tmbim6	+
3	60629274	60629279	Mbnl1	+
1	75362689	75362694	Des	+
19	47090602	47090607	Usmg5	-
13	74360826	74360831	Lrrc14b	-
15	75925345	75925350	Tsta3	-
17	56119828	56119833	Lrg1	-
5	5782280	5782285	Gm15459	-
14	55581436	55581441	Psme1	+
7	28766321	28766326	Nfkbib	-
15	100260081	100260086	Atf1	+
8	22578052	22578057	Vdac3	-
1	120113351	120113356	Dbi	-
4	10848872	10848877	Gm12918	+
2	4938559	4938564	Phyh	+
4	139288909	139288914	Capzb	+
10	81179435	81179440	Eef2	+
3	87915574	87915579	Hdgf	+
8	70868625	70868630	Ccdc124	-
9	108662319	108662324	Slc25a20	+
14	76506799	76506804	Tsc22d1	+
17	12974774	12974779	Wtap	-
14	55897489	55897494	Sdr39u1	-
6	142770578	142770583	Cmas	+
7	79451587	79451592	Polg	-
7	19565187	19565192	Gemin7	-
17	29296571	29296576	BC004004	+
4	3941289	3941294	Chchd7	+
17	33747056	33747061	Rab11b	-
7	28386843	28386848	Med29	-
7	30484759	30484764	Rps12-ps4	-
9	123790205	123790210	AC165425.1;	-
7	30554962	30554967	Hspb6	+
2	28470387	28470392	Mrps2	+
16	11220560	11220565	Gspt1	-
6	17341062	17341067	Cav1	+
14	32659790	32659795	3425401B19I	-

1	75215318	75215323	Tuba4a	-
11	86630413	86630418	Vmp1	-
12	84774714	84774719	Isca2	+
12	110976535	110976540	Ankrd9	-
11	52385590	52385595	Vdac1	+
11	21333639	21333644	Ugp2	-
10	78167440	78167445	D10Jhu81e	-
17	56259656	56259661	Fem1a	+
8	82344417	82344422	Il15	-
7	31055561	31055566	Fxyd1	-
5	88659974	88659979	Grsf1	-
7	126979862	126979867	Cdipt	+
19	7057144	7057149	Macrod1	+
14	54949469	54949474	Myh6	-
2	119660296	119660301	Ndufaf1	-
3	95663069	95663074	Mcl1	+
5	117377594	117377599	Wsb2	+
17	75537166	75537171	Fam98a	-
8	24438345	24438350	Tcim	-
4	15917735	15917740	Decr1	-
8	22579071	22579076	Vdac3	-
3	88929471	88929476	Dap3	-
2	122151650	122151655	B2m	+
12	69584555	69584560	Sos2	-
7	120659470	120659475	Uqcrc2	+
19	24875834	24875839	Gm10053	+
17	56721391	56721396	Ndufa11	+
15	82041728	82041733	Snu13	-
7	93179603	93179608	Gm15501	-
1	171173340	171173345	Pcp4l1	-
7	15949497	15949502	Ehd2	-
7	98716976	98716981	Thap12	+
11	95492087	95492092	Spop	+
18	38262487	38262492	Dele1	+
16	38378146	38378151	Popdc2	+
2	67526217	67526222	Xirp2	+
15	27593783	27593788	Ank	+
2	132311762	132311767	Cds2	+
2	32234036	32234041	Prrc2b	+
1	37897728	37897733	Mrpl30	+
19	32466376	32466381	Rpl9-ps6	-
14	8170385	8170390	Pdhb	-
11	100887283	100887288	Stat3	-
4	33246344	33246349	Pnrc1	-
16	4480597	4480602	Srl	-

15	79761883	79761888	Dnal4	-
1	63179318	63179323	Eef1b2	+
10	14655653	14655658	Vta1	-
1	51301992	51301997	Cavin2	+
8	111622772	111622777	Znrf1	+
10	79711686	79711691	Bsg	+
13	54499787	54499792	Gm2830	-
12	16536493	16536498	Lpin1	-
11	69990265	69990270	Ctdnep1	+
3	32934948	32934953	Usp13	+
1	86909027	86909032	Gm6136	-
1	172273116	172273121	Atp1a2	-
9	15262193	15262198	Med17	-
2	173779258	173779263	Vapb	+
1	171219355	171219360	Tomm40l	-
19	8916212	8916217	Ganab	+
1	157420022	157420027	2810025M15	+
10	97513596	97513601	Dcn	+
10	128920653	128920658	Rdh5;Bloc1s	-
16	16268523	16268528	Pkp2	+
11	115410708	115410713	Mrpl58	+
5	129022998	129023003	Ran	+
7	98717897	98717902	Thap12	+
8	85538421	85538426	Dnaja2	-
10	29698998	29699003	Gm10275	-
19	3455249	3455254	Ppp6r3	-
6	51466628	51466633	Hnrnpa2b1	-
5	122323858	122323863	Pptc7	+
11	70010309	70010314	Acadvl	-
14	61236856	61236861	Sgcg	-
6	85134111	85134116	Spr	-
9	110981837	110981842	Lrrc2	+
7	31051939	31051944	Fxyd1	-
19	34246105	34246110	Acta2	-
15	5121118	5121123	Gm10250	-
17	65613340	65613345	Vapa	-
4	134525901	134525906	Mtfr1l	-
2	19394839	19394844	Msrb2	+
9	43222433	43222438	Oaf	-
1	161247285	161247290	Prdx6	-
8	95864518	95864523	Got2	-
5	104079445	104079450	Sparcl1	-
5	122459516	122459521	Atp2a2	-
7	105641182	105641187	Timm10b;Gn	+
16	38362880	38362885	Popdc2	+

17	48419602	48419607	Apobec2	-
19	6397679	6397684	Pygm	+
8	27275485	27275490	Eif4ebp1	+
11	78290442	78290447	2610507B11I	+
7	100997552	100997557	P2ry2	-
7	45021425	45021430	Rras	+
3	36449233	36449238	Anxa5	-
11	70013063	70013068	Acadvl	-
11	61343462	61343467	Slc47a1	-
19	7034705	7034710	Stip1	-
8	85080392	85080397	Wdr83	-
16	20692447	20692452	Eif4g1	+
11	121328355	121328360	Wdr45b	-
3	104656493	104656498	Slc16a1	+
3	79604137	79604142	Etfdh	-
1	75360852	75360857	Des	+
9	120960096	120960101	Ctnnb1	+
11	101278317	101278322	Coa3	-
2	76705184	76705189	Ttn	-
8	27275329	27275334	Eif4ebp1	+
17	29137103	29137108	Rpl35a-ps3	-
14	54944743	54944748	Myh6	-
4	41012278	41012283	Nfx1	+
6	83806416	83806421	Paip2b	-
8	72585965	72585970	Tmem38a	+
11	95027118	95027123	Pdk2	-
5	67057026	67057031	Limch1	+
7	28305582	28305587	Timm50	-
9	120129170	120129175	Rpsa	+
1	40855930	40855935	Tmem182	+
14	25700229	25700234	Ppif	+
10	80665627	80665632	Mknk2	-
17	87433957	87433962	Calm2	-
8	111622948	111622953	Znrf1	+
19	6058274	6058279	Fau	+
3	95661852	95661857	Mcl1	+
10	127064934	127064939	Cdk4	+
7	127848804	127848809	Stx4a	+
17	6984172	6984177	Rnaset2b;Gr	+
1	175606076	175606081	Fh1	-
16	4486451	4486456	Srl	-
17	20965276	20965281	Ppp2r1a	+
2	127297729	127297734	Stard7	+
4	155740871	155740876	Atad3a	-
10	59988105	59988110	Anapc16	-

2	24974340	24974345	Mrpl41	-
2	177478974	177478979	Zfp970	+
3	19691826	19691831	Trim55	+
12	36109753	36109758	Bzw2	-
7	79133830	79133835	Mfge8	-
4	141115413	141115418	Szrd1	-
3	108427316	108427321	Sars	-
11	70012605	70012610	Acadvl	-
11	58933116	58933121	Rnf187	-
7	142376003	142376008	Gm49369;Ct	-
11	69968503	69968508	Elp5	-
13	97131096	97131101	Nsa2	-
11	59211597	59211602	Arf1	-
8	128724237	128724242	Itgb1	+
15	85463001	85463006	Atxn10	+
8	107045534	107045539	Vps4a	+
4	154157495	154157500	Tprgl	-
3	102145627	102145632	Casq2	+
13	75850027	75850032	Glrx	+
9	106658983	106658988	Tex264	-
1	90215433	90215438	Ackr3	+
9	108565945	108565950	Ndufaf3	-
16	17153429	17153434	Ube2l3	-
10	40289054	40289059	Amd1	-
9	116040887	116040892	Gm9385	-
17	56259477	56259482	Fem1a	+
4	119282372	119282377	Ybx1	-
3	144692102	144692107	Sh3glb1	-
10	57516428	57516433	Serinc1	-
6	71874874	71874879	Immt	+
4	133277547	133277552	Tmem222	-
6	24603434	24603439	Lmod2	+
3	123016557	123016562	Myoz2	-
19	53866844	53866849	Rbm20	+
14	63152413	63152418	Fdft1	-
3	142304454	142304459	Pdlim5	-
1	66831215	66831220	Acadl	-
7	142380841	142380846	Gm49369;Ct	-
17	33691787	33691792	March2	-
12	31351347	31351352	Dld	-
15	76721866	76721871	Lrrc24;C030	-
1	66853204	66853209	Acadl	-
2	35303216	35303221	Gsn	+
4	136551019	136551024	Kdm1a	-
5	117282896	117282901	Pebp1	-

17	29328620	29328625	Pi16	+
2	70021236	70021241	Ubr3	+
7	84634265	84634270	Zfand6	-
11	100320143	100320148	Eif1	+
16	31948218	31948223	0610012G03	-
17	47378522	47378527	Mrps10	+
9	50764772	50764777	1110032A03I	-
2	91056008	91056013	Psmc3	+
7	142376088	142376093	Gm49369;Ct	-
8	22580415	22580420	Vdac3	-
10	13515234	13515239	Fuca2	+
4	75278116	75278121	Dmac1	-
17	24852794	24852799	Hagh	+
5	137529191	137529196	Gnb2;Epo	-
7	29177157	29177162	Psmd8	-
17	26218195	26218200	Fam234a	-
10	60299179	60299184	Psap	+
5	104059746	104059751	Nudt9	+
15	89371830	89371835	Sco2	-
6	112459815	112459820	Cav3	+
1	4776653	4776658	Mrpl15	-
10	43440246	43440251	Pdss2	+
14	26656296	26656301	Arf4	+
4	118414872	118414877	Med8	+
4	133266713	133266718	Tmem222	-
10	80293850	80293855	Rps15	+
17	25782169	25782174	Narfl	+
8	70895945	70895950	Rpl18a	-
5	115295957	115295962	Coq5	+
6	34304327	34304332	Akr1b3	-
19	36117859	36117864	Ankrd1	-
3	94886911	94886916	Psmb4	-
14	46775829	46775834	Cnih1	-
13	64363926	64363931	Ctsl	-
7	3662958	3662963	Leng1	-
1	128038847	128038852	Rpl28-ps1	+
17	24639701	24639706	Slc9a3r2	-
6	17341046	17341051	Cav1	+
14	101634171	101634176	Commd6	-
15	57871120	57871125	Derl1	-
2	36039831	36039836	Ndufa8	-
8	71475040	71475045	Dda1	+
9	71555716	71555721	Myzap	-
2	28933594	28933599	Cfap77	-
11	70654398	70654403	Pfn1	-

6	47525729	47525734	Cul1	+
7	5097962	5097967	Epn1	+
5	104088501	104088506	Sparcl1	-
2	131178120	131178125	Cenpb;Spef1	-
11	68902312	68902317	Rpl26	+
5	125386866	125386871	Ubc	-
10	88743873	88743878	Arl1	+
4	136268048	136268053	Tcea3	+
9	71515158	71515163	Myzap	-
5	77349389	77349394	Igfbp7	-
19	6397629	6397634	Pygm	+
7	89851753	89851758	Me3	+
10	29699133	29699138	Gm10275	-
17	33692028	33692033	March2	-
3	135466153	135466158	Ube2d3	+
11	52388921	52388926	Vdac1	+
14	51905836	51905841	Ndrg2	-
2	130175430	130175435	Snrpb	-
9	53592022	53592027	Acat1	-
3	32737124	32737129	Ndufb5	+
19	27253749	27253754	Vldlr	+
2	163625024	163625029	Serinc3	-
19	40243463	40243468	Pdlim1	-
1	45345014	45345019	Col3a1	+
5	92441527	92441532	Scarb2	-
8	107581269	107581274	Psmd7	-
11	116132034	116132039	Mrpl38	-
17	24897080	24897085	Mrps34;Nme	+
7	141010437	141010442	Ifitm3	-
11	97048761	97048766	Mrpl10	+
17	24728417	24728422	Rpl3l	+
13	3565733	3565738	Gdi2	+
17	26506095	26506100	Dusp1	-
2	10069710	10069715	Atp5c1	-
19	9019084	9019089	Ahnak	+
3	150073221	150073226	Rpsa-ps10	-
8	71592323	71592328	Pgls	+
4	116638990	116638995	Akr1a1	-
9	66065520	66065525	Ppib	+
7	141160676	141160681	Rnh1	-
9	75345119	75345124	Gnb5	+
14	51905989	51905994	Ndrg2	-
14	118163206	118163211	Gpr180	+
8	82334488	82334493	Il15	-
11	78501865	78501870	Vtn	+

4	46115854	46115859	Tmod1	+
15	102096867	102096872	Eif4b	+
14	24493415	24493420	Rps24	+
19	36115501	36115506	Ankrd1	-
13	43402928	43402933	Ranbp9	-
6	136617316	136617321	Plbd1	-
13	55597123	55597128	Tmed9	+
7	19565071	19565076	Gemin7	-
10	80054569	80054574	Gpx4	+
7	51747725	51747730	Gm7336	+
2	30402774	30402779	Crat	-
15	76904970	76904975	Rpl8	+
6	50565568	50565573	Cycs	-
15	4155036	4155041	Oxct1	+
12	71123370	71123375	Timm9	-
7	137461397	137461402	Glrx3	+
15	89418812	89418817	Gm44502;Cp	-
11	106058969	106058974	Dcaf7	+
11	101247672	101247677	Ramp2	+
4	10848484	10848489	Gm12918	+
7	48839522	48839527	Csrp3	-
15	98932018	98932023	Tuba1b	-
19	29020895	29020900	Ak3	-
11	6428332	6428337	H2afv	-
1	80267219	80267224	Cul3	-
6	83119267	83119272	Wbp1	-
19	10903015	10903020	Prpf19	+
6	72154212	72154217	St3gal5	+
1	181242154	181242159	Rpl35a-ps2	-
9	123023249	123023254	Tmem42;Gm	+
1	131055942	131055947	Mapkapk2	-
9	96333582	96333587	Atp1b3	-
12	110858232	110858237	Wdr20	-
8	22569369	22569374	Slc20a2	+
11	52246333	52246338	Skp1a	+
2	130281239	130281244	Idh3b	-
14	76506725	76506730	Tsc22d1	+
17	8296841	8296846	Mpc1	+
11	52388502	52388507	Vdac1	+
19	4127761	4127766	Tmem134	+
5	125386340	125386345	Ubc	-
16	91425155	91425160	Il10rb	+
6	145216212	145216217	Etfrf1	+
9	60874038	60874043	Uaca	+
13	32701131	32701136	Mylk4	-

19	18581271	18581276	Ostf1	-
7	30727839	30727844	Tmem147	-
13	63302578	63302583	2010111I01F	+
11	69994830	69994835	Gabarap	+
15	43527674	43527679	Emc2	+
14	51906983	51906988	Ndrg2	-
2	38642217	38642222	Psmb7	-
10	79709794	79709799	Bsg	+
5	104061708	104061713	Nudt9	+
13	43394801	43394806	Sirt5	+
8	13253949	13253954	Adprhl1	-
2	91134577	91134582	Mybpc3	+
8	84890760	84890765	Gcdh	-
19	46551634	46551639	Arl3	-
4	139648380	139648385	Gm21969;Al	+
10	19894764	19894769	Pex7	-
12	65063716	65063721	Fkbp3	-
15	43278331	43278336	Eif3e	-
9	103210515	103210520	Gm20425;Tr	-
17	45585418	45585423	Slc29a1	-
6	87843406	87843411	Cnbp	-
1	72859137	72859142	Igfbp5	-
2	131938344	131938349	Prnp;Prn	+
5	53277993	53277998	Smim20	+
7	27465749	27465754	Blvrb	+
14	32191381	32191386	Timm23	-
11	93955847	93955852	Nme2;Gm20	-
9	108340093	108340098	Gpx1	+
1	63160970	63160975	Ndufs1	-
2	103022093	103022098	Pdhx	-
15	76721971	76721976	Lrrc24;C030	-
15	74958857	74958862	Ly6e	+
11	52121910	52121915	Ppp2ca	+
15	74958921	74958926	Ly6e	+
3	96528341	96528346	Hfe2	+
3	108012426	108012431	Gstm1	-
12	40036210	40036215	Arl4a	-
14	59348828	59348833	Phf11d	-
2	84671443	84671448	Gm28635;Tr	-
11	70982256	70982261	C1qbp	-
15	81899133	81899138	Aco2	+
11	94125619	94125624	Spag9	+
9	120573777	120573782	Rpl14	+
11	52385659	52385664	Vdac1	+
9	64725502	64725507	Rab11a	-

5	143505745	143505750	Rac1	-
10	88477070	88477075	Chpt1	-
7	93179230	93179235	Gm15501	-
3	79604946	79604951	Etfdh	-
1	53321102	53321107	Osgepl1	+
1	36423483	36423488	Lman2l	-
14	25699951	25699956	Ppif	+
9	120960022	120960027	Ctnnb1	+
7	81535126	81535131	Fsd2	-
19	4035912	4035917	Gstp1	-
2	22588124	22588129	Gm13340	-
13	74323910	74323915	Sdha	-
5	124493108	124493113	Rilpl1	-
6	83119113	83119118	Wbp1	-
10	127498524	127498529	R3hdm2	+
13	3565300	3565305	Gdi2	+
2	90898406	90898411	Ndufs3	-
11	70651974	70651979	Pfn1	-
8	84842558	84842563	Calr	-
3	20122495	20122500	Gyg	-
8	24438411	24438416	Tcim	-
8	122883379	122883384	Gm20388	-
4	107064591	107064596	Mrpl37	-
11	70238167	70238172	Gm21988;Rr	-
11	119919400	119919405	Chmp6	+
1	86530148	86530153	Ptma	+
5	92412162	92412167	Art3	+
11	116849047	116849052	Mettl23	+
6	113710838	113710843	Tatdn2	+
4	117884638	117884643	Atp6v0b	-
14	73363072	73363077	Itm2b	-
17	15476064	15476069	Psmb1	-
12	55410127	55410132	Psma6	+
1	171294702	171294707	Ufc1	-
11	5803299	5803304	Pgam2	-
5	104093027	104093032	Sparcl1	-
18	5118637	5118642	Svil	+
2	150583997	150584002	Apmap	-
6	119924073	119924078	Wnk1	-
15	10447105	10447110	Dnajc21	-
17	35835250	35835255	Tubb5	-
15	58933865	58933870	Ndufb9;Gm4	+
1	16677358	16677363	Tmem70	+
12	84368590	84368595	Coq6	+
17	12683352	12683357	Igf2r	-

16	20560531	20560536	Abcf3	+
8	34170423	34170428	Saraf	+
2	18685954	18685959	Bmi1	+
8	121549608	121549613	Fbxo31	-
2	152280909	152280914	Csnk2a1	+
11	51685659	51685664	0610009B22I	-
7	73776615	73776620	Fam174b	+
6	39802398	39802403	Mrps33	-
4	147987043	147987048	Nppb	+
15	76171209	76171214	Plec	-
6	128430698	128430703	Fkbp4	-
19	10501783	10501788	Sdhaf2	-
11	69943357	69943362	Slc2a4	-
7	44854899	44854904	Akt1s1	+
14	50951556	50951561	Pnp;Gm4934	+
6	133105955	133105960	Smim10l1	+
9	120014319	120014324	Xirp1;Cx3cr1	-
3	153907365	153907370	Rabggtb	-
10	84613935	84613940	Tcp11l2	+
7	142377111	142377116	Gm49369;Ct	-
18	36742602	36742607	Ndufa2	-
5	100654983	100654988	Coq2	-
9	78479913	78479918	Eef1a1	-
7	19082415	19082420	Dmwd	+
14	65979725	65979730	Clu	+
10	53345162	53345167	Pln	+
10	81179945	81179950	Eef2	+
2	13579449	13579454	Vim	+
6	90619632	90619637	Slc41a3	+
2	90902915	90902920	Ndufs3	-
9	22448553	22448558	Rp9	-
13	46649594	46649599	Cap2	+
7	126780198	126780203	Ypel3	+
7	139579411	139579416	Inpp5a	+
7	118525837	118525842	Coq7	-
3	154827625	154827630	Tnni3k	-
14	101931207	101931212	Lmo7	+
16	17086989	17086994	Ppil2	-
9	72749422	72749427	Nedd4	+
11	70646193	70646198	Slc25a11	-
14	73365828	73365833	Itm2b	-
12	83993399	83993404	Acot2	+
5	30658148	30658153	Slc35f6	+
6	140747141	140747146	Rpl38-ps2	-
17	56614007	56614012	Rpl36	+

12	108106858	108106863	Setd3	-
8	72222366	72222371	Fam32a	+
6	24598013	24598018	Lmod2	+
11	6339900	6339905	Ogdh	+
11	95029698	95029703	Pdk2	-
13	90104591	90104596	Tmem167	+
16	38445856	38445861	Adprh	-
2	13574682	13574687	Vim	+
7	126696631	126696636	Bola2	+
11	97328759	97328764	Mrpl45	+
3	123006210	123006215	Myoz2	-
11	4737526	4737531	Zmat5	+
11	119918988	119918993	Chmp6	+
1	86543427	86543432	Pde6d	-
3	95862768	95862773	Mrps21	-
11	75410147	75410152	Serpinf1	-
8	13219021	13219026	Lamp1	-
19	5728312	5728317	Fam89b;Sss	-
9	65689289	65689294	Oaz2	+
1	134428257	134428262	Adipor1	+
6	5484775	5484780	Pdk4	-
18	35613435	35613440	Paip2	+
3	108016417	108016422	Gstm1	-
15	99037723	99037728	Tuba1c	+
15	38488617	38488622	Azin1	-
11	68973121	68973126	Rangrf	-
8	119348360	119348365	Hsbp1	+
11	97768254	97768259	Atp5l2-ps	+
3	95659380	95659385	Mcl1	+
5	114250461	114250466	Acacb	+
6	124716646	124716651	Phb2	+
6	34303987	34303992	Akr1b3	-
11	105989106	105989111	Ace	+
1	66831438	66831443	Acadl	-
16	90226166	90226171	Sod1	+
16	21934660	21934665	Tmem41a	-
7	140101651	140101656	Fuom	-
1	37987552	37987557	Txndc9	-
7	81792668	81792673	Btbd1	-
11	75705657	75705662	Crk	+
14	74742104	74742109	Esd	+
1	90215040	90215045	Ackr3	+
7	35643664	35643669	Pdcd5	-
8	46526335	46526340	Acsl1	+
10	3464798	3464803	Ppp1r14c	+

15	99408955	99408960	Tmbim6	+
8	70895388	70895393	Rpl18a	-
4	136880299	136880304	C1qb	-
5	104085137	104085142	Sparcl1	-
5	33372838	33372843	Maea	+
7	130985217	130985222	Htra1	+
11	88339943	88339948	Msi2	-
19	43502984	43502989	Got1	-
17	72941081	72941086	Lbh	+
11	70525939	70525944	Psmb6	+
10	121412109	121412114	Rassf3	-
5	92392671	92392676	Art3	+
15	31585408	31585413	Cmbl	+
3	97694024	97694029	Pde4dip	-
3	152237391	152237396	Nexn	-
5	122362179	122362184	Vps29	+
2	30185356	30185361	Kyat1	-
9	106235396	106235401	Alas1	-
7	141009639	141009644	Ifitm3	-
18	60812432	60812437	Cd74	+
1	58900718	58900723	Trak2	-
6	113737958	113737963	Sec13	-
4	138312274	138312279	Ddost	+
18	11085026	11085031	Gata6	+
6	34396900	34396905	Akr1b10	+
17	12915948	12915953	Mrpl18	-
7	120659231	120659236	Uqcrc2	+
5	117377541	117377546	Wsb2	+
8	13242633	13242638	Adprhl1	-
1	75215356	75215361	Tuba4a	-
1	63143797	63143802	Ndufs1	-
7	19295983	19295988	Rtn2	+
2	154694456	154694461	Chmp4b	+
17	5816423	5816428	Gm10231	-
11	88210702	88210707	Mrps23	+
10	79676042	79676047	Tpgs1	+
18	60777811	60777816	Rps14	+
2	25561910	25561915	Edf1	+
2	13077100	13077105	Rsu1	-
4	137320299	137320304	Cdc42	-
8	121596940	121596945	Map1lc3b;Gr	+
4	91852147	91852152	Gm12671	-
11	20335868	20335873	Gm12033	-
16	31253659	31253664	Ppp1r2	-
5	122466039	122466044	Atp2a2	-

7	67231608	67231613	Mef2a	-
2	91134496	91134501	Mybpc3	+
4	42980617	42980622	Vcp	-
6	86516059	86516064	Gm44386	-
11	106377697	106377702	Icam2	-
15	79028807	79028812	H1f0	+
5	17820503	17820508	Cd36	-
16	48842889	48842894	Retnla	+
11	52122595	52122600	Ppp2ca	+
13	43545702	43545707	Mcur1	-
2	68211226	68211231	Stk39	-
17	26838786	26838791	Nkx2-5	-
2	174346393	174346398	Gnas	+
4	57191290	57191295	Ptpn3	-
17	45568382	45568387	Hsp90ab1	-
18	77778514	77778519	Atp5a1	+
3	95989317	95989322	Plekho1	-
17	24437449	24437454	Eci1	+
4	40732994	40732999	Dnaja1	+
10	42313323	42313328	Afg1l	-
15	4154833	4154838	Oxct1	+
12	31333467	31333472	Dld	-
5	36795375	36795380	Mrfap1	-
2	25271091	25271096	Ssna1	-
18	80295613	80295618	Kcng2	-
17	24849280	24849285	Fahd1	-
16	24393508	24393513	Lppos	-
17	13010597	13010602	Sod2	+
9	51943618	51943623	Fdx1	-
9	96895748	96895753	Gm10123	+
9	59675105	59675110	Gm49759;Pk	+
9	108947632	108947637	Uqcrc1	+
13	49624264	49624269	Ogn	+
8	123834847	123834852	Rab4a;Gm2(	+
17	46674782	46674787	Klhdc3	-
4	41715400	41715405	Dctn3	-
10	97518007	97518012	Dcn	+
8	72222849	72222854	Fam32a	+
9	44410046	44410051	Rps25	+
5	139338272	139338277	Cox19	-
5	53278070	53278075	Smim20	+
12	84314838	84314843	Ptgr2	+
7	34215972	34215977	Gpi1	-
7	93179255	93179260	Gm15501	-
6	72369316	72369321	Vamp5	-

7	140106316	140106321	Echs1	-
8	123403385	123403390	Tcf25;Gm20:	+
16	17154035	17154040	Ube2l3	-
17	33747553	33747558	Rab11b	-
10	80037321	80037326	Polr2e	-
5	36795332	36795337	Mrfap1	-
19	7066105	7066110	Macrod1	+
2	28934464	28934469	Gm13394;Cf	-
4	155832982	155832987	Aurkaip1	+
10	94220751	94220756	Ndufa12	+
7	127908350	127908355	Bckdk	+
7	111072693	111072698	Eif4g2	-
19	47083509	47083514	Usmg5	-
14	51906133	51906138	Ndrg2	-
2	173779128	173779133	Vapb	+
4	105232276	105232281	Plpp3	+
9	59670716	59670721	Pkm	+
18	11084578	11084583	Gata6	+
1	93716716	93716721	Thap4	-
17	24742470	24742475	Msrb1	+
9	78478502	78478507	Eef1a1	-
2	180713261	180713266	Gid8	+
15	89155702	89155707	Plxnb2	-
3	95741377	95741382	Tars2	-
2	164839477	164839482	Ctsa	+
5	137035691	137035696	Ap1s1	-
7	36118353	36118358	Rpl17-ps9	+
8	11448732	11448737	Col4a2	+
13	54593791	54593796	Cltb	-
4	41135897	41135902	Ube2r2	+
17	56751351	56751356	Nrtn	-
19	5040604	5040609	B4gat1;AC12	+
14	20694661	20694666	Sec24c	+
7	28825387	28825392	Ech1	+
8	71465485	71465490	Mrpl34	+
1	74353309	74353314	Pnkd	+
17	35267279	35267284	H2-D1	+
6	119924278	119924283	Wnk1	-
9	72749713	72749718	Nedd4	+
7	66049930	66049935	Pcsk6;Gm45	+
17	56259389	56259394	Fem1a	+
6	85134102	85134107	Spr	-
9	50637939	50637944	Dlat	-
1	120067666	120067671	Tmem37	-
5	17782546	17782551	Cd36	-

19	4000827	4000832	Nudt8;Gm49	+
6	81962362	81962367	Mrpl19	-
2	108950338	108950343	Gm13910	+
2	143943224	143943229	Dstn	+
1	180178938	180178943	Coq8a	-
3	142303215	142303220	Pdlim5	-
9	123592455	123592460	Sacm1l	+
11	63962923	63962928	Cox10	-
4	91851635	91851640	Gm12669	-
17	14404414	14404419	Smoc2	+
9	50344613	50344618	Rpl10-ps3	-
5	92414347	92414352	Art3	+
9	78479874	78479879	Eef1a1	-
9	69482980	69482985	Anxa2	+
9	71479496	71479501	Polr2m	-
2	108949739	108949744	Gm13910	+
15	102665211	102665216	Atp5g2	-
9	75056684	75056689	Arpp19	+
10	19609495	19609500	Ifngr1	+
8	124889312	124889317	Gnpat	+
10	79977414	79977419	Tmem259	-
2	103456896	103456901	Cat	-
4	107904146	107904151	Cpt2	-
13	74359818	74359823	Lrrc14b	-
15	98127488	98127493	Pfkm	+
7	139579513	139579518	Inpp5a	+
18	60803959	60803964	Cd74	+
10	128911618	128911623	Cd63	+
8	95877737	95877742	Got2	-
12	8922280	8922285	Laptm4a	+
12	84374420	84374425	Entpd5	-
2	30284470	30284475	Dolk	-
11	6271120	6271125	Tmed4	-
1	132032456	132032461	Elk4	+
11	69990291	69990296	Ctdnep1	+
9	50751276	50751281	Hspb2	-
2	174345122	174345127	Gnas	+
12	100206941	100206946	Calm1	+
6	124812867	124812872	Tpi1	-
2	158118869	158118874	Tgm2	-
11	70658405	70658410	Eno3	+
8	25816951	25816956	Ash2l	-
2	25505405	25505410	Fbxw5	+
5	129022379	129022384	Ran	+
10	79675971	79675976	Tpgs1	+

1	75245427	75245432	Dnajb2	+
17	34030914	34030919	Gm20427;Sl	-
3	123111083	123111088	Synpo2	-
11	51791722	51791727	Sar1b	+
17	8283919	8283924	Mpc1	+
5	147869425	147869430	Pomp	+
10	7777852	7777857	Ginm1	-
6	73276869	73276874	Suclg1	+
8	54521415	54521420	Spcs3	-
9	90097422	90097427	Morf4l1	-
2	151561322	151561327	Fkbp1a	+
19	60864133	60864138	Prdx3	-
2	32681952	32681957	Eng	+
19	11774196	11774201	Mrpl16	+
5	123578619	123578624	Clip1	-
15	4147595	4147600	Oxct1	+
5	138162138	138162143	Cops6	+
14	67016142	67016147	Ppp2r2a	-
6	91464622	91464627	Chchd4	-
9	54601151	54601156	Idh3a	+
11	101419166	101419171	Ptges3l;Gm2	-
1	185428009	185428014	Eprs	+
8	94850194	94850199	Coq9	+
5	30955203	30955208	Preb	-
3	101581822	101581827	Atp1a1	-
1	93407850	93407855	Hdlbp	-
8	107043076	107043081	Vps4a	+
2	35307748	35307753	Gsn	+
4	156220627	156220632	Perm1	+
3	87915871	87915876	Hdgf	+
11	94339756	94339761	Ankrd40	+
4	147874958	147874963	Mfn2	-
3	144691434	144691439	Sh3glb1	-
5	121205799	121205804	Rpl6	+
12	36021811	36021816	Tspan13	-
11	21562953	21562958	Mdh1	-
18	67653264	67653269	Psmg2	+
7	140112534	140112539	Echs1	-
19	8927475	8927480	Rom1	-
11	6428376	6428381	H2afv	-
5	124118144	124118149	Arl6ip4	+
3	144691421	144691426	Sh3glb1	-
3	104657233	104657238	Slc16a1	+
19	6986367	6986372	Vegfb	-
7	19696544	19696549	Apoe	-

11	43420325	43420330	Pttg1	-
9	108205879	108205884	Dag1	-
10	72674600	72674605	Zwint	+
7	45458029	45458034	Ftl1	-
9	50344501	50344506	Rpl10-ps3	-
2	32962646	32962651	Rpl12	+
19	8793312	8793317	Polr2g	-
6	39598307	39598312	Ndufb2	+
18	67404854	67404859	Afg3l2	-
7	112110194	112110199	Usp47	+
6	90646394	90646399	Slc41a3	+
19	43664309	43664314	Slc25a28	-
11	48803447	48803452	Rack1	+
3	10208462	10208467	Fabp4	-
14	20391338	20391343	Mrps16	-
17	8296584	8296589	Mpc1	+
5	105797354	105797359	Rps15a-ps5	-
14	55490015	55490020	Dhrs4	+
5	30166670	30166675	Hadhb	+
9	15544990	15544995	Smco4	+
9	59665252	59665257	Pkm	+
3	97690520	97690525	Mir7225;Pde	-
18	84096185	84096190	Zadh2	+
5	22613151	22613156	Rpl17-ps5	-
1	171143640	171143645	Sdhc	-
16	37647639	37647644	Ndufb4	-
7	78775635	78775640	Mrpl46	-
5	147078626	147078631	Polr1d	+
11	87981368	87981373	Dynll2	-
11	31545862	31545867	Ncoa2	+
17	33829665	33829670	Ndufa7	+
17	46548747	46548752	Srf	-
11	51986266	51986271	Ube2b;Gm2	-
19	5457060	5457065	Ccdc85b	-
5	125017414	125017419	Ncor2	-
10	95548811	95548816	Nudt4	-
15	6654108	6654113	Gm7666;Fyb	+
19	5493945	5493950	Cfl1	+
19	36115261	36115266	Ankrd1	-
4	122855839	122855844	Ppt1	+
9	44920661	44920666	Atp5l	-
5	33373045	33373050	Maea	+
10	79688282	79688287	Cdc34	+
8	116983896	116983901	Gcsh	-
2	84765626	84765631	Serping1	-

2	101562299	101562304	B230118H07	-
7	67231670	67231675	Mef2a	-
5	124461714	124461719	Kmt5a	+
5	135789226	135789231	Mdh2	+
7	105558682	105558687	Apbb1	-
9	21921041	21921046	Tmem205	-
10	80828982	80828987	Oaz1	+
13	64366595	64366600	Ctsl	-
12	110666904	110666909	Dync1h1	+
4	155074950	155074955	Rer1	-
11	6433811	6433816	H2afv	-
9	54601093	54601098	Idh3a	+
11	115490592	115490597	Nt5c	-
6	115618972	115618977	Raf1	-
2	90898690	90898695	Ndufs3	-
1	36699561	36699566	Actr1b	-
11	67811489	67811494	Dhrs7c	+
1	172505894	172505899	Tagln2	+
1	167372406	167372411	Mgst3	-
8	11448749	11448754	Col4a2	+
10	13515203	13515208	Fuca2	+
14	32180626	32180631	Timm23	-
6	146650475	146650480	Med21	+
10	60301722	60301727	Psap	+
14	54941978	54941983	Myh6	-
14	32103023	32103028	Oxnad1	+
16	90222788	90222793	Sod1	+
8	34170181	34170186	Saraf	+
1	151363461	151363466	Ivns1abp	+
6	32792470	32792475	Chchd3	-
11	95491994	95491999	Spop	+
10	41530948	41530953	Cd164	+
9	110083775	110083780	Map4	+
6	48702270	48702275	Gimap6	-
5	125386188	125386193	Ubc	-
7	45720799	45720804	Rpl18	+
15	58899676	58899681	Rnf139;Gm4	+
8	107580478	107580483	Psmd7	-
1	59639829	59639834	Sumo1	-
10	18856767	18856772	Perp	+
8	25022934	25022939	Tm2d2	+
13	91863200	91863205	Ckmt2	-
9	22113244	22113249	Elof1	-
5	100547325	100547330	Cops4	+
17	40961762	40961767	Mut	+

12	85133213	85133218	Dlst	+
4	59618356	59618361	Hsdl2	+
10	79711954	79711959	Bsg	+
3	10016265	10016270	Fabp5	+
19	4035466	4035471	Gstp1	-
14	51908647	51908652	Ndrg2	-
15	57871629	57871634	Derl1	-
4	139291441	139291446	Capzb	+
1	24613956	24613961	Gm28437	-
4	147910225	147910230	Plod1	-
8	83995919	83995924	Prkaca	+
7	46855611	46855616	Ldha	+
9	37222799	37222804	Tmem218	+
7	35044377	35044382	Pepd	+
11	3520912	3520917	Smtn	-
2	76709443	76709448	Ttn	-
6	4008232	4008237	Gng11	+
13	55597562	55597567	Tmed9	+
17	78378458	78378463	Fez2	-
9	120131063	120131068	Rpsa	+
1	180166617	180166622	Coq8a	-
2	10059528	10059533	Atp5c1	-
10	97506581	97506586	Dcn	+
17	35149520	35149525	Prrc2a	-
14	54950058	54950063	Myh6	-
10	77598491	77598496	Pttg1ip	+
18	36683590	36683595	Slc35a4	+
2	155847122	155847127	Uqcc1	-
6	33060092	33060097	Chchd3	-
3	116577492	116577497	Lrrc39	+
6	121226497	121226502	Tuba8	+
7	130764458	130764463	Tacc2	+
18	50091273	50091278	Tnfaip8	+
10	80254913	80254918	Ndufs7	+
16	4766189	4766194	Hmox2	+
18	76940528	76940533	Ier3ip1;Hdhd	+
8	72815023	72815028	Large1	-
12	87273727	87273732	Ahsa1	+
5	3236762	3236767	Gm15772	+
11	116245551	116245556	Srp68	-
4	132854319	132854324	Stx12	-
17	6084618	6084623	Gtf2h5	+
12	110858842	110858847	Mpc1-ps	-
10	17845389	17845394	Txlnb	+
5	122460193	122460198	Atp2a2	-

4	45107942	45107947	Tomm5	-
6	116652011	116652016	Depp1	+
5	30122210	30122215	Hadha	-
4	129144551	129144556	Fndc5	+
13	38197610	38197615	Dsp	+
6	124811924	124811929	Tpi1	-
17	33997408	33997413	H2-K1	-
1	180166944	180166949	Coq8a	-
10	77269463	77269468	Pofut2	+
15	84211139	84211144	Samm50	+
7	139578548	139578553	Inpp5a	+
4	141423901	141423906	Hspb7	+
14	29974759	29974764	Selenok	+
10	77269174	77269179	Pofut2	+
11	70977871	70977876	C1qbp	-
18	35617028	35617033	Paip2	+
5	21803262	21803267	Psmc2	+
14	60780271	60780276	C1qtnf9	+
9	119483633	119483638	Scn5a	-
14	63146617	63146622	Fdft1	-
13	59449707	59449712	Agtpbp1	-
18	38258302	38258307	Dele1	+
11	55402047	55402052	Sparc	-
13	112866909	112866914	Plpp1	+
11	59020476	59020481	Obscn	-
13	41310282	41310287	Nedd9	-
11	50200218	50200223	Sqstm1	-
19	4007523	4007528	Ndufv1	-
16	18312723	18312728	Tango2	-
15	89086816	89086821	Trabd	+
12	110666779	110666784	Dync1h1	+
15	98950116	98950121	Tuba1a;Gm4	-
9	120574096	120574101	Rpl14	+
11	21562968	21562973	Mdh1	-
8	13172607	13172612	Lamp1	+
3	122016054	122016059	Arhgap29	+
16	87490586	87490591	Cct8	-
10	128361369	128361374	Cs	+
11	115607269	115607274	Mrps7	+
11	95831668	95831673	Phospho1;Zf	+
2	173033963	173033968	Rbm38	+
4	116991470	116991475	Urod	-
1	164437649	164437654	Atp1b1	-
11	69667374	69667379	Eif4a1	-
4	144927376	144927381	Dhrs3	+

18	34938255	34938260	Hspa9	-
7	30563057	30563062	Psenen	-
8	121596950	121596955	Map1lc3b;Gr	+
15	43527756	43527761	Emc2	+
8	107263100	107263105	Rps18-ps3	-
6	94603797	94603802	Slc25a26	+
12	100114837	100114842	Psmc1	+
19	6391475	6391480	Pygm	+
13	12282617	12282622	Actn2	-
17	56258725	56258730	Fem1a	+
7	30186022	30186027	Cox7a1	+
10	76709929	76709934	Col6a1	-
19	57033575	57033580	Ablim1	-
11	97206845	97206850	Npepps	-
10	81179623	81179628	Eef2	+
13	33918424	33918429	Serpinb6a	-
11	69554742	69554747	Efnb3	-
10	85949598	85949603	Rtcb	-
17	35053080	35053085	Clic1	+
3	90511320	90511325	S100a1	-
18	35616962	35616967	Paip2	+
7	122148573	122148578	Dctn5	+
7	25624714	25624719	Dmac2	+
10	71331064	71331069	Cisd1	-
4	10848940	10848945	Gm12918	+
6	72433014	72433019	Mat2a	-
3	153922587	153922592	Acadm	-
3	36460482	36460487	Anxa5	-
4	91851729	91851734	Gm12671	-
8	95864772	95864777	Got2	-
14	120947304	120947309	Ipo5	+
9	98446437	98446442	Rbp1	+
3	129895772	129895777	Pla2g12a	+
3	94448562	94448567	Mrpl9	+
12	98785706	98785711	Zc3h14	+
5	65388593	65388598	Rpl9	-
5	20886633	20886638	Tmem60	+
15	99409043	99409048	Tmbim6	+
19	8927090	8927095	B3gat3	+
1	172197800	172197805	Pea15a	-
9	108205360	108205365	Dag1	-
1	55071951	55071956	Coq10b	+
19	52991373	52991378	Xpnpep1	-
6	124811905	124811910	Tpi1	-
5	31131187	31131192	Trim54	+

5	116422344	116422349	Hspb8	-
2	75675905	75675910	Nfe2l2	-
2	127298534	127298539	Stard7	+
19	24272384	24272389	Fxn	-
3	121765079	121765084	Abcd3	-
15	85462322	85462327	Atxn10	+
7	16455464	16455469	Tmem160	+
3	102086706	102086711	Casq2	+
10	80403092	80403097	Uqcr11	-
6	142588313	142588318	Abcc9	-
1	180169644	180169649	Coq8a	-
7	101911766	101911771	Lamtor1	+
10	7768277	7768282	Ginm1	-
9	65689575	65689580	Oaz2	+
17	33997387	33997392	H2-K1	-
16	87484010	87484015	Cct8	-
8	124909292	124909297	Egln1	-
16	95720787	95720792	Ets2	+
10	80145297	80145302	Atp5d	+
7	138891577	138891582	Bnip3	-
12	85343345	85343350	Tmed10	-
7	101512740	101512745	Gm45837;Pc	+
13	23739548	23739553	Hist1h1c	+
6	71861033	71861038	Immt	+
11	50093762	50093767	Rnf130	+
8	11835123	11835128	Arhgef7	+
6	85468204	85468209	Cct7	+
2	153009729	153009734	Pdrg1	-
11	40749548	40749553	Ccng1	-
4	49586335	49586340	Tmem246	-
2	155278323	155278328	Pigu	-
2	25998830	25998835	Ubac1	-
3	138455291	138455296	Adh5	+
12	84774761	84774766	Isca2	+
10	81528979	81528984	Gna11	-
3	104793945	104793950	Rhoc	+
10	100099465	100099470	Kitl	+
2	119660409	119660414	Ndufaf1	-
8	119345616	119345621	Hsbp1	+
17	87433819	87433824	Calm2	-
9	22073445	22073450	Ecsit	-
5	115561157	115561162	Rplp0	+
7	38220735	38220740	Plekhf1	-
5	122406143	122406148	Arpc3	+
17	20962710	20962715	Ppp2r1a	+

11	71010281	71010286	Derl2	-
6	71874394	71874399	Immt	+
6	115246029	115246034	Timp4	-
10	40290557	40290562	Amd1	-
3	95128324	95128329	Tmod4	+
11	95026689	95026694	Pdk2	-
1	45375888	45375893	Col5a2	-
4	116637980	116637985	Akr1a1	-
2	90904428	90904433	Ndufs3	-
6	56883778	56883783	Nt5c3	-
3	104794163	104794168	Rhoc	+
9	71483272	71483277	Polr2m	-
12	111828237	111828242	Zfyve21	+
10	123113334	123113339	Usp15	-
3	142302772	142302777	Pdlim5	-
6	142507901	142507906	Ldhb	-
5	30142790	30142795	Hadha	-
7	73775461	73775466	Fam174b	+
7	59999388	59999393	Gm38393;Sr	-
18	76970850	76970855	Hdhd2;Hdhd:	+
1	151349424	151349429	Ivns1abp	+
11	69917436	69917441	Eif5a	-
11	58994285	58994290	Obscn	-
11	31548941	31548946	Ncoa2	+
5	142903530	142903535	Actb	-
6	55347287	55347292	Aqp1	+
6	72360057	72360062	Rnf181	-
2	13582663	13582668	Vim	+
10	128352742	128352747	Cs	+
13	34986161	34986166	Eci2	-
4	9620952	9620957	Asph	-
5	69517017	69517022	Yipf7	-
13	90104451	90104456	Tmem167	+
10	117277909	117277914	Lyz2	-
7	109894239	109894244	Dennd5a	-
4	154158234	154158239	Tprgl	-
4	138313713	138313718	Pink1	-
1	66857492	66857497	Acadl	-
18	65981240	65981245	Lman1	-
5	32492426	32492431	Ppp1cb	+
7	111072403	111072408	Eif4g2	-
3	138451410	138451415	Adh5	+
8	68906367	68906372	Lpl	+
1	63148461	63148466	Ndufs1	-
8	124914091	124914096	Egln1	-

7	34202420	34202425	Gpi1	-
11	21329810	21329815	Ugp2	-
11	68972662	68972667	Rangrf	-
10	88741970	88741975	Arl1	+
1	92641887	92641892	Cops9	-
11	117813336	117813341	Syngr2;Gm2	+
1	131055051	131055056	Mapkapk2	-
10	21999385	21999390	Sgk1	+
10	13515085	13515090	Fuca2	+
13	66900822	66900827	Uqcrb	-
2	164832837	164832842	Neurl2	-
16	11193258	11193263	Rsl1d1	-
5	112688962	112688967	Myo18b	-
17	33824465	33824470	Rps28	-
5	122467779	122467784	Atp2a2	-
16	15888049	15888054	Cebpd	+
4	106662590	106662595	Ttc4	-
4	57950843	57950848	Txn1	-
3	131248428	131248433	Hadh	-
3	79603907	79603912	Etfdh	-
3	90499967	90499972	Chtop	-
13	12280563	12280568	Actn2	-
9	65322611	65322616	Clpx	+
11	21565895	21565900	Mdh1	-
8	27275199	27275204	Eif4ebp1	+
9	21508134	21508139	Tmed1	-
1	45375999	45376004	Col5a2	-
14	54947080	54947085	Myh6	-
1	160197094	160197099	Mrps14	+
2	22588163	22588168	Gm13340	-
2	35314099	35314104	Stom	-
7	25630157	25630162	Bckdha	-
17	66494821	66494826	Rab12	-
2	76707131	76707136	Ttn	-
8	22463140	22463145	Smim19	-
4	59610865	59610870	Hsdl2	+
2	35296438	35296443	Gsn	+
1	92460399	92460404	Ndufa10	-
2	163725902	163725907	Pkig	+
13	38500738	38500743	Txndc5	-
7	132557669	132557674	Oat;Fgfr2	-
2	35180448	35180453	Rab14	-
11	23002231	23002236	Cct4	+
17	25796631	25796636	Metrn	-
19	8772369	8772374	Tmem223	+

17	44279681	44279686	Clic5	+
17	44039353	44039358	Rcan2	+
14	20659289	20659294	Synpo2l	-
2	156873258	156873263	Rab5if	+
19	41526856	41526861	Lcor	+
17	34028421	34028426	Gm20427;Sl	-
1	91276404	91276409	Ube2f	+
5	31191686	31191691	Eif2b4	-
8	46207313	46207318	Slc25a4	-
6	136488802	136488807	Rpl36a-ps3	+
14	31000216	31000221	Spcs1	-
19	36733545	36733550	Ppp1r3c	-
6	125306836	125306841	Ltbr	-
6	35261575	35261580	1810058I24F	+
5	145146441	145146446	Bud31	+
3	152237855	152237860	Nexn	-
19	6906931	6906936	Prdx5	-
14	21447846	21447851	Adk	+
1	171150582	171150587	Sdhc	-
1	118608346	118608351	Clasp1	+
2	84765569	84765574	Serping1	-
9	108327637	108327642	Rhoa	+
9	107592878	107592883	Ifrd2	+
7	30564270	30564275	U2af1l4;Gm4	+
15	51796472	51796477	Eif3h	-
10	80583767	80583772	Abhd17a	-
11	69942833	69942838	Slc2a4	-
1	42898473	42898478	Mrps9	+
8	34170748	34170753	Saraf	+
4	155074559	155074564	Rer1	-
2	39013117	39013122	Arpc5l	+
1	63179308	63179313	Eef1b2	+
17	78378474	78378479	Fez2	-
7	78775527	78775532	Mrpl46	-
7	28305678	28305683	Timm50	-
9	108340009	108340014	Gpx1	+
5	30119912	30119917	Hadha	-
7	19152568	19152573	Snrpd2	+
10	62494503	62494508	Srgn	-
15	76345064	76345069	Cyc1	+
13	74303933	74303938	Pdcd6	-
4	149233823	149233828	Kif1b	-
11	43776238	43776243	Adra1b	-
11	115417897	115417902	Atp5h	-
2	121437762	121437767	Pdia3	+

12	103321505	103321510	Asb2	-
11	106782211	106782216	Ddx5	-
10	127281759	127281764	Dctn2	+
9	123156972	123156977	Clec3b	+
4	42983562	42983567	Vcp	-
17	14403986	14403991	Smoc2	+
8	85352020	85352025	Mylk3	-
5	110281204	110281209	Pxmp2	-
4	117673846	117673851	Eri3	+
4	126232536	126232541	Map7d1	-
14	75847257	75847262	Tpt1	+
9	107614734	107614739	Gnai2	-
10	77597953	77597958	Pttg1ip	+
19	5493988	5493993	Cfl1	+
10	85119923	85119928	Mterf2	-
10	33996597	33996602	Rwdd1	-
1	34307972	34307977	Dst	+
7	25625175	25625180	Dmac2	+
11	20335005	20335010	Steap3	-
9	21767172	21767177	Kank2	-
2	84765397	84765402	Serping1	-
6	87028373	87028378	Nfu1	+
17	53866922	53866927	Mrps36-ps1	-
14	20392112	20392117	Mrps16	-
12	110582960	110582965	Ppp2r5c	+
16	4482817	4482822	Srl	-
11	69990329	69990334	Ctdnep1	+
7	118689019	118689024	Gde1	-
19	37269217	37269222	Ide	-
9	120128553	120128558	Rpsa	+
16	91113760	91113765	Cfap298	-
18	80212209	80212214	Gm16286;Tx	+
2	4927383	4927388	Phyh	+
5	122471187	122471192	Atp2a2	-
2	84765815	84765820	Serping1	-
8	119323519	119323524	Cdh13	+
2	26347047	26347052	Gpsm1	+
17	10206822	10206827	Qk	-
14	79201655	79201660	Vwa8	+
14	54953863	54953868	Myh6	-
17	35384411	35384416	H2-Q4	+
13	24813035	24813040	BC005537	+
17	39846754	39846759	Tns1	+
4	129143477	129143482	Fndc5	+
15	81914889	81914894	Aco2	+

14	105682188	105682193	Gm10076	+
15	4059979	4059984	Oxct1	+
7	30555207	30555212	Hspb6	+
5	143563344	143563349	Fam220a;Fa	+
10	33157112	33157117	Trdn	+
16	38362906	38362911	Popdc2	+
7	100486283	100486288	Ucp3	+
6	86525047	86525052	Pcbp1	-
7	5097761	5097766	Epn1	+
1	171288857	171288862	Ufc1	-
7	97407741	97407746	Ndufc2	+
11	95680500	95680505	Phb	+
3	69072242	69072247	Kpna4	-
19	43515706	43515711	Got1	-
1	75216573	75216578	Tuba4a	-
9	54599627	54599632	Idh3a	+
1	125595462	125595467	Slc35f5	+
2	163721269	163721274	Pkig	+
7	137468018	137468023	Glrx3	+
6	83343660	83343665	Mob1a	+
6	55345883	55345888	Aqp1	+
17	34028983	34028988	Gm20427;Sl	-
19	6951783	6951788	Bad	+
2	144595280	144595285	Smim26	+
7	111072132	111072137	Eif4g2	-
6	17288689	17288694	Cav2	+
7	105742306	105742311	Ilk	+
17	56614381	56614386	Lonp1	-
5	122868697	122868702	Rnf34	+
7	78780590	78780595	Mrpl46	-
3	129878861	129878866	Pla2g12a	+
19	5842798	5842803	Neat1	-
6	127087833	127087838	Tigar	-
16	18301097	18301102	Tango2	-
11	101288615	101288620	Becn1	-
8	126941212	126941217	Tomm20	-
11	32296766	32296771	Hba-a2	+
2	84765748	84765753	Serping1	-
5	134620854	134620859	Eif4h	-
8	27053845	27053850	Plpbp	+
2	84453841	84453846	Tfpi	-
12	10395286	10395291	Rdh14	+
6	24603562	24603567	Lmod2	+
7	110813573	110813578	Rnf141	-
5	36471442	36471447	Grpel1	+

17	27632308	27632313	Rps10;RPS1	-
9	57532418	57532423	Cox5a	+
11	120104653	120104658	Slc38a10	-
17	12974714	12974719	Wtap	-
10	128401547	128401552	Nabp2	-
11	115514235	115514240	Jpt1	-
15	37000082	37000087	Zfp706	-
7	46891142	46891147	Tsg101	-
11	70235348	70235353	0610010K14I	-
9	44742197	44742202	Arcn1	-
9	72748735	72748740	Nedd4	+
13	59756003	59756008	Isca1	-
7	126980045	126980050	Cdipt	+
1	36531540	36531545	Ankrd23;Gm	-
1	85908090	85908095	Itm2c	+
17	24849785	24849790	Fahd1	-
8	84970279	84970284	Prdx2	+
16	36964366	36964371	Fbxo40	-
8	107580454	107580459	Psmd7	-
8	84842128	84842133	Calr	-
7	143513872	143513877	Nap1l4	-
18	53406539	53406544	Ppic	-
14	55899223	55899228	Sdr39u1	-
3	97690642	97690647	Pde4dip	-
17	24437278	24437283	Eci1	+
5	97884985	97884990	Antxr2	-
2	127298853	127298858	Stard7	+
4	107897166	107897171	0610037L13I	+
1	51302761	51302766	Cavin2	+
3	115712669	115712674	S1pr1	-
4	116639952	116639957	Akr1a1	-
9	121857531	121857536	Higd1a	-
7	100485340	100485345	Ucp3	+
7	45883008	45883013	Kdelr1	+
19	47864622	47864627	Gsto1	+
16	37654272	37654277	Ndufb4	-
10	56968515	56968520	Gja1	-
6	87845785	87845790	Cnbp	-
6	125683542	125683547	Vwf	+
12	84756781	84756786	Npc2	-
8	23241371	23241376	Golga7	-
5	30623049	30623054	Kcnk3	+
7	31054774	31054779	Fxyd1	-
6	34304075	34304080	Akr1b3	-
1	63152824	63152829	Ndufs1	-

2	156147914	156147919	Rbm39	-
13	58392199	58392204	Hnrnpk	-
9	50597209	50597214	Sdhd	-
10	56390344	56390349	Gja1	+
6	72848330	72848335	Kcmf1	-
7	105742284	105742289	Ilk	+
7	4794137	4794142	Rpl28	+
1	55089053	55089058	Hspe1	+
13	24752013	24752018	Gmnn	-
5	30172704	30172709	Hadhb	+
5	33248441	33248446	Ctbp1	-
5	104085091	104085096	Sparcl1	-
4	15918992	15918997	Decr1	-
5	90773322	90773327	Pf4	+
12	8498612	8498617	Rhob	-
10	128368953	128368958	Coq10a	-
5	124571066	124571071	Eif2b1	-
7	28350992	28350997	Rps16	+
13	75849820	75849825	Glrx	+
7	108940698	108940703	Eif3f	+
6	57689415	57689420	Pyurf	-
17	24639525	24639530	Slc9a3r2	-
9	59665943	59665948	Pkm	+
7	118118970	118118975	Arl6ip1	-
4	99970096	99970101	Pgm1	+
7	80100839	80100844	Idh2	-
8	70757151	70757156	Rab3a	+
19	29021937	29021942	Ak3	-
10	128359501	128359506	Cs	+
15	98128176	98128181	Pfkm	+
4	126232648	126232653	Map7d1	-
4	132553092	132553097	Dnajc8	+
17	72940995	72941000	Lbh	+
2	136881429	136881434	Mkks;AL731	-
17	8297340	8297345	Mpc1	+
11	120579232	120579237	Arhgdia	-
6	147060144	147060149	Mrps35	+
17	66089468	66089473	Ndufv2	-
5	77087555	77087560	Hopx	-
5	117091923	117091928	Suds3	-
7	111070816	111070821	Eif4g2	-
13	23739498	23739503	Hist1h1c	+
5	30135069	30135074	Hadha	-
4	119279030	119279035	Ybx1	-
4	55527451	55527456	Klf4	-

6	48701876	48701881	Gimap6	-
2	61785502	61785507	Psmd14	+
17	24639361	24639366	Slc9a3r2	-
13	24818047	24818052	Acot13	-
9	21354140	21354145	Slc44a2	+
11	106073656	106073661	Taco1	+
2	10056101	10056106	Atp5c1	-
7	126490475	126490480	Tufm	+
2	130281089	130281094	Idh3b	-
2	118881204	118881209	Ivd	+
11	58998312	58998317	Obscn	-
14	20500507	20500512	Ppp3cb	-
11	59183941	59183946	Guk1	-
11	5704481	5704486	Mrps24	-
15	102635327	102635332	Rpl39-ps	+
6	88826973	88826978	Mgll	+
2	32681636	32681641	Eng	+
10	81179082	81179087	Eef2	+
2	76705149	76705154	Ttn	-
9	20895989	20895994	Eif3g	-
18	25133305	25133310	Fhod3	+
5	31622399	31622404	Mrpl33;Gm4:	+
4	57370939	57370944	Ptpn3	-
14	60903419	60903424	Mipep	+
7	99479274	99479279	Rps3	-
1	80267104	80267109	Cul3	-
8	11514121	11514126	Cars2	-
6	55346890	55346895	Aqp1	+
17	35267029	35267034	H2-D1	+
17	24722758	24722763	Ndufb10	-
2	32633440	32633445	Ak1	+
11	52122015	52122020	Ppp2ca	+
9	7932304	7932309	Yap1	-
11	77468538	77468543	Coro6	+
11	5967393	5967398	Ykt6	+
5	30126602	30126607	Hadha	-
4	137320606	137320611	Cdc42	-
2	120091086	120091091	Ehd4	-
1	135768698	135768703	Phlda3	+
19	40292709	40292714	Pdlim1	-
4	127247373	127247378	Smim12	+
13	30545274	30545279	Uqcrfs1	-
11	115608236	115608241	Mif4gd	-
11	97781811	97781816	Rpl23	-
4	107904190	107904195	Cpt2	-

16	35303703	35303708	Adcy5	+
18	35590662	35590667	Matr3	+
10	29698980	29698985	Gm10275	-
1	118609021	118609026	Clasp1	+
8	70700011	70700016	Jund	+
14	36875384	36875389	Ccser2	-
15	3279620	3279625	Selenop	+
5	115240833	115240838	Pop5	+
8	125418293	125418298	Sipa1l2	-
2	173034009	173034014	Rbm38	+
10	128076538	128076543	Ptges3	+
19	29017432	29017437	Cdc37l1	+
9	70008224	70008229	Bnip2	+
15	79028789	79028794	H1f0	+
2	32573469	32573474	Dpm2	+
5	115298684	115298689	Dynll1	-
2	155389543	155389548	Trp53inp2	+
5	125387003	125387008	Ubc	-
18	38840428	38840433	Fgf1	-
18	77778549	77778554	Atp5a1	+
4	141422558	141422563	Hspb7	+
4	154896568	154896573	Fam213b	-
2	156389787	156389792	2900097C17	-
1	10034929	10034934	Cops5	-
19	24261949	24261954	Fxn	-
7	93179360	93179365	Gm15501	-
11	43775388	43775393	Adra1b	-
8	106884509	106884514	Chtf8;Chtf8	-
6	51464166	51464171	Hnrnpa2b1	-
7	28974149	28974154	Eif3k	-
4	136880856	136880861	C1qb	-
4	15919923	15919928	Decr1	-
4	117154739	117154744	Rps8	-
5	88659755	88659760	Grsf1	-
5	77349419	77349424	Igfbp7	-
15	83154452	83154457	Cyb5r3	-
17	56997513	56997518	Alkbh7	+
5	115801650	115801655	Rab35	-
1	63166312	63166317	Ndufs1	-
9	65676630	65676635	Oaz2	+
11	115607218	115607223	Mrps7	+
9	4308810	4308815	Aasdhppt	-
11	88211045	88211050	Mrps23	+
2	150618318	150618323	Acss1	-
3	144597547	144597552	Selenof	+

4	134927166	134927171	Rsrp1	+
8	23244742	23244747	Golga7	-
15	34283586	34283591	Laptm4b	+
7	64400186	64400191	Mcee	+
5	17782931	17782936	Cd36	-
1	182467869	182467874	Capn2	-
11	60199896	60199901	Srebf1	-
4	141424709	141424714	Hspb7	+
12	100872072	100872077	Dglucy	+
7	84615510	84615515	Zfand6	-
7	105557678	105557683	Smpd1	+
16	37835921	37835926	Fstl1	+
5	147078932	147078937	Polr1d	+
14	8166257	8166262	Pdhb	-
3	96528250	96528255	Hfe2	+
5	121445002	121445007	Erp29	-
2	155389479	155389484	Trp53inp2	+
11	30880272	30880277	Psme4	+
5	91626225	91626230	Parm1	+
1	90612688	90612693	Cops8	+
16	11193710	11193715	Rsl1d1	-
10	80665430	80665435	Mknk2	-
17	26839113	26839118	Nkx2-5	-
13	34987526	34987531	Eci2	-
7	64412014	64412019	Mcee	+
4	139291514	139291519	Capzb	+
1	63157171	63157176	Ndufs1	-
13	64185731	64185736	Habp4	+
13	115088525	115088530	Pelo;Itga1;Gr	-
8	13256473	13256478	Dcun1d2	-
7	120641696	120641701	Uqcrc2	+
16	4481443	4481448	Srl	-
15	76171360	76171365	Plec	-
1	97706759	97706764	Ppip5k2	-
2	38590736	38590741	Psmb7	-
1	191782870	191782875	Lpgat1	+
12	54180179	54180184	Egln3	-
4	34566628	34566633	Akirin2	+
5	129989377	129989382	Gusb	-
17	26212966	26212971	Fam234a	-
9	54604579	54604584	Idh3a	+
17	49993878	49993883	Rftn1	-
2	91135380	91135385	Mybpc3	+
15	82329731	82329736	Naga	-
11	52246221	52246226	Skp1a	+

9	78478718	78478723	Eef1a1	-
8	124909746	124909751	Egln1	-
1	178448786	178448791	Efcab2	+
18	75243305	75243310	Dym	+
9	53582037	53582042	Acat1	-
5	112692341	112692346	Myo18b	-
4	40937169	40937174	Bag1	-
15	83547248	83547253	Mcat	-
4	134898338	134898343	Tmem50a	-
15	25973157	25973162	Retreg1	+
11	69396536	69396541	Naa38	+
15	82041738	82041743	Snu13	-
15	4154354	4154359	Oxct1	+
10	62269296	62269301	Hk1	-
13	98313114	98313119	Btf3	-
15	82042357	82042362	Snu13	-
12	8499271	8499276	Rhob	-
15	76905934	76905939	Rpl8	+
8	46536013	46536018	Acsl1	+
9	7189240	7189245	Dcun1d5	+
8	70534950	70534955	Fkbp8	+
2	162934597	162934602	Srsf6	+
16	4937071	4937076	Mgrn1	+
11	107167487	107167492	Nol11	-
2	50288998	50289003	Mmadhc	-
15	4154859	4154864	Oxct1	+
8	107581026	107581031	Psmd7	-
14	29027505	29027510	Lrtm1	+
1	75217255	75217260	Tuba4a	-
1	171288760	171288765	Ufc1	-
5	123579416	123579421	Clip1	-
8	79274251	79274256	Mmaa	-
5	41625413	41625418	Rab28	-
6	29461692	29461697	Flnc	+
15	74995244	74995249	Ly6a	-
17	86832691	86832696	Epas1	+
6	6558394	6558399	Sem1	-
16	36043956	36043961	Fam162a	-
13	59756449	59756454	Isca1	-
5	116409039	116409044	Hspb8	-
6	71872855	71872860	Immt	+
16	36046417	36046422	Fam162a	-
2	152313868	152313873	Tbc1d20	+
11	86585240	86585245	Vmp1	-
7	121076695	121076700	Mettl9	+

11	70011248	70011253	Acadvl	-
11	94659947	94659952	Mrpl27	+
13	14625573	14625578	Psma2	+
5	125386410	125386415	Ubc	-
10	117280739	117280744	Lyz2	-
11	40680718	40680723	Mat2b	-
15	5120975	5120980	Gm10250	-
7	15949250	15949255	Ehd2	-
11	115415752	115415757	Atp5h	-
14	24495840	24495845	Rps24	+
9	121712683	121712688	Ss18l2	+
10	77617395	77617400	Sumo3;Gm4!	+
17	20965558	20965563	Ppp2r1a	+
13	24820825	24820830	Acot13	-
4	132530813	132530818	Atpif1	-
11	31545960	31545965	Ncoa2	+
11	116132897	116132902	Mrpl38	-
5	100547513	100547518	Cops4	+
2	36044407	36044412	Ndufa8	-
7	19093559	19093564	Dmpk	+
10	7630387	7630392	Pcmt1	-
8	122614369	122614374	Trappc2l;Gm	+
15	3279343	3279348	Selenop	+
9	64180541	64180546	Snapc5	+
15	76722533	76722538	C030006K11	-
1	10032413	10032418	Cops5	-
19	45793120	45793125	Kcnip2	-
12	55490645	55490650	Nfkbia	-
3	97690420	97690425	Pde4dip	-
8	70010559	70010564	Gm7730	-
7	138882502	138882507	Ppp2r2d	+
11	88339737	88339742	Msi2	-
2	125301335	125301340	Fbn1	-
7	80711654	80711659	Iqgap1	-
16	36052348	36052353	Fam162a	-
3	96561083	96561088	Txnip	+
4	129715600	129715605	Khdrbs1	-
9	71479294	71479299	Polr2m	-
19	53588424	53588429	Nutf2-ps1	-
1	24613914	24613919	Gm28437	-
18	3511389	3511394	Bambi	+
4	140514937	140514942	Arhgef10l	-
6	95474666	95474671	Suclg2	-
7	99345700	99345705	Serpinh1	-
11	48803871	48803876	Rack1	+

14	73366418	73366423	Itm2b	-
17	78920076	78920081	Cebpzos	+
7	30555271	30555276	Hspb6	+
8	85365086	85365091	Mylk3	-
11	6172699	6172704	Rps15a-ps6;	-
10	59988112	59988117	Anapc16	-
17	70996762	70996767	Myl12a;Myl1:	-
5	140759771	140759776	Gna12	-
19	6398043	6398048	Pygm	+
7	44981589	44981594	Prmt1	-
3	95032776	95032781	Psmd4	-
15	51786577	51786582	Eif3h	-
4	107904479	107904484	Cpt2	-
5	136965918	136965923	Fis1	+
2	103021483	103021488	Pdhx	-
3	105955896	105955901	Atp5f1	-
7	30641374	30641379	Rbm42;Gm2	-
6	29376050	29376055	Calu	+
8	13146855	13146860	Cul4a	+
4	135214209	135214214	Clic4	-
12	76319743	76319748	Mthfd1	+
9	105077398	105077403	Mrpl3	+
9	31149757	31149762	Aplp2	-
1	34308386	34308391	Dst	+
3	153930326	153930331	Acadm	-
19	57033371	57033376	Ablim1	-
5	65449945	65449950	Gm43552;Sr	-
4	45804396	45804401	Aldh1b1	+
2	84672032	84672037	Gm28635;Tr	-
3	132672142	132672147	Aimp1	-
4	119281605	119281610	Ybx1	-
5	129128037	129128042	Ran	-
8	41340767	41340772	Asah1	-
9	108336993	108336998	Rhoa	+
14	8166281	8166286	Pdhb	-
6	147070704	147070709	Mrps35	+
15	74959207	74959212	Ly6e	+
5	135783372	135783377	Mdh2	+
15	96687887	96687892	Slc38a2	-
8	124908875	124908880	Egln1	-
19	34327248	34327253	Fas	+
8	123893657	123893662	Acta1	-
13	3561877	3561882	Gdi2	+
1	58901119	58901124	Trak2	-
6	100867603	100867608	Ppp4r2	+

2	130565257	130565262	Mrps26	+
19	4116096	4116101	Aip	-
5	115110910	115110915	Acads	-
3	37405223	37405228	Nudt6	-
10	78282446	78282451	Agpat3	-
16	91563076	91563081	Ifngr2	+
2	167690348	167690353	Cebpb	+
8	41340675	41340680	Asah1	-
4	47312157	47312162	Col15a1	+
1	172280006	172280011	Atp1a2	-
19	3911612	3911617	Ndufs8	-
13	93045969	93045974	Cmya5	-
18	36402794	36402799	Cystm1	+
1	153907885	153907890	Glul	+
7	3704402	3704407	Rps9	+
6	72370195	72370200	Vamp5	-
4	57371536	57371541	Gm12537	-
8	64592679	64592684	Cpe	-
2	130281690	130281695	Idh3b	-
5	137747165	137747170	Tsc22d4	+
18	34949072	34949077	Hspa9	-
15	76249522	76249527	Grina	+
17	84710618	84710623	Lrpprc	-
15	77915674	77915679	Txn2	-
7	73776831	73776836	Fam174b	+
2	25583620	25583625	Rabl6	-
5	103989870	103989875	Hsd17b11	-
1	180168386	180168391	Coq8a	-
3	58102873	58102878	Pfn2	-
17	48423320	48423325	Apobec2	-
7	126489827	126489832	Tufm	+
7	25757686	25757691	Axl	-
14	45341312	45341317	Psmc6	+
3	144188957	144188962	Lmo4	-
17	46891519	46891524	Tbcc	+
16	84827967	84827972	Atp5j	-
15	76070432	76070437	Puf60	-
11	115430757	115430762	Kctd2	+
9	104116230	104116235	Acad11	+
13	58126554	58126559	Hnrnpa0	-
18	80211563	80211568	Gm16286;Tx	+
2	91132681	91132686	Mybpc3	+
5	30622236	30622241	Kcnk3	+
17	34934845	34934850	Neu1	+
11	55395860	55395865	Sparc	-

10	71228454	71228459	Tfam	-
11	73176610	73176615	Emc6	-
17	24895896	24895901	Mrps34	+
16	92312967	92312972	Smim11	+
5	36471216	36471221	Grpel1	+
16	56015674	56015679	Pcnp	-
13	11553658	11553663	Ryr2	-
14	73362560	73362565	Itm2b	-
7	31054396	31054401	Fxyd1	-
11	68973190	68973195	Rangrf	-
4	48673036	48673041	Cavin4	+
14	65530841	65530846	Elp3	-
13	56639046	56639051	Tgfbi	+
1	135768872	135768877	Phlda3	+
6	136612161	136612166	Plbd1	-
8	84837738	84837743	Rad23a	-
3	88927053	88927058	Dap3	-
12	78848450	78848455	Atp6v1d	-
19	36116828	36116833	Ankrd1	-
3	57646434	57646439	Commd2	-
10	57516073	57516078	Serinc1	-
7	27307883	27307888	Ltbp4	-
5	122324060	122324065	Pptc7	+
11	54911173	54911178	Tnip1	-
17	26217297	26217302	Fam234a	-
15	81907486	81907491	Aco2	+
8	95713724	95713729	Ndrg4	+
11	62552860	62552865	Ubb	+
2	83664058	83664063	Zc3h15	+
9	59678976	59678981	Pkm	+
17	24742597	24742602	Msrb1	+
12	36014943	36014948	Tspan13	-
11	70238200	70238205	Gm21988;Rr	-
6	87015590	87015595	Nfu1	+
11	104584002	104584007	Myl4	+
10	33476004	33476009	Trdn	+
8	19493428	19493433	Rpl19-ps11	+
6	56882849	56882854	Nt5c3	-
19	36119392	36119397	Ankrd1	-
12	85342870	85342875	Tmed10	-
18	36742518	36742523	Ndufa2	-
5	122459574	122459579	Atp2a2	-
2	180162496	180162501	Osbpl2	+
2	119660343	119660348	Ndufaf1	-
11	102436483	102436488	Grn	+

18	35251183	35251188	Ctnna1	+
3	79605002	79605007	Etfdh	-
2	155242743	155242748	Dynlrb1	+
6	113515537	113515542	Emc3	-
17	14403538	14403543	Smoc2	+
5	115242646	115242651	Rnf10	-
11	73181343	73181348	Tax1bp3	+
6	124712079	124712084	Emg1	-
2	158116677	158116682	Tgm2	-
7	105640766	105640771	Timm10b;Gn	+
8	85260970	85260975	Vps35	-
2	34623796	34623801	Mapkap1	+
19	10629406	10629411	Ddb1	+
7	142377450	142377455	Gm49369;Ct	-
15	88864864	88864869	Pim3	+
12	32839238	32839243	Nampt	+
11	44454634	44454639	Ublcp1	-
9	21067881	21067886	Gm49373;Gı	-
6	124711881	124711886	Emg1	-
3	95661727	95661732	Mcl1	+
2	101562291	101562296	B230118H07	-
6	136612263	136612268	Plbd1	-
7	126795650	126795655	Aldoa	-
8	84844436	84844441	Calr	-
5	110785414	110785419	Ulk1	-
1	9942093	9942098	Snhg6	-
5	24592737	24592742	Smarcd3	-
2	32678990	32678995	Eng	+
11	98800076	98800081	Msl1	+
3	89405929	89405934	Flad1	-
3	94884985	94884990	Psmb4	-
17	34028577	34028582	Gm20427;Sl	-
8	23148698	23148703	Ank1	+
5	5782340	5782345	Gm15459	-
14	122159154	122159159	Tm9sf2	+
9	59678772	59678777	Pkm	+
3	8802359	8802364	Mrps28	-
5	17828960	17828965	Cd36	-
6	24604195	24604200	Lmod2	+
15	76354110	76354115	Maf1	+
3	146838807	146838812	Gm10288	-
18	50091738	50091743	Tnfaip8	+
7	128546511	128546516	Bag3	+
2	84769885	84769890	Serping1	-
11	97711176	97711181	Psmb3	+

11	116215070	116215075	Ten1	+
18	36742625	36742630	Ndufa2	-
11	69919296	69919301	Eif5a	-
11	101278217	101278222	Coa3	-
2	31026294	31026299	Fnbp1	-
2	132529245	132529250	Gpcpd1	-
19	45005678	45005683	Mrpl43	-
4	42980539	42980544	Vcp	-
11	88831396	88831401	Akap1	-
10	97506616	97506621	Dcn	+

Chromosomal Locations for m6A sites in Control vs METTL3-KO ("METTL3-Dependent") Conditions

chrom	chromStart	chromEnd	name	strand
4	141425129	141425134	Hspb7	+
9	61913346	61913351	Rplp1	-
4	141424038	141424043	Hspb7	+
2	114050358	114050363	Actc1	-
2	91136389	91136394	Mybpc3	+
16	38377985	38377990	Popdc2	+
9	54714965	54714970	Dnaja4	+
15	73751868	73751873	Ptp4a3	+
8	46209213	46209218	Slc25a4	-
19	43500087	43500092	Got1	-
11	75765445	75765450	Ywhae	+
12	103325174	103325179	Asb2	-
13	30540719	30540724	Uqcrfs1	-
4	141424507	141424512	Hspb7	+
11	101278328	101278333	Coa3	-
7	130936347	130936352	Htra1	+
3	105943859	105943864	Atp5f1	-
11	69089879	69089884	Vamp2	+
11	95680297	95680302	Phb	+
17	71857154	71857159	Clip4	+
8	106573028	106573033	Gm10073	-
4	141421765	141421770	Hspb7	+
13	23739975	23739980	Hist1h1c	+
2	91058071	91058076	Psmc3	+
7	30554699	30554704	Hspb6	+
18	34938666	34938671	Hspa9	-
2	34776151	34776156	Hspa5	+
17	33691668	33691673	March2	-
17	24896132	24896137	Mrps34	+
11	5803462	5803467	Pgam2	-
13	74331352	74331357	Sdha	-
7	46084084	46084089	Nomo1	+
11	87981368	87981373	Dynll2	-
12	111454472	111454477	Tnfaip2	+
10	18856804	18856809	Perp	+
15	102281179	102281184	Mfsd5	+
18	80295678	80295683	Kcng2	-
6	133106233	133106238	Smim10l1	+
7	99479877	99479882	Rps3	-
3	32577553	32577558	Mfn1	+
6	119353487	119353492	Adipor2	-

6	11907364	11907369	Ndufa4	-
11	84834498	84834503	Ggnbp2	-
6	24597906	24597911	Lmod2	+
10	75937707	75937712	Chchd10	+
1	161241474	161241479	Prdx6	-
10	91116969	91116974	Slc25a3	-
17	40961216	40961221	Mut	+
9	64178225	64178230	Rpl4	+
9	50634768	50634773	Dlat	-
4	141424514	141424519	Hspb7	+
9	50650835	50650840	Dlat	-
14	15357994	15357999	Lrrc3b	-
3	51250204	51250209	Noct	+
7	3617390	3617395	Ndufa3	+
3	97690341	97690346	Pde4dip	-
11	115607060	115607065	Mrps7	+
9	107651733	107651738	Slc38a3	-
5	116422364	116422369	Hspb8	-
2	75640906	75640911	Rps6-ps4	+
4	140966574	140966579	Sdhb	+
15	4155247	4155252	Oxct1	+
5	24565648	24565653	Abcf2	-
4	95049990	95049995	Jun	-
9	108945379	108945384	Uqcrc1	+
4	15919046	15919051	Decr1	-
13	23684286	23684291	Hist1h2bc	+
15	3317842	3317847	Ghr	-
11	5803481	5803486	Pgam2	-
15	98932252	98932257	Tuba1b;Gm4	-
5	53278155	53278160	Smim20	+
1	55082066	55082071	Hspd1	-
7	126796251	126796256	Aldoa	-
11	58998005	58998010	Obscn	-
15	73751531	73751536	Ptp4a3	+
8	94854436	94854441	Coq9	+
7	19414273	19414278	Ckm	+
15	98932321	98932326	Tuba1b;Gm4	-
7	30555236	30555241	Hspb6	+
10	81182257	81182262	Eef2	+
10	78162557	78162562	D10Jhu81e	-
14	32180396	32180401	Timm23	-
3	96560814	96560819	Txnip	+
7	28831402	28831407	Ech1	+
6	91487815	91487820	Tmem43	+
10	94220900	94220905	Ndufa12	+

4	59618159	59618164	Hsdl2	+
12	110692143	110692148	Hsp90aa1	-
2	75640130	75640135	Rps6-ps4	+
5	36795332	36795337	Mrfap1	-
9	106431478	106431483	Rpl29	+
15	58783672	58783677	Tmem65	-
11	120347680	120347685	Actg1	-
13	74322455	74322460	Sdha	-
17	46251740	46251745	Yipf3	+
2	35303216	35303221	Gsn	+
1	131055019	131055024	Mapkapk2	-
4	141423940	141423945	Hspb7	+
6	50565006	50565011	Cycs	-
9	50751442	50751447	Hspb2	-
8	46207415	46207420	Slc25a4	-
10	94220923	94220928	Ndufa12	+
9	120130181	120130186	Rpsa	+
5	135790357	135790362	Mdh2	+
2	75641871	75641876	Rps6-ps4	+
5	122453702	122453707	Atp2a2	-
2	120090485	120090490	Ehd4	-
14	66987507	66987512	Bnip3l	-
11	5803118	5803123	Pgam2	-
2	30402188	30402193	Crat	-
9	59679142	59679147	Pkm	+
13	62082430	62082435	Gm48228	-
17	35832753	35832758	Flot1	+
6	24604137	24604142	Lmod2	+
15	96688142	96688147	Slc38a2	-
16	4480735	4480740	Srl	-
11	54979335	54979340	Anxa6	-
7	142385581	142385586	Ctsd	-
14	26414180	26414185	Slmap	-
11	51991434	51991439	Ube2b;Gm26	-
7	16454187	16454192	Tmem160	+
1	92464473	92464478	Ndufa10	-
10	53343816	53343821	Pln	+
17	24471108	24471113	Pgp	+
1	75215932	75215937	Tuba4a	-
9	50634999	50635004	Dlat	-
1	66831041	66831046	Acadl	-
12	110858095	110858100	Wdr20	-
4	138313926	138313931	Pink1	-
18	6201087	6201092	Kif5b	-
8	104628299	104628304	Rrad	-

2	164837069	164837074	Ctsa	+
11	70644687	70644692	Slc25a11	-
1	120113424	120113429	Dbi	-
6	91464983	91464988	Chchd4	-
15	3279126	3279131	Selenop	+
1	85907770	85907775	Itm2c	+
4	116074979	116074984	Uqcrh	-
1	75216370	75216375	Tuba4a	-
19	4008598	4008603	Ndufv1	-
18	11085026	11085031	Gata6	+
8	46207597	46207602	Slc25a4	-
2	120091003	120091008	Ehd4	-
11	4702033	4702038	Uqcr10	-
5	121205799	121205804	Rpl6	+
11	83404749	83404754	Ap2b1	+
15	5120696	5120701	Gm10250	-
15	77017648	77017653	Mb	-
2	76709578	76709583	Ttn	-
15	79029400	79029405	H1f0	+
9	66949423	66949428	Rps27l	+
3	27244678	27244683	Nceh1	+
2	28933490	28933495	Cfap77	-
6	128436665	128436670	Fkbp4	-
7	67231384	67231389	Mef2a	-
9	56136517	56136522	Tspan3	-
11	87979739	87979744	Dynll2	-
16	16261719	16261724	Pkp2	+
1	128038864	128038869	Rpl28-ps1	+
1	93408090	93408095	Hdlbp	-
2	150830631	150830636	Pygb	+
14	54614437	54614442	Psmb5	-
8	11453733	11453738	Rab20	-
8	128723001	128723006	Itgb1	+
8	69895251	69895256	Ndufa13;Yjef	-
4	41191068	41191073	Ube2r2	+
5	139338171	139338176	Cox19	-
2	25222709	25222714	Tubb4b	-
11	94339707	94339712	Ankrd40	+
7	19419480	19419485	Ckm	+
6	82725712	82725717	Hk2	-
6	72369316	72369321	Vamp5	-
19	60864186	60864191	Prdx3	-
8	46534688	46534693	Acsl1	+
17	48423014	48423019	Apobec2	-
1	75360680	75360685	Des	+

7	116104294	116104299	1110004F10I	+
7	143513646	143513651	Nap1l4	-
6	87843201	87843206	Cnbp	-
5	115801668	115801673	Rab35	-
14	51905681	51905686	Ndrg2	-
4	73943030	73943035	2310002L09I	-
7	111074739	111074744	Eif4g2	-
4	133965170	133965175	Hmgn2	-
8	84977268	84977273	Junb	-
10	7768277	7768282	Ginm1	-
9	107301517	107301522	Cish	+
1	10025053	10025058	Cops5	-
3	36091421	36091426	Gm43079;Ac	+
11	58994348	58994353	Obscn	-
7	105555535	105555540	Smpd1	+
10	57519379	57519384	Serinc1	-
7	80100832	80100837	Idh2	-
9	50635730	50635735	Dlat	-
1	45348600	45348605	Col3a1	+
5	125388240	125388245	Ubc	-
15	77015614	77015619	Mb	-
6	50565537	50565542	Cycs	-
19	21281468	21281473	Zfand5	+
2	118882595	118882600	Ivd	+
11	69943357	69943362	Slc2a4	-
10	75937400	75937405	Chchd10	+
5	122454000	122454005	Atp2a2	-
2	121458279	121458284	Serf2;Hypk	+
11	116849992	116849997	Srsf2	-
19	11774575	11774580	Mrpl16	+
5	135012047	135012052	Abhd11	+
10	91116941	91116946	Slc25a3	-
11	120105511	120105516	Slc38a10	-
17	15494415	15494420	Psmb1	-
2	156311892	156311897	Scand1	-
5	122457615	122457620	Atp2a2	-
15	76171360	76171365	Plec	-
9	106215001	106215006	Twf2	+
4	141423954	141423959	Hspb7	+
16	91928927	91928932	Atp5o	-
5	115110836	115110841	Acads	-
7	4519441	4519446	Tnni3	-
19	43500477	43500482	Got1	-
4	139485998	139486003	Ubr4	+
1	131055942	131055947	Mapkapk2	-

14	34171004	34171009	Rpl23a-ps3	+
9	71478949	71478954	Polr2m	-
8	107439405	107439410	Rps26-ps1	-
4	40936603	40936608	Bag1	-
14	66085306	66085311	Ephx2	-
13	100736199	100736204	Mrps36	-
13	119335848	119335853	Nnt;Nnt	-
6	71214140	71214145	Smyd1	-
11	115607177	115607182	Mrps7	+
15	83376579	83376584	Pacsin2	-
7	19531265	19531270	Ppp1r37	-
7	28832133	28832138	Ech1	+
10	128361119	128361124	Cs	+
18	50091372	50091377	Tnfaip8	+
11	97328751	97328756	Mrpl45	+
10	91119699	91119704	Slc25a3	-
1	75360640	75360645	Des	+
15	76354110	76354115	Maf1	+
9	110980901	110980906	Lrrc2	+
10	121410608	121410613	Rassf3	-
1	156051669	156051674	Tor1aip2	+
6	87843867	87843872	Cnbp	-
13	34073913	34073918	Bphl	+
9	108207584	108207589	Dag1	-
10	128084036	128084041	Atp5b	+
17	66495127	66495132	Rab12	-
2	119660550	119660555	Ndufaf1	-
2	30402525	30402530	Crat	-
16	38374240	38374245	Popdc2	+
11	52246333	52246338	Skp1a	+
13	91859304	91859309	Ckmt2	-
7	99479917	99479922	Rps3	-
2	32634736	32634741	Ak1	+
19	9984850	9984855	Fth1	+
5	122453877	122453882	Atp2a2	-
11	69943029	69943034	Slc2a4	-
2	163336411	163336416	Jph2	-
7	102110908	102110913	Art1	+
9	54715890	54715895	Dnaja4	+
14	76507175	76507180	Tsc22d1	+
4	116691870	116691875	Prdx1	+
7	138882494	138882499	Ppp2r2d	+
4	141619610	141619615	Slc25a34	-
4	136880220	136880225	C1qb	-
8	94854315	94854320	Coq9	+

19	4004760	4004765	Gm49405;Dc	+
4	40279261	40279266	Ndufb6	-
11	40748844	40748849	Ccng1	-
9	120014279	120014284	Xirp1;Cx3cr1	-
2	91131440	91131445	Mybpc3	+
1	82747080	82747085	Mff	+
18	80295585	80295590	Kcng2	-
8	19493428	19493433	Rpl19-ps11	+
11	6349132	6349137	Ogdh	+
9	51943451	51943456	Fdx1	-
10	80292830	80292835	Rps15	+
1	125393595	125393600	Actr3	-
4	117268134	117268139	Tmem53	+
7	100640925	100640930	Rab6a	+
19	37221670	37221675	March5	+
18	20031557	20031562	Dsc2	-
12	113144059	113144064	Crip2	+
11	93963282	93963287	Gm20390;Nr	-
7	112355026	112355031	Mical2	+
2	150619570	150619575	Acss1	-
16	56035122	56035127	Trmt10c	-
4	130315312	130315317	Fabp3	+
7	34202390	34202395	Gpi1	-
3	153922655	153922660	Acadm	-
10	88474255	88474260	Chpt1	-
10	80253758	80253763	Ndufs7	+
2	120090357	120090362	Ehd4	-
9	40804355	40804360	Hspa8	+
3	152185108	152185113	Dnajb4	-
3	10204546	10204551	Fabp4	-
11	58932644	58932649	Rnf187	-
6	99877743	99877748	Gpr27	-
7	101822878	101822883	Inppl1	-
1	54997132	54997137	Sf3b1	-
9	108945374	108945379	Uqcrc1	+
4	117894554	117894559	Ipo13	-
1	79781244	79781249	Mrpl44	+
12	8938698	8938703	Laptm4a	+
2	148871930	148871935	Cst3	-
1	151364171	151364176	Ivns1abp	+
1	171129573	171129578	Sdhc	-
7	126491819	126491824	Atxn2l	-
6	113515900	113515905	Emc3	-
6	29375948	29375953	Calu	+
11	88831100	88831105	Akap1	-

11	62552860	62552865	Ubb	+
2	75642504	75642509	Rps6-ps4	+
5	143564057	143564062	Fam220a;Fa	+
14	65975723	65975728	Clu	+
11	70978120	70978125	C1qbp	-
9	50605129	50605134	Timm8b	+
6	87843418	87843423	Cnbp	-
17	24849732	24849737	Fahd1	-
7	142376452	142376457	Gm49369;Ct	-
7	15920329	15920334	Selenow	-
10	13515234	13515239	Fuca2	+
11	100320663	100320668	Eif1	+
11	59012523	59012528	Obscn	-
7	102111009	102111014	Art1	+
2	119599785	119599790	1700020I14F	+
10	128362002	128362007	Cs	+
1	172066583	172066588	Ncstn	-
16	90342577	90342582	Gm49708	-
14	34529216	34529221	Ldb3	-
2	118880874	118880879	Ivd	+
4	129143351	129143356	Fndc5	+
8	56592032	56592037	Fbxo8	+
4	141424029	141424034	Hspb7	+
4	107904146	107904151	Cpt2	-
4	133965327	133965332	Hmgn2	-
14	21838537	21838542	Vdac2	+
7	121076424	121076429	Mettl9	+
15	25973385	25973390	Retreg1	+
15	89054889	89054894	Mov10l1	+
7	105555345	105555350	Smpd1	+
11	32296506	32296511	Hba-a2	+
19	36731855	36731860	Ppp1r3c	-
11	100321486	100321491	Eif1	+
16	91563412	91563417	Ifngr2	+
7	43457690	43457695	Etfb;Gm4523	+
14	25699858	25699863	Ppif	+
7	142385466	142385471	Ctsd	-
7	73776650	73776655	Fam174b	+
6	5485196	5485201	Pdk4	-
5	133475418	133475423	Gm10051	+
17	83502584	83502589	Cox7a2l	-
7	28829812	28829817	Ech1	+
9	101105990	101105995	Ppp2r3a	-
5	115561170	115561175	Rplp0	+
13	91863254	91863259	Ckmt2	-

2	150618544	150618549	Acss1	-
9	50751551	50751556	Hspb2	-
11	40753911	40753916	Ccng1	-
17	56112141	56112146	Plin5	-
1	36530324	36530329	Ankrd23;Gm	-
1	191306929	191306934	Nenf	-
9	54604340	54604345	Idh3a	+
10	80145560	80145565	Atp5d	+
4	108044789	108044794	Podn;Scp2	-
19	44555036	44555041	Ndufb8	-
11	70978082	70978087	C1qbp	-
11	69089174	69089179	Vamp2	+
14	66086277	66086282	Ephx2	-
15	77915598	77915603	Txn2	-
4	150897351	150897356	Park7	-
2	121457163	121457168	Serf2;Hypk	+
2	163336833	163336838	Jph2	-
1	16677172	16677177	Tmem70	+
2	91121110	91121115	Mybpc3	+
10	78167395	78167400	D10Jhu81e	-
11	116173531	116173536	Acox1	-
11	20335901	20335906	Gm12033	-
11	4702029	4702034	Uqcr10	-
5	114250538	114250543	Acacb	+
1	182279604	182279609	Degs1	-
4	134927051	134927056	Rsrp1	+
3	96196912	96196917	Bola1	-
10	62269684	62269689	Hk1	-
12	16536598	16536603	Lpin1	-
1	43131738	43131743	Fhl2	-
9	50596665	50596670	Sdhd	-
7	89851753	89851758	Me3	+
3	32751507	32751512	Ndufb5	+
6	82726428	82726433	Hk2	-
2	91134787	91134792	Mybpc3	+
4	127247461	127247466	Smim12	+
17	81385809	81385814	Slc8a1	-
11	106786101	106786106	Ddx5	-
1	63143797	63143802	Ndufs1	-
2	71274901	71274906	Slc25a12	-
5	36967141	36967146	Wfs1	-
11	120543473	120543478	Mcrip1	-
11	78073511	78073516	Eral1	-
19	9984564	9984569	Fth1	+
15	77015886	77015891	Mb	-

4	108816807	108816812	Btf3l4	-
18	77780238	77780243	Atp5a1	+
9	107614849	107614854	Gnai2	-
3	116506890	116506895	Rtca	-
3	133378035	133378040	Ppa2	+
7	48835497	48835502	Csrp3	-
7	100486309	100486314	Ucp3	+
14	26414237	26414242	Slmap	-
6	117180977	117180982	Cxcl12	+
17	25863847	25863852	Mcrip2	-
13	74322410	74322415	Sdha	-
17	70996886	70996891	Myl12a;Myl1:	-
6	124811098	124811103	Tpi1	-
13	64364014	64364019	Ctsl	-
11	69554465	69554470	Efnb3	-
3	67474871	67474876	Gfm1	+
7	81344987	81344992	Rps17	-
12	21391315	21391320	Ywhaq	-
11	59012211	59012216	Obscn	-
4	148617257	148617262	Tardbp	-
9	67028073	67028078	Tpm1	-
15	76646174	76646179	Cyhr1	-
11	70644613	70644618	Slc25a11	-
17	34957040	34957045	Hspa1b	-
2	104114127	104114132	Cd59a	+
5	122454094	122454099	Atp2a2	-
10	80796413	80796418	Plekhj1	-
2	75642147	75642152	Rps6-ps4	+
9	21139785	21139790	Cdc37	-
2	131178180	131178185	Cenpb;Spef1	-
17	45568419	45568424	Hsp90ab1	-
11	49678959	49678964	Gm12191;Cr	-
7	138882694	138882699	Ppp2r2d	+
9	50635274	50635279	Dlat	-
11	106074588	106074593	Taco1	+
7	78775479	78775484	Mrpl46	-
17	53506333	53506338	Rab5a	+
11	49262006	49262011	Mgat1	+
10	60301804	60301809	Psap	+
16	18407834	18407839	Comt	-
5	112315157	112315162	Tpst2	+
7	28257556	28257561	Dyrk1b	-
18	75230159	75230164	Dym	+
2	24974354	24974359	Mrpl41	-
1	90214641	90214646	Ackr3	+

13	59756842	59756847	Isca1	-
7	30190529	30190534	Capns1	-
19	6908055	6908060	Prdx5	-
11	31665612	31665617	Bod1	-
7	80095811	80095816	Idh2	-
11	48800488	48800493	Rack1	+
11	5803327	5803332	Pgam2	-
10	53345804	53345809	Pln	+
19	6389239	6389244	Pygm	+
15	96688481	96688486	Slc38a2	-
10	21368455	21368460	Hbs1l	+
17	30594030	30594035	Glo1;AC1744	-
17	13010601	13010606	Sod2	+
3	131245212	131245217	Hadh	-
7	28256720	28256725	Dyrk1b	-
11	48803885	48803890	Rack1	+
9	108948961	108948966	Uqcrc1	+
19	4000827	4000832	Nudt8;Gm49	+
13	46650232	46650237	Cap2	+
2	26593411	26593416	Agpat2	-
8	22569369	22569374	Slc20a2	+
9	55464798	55464803	Etfa	-
3	32520645	32520650	Zfp639	+
5	129758097	129758102	Nipsnap2	+
2	103022049	103022054	Pdhx	-
8	84886920	84886925	Gcdh	-
2	75640167	75640172	Rps6-ps4	+
4	95050202	95050207	Jun	-
1	24615144	24615149	Gm28661	-
1	40855370	40855375	Tmem182	+
9	67031462	67031467	Tpm1	-
9	108502041	108502046	Usp19	+
11	120488447	120488452	Mrpl12	+
9	21008731	21008736	Mrpl4	+
11	21557484	21557489	Mdh1	-
7	105641250	105641255	Timm10b;Gn	+
9	108207725	108207730	Dag1	-
7	28377441	28377446	Zfp36	-
7	108940698	108940703	Eif3f	+
9	67036100	67036105	Tpm1	-
4	149744698	149744703	Slc25a33	-
8	94394924	94394929	Herpud1	+
3	24333302	24333307	Gm7536	+
17	6084618	6084623	Gtf2h5	+
3	105943051	105943056	Atp5f1	-

6	72153925	72153930	St3gal5	+
16	35304293	35304298	Adcy5	+
14	63152413	63152418	Fdft1	-
5	135909320	135909325	Ywhag	-
2	50279923	50279928	Mmadhc	-
11	31548501	31548506	Ncoa2	+
8	93972763	93972768	Amfr	-
17	79849969	79849974	Atl2	-
11	115607463	115607468	Mrps7	+
2	10059528	10059533	Atp5c1	-
12	101969524	101969529	Ndufb1-ps	-
19	36115006	36115011	Ankrd1	-
8	46535986	46535991	Acsl1	+
3	95989317	95989322	Plekho1	-
7	118525837	118525842	Coq7	-
2	181148635	181148640	Eef1a2	-
1	171129503	171129508	Sdhc	-
9	50754525	50754530	Cryab	+
3	7444855	7444860	Pkia	+
18	60776453	60776458	Rps14	+
1	34444517	34444522	Imp4	+
4	134529201	134529206	Mtfr1l	-
5	24581662	24581667	Gm10221	-
6	122456934	122456939	Gm8430	+
6	115619148	115619153	Raf1	-
11	5704481	5704486	Mrps24	-
15	83376154	83376159	Pacsin2	-
9	21589069	21589074	Carm1	+
11	74679598	74679603	Pafah1b1	-
11	54983313	54983318	Anxa6	-
18	36402794	36402799	Cystm1	+
15	73751445	73751450	Ptp4a3	+
5	114250503	114250508	Acacb	+
1	155098150	155098155	Ier5	-
9	79755494	79755499	Cox7a2	-
1	43196656	43196661	Fhl2	-
7	142383426	142383431	Ctsd	-
9	110767992	110767997	Myl3	+
3	154786521	154786526	Tnni3k	-
5	30623049	30623054	Kcnk3	+
14	54614451	54614456	Psmb5	-
15	102707090	102707095	Calcoco1	-
10	17845278	17845283	Txlnb	+
14	75846231	75846236	Tpt1	+
13	98310086	98310091	Btf3	-

1	171238817	171238822	Ndufs2	-
11	23649087	23649092	Pex13	-
7	45720711	45720716	Rpl18	+
10	17845090	17845095	Txlnb	+
7	105558317	105558322	Smpd1	+
5	33247887	33247892	Ctbp1	-
5	116409282	116409287	Hspb8	-
14	31371545	31371550	Capn7	+
1	135852031	135852036	Tnnt2	+
7	105557772	105557777	Smpd1	+
4	59617905	59617910	Hsdl2	+
8	83573871	83573876	Tecr	-
2	121548659	121548664	Frmd5	-
11	97048872	97048877	Mrpl10	+
12	31331697	31331702	Dld	-
10	80254913	80254918	Ndufs7	+
9	67033889	67033894	Tpm1	-
4	49514382	49514387	Mrpl50	-
15	35931994	35931999	Cox6c	-
3	27244750	27244755	Nceh1	+
12	31332239	31332244	Dld	-
10	17724595	17724600	Cited2	+
4	107907993	107907998	Cpt2	-
2	112363173	112363178	Emc4	-
7	45916529	45916534	Tmem143	+
13	55188824	55188829	Zfp346	+
2	91125289	91125294	Mybpc3	+
16	90226301	90226306	Sod1	+
9	120960161	120960166	Ctnnb1	+
9	67029688	67029693	Tpm1	-
9	56938238	56938243	Imp3	+
7	16915988	16915993	Calm3	-
15	83547458	83547463	Mcat	-
11	54869861	54869866	Hint1	+
9	123307308	123307313	Scp2-ps2	-
7	45709869	45709874	Dbp	+
3	152237258	152237263	Nexn	-
10	86732054	86732059	Fabp3-ps1	-
10	53344420	53344425	Pln	+
5	24444176	24444181	Fastk	-
4	57371432	57371437	Gm12537	-
7	25625016	25625021	Dmac2	+
6	124934689	124934694	Mlf2	+
14	31210153	31210158	Tnnc1	+
7	30185964	30185969	Cox7a1	+

2	130281035	130281040	Idh3b	-
3	146505496	146505501	Gng5	+
17	39846540	39846545	Tns1	+
1	37417240	37417245	Coa5	-
5	104079497	104079502	Sparcl1	-
4	15930951	15930956	Decr1	-
4	129143842	129143847	Fndc5	+
11	120488133	120488138	Mrpl12	+
11	75513041	75513046	Rilp	+
5	45434664	45434669	Qdpr	-
16	84834817	84834822	Atp5j	-
17	25832241	25832246	Stub1	-
19	6982675	6982680	Vegfb	-
7	4522405	4522410	Tnni3	-
17	56608970	56608975	2410015M20	-
9	108205879	108205884	Dag1	-
17	56258803	56258808	Fem1a	+
8	15133407	15133412	Myom2	+
1	75487289	75487294	Obsl1	-
8	119410803	119410808	Mlycd	+
2	19659534	19659539	Otud1	+
1	135309181	135309186	Timm17a	-
2	75641481	75641486	Rps6-ps4	+
3	105959187	105959192	Atp5f1	-
7	99153934	99153939	Dgat2	-
9	26975585	26975590	Acad8	-
8	102865269	102865274	Gm8730	-
7	127908065	127908070	Bckdk	+
3	94884376	94884381	Psmb4	-
7	126780263	126780268	Ypel3	+
9	119485645	119485650	Scn5a	-
2	114050538	114050543	Actc1	-
18	80296061	80296066	Kcng2	-
1	171129461	171129466	Sdhc	-
11	69994608	69994613	Gabarap	+
11	5803175	5803180	Pgam2	-
2	144595227	144595232	Smim26	+
7	128546419	128546424	Bag3	+
3	96527221	96527226	Hfe2	+
1	151364226	151364231	Ivns1abp	+
8	107580497	107580502	Psmd7	-
2	76704549	76704554	Ttn	-
7	78775469	78775474	Mrpl46	-
12	4236530	4236535	Ptrhd1	+
9	108207186	108207191	Dag1	-

3	105942863	105942868	Atp5f1	-
6	91487251	91487256	Tmem43	+
17	29251551	29251556	Ppil1	-
11	31548373	31548378	Ncoa2	+
14	57826846	57826851	Mrpl57	+
18	80295690	80295695	Kcng2	-
2	28934362	28934367	Gm13394;Cf	-
9	101107455	101107460	Ppp2r3a	-
3	130729275	130729280	Rpl34	-
14	63142660	63142665	Ctsb	+
2	164833328	164833333	Neurl2	-
13	54593723	54593728	Cltb	-
13	12275079	12275084	Actn2	-
16	91926467	91926472	Atp5o;Atp5o	-
7	138891366	138891371	Bnip3	-
5	30119912	30119917	Hadha	-
16	31948010	31948015	0610012G03	-
1	4782639	4782644	Mrpl15	-
12	112649114	112649119	Siva1	+
12	110898480	110898485	Rps19-ps6;T	+
5	5782117	5782122	Gm15459	-
12	84439637	84439642	Aldh6a1	-
17	29041958	29041963	Srsf3	+
19	40243412	40243417	Pdlim1	-
1	165775167	165775172	Creg1	+
15	27594285	27594290	Ank	+
2	131936863	131936868	Prnp;Prn	+
10	61692997	61693002	Sar1a	+
11	54979463	54979468	Anxa6	-
2	25223942	25223947	Tubb4b	-
11	21557413	21557418	Mdh1	-
2	174341916	174341921	Gnas	+
3	105954024	105954029	Atp5f1	-
10	59396007	59396012	Gm10273	+
10	53606254	53606259	Asf1a	+
5	122467779	122467784	Atp2a2	-
7	80390613	80390618	Furin	-
3	97690553	97690558	Mir7225;Pde	-
11	115498185	115498190	Jpt1	-
17	24433154	24433159	Eci1	+
11	70978090	70978095	C1qbp	-
11	88209906	88209911	Mrps23	+
9	21008658	21008663	Mrpl4	+
8	72321142	72321147	Klf2	+
7	141473293	141473298	Polr2l	-

2	26347047	26347052	Gpsm1	+
8	46207419	46207424	Slc25a4	-
9	55461681	55461686	Etfa	-
18	77782672	77782677	Atp5a1	+
14	65979678	65979683	Clu	+
3	153922727	153922732	Acadm	-
9	67028027	67028032	Tpm1	-
11	115606951	115606956	Mrps7	+
13	74407255	74407260	Ftl1-ps1	+
7	128010902	128010907	Trim72	+
6	72369175	72369180	Vamp5	-
1	134755538	134755543	Ppp1r12b	-
4	141619941	141619946	Slc25a34	-
15	99724719	99724724	Gpd1	+
17	56259702	56259707	Fem1a	+
13	49195919	49195924	Ninj1	+
18	6788786	6788791	AC131796.2;	+
15	31594518	31594523	Cct5	-
2	108950624	108950629	Gm13910	+
5	30623745	30623750	Kcnk3	+
10	128362346	128362351	Cs	+
2	150831161	150831166	Pygb	+
17	66083473	66083478	Ndufv2	-
18	20603799	20603804	Dsg2	+
6	72842697	72842702	Kcmf1	-
7	93179255	93179260	Gm15501	-
3	95740105	95740110	Tars2	-
10	128086126	128086131	Atp5b	+
8	119348436	119348441	Hsbp1	+
6	17666678	17666683	Capza2	+
3	79812780	79812785	Tmem144	-
3	32746463	32746468	Ndufb5	+
9	105077160	105077165	Mrpl3	+
13	54591021	54591026	Higd2a	+
1	172274584	172274589	Atp1a2	-
18	16589956	16589961	Cdh2	-
11	59775707	59775712	Mprip	+
5	73633855	73633860	Sgcb	-
2	75640658	75640663	Rps6-ps4	+
13	41276198	41276203	Smim13	+
4	129593031	129593036	Eif3i	-
7	128442379	128442384	Tial1	-
2	122152952	122152957	B2m	+
15	93391082	93391087	Zcrb1	-
2	132530532	132530537	Gpcpd1	-

3	37714916	37714921	Rps23-ps1	+
8	25022686	25022691	Tm2d2	+
15	82350323	82350328	Ndufa6	-
4	129715612	129715617	Khdrbs1	-
19	42151497	42151502	Marveld1	+
4	21910650	21910655	Coq3	+
8	22463140	22463145	Smim19	-
14	73365756	73365761	Itm2b	-
10	53345584	53345589	Pln	+
7	45125817	45125822	Rpl13a	-
12	84372526	84372531	Coq6	+
3	37714761	37714766	Rps23-ps1	+
11	30649346	30649351	Acyp2	-
13	49196039	49196044	Ninj1	+
1	171143640	171143645	Sdhc	-
3	142302597	142302602	Pdlim5	-
17	26506329	26506334	Dusp1	-
11	40750409	40750414	Ccng1	-
13	64364076	64364081	Ctsl	-
5	36795382	36795387	Mrfap1	-
4	141425045	141425050	Hspb7	+
9	108337220	108337225	Rhoa	+
1	82751773	82751778	Mff	+
2	38640131	38640136	Psmb7	-
17	56993458	56993463	Clpp	+
13	38500469	38500474	Txndc5	-
11	58207065	58207070	Igtp;Irgm2	+
5	104089671	104089676	Sparcl1	-
5	122489267	122489272	Atp2a2	-
19	6062250	6062255	Znhit2	+
17	35895848	35895853	2310061I04F	-
2	30402541	30402546	Crat	-
9	48651924	48651929	Gm5617	-
8	109672102	109672107	Ist1	-
7	3704382	3704387	Rps9	+
2	71274871	71274876	Slc25a12	-
5	115242515	115242520	Rnf10	-
19	45005388	45005393	Mrpl43	-
13	11554141	11554146	Ryr2	-
6	72153735	72153740	St3gal5	+
8	84834260	84834265	Gadd45gip1	+
5	134620118	134620123	Eif4h	-
10	71228425	71228430	Tfam	-
2	91056683	91056688	Psmc3	+
10	80255995	80256000	Ndufs7	+

1	4776440	4776445	Mrpl15	-
10	69290454	69290459	Rhobtb1	+
12	84016942	84016947	Acot1	+
5	24803457	24803462	Rheb	-
5	53278083	53278088	Smim20	+
7	140106316	140106321	Echs1	-
4	116074961	116074966	Uqcrh	-
11	95680487	95680492	Phb	+
9	94520088	94520093	1190002N15	-
18	74798377	74798382	Acaa2	+
19	6985445	6985450	Vegfb	-
1	171143588	171143593	Sdhc	-
9	50344613	50344618	Rpl10-ps3	-
7	45125871	45125876	Rpl13a	-
9	103353114	103353119	Cdv3	-
11	115607138	115607143	Mrps7	+
10	67545289	67545294	Ado	-
14	46776204	46776209	Cnih1	-
3	88057797	88057802	Naxe	-
1	161241258	161241263	Prdx6	-
17	66495433	66495438	Rab12	-
11	69916880	69916885	Eif5a	-
5	30126602	30126607	Hadha	-
11	40748780	40748785	Ccng1	-
16	55966608	55966613	Rpl24;Gm28	+
17	10209236	10209241	Qk	-
8	23149643	23149648	Ank1	+
11	54908631	54908636	Gpx3	+
8	68906209	68906214	Lpl	+
11	69943370	69943375	Slc2a4	-
16	36963879	36963884	Fbxo40	-
3	34061846	34061851	Fxr1	+
6	121226549	121226554	Tuba8	+
5	122174549	122174554	Ppp1cc	+
3	123016557	123016562	Myoz2	-
10	128353189	128353194	Cs	+
8	22561016	22561021	Slc20a2	+
4	141421794	141421799	Hspb7	+
14	21845380	21845385	Vdac2	+
17	56259256	56259261	Fem1a	+
10	45875474	45875479	Gpx4-ps2	+
2	75641445	75641450	Rps6-ps4	+
2	90904428	90904433	Ndufs3	-
12	8499361	8499366	Rhob	-
15	38692140	38692145	Atp6v1c1	+

9	107300836	107300841	Cish	+
13	38198436	38198441	Dsp	+
14	54947069	54947074	Myh6	-
19	43500034	43500039	Got1	-
6	120795703	120795708	Atp6v1e1	-
10	128361530	128361535	Cs	+
7	19414984	19414989	Ckm	+
11	6270505	6270510	Tmed4	-
9	107593163	107593168	Ifrd2	-
8	124344932	124344937	Galnt2;Gm2(	+
16	96122105	96122110	Hmgn1	-
6	47525798	47525803	Cul1	+
9	75031802	75031807	Fam214a	+
5	73313617	73313622	Ociad1	+
8	124909995	124910000	Egln1	-
16	31457015	31457020	Bdh1	+
14	25699843	25699848	Ppif	+
7	44978909	44978914	Prmt1	-
18	35799953	35799958	AC141471.2;	+
10	53344985	53344990	Pln	+
11	55499731	55499736	G3bp1	+
16	38362462	38362467	Popdc2	+
2	30404529	30404534	Crat	-
18	65266167	65266172	Alpk2	-
6	99877916	99877921	Tpt1-ps3	-
15	74749257	74749262	Lynx1	-
9	78479913	78479918	Eef1a1	-
2	172509543	172509548	Gm14303	-
1	183276812	183276817	Brox	-
17	39847231	39847236	Gm42418	+
7	122090902	122090907	Ndufab1	-
11	70728423	70728428	Kif1c	+
6	24604638	24604643	Lmod2	+
1	75367206	75367211	Des	+
4	33246012	33246017	Pnrc1	-
7	48830483	48830488	Csrp3	-
17	81386508	81386513	Slc8a1	-
2	166945905	166945910	Cse1l	+
17	81386700	81386705	Slc8a1	-
7	45338889	45338894	Hrc	+
2	114051991	114051996	Actc1	-
11	69667562	69667567	Eif4a1	-
19	32466376	32466381	Rpl9-ps6	-
16	20692461	20692466	Eif4g1	+
9	52087719	52087724	Rdx	+

4	49513568	49513573	Mrpl50	-
12	105040431	105040436	Glrx5	+
3	104653505	104653510	Slc16a1	+
13	49733932	49733937	Iars	+
4	129593343	129593348	Eif3i	-
5	17782692	17782697	Cd36	-
15	101274514	101274519	Nr4a1	+
2	155389096	155389101	Trp53inp2	+
17	44278175	44278180	Clic5	+
15	4094068	4094073	Oxct1	+
13	54590981	54590986	Higd2a	+
12	76580839	76580844	Sptb	-
5	140443189	140443194	Eif3b	+
11	59012712	59012717	Obscn	-
9	108948563	108948568	Uqcrc1	+
3	104656450	104656455	Slc16a1	+
17	86998774	86998779	Rhoq	+
11	6356586	6356591	Ogdh	+
2	35180419	35180424	Rab14	-
3	126843740	126843745	Camk2d	+
12	12912093	12912098	Rpl36-ps3;G	+
9	40803350	40803355	Hspa8	+
17	26839290	26839295	Nkx2-5	-
16	23108611	23108616	Eif4a2	+
11	50234900	50234905	Mgat4b	+
4	148146870	148146875	Fbxo6	-
19	24476499	24476504	Fam122a;Pip	-
2	150830428	150830433	Pygb	+
8	46535862	46535867	Acsl1	+
11	31549425	31549430	Ncoa2	+
12	17283981	17283986	Pdia6	+
11	31549379	31549384	Ncoa2	+
17	24849785	24849790	Fahd1	-
10	24598321	24598326	Ccn2	+
17	13010597	13010602	Sod2	+
8	27275199	27275204	Eif4ebp1	+
9	53583488	53583493	Acat1	-
12	103408189	103408194	Ddx24	-
18	77757716	77757721	Haus1	-
6	71124814	71124819	Rpl34-ps1	+
17	29137008	29137013	Rpl35a-ps3	-
6	71874514	71874519	Immt	+
4	43664076	43664081	Gm12481	+
11	82937831	82937836	Unc45b	+
19	8978049	8978054	Eef1g	+

4	139291421	139291426	Capzb	+
10	42502347	42502352	Snx3	+
10	17724712	17724717	Cited2	+
5	120485377	120485382	Plbd2	-
8	72815371	72815376	Large1	-
10	81182435	81182440	Eef2	+
7	34207891	34207896	Gpi1	-
4	149744515	149744520	Slc25a33	-
8	105524892	105524897	Atp6v0d1	-
11	54870284	54870289	Hint1	+
14	65975738	65975743	Clu	+
16	38369581	38369586	Popdc2	+
7	13033941	13033946	Chmp2a	-
3	90231810	90231815	Jtb	+
7	114267150	114267155	Psma1	-
17	24849815	24849820	Fahd1	-
1	120113414	120113419	Dbi	-
8	120081396	120081401	Zdhhc7	-
10	17724171	17724176	Cited2	+
7	120649396	120649401	Uqcrc2	+
5	139395564	139395569	Gpr146	+
4	138439872	138439877	Mul1	+
3	102145901	102145906	Casq2	+
2	34776430	34776435	Hspa5	+
18	35806816	35806821	AC141471.2;	+
15	76345872	76345877	Cyc1	+
18	50091706	50091711	Tnfaip8	+
15	41866209	41866214	Abra	-
3	51407349	51407354	Ndufc1	-
18	75009780	75009785	BC031181	+
19	5423823	5423828	Drap1	-
1	90612934	90612939	Cops8	+
9	36779867	36779872	Ei24	-
7	142009027	142009032	Mob2	-
14	61221743	61221748	Sgcg	-
1	52862265	52862270	Hibch	+
10	13515257	13515262	Fuca2	+
8	4259961	4259966	Timm44	-
9	110982178	110982183	Lrrc2	+
11	48803871	48803876	Rack1	+
11	69917436	69917441	Eif5a	-
8	124889365	124889370	Gnpat	+
14	34338866	34338871	Glud1	+
18	36791178	36791183	Hars2	+
2	155247887	155247892	Dynlrb1	+

5	5782957	5782962	Gm15459	-
1	171238831	171238836	Ndufs2	-
1	151351889	151351894	Ivns1abp	+
2	90853094	90853099	Mtch2	+
19	45005461	45005466	Mrpl43	-
16	36046417	36046422	Fam162a	-
8	69894555	69894560	Ndufa13;Yjef	-
11	40748734	40748739	Ccng1	-
11	52120949	52120954	Ppp2ca	+
1	135301792	135301797	Timm17a	-
13	66901484	66901489	Uqcrb	-
3	142302876	142302881	Pdlim5	-
8	46535552	46535557	Acsl1	+
17	83502289	83502294	Cox7a2l	-
11	60864626	60864631	Tmem11	-
13	91859902	91859907	Ckmt2	-
11	21559851	21559856	Mdh1	-
2	163466783	163466788	Fitm2	-
1	80267122	80267127	Cul3	-
5	97884985	97884990	Antxr2	-
13	3565172	3565177	Gdi2	+
7	100485971	100485976	Ucp3	+
7	51747706	51747711	Gm7336	+
5	125017252	125017257	Ncor2	-
14	55480089	55480094	Dhrs4	+
10	53344195	53344200	Pln	+
14	45331925	45331930	Psmc6	+
17	13017595	13017600	Sod2	+
8	64611380	64611385	Cpe	-
1	160203208	160203213	Cacybp	-
11	3917328	3917333	Tcn2	-
5	31136194	31136199	Trim54	+
17	45568399	45568404	Hsp90ab1	-
6	124934704	124934709	Mlf2	+
13	119357652	119357657	Nnt;Nnt	-
8	95870897	95870902	Got2	-
7	30624563	30624568	Cox6b1	-
6	11905223	11905228	Ndufa4	-
5	122471178	122471183	Atp2a2	-
15	51786655	51786660	Eif3h	-
13	30541201	30541206	Uqcrfs1	-
8	83611090	83611095	Dnajb1	+
5	130188690	130188695	Gm15920;Ra	+
2	157556793	157556798	Blcap	-
7	130391758	130391763	Ate1;Fgfr2	-

2	174330317	174330322	Gnas	+
5	122453780	122453785	Atp2a2	-
7	46855344	46855349	Ldha	+
5	30119779	30119784	Hadha	-
19	43500007	43500012	Got1	-
4	123935955	123935960	Rragc	+
16	4480415	4480420	Srl	-
9	120013970	120013975	Xirp1;Cx3cr1	-
3	19691758	19691763	Trim55	+
19	32673216	32673221	Atad1	-
11	102054378	102054383	Cd300lg	+
5	125386547	125386552	Ubc	-
6	52556447	52556452	Hibadh	-
15	4101804	4101809	Oxct1	+
17	6037981	6037986	Synj2	+
9	108947454	108947459	Uqcrc1	+
6	71881034	71881039	Ptcd3	-
7	127908431	127908436	Bckdk	+
4	49585594	49585599	Tmem246	-
7	128546608	128546613	Bag3	+
1	193272335	193272340	G0s2	-
1	178475879	178475884	Efcab2	+
12	110858860	110858865	Mpc1-ps	-
3	101581754	101581759	Atp1a1	-
9	100982580	100982585	Pccb	-
3	97690110	97690115	Pde4dip	-
5	83279093	83279098	Tecrl	-
7	126795429	126795434	Aldoa	-
3	86139061	86139066	Rps3a1	-
2	155846986	155846991	Uqcc1	-
16	23110136	23110141	Eif4a2	+
11	74674203	74674208	Pafah1b1	-
18	25132934	25132939	Fhod3	+
11	65688663	65688668	Map2k4	-
14	45348682	45348687	Psmc6	+
3	153910797	153910802	Rabggtb	-
13	86046687	86046692	Cox7c	-
6	142588401	142588406	Abcc9	-
13	38198377	38198382	Dsp	+
13	11566970	11566975	Ryr2	-
2	38643387	38643392	Psmb7	-
18	35253606	35253611	Ctnna1	+
13	58127183	58127188	Hnrnpa0	-
3	95988981	95988986	Plekho1	-
7	105558682	105558687	Apbb1	-

3	142302757	142302762	Pdlim5	-
3	152186742	152186747	Dnajb4	-
1	75215791	75215796	Tuba4a	-
9	78480032	78480037	Eef1a1	-
2	136881429	136881434	Mkks;AL731	-
3	101581829	101581834	Atp1a1	-
1	75362903	75362908	Des	+
5	129881267	129881272	Chchd2;Phk	-
16	4937143	4937148	Mgrn1	+
11	70010243	70010248	Acadvl	-
13	58177569	58177574	Ubqln1	-
1	63143896	63143901	Ndufs1	-
3	79604980	79604985	Etfdh	-
5	122454288	122454293	Atp2a2	-
1	90215214	90215219	Ackr3	+
2	121551157	121551162	Frmd5	-
11	58955414	58955419	Hist3h2a;Trir	+
11	59205752	59205757	Mrpl55	+
9	120014159	120014164	Xirp1;Cx3cr1	-
19	5456590	5456595	Ccdc85b	-
3	101581760	101581765	Atp1a1	-
6	98021211	98021216	Mitf	+
18	38262018	38262023	Dele1	+
1	36698549	36698554	Actr1b	-
14	76507242	76507247	Tsc22d1	+
6	90645809	90645814	Slc41a3	+
8	83572309	83572314	Tecr	-
6	24604461	24604466	Lmod2	+
8	13172572	13172577	Lamp1	+
7	12925735	12925740	Rps5	+
11	6349156	6349161	Ogdh	+
10	79688123	79688128	Cdc34	+
17	48423086	48423091	Apobec2	-
2	34775212	34775217	Hspa5	+
6	99877706	99877711	Gpr27	-
4	129143064	129143069	Fndc5	+
1	165481105	165481110	Mpc2	+
8	83611884	83611889	Dnajb1	+
17	56112033	56112038	Plin5	-
10	117045792	117045797	Lrrc10	+
9	40803764	40803769	Hspa8	+
17	24730993	24730998	Rpl3l	+
13	59758968	59758973	Isca1;Gm49	-
7	118109975	118109980	Rps15a	-
12	100120097	100120102	Psmc1	+

17	56998513	56998518	Alkbh7	+
16	4482245	4482250	Srl	-
4	120667372	120667377	Cited4	+
11	78750705	78750710	Ccnq	-
12	51619856	51619861	Strn3	-
3	89450187	89450192	Pbxip1	+
17	44039209	44039214	Rcan2	+
12	103321505	103321510	Asb2	-
8	33863023	33863028	Rbpms	-
12	112654405	112654410	Akt1	-
2	167515569	167515574	Rnf114	+
18	80212121	80212126	Gm16286;Tx	+
10	79977462	79977467	Tmem259	-
7	30192373	30192378	Capns1	-
1	78469263	78469268	Farsb	-
1	170142418	170142423	Uap1	-
18	38840259	38840264	Fgf1	-
7	35548024	35548029	Nudt19	-
3	95661566	95661571	Mcl1	+
14	8166327	8166332	Pdhb	-
6	51465183	51465188	Hnrnpa2b1	-
7	138882771	138882776	Ppp2r2d	+
2	34776185	34776190	Hspa5	+
18	68260825	68260830	Fam210a	-
9	40803914	40803919	Hspa8	+
7	141881880	141881885	Tollip	-
2	25222991	25222996	Tubb4b	-
6	36528158	36528163	Chrm2	+
10	71331030	71331035	Cisd1	-
14	63200388	63200393	Gata4	-
18	80296223	80296228	Kcng2	-
3	152237863	152237868	Nexn	-
7	141473229	141473234	Polr2l	-
10	128044760	128044765	Naca	+
12	103321219	103321224	Asb2	-
8	57323942	57323947	Hand2	+
18	34943119	34943124	Hspa9	-
10	78162139	78162144	D10Jhu81e	-
18	64461576	64461581	Fech	-
11	102994747	102994752	Dcakd	-
11	61777226	61777231	Ulk2	-
13	64363893	64363898	Ctsl	-
7	45493755	45493760	Nucb1	-
19	5107973	5107978	Klc2	-
6	140652763	140652768	Aebp2	+

7	81161523	81161528	Ndufs6b	-
9	50753458	50753463	Cryab	+
9	55512069	55512074	Etfa	-
19	11774514	11774519	Mrpl16	+
7	126765529	126765534	Mapk3	+
2	121311272	121311277	Ppip5k1	-
7	128546153	128546158	Bag3	+
1	36532140	36532145	Ankrd23;Gm	-
14	34577085	34577090	Ldb3	-
2	134595580	134595585	Tmx4	-
11	75625467	75625472	Pitpna	+
5	3641446	3641451	Gatad1	-
6	142498664	142498669	Ldhb	-
7	105641068	105641073	Timm10b;Gn	+
1	177449989	177449994	Zbtb18	+
1	16229120	16229125	Stau2	-
3	116923472	116923477	Palmd	-
19	36732155	36732160	Ppp1r3c	-
3	102146145	102146150	Casq2	+
13	64364131	64364136	Ctsl	-
10	80054569	80054574	Gpx4	+
17	46017064	46017069	Vegfa	-
1	135301762	135301767	Timm17a	-
11	117813336	117813341	Syngr2;Gm2	+
19	43500311	43500316	Got1	-
11	115417897	115417902	Atp5h	-
3	138447163	138447168	Adh5	+
7	99158871	99158876	Dgat2	-
14	61219572	61219577	Sgcg	-
9	53589222	53589227	Acat1	-
17	24720568	24720573	Rps2	+
9	56937970	56937975	Imp3	+
8	95864991	95864996	Got2	-
4	46115679	46115684	Tmod1	+
1	86350524	86350529	Ncl	-
10	13515367	13515372	Fuca2	+
10	53345271	53345276	Pln	+
9	71478751	71478756	Polr2m	-
13	73816079	73816084	Slc12a7	+
10	79709682	79709687	Bsg	+
4	155832982	155832987	Aurkaip1	+
7	64412014	64412019	Mcee	+
10	86731894	86731899	Hsp90b1	-
8	22569524	22569529	Slc20a2	+
3	150073167	150073172	Rpsa-ps10	-

4	127247373	127247378	Smim12	+
4	15945350	15945355	Decr1	-
15	57871665	57871670	Derl1	-
6	142505448	142505453	Ldhb	-
16	4480506	4480511	Srl	-
7	28257085	28257090	Dyrk1b	-
12	112654158	112654163	Akt1	-
8	72586537	72586542	Tmem38a	+
7	30185335	30185340	Cox7a1	+
14	46853997	46854002	Cgrrf1	+
2	25223216	25223221	Tubb4b	-
12	111730106	111730111	Apopt1	+
2	4919111	4919116	Phyh	+
11	30506366	30506371	Acyp2	-
7	105746480	105746485	Tpp1	-
11	31549513	31549518	Ncoa2	+
17	20965583	20965588	Ppp2r1a	+
5	129022998	129023003	Ran	+
10	81486567	81486572	Ncln	-
13	24813737	24813742	BC005537	+
2	71276644	71276649	Slc25a12	-
1	153909467	153909472	Glul	+
5	115103294	115103299	Rpl37rt	-
3	32751139	32751144	Ndufb5	+
9	108391941	108391946	Usp4	+
4	156218330	156218335	Perm1	+
5	124493269	124493274	Rilpl1	-
7	28257741	28257746	Dyrk1b	-
5	145183741	145183746	Atp5j2	-
3	24333342	24333347	Gm7536	+
4	24903413	24903418	Ndufaf4	+
19	8973081	8973086	Eef1g	+
7	31051949	31051954	Fxyd1	-
2	173779286	173779291	Vapb	+
7	4521354	4521359	Tnni3	-
3	19226249	19226254	Pde7a	-
9	95561551	95561556	Paqr9	+
11	71010281	71010286	Derl2	-
5	140327960	140327965	Mrm2	-
9	55454698	55454703	Etfa	-
6	124810959	124810964	Tpi1	-
4	147875214	147875219	Mfn2	-
9	21595812	21595817	Timm29	+
10	128922682	128922687	Rdh5;Bloc1s	-
1	63150069	63150074	Ndufs1	-

7	81553033	81553038	Fsd2	-
3	101581858	101581863	Atp1a1	-
11	120560431	120560436	P4hb	-
7	81794277	81794282	Btbd1	-
18	50091502	50091507	Tnfaip8	+
18	34940858	34940863	Hspa9	-
1	164457802	164457807	Atp1b1	-
3	144691988	144691993	Sh3glb1	-
13	43151779	43151784	Tbc1d7	-
5	138163952	138163957	Cops6	+
11	67811656	67811661	Dhrs7c	+
14	34561409	34561414	Ldb3	-
2	121545979	121545984	Frmd5	-
3	104653117	104653122	Slc16a1	+
4	45105504	45105509	Tomm5	-
8	13235741	13235746	Adprhl1	-
16	4767878	4767883	Cdip1	-
3	79604997	79605002	Etfdh	-
14	55569517	55569522	Dcaf11	+
5	122458137	122458142	Atp2a2	-
7	130962074	130962079	Htra1	+
11	30880272	30880277	Psme4	+
1	131055961	131055966	Mapkapk2	-
7	13032088	13032093	Chmp2a	-
17	35821749	35821754	Ier3	+
2	26137177	26137182	Tmem250-ps	-
4	41758580	41758585	Galt;Il11ra1	+
10	60302286	60302291	Psap	+
7	143066685	143066690	Cd81	+
18	23892067	23892072	Mapre2	+
13	59755910	59755915	Isca1	-
6	71907654	71907659	Ptcd3	-
4	115811340	115811345	Atpaf1	+
11	88210990	88210995	Mrps23	+
5	123520079	123520084	Diablo;Gm49	-
3	108014823	108014828	Gstm1	-
7	140099656	140099661	Fuom	-
3	79813257	79813262	Tmem144	-
2	75640136	75640141	Rps6-ps4	+
17	24896006	24896011	Mrps34	+
2	114047426	114047431	Actc1	-
16	56033746	56033751	Trmt10c	-
11	75705883	75705888	Crk	+
8	125747714	125747719	Ntpcr	+
4	138439817	138439822	Mul1	+

8	46527265	46527270	Acsl1	+
18	67407393	67407398	Afg3l2	-
15	89418812	89418817	Gm44502;Cp	-
18	80212209	80212214	Gm16286;Tx	+
14	65976915	65976920	Clu	+
12	110858764	110858769	Mpc1-ps	-
4	127247125	127247130	Smim12	+
1	34444216	34444221	Imp4	+
11	70646134	70646139	Slc25a11	-
16	84824658	84824663	Jam2	+
1	128237316	128237321	R3hdm1	+
13	11553658	11553663	Ryr2	-
15	75901235	75901240	Eef1d	-
1	91285732	91285737	Ube2f	+
10	77269174	77269179	Pofut2	+
1	172135002	172135007	Pex19	+
11	72683578	72683583	Ube2g1	+
11	6419598	6419603	Ppia	+
15	102473296	102473301	Pcbp2	+
2	166948919	166948924	Stau1	-
8	72586146	72586151	Tmem38a	+
2	32633839	32633844	Ak1	+
2	150830507	150830512	Pygb	+
6	113493009	113493014	Creld1	+
16	20128903	20128908	Klhl24	+
16	4764877	4764882	Hmox2	+
4	126232467	126232472	Map7d1	-
7	121989272	121989277	Gga2	-
15	25971886	25971891	Retreg1	+
9	59563926	59563931	Hexa	+
11	115503055	115503060	Jpt1	-
10	34283692	34283697	Tspyl1	+
3	67474847	67474852	Gfm1	+
11	70526325	70526330	Psmb6	+
8	69901607	69901612	Ndufa13;Yjef	-
19	4007846	4007851	Ndufv1	-
5	121598608	121598613	Acad12	-
11	52122095	52122100	Ppp2ca	+
17	26792368	26792373	Bnip1	+
9	83952556	83952561	Bckdhb	+
16	20692494	20692499	Eif4g1	+
2	119596258	119596263	1700020I14R	+
3	86138493	86138498	Rps3a1	-
12	99190957	99190962	Foxn3	-
7	97550512	97550517	Aamdc	-

10	17725001	17725006	Cited2	+
16	36964469	36964474	Fbxo40	-
5	77094972	77094977	Hopx	-
17	72941599	72941604	Lbh	+
7	101822888	101822893	Inppl1	-
10	24597631	24597636	Ccn2	+
2	174345283	174345288	Gnas	+
9	105077436	105077441	Mrpl3	+
14	34529275	34529280	Ldb3	-
17	33966108	33966113	Vps52	+
7	122093661	122093666	Ndufab1	-
7	19508810	19508815	Trappc6a	+
19	37271530	37271535	Ide	-
10	80318743	80318748	2310011J03I	-
5	121528960	121528965	Adam1a;Gm	-
13	30382087	30382092	Agtr1a	+
11	52385659	52385664	Vdac1	+
11	120347130	120347135	Actg1	-
5	122469366	122469371	Atp2a2	-
9	61913325	61913330	Rplp1	-
11	31548353	31548358	Ncoa2	+
17	12228021	12228026	Map3k4	-
8	119323675	119323680	Cdh13	+
2	32634934	32634939	Ak1	+
1	65159189	65159194	Idh1	-
11	59211995	59212000	Arf1	-
14	25700288	25700293	Ppif	+
6	142495573	142495578	Ldhb	-
3	96560875	96560880	Txnip	+
5	147078932	147078937	Polr1d	+
3	89271927	89271932	Efna1	-
10	71331081	71331086	Cisd1	-
1	74395734	74395739	Ctdsp1	+
15	100619434	100619439	Dazap2	+
1	36531106	36531111	Ankrd23;Gm	-
11	75508804	75508809	Prpf8	+
9	66511843	66511848	Fbxl22	-
17	45698302	45698307	Mrpl14	+
7	19638161	19638166	Clptm1	-
4	14809987	14809992	Otud6b	-
4	42956589	42956594	Dnajb5	+
12	81996542	81996547	Pcnx	+
8	124910018	124910023	Egln1	-
8	83611402	83611407	Dnajb1	+
10	79710383	79710388	Bsg	+

11	95680621	95680626	Phb	+
16	84827967	84827972	Atp5j	-
4	132899322	132899327	Fam76a	-
4	139649362	139649367	Gm21969;Al	+
8	119410709	119410714	Mlycd	+
7	126795714	126795719	Aldoa	-
15	96688133	96688138	Slc38a2	-
11	32232985	32232990	Nprl3	-
17	48423109	48423114	Apobec2	-
11	78289392	78289397	2610507B11	+
15	73093766	73093771	Chrac1	+
1	165774156	165774161	Creg1	+
11	98438019	98438024	Mien1	-
17	56258566	56258571	Fem1a	+
2	166074367	166074372	Sulf2	-
15	27593530	27593535	Ank	+
4	45396811	45396816	Slc25a51	-
4	136255158	136255163	Tcea3	+
7	142376003	142376008	Gm49369;Ct	-
8	128505388	128505393	Pard3	+
5	114138823	114138828	Ung	+
4	129600446	129600451	Eif3i	-
2	177478925	177478930	Zfp970	+
14	34344814	34344819	Glud1	+
13	62082836	62082841	Gm48228	-
11	121440127	121440132	Fn3k	+
10	80255977	80255982	Ndufs7	+
6	71214312	71214317	Smyd1	-
9	120130308	120130313	Rpsa	+
17	29137103	29137108	Rpl35a-ps3	-
4	156220820	156220825	Perm1	+
17	35910424	35910429	Mrps18b	-
1	90215228	90215233	Ackr3	+
5	5783541	5783546	Gm15459	-
6	47525975	47525980	Cul1	+
9	107651280	107651285	Slc38a3	-
11	106782029	106782034	Ddx5	-
9	120959507	120959512	Ctnnb1	+
17	56258499	56258504	Fem1a	+
6	13084813	13084818	Tmem106b	+
6	86525075	86525080	Pcbp1	-
17	22852529	22852534	Zfp945	-
7	142376088	142376093	Gm49369;Ct	-
1	172285703	172285708	Atp1a2	-
8	94842731	94842736	Coq9	+

15	97791532	97791537	Slc48a1	+
8	95864601	95864606	Got2	-
17	33746890	33746895	Rab11b	-
9	90097882	90097887	Morf4l1	-
3	89268346	89268351	Slc50a1	-
3	138455180	138455185	Adh5	+
8	107046846	107046851	Pdf;Cog8	-
11	70012253	70012258	Acadvl	-
11	75599966	75599971	Gm12338	-
10	128088448	128088453	Atp5b	+
11	6348251	6348256	Ogdh	+
7	120641680	120641685	Uqcrc2	+
13	49203175	49203180	Card19	-
3	135465551	135465556	Ube2d3	+
5	135908500	135908505	Ywhag	-
7	93179603	93179608	Gm15501	-
11	94659802	94659807	Mrpl27	+
8	25022848	25022853	Tm2d2	+
1	182276277	182276282	Degs1	-
18	34347023	34347028	Reep5	-
10	80319116	80319121	2310011J03l	-
9	108391824	108391829	Usp4	+
4	46115563	46115568	Tmod1	+
14	75846212	75846217	Tpt1	+
13	63301830	63301835	2010111I01F	+
9	104126987	104126992	Acad11	+
18	77782354	77782359	Atp5a1	+
17	24472712	24472717	Pgp	+
11	95680107	95680112	Phb	+
10	79685340	79685345	Cdc34	+
13	12269537	12269542	Actn2	-
16	4045935	4045940	Trap1	-
7	120659461	120659466	Uqcrc2	+
9	71483489	71483494	Polr2m	-
17	45569690	45569695	Hsp90ab1	-
16	16303341	16303346	Yars2	+
11	40748696	40748701	Ccng1	-
7	80096109	80096114	Idh2	-
4	154021138	154021143	Lrrc47	+
9	53581800	53581805	Acat1	-
11	97688457	97688462	Cisd3	+
10	79735503	79735508	Hcn2	+
5	121576545	121576550	Aldh2	-
3	87922413	87922418	Mrpl24	+
3	153925822	153925827	Acadm	-

14	105682056	105682061	Gm10076	+
7	4520445	4520450	Tnni3	-
9	54601093	54601098	Idh3a	+
17	12682746	12682751	Igf2r	-
2	84441969	84441974	Tfpi	-
3	130706015	130706020	Ostc	-
8	123952758	123952763	Abcb10	-
2	144251051	144251056	Snx5	-
9	67031982	67031987	Tpm1	-
14	21381552	21381557	Adk	+
11	114668939	114668944	Rpl38	+
11	109669349	109669354	Prkar1a	+
7	34205849	34205854	Gpi1	-
6	38688324	38688329	Luc7l2	-
2	30402325	30402330	Crat	-
11	20335181	20335186	Gm12033	-
17	56101540	56101545	Plin4	-
4	42982962	42982967	Vcp	-
10	24597598	24597603	Ccn2	+
15	58783223	58783228	Tmem65	-
17	56258421	56258426	Fem1a	+
5	135786291	135786296	Mdh2	+
9	105412862	105412867	Atp2c1	-
1	75362594	75362599	Des	+
2	163466527	163466532	Fitm2	-
15	5120657	5120662	Gm10250	-
11	94659947	94659952	Mrpl27	+
13	38198260	38198265	Dsp	+
6	97247381	97247386	Lmod3	-
17	12911412	12911417	Mrpl18	-
9	123307153	123307158	Scp2-ps2	-
2	119660389	119660394	Ndufaf1	-
11	90644728	90644733	Cox11	+
7	28386843	28386848	Med29	-
5	116408636	116408641	Hspb8	-
17	56722109	56722114	Ndufa11	+
17	23674046	23674051	Hcfc1r1	+
14	65975657	65975662	Clu	+
5	124493524	124493529	Rilpl1	-
9	48495889	48495894	Gm5617	+
9	96896183	96896188	Gm10123	+
14	18280055	18280060	Nkiras1	+
11	20225021	20225026	Rab1a	+
6	133106476	133106481	Smim10l1	+
17	48423210	48423215	Apobec2	-

15	31594190	31594195	Cct5	-
3	144691997	144692002	Sh3glb1	-
5	121814553	121814558	Atxn2	+
9	58652639	58652644	Nptn	+
4	141424900	141424905	Hspb7	+
8	23148689	23148694	Ank1	+
10	80145288	80145293	Atp5d	+
16	18310887	18310892	Tango2	-
11	101580603	101580608	Nbr1	+
8	85030154	85030159	Trir	+
11	50207576	50207581	Sqstm1	-
12	8499370	8499375	Rhob	-
13	74322289	74322294	Sdha	-
6	133106384	133106389	Smim10l1	+
6	125123390	125123395	Chd4	+
16	17154195	17154200	Ube2l3	-
2	158117365	158117370	Tgm2	-
5	24581799	24581804	Gm10221	-
11	116245222	116245227	Srp68	-
17	47791963	47791968	Tfeb	+
15	75597915	75597920	Gpihbp1	+
12	69284579	69284584	Klhdc1	+
6	71871552	71871557	Immt	+
19	4004602	4004607	Gm49405;Dc	+
14	8165327	8165332	Pxk	-
9	21273524	21273529	Atg4d	+
7	118688582	118688587	Gde1	-
19	6911752	6911757	Esrra	-
10	56390164	56390169	Gja1	+
5	17783033	17783038	Cd36	-
2	131247267	131247272	Mavs	+
5	116422585	116422590	Hspb8	-
13	48880112	48880117	Fam120a	-
5	135790195	135790200	Mdh2	+
8	95864226	95864231	Got2	-
9	50756405	50756410	Cryab	+
13	45545945	45545950	Gmpr	+
7	118529674	118529679	Coq7	-
4	151060932	151060937	Camta1	-
2	26921041	26921046	Surf4	-
7	25711420	25711425	Ccdc97	-
4	123935759	123935764	Rragc	+
11	94334662	94334667	Ankrd40	+
11	4737526	4737531	Zmat5	+
19	9018828	9018833	Ahnak	+

4	41190982	41190987	Ube2r2	+
6	71872855	71872860	Immt	+
3	123006387	123006392	Myoz2	-
15	83376371	83376376	Pacsin2	-
8	13465689	13465694	Gas6	-
2	75641056	75641061	Rps6-ps4	+
4	140966516	140966521	Sdhb	+
3	148816875	148816880	Adgrl2	-
2	30413128	30413133	Crat	-
1	191309974	191309979	Nenf	-
14	73365828	73365833	Itm2b	-
14	54367672	54367677	Oxa1l	+
2	118882380	118882385	Ivd	+
10	75937619	75937624	Chchd10	+
5	24409733	24409738	Abcb8	+
9	70779804	70779809	Adam10	+
17	12227842	12227847	Map3k4	-
17	34957036	34957041	Hspa1b	-
15	51842476	51842481	Eif3h	-
5	77087370	77087375	Hopx	-
1	135852212	135852217	Tnnt2	+
9	107537966	107537971	Tmem115	+
14	88123510	88123515	Rps3a2	-
9	54589897	54589902	Idh3a	+
2	130654668	130654673	Ddrgk1	-
16	42955641	42955646	BC002163;ZI	+
5	73313635	73313640	Ociad1	+
15	4094015	4094020	Oxct1	+
5	116408977	116408982	Hspb8	-
5	33248441	33248446	Ctbp1	-
8	85325499	85325504	Mylk3	-
6	66875588	66875593	Gm9794	-
2	120089605	120089610	Ehd4	-
17	87433581	87433586	Calm2	-
11	48801660	48801665	Rack1	+
9	121712716	121712721	Ss18l2	+
8	33929547	33929552	Rbpms	-
6	72433550	72433555	Mat2a	-
6	71214186	71214191	Smyd1	-
16	18872331	18872336	Mrpl40	-
7	30563057	30563062	Psenen	-
10	81565642	81565647	Aes	+
18	77782569	77782574	Atp5a1	+
1	64994315	64994320	Rpl10a-ps1	-
11	6340450	6340455	Ogdh	+

6	50564899	50564904	Cycs	-
3	131234166	131234171	Hadh	-
7	130743541	130743546	Tacc2	+
8	105937961	105937966	Psmb10	-
7	101822963	101822968	Inppl1	-
11	51985793	51985798	Ube2b;Gm2(	-
4	129144016	129144021	Fndc5	+
14	34567500	34567505	Ldb3	-
7	128546589	128546594	Bag3	+
6	47525849	47525854	Cul1	+
7	73775862	73775867	Fam174b	+
13	43394809	43394814	Sirt5	+
2	114049336	114049341	Actc1	-
7	3706695	3706700	Rps9	+
4	3973463	3973468	Gm11808	-
8	126425013	126425018	Coa6	+
15	27593397	27593402	Ank	+
10	80643319	80643324	Btbd2	-
14	55576915	55576920	Fitm1	+
9	50635830	50635835	Dlat	-
13	30382110	30382115	Agtr1a	+
2	22588124	22588129	Gm13340	-
3	7445144	7445149	Pkia	+
4	46115497	46115502	Tmod1	+
10	18856987	18856992	Perp	+
7	30554657	30554662	Hspb6	+
17	27634090	27634095	Rps10;RPS1	-
4	95049979	95049984	Jun	-
19	9982870	9982875	Fth1	+
2	181856771	181856776	Pcmtd2	+
2	132529228	132529233	Gpcpd1	-
1	166098286	166098291	Dusp27	-
17	86997341	86997346	Rhoq	+
11	75619282	75619287	Pitpna	+
11	116023768	116023773	H3f3b	-
3	152237773	152237778	Nexn	-
7	31053909	31053914	Fxyd1	-
11	116023444	116023449	H3f3b	-
17	24722446	24722451	Ndufb10	-
10	53344150	53344155	Pln	+
2	163636842	163636847	Serinc3	-
11	62317410	62317415	Ncor1	-
5	117092814	117092819	Suds3	-
10	34283789	34283794	Tspyl1	+
2	26395668	26395673	Pmpca	+

17	23827513	23827518	Elob	-
10	95548547	95548552	Nudt4	-
8	13175014	13175019	Lamp1	+
1	75362922	75362927	Des	+
17	29301451	29301456	BC004004	+
2	76707062	76707067	Ttn	-
9	49041630	49041635	Usp28	+
6	17339455	17339460	Cav1	+
13	23739498	23739503	Hist1h1c	+
6	99878026	99878031	Tpt1-ps3	-
8	11448732	11448737	Col4a2	+
17	35895860	35895865	2310061I04F	-
4	155074559	155074564	Rer1	-
11	60864593	60864598	Tmem11	-
18	80212417	80212422	Gm16286;Tx	+
3	97690563	97690568	Pde4dip	-
11	75764952	75764957	Ywhae	+
4	139104001	139104006	Minos1	-
17	56112926	56112931	Plin5	-
12	110691182	110691187	Hsp90aa1	-
9	52088129	52088134	Rdx	+
1	74289373	74289378	Tmbim1	-
3	104655815	104655820	Slc16a1	+
10	43440272	43440277	Pdss2	+
14	54363377	54363382	Oxa1l	+
8	95865212	95865217	Got2	-
5	125386126	125386131	Ubc	-
3	122266394	122266399	Gclm	+
14	65971730	65971735	Clu	+
5	122457001	122457006	Atp2a2	-
1	40856051	40856056	Tmem182	+
14	50926606	50926611	Apex1	+
17	24853665	24853670	Hagh	+
2	76705264	76705269	Ttn	-
3	95898011	95898016	Car14	-
1	164438010	164438015	Atp1b1	-
15	103244414	103244419	Hnrnpa1	+
4	140979140	140979145	Sdhb	+
14	48079168	48079173	Ktn1	-
7	139579607	139579612	Inpp5a	+
12	83993641	83993646	Acot2	+
17	40960714	40960719	Mut	+
15	81914920	81914925	Aco2	+
17	66080819	66080824	Ndufv2	-
6	32792772	32792777	Chchd3	-

11	21323248	21323253	Ugp2	-
14	55662452	55662457	Nedd8	-
2	125673800	125673805	Eid1	+
10	128360107	128360112	Cs	+
4	141113197	141113202	Szrd1	-
10	61693235	61693240	Sar1a	+
8	70010831	70010836	Gm7730	-
17	26126468	26126473	Mrpl28	+
5	139338066	139338071	Cox19	-
12	28681968	28681973	Adi1	+
1	80271546	80271551	Cul3	-
11	51685927	51685932	0610009B22I	-
6	108140283	108140288	Rpl36-ps12;S	-
2	104426686	104426691	Hipk3	-
15	33594273	33594278	Cpq	+
1	172273446	172273451	Atp1a2	-
9	4296724	4296729	Aasdhppt	-
6	115618934	115618939	Raf1	-
16	56034180	56034185	Trmt10c	-
5	148505716	148505721	Ubl3	-
10	71228228	71228233	Tfam	-
15	55540155	55540160	Mrpl13	-
13	58394694	58394699	Hnrnpk	-
8	124948384	124948389	Egln1	-
3	20122704	20122709	Gyg	-
2	4938472	4938477	Phyh	+
12	84431101	84431106	Aldh6a1	-
13	73331782	73331787	Mrpl36	+
9	20644826	20644831	Ubl5	+
16	23111513	23111518	Eif4a2	+
7	3297174	3297179	Myadm;Prkc	+
4	148617382	148617387	Tardbp	-
2	25466736	25466741	Ptgds	-
1	51301881	51301886	Cavin2	+
8	119348103	119348108	Hsbp1	+
13	73814946	73814951	Slc12a7	+
1	37418977	37418982	Coa5	-
15	88863465	88863470	Pim3	+
17	48416841	48416846	Oard1	+
10	78169392	78169397	D10Jhu81e	-
17	46251890	46251895	Yipf3	+
5	115560964	115560969	Rplp0	+
14	105682015	105682020	Gm10076	+
2	174346303	174346308	Gnas	+
2	4938399	4938404	Phyh	+

11	5525089	5525094	Xbp1	+
10	41907419	41907424	Sesn1	+
14	34528231	34528236	Ldb3	-
9	101107281	101107286	Ppp2r3a	-
19	34811224	34811229	Pank1	-
11	29161530	29161535	Pnpt1	+
9	55486740	55486745	Etfa	-
6	5485374	5485379	Pdk4	-
17	35149936	35149941	Prrc2a	-
7	28830217	28830222	Ech1	+
10	26984395	26984400	Lama2	-
1	36534099	36534104	Ankrd23;Gm	-
4	105031669	105031674	Prkaa2	-
10	34283251	34283256	Tspyl1	+
11	70729077	70729082	Kif1c	+
11	87980241	87980246	Dynll2	-
19	42123527	42123532	Avpi1	-
2	155389506	155389511	Trp53inp2	+
5	124550102	124550107	Tmed2	+
7	73776049	73776054	Fam174b	+
17	23675127	23675132	Hcfc1r1	+
3	5860439	5860444	Selenot	+
7	54412117	54412122	Svip	-
9	107598960	107598965	Sema3b	-
13	49208128	49208133	Card19	-
14	118157988	118157993	Gpr180	+
15	89156120	89156125	Plxnb2	-
3	101576529	101576534	Atp1a1	-
4	139318396	139318401	Akr7a5	+
11	21333708	21333713	Ugp2	-
2	155389704	155389709	Trp53inp2	+
6	55347176	55347181	Aqp1	+
3	104656721	104656726	Slc16a1	+
19	11774196	11774201	Mrpl16	+
11	75764705	75764710	Ywhae	+
19	27253786	27253791	Vldlr	+
3	10208534	10208539	Fabp4	-
13	113663311	113663316	Hspb3	-
13	18031583	18031588	Vdac3-ps1	-
1	90612504	90612509	Cops8	+
15	58933865	58933870	Ndufb9;Gm4	+
4	91805540	91805545	Mtap	-
4	15929861	15929866	Decr1	-
3	32577617	32577622	Mfn1	+
13	35979504	35979509	Lyrm4	-

5	69516903	69516908	Yipf7	-
19	6911256	6911261	Esrra	-
7	131568541	131568546	Bub3	+
8	124909017	124909022	Egln1	-
5	122405971	122405976	Arpc3	+
19	9984921	9984926	Fth1	+
16	17280475	17280480	Pi4ka	-
15	98132089	98132094	Pfkm	+
3	102145791	102145796	Casq2	+
17	81386945	81386950	Slc8a1	-
5	24441552	24441557	Fastk	-
9	101107481	101107486	Ppp2r3a	-
6	40476403	40476408	Ssbp1	+
8	124910513	124910518	Egln1	-
12	110582039	110582044	Ppp2r5c	+
15	76070305	76070310	Puf60	-
16	4764948	4764953	Hmox2	+
2	155817894	155817899	BC029722	-
2	36036623	36036628	Ndufa8	-
10	13647368	13647373	Aig1	-
9	104127307	104127312	Acad11	+
15	81907457	81907462	Aco2	+
4	21896614	21896619	Coq3	+
12	8931655	8931660	Laptm4a	+
15	99409837	99409842	Tmbim6	+
2	150831523	150831528	Pygb	+
2	119660045	119660050	Ndufaf1	-
8	123893247	123893252	Acta1	-
6	72606095	72606100	Retsat	+
7	109893979	109893984	Dennd5a	-
11	40749934	40749939	Ccng1	-
12	72659478	72659483	Dhrs7	-
5	136120003	136120008	Polr2j	+
2	150618711	150618716	Acss1	-
7	144929998	144930003	Ccnd1	-
1	24615219	24615224	Gm28661	-
17	47791564	47791569	Tfeb	+
6	125193394	125193399	Mrpl51	+
2	32707570	32707575	Cdk9	-
10	78159527	78159532	Pfkl	-
11	68902300	68902305	Rpl26	+
4	3973524	3973529	Gm11808	-
2	34775980	34775985	Hspa5	+
5	77087611	77087616	Hopx	-
7	28258222	28258227	Dyrk1b	-

5	97886199	97886204	Antxr2	-
2	68211281	68211286	Stk39	-
7	121076414	121076419	Mettl9	+
11	102436457	102436462	Grn	+
5	122456416	122456421	Atp2a2	-
12	3249673	3249678	Rab10	-
2	18684480	18684485	Bmi1	+
6	90645669	90645674	Slc41a3	+
2	71282524	71282529	Slc25a12	-
9	108339954	108339959	Gpx1	+
1	151363821	151363826	Ivns1abp	+
9	65690252	65690257	Oaz2	+
1	97834506	97834511	Pam	-
5	147078616	147078621	Polr1d	+
8	71381561	71381566	Nr2f6	-
8	34170594	34170599	Saraf	+
3	95622684	95622689	Rps10-ps1	+
10	77988174	77988179	Pfkl	-
7	84615655	84615660	Zfand6	-
11	6355565	6355570	Ogdh	+
10	91116819	91116824	Slc25a3	-
15	89155702	89155707	Plxnb2	-
1	156366097	156366102	Gm2000	+
1	93408386	93408391	Hdlbp	-
4	41191086	41191091	Ube2r2	+
7	142376686	142376691	Gm49369;Ct	-
7	43454539	43454544	Etfb;Gm4523	+
10	45369375	45369380	Bves	+
8	84661661	84661666	Ier2	-
4	49586125	49586130	Tmem246	-
14	26915139	26915144	Asb14	+
9	21266964	21266969	Atg4d	+
11	40748706	40748711	Ccng1	-
19	43499867	43499872	Got1	-
8	69894384	69894389	Ndufa13;Yjef	-
15	103345076	103345081	Itga5	-
18	80212451	80212456	Gm16286;Tx	+
3	52982719	52982724	Cog6	-
10	53345466	53345471	Pln	+
8	36568302	36568307	Dlc1	-
10	40287851	40287856	Amd1	-
17	24640144	24640149	Slc9a3r2	-
2	75641370	75641375	Rps6-ps4	+
11	40749031	40749036	Ccng1	-
19	37021936	37021941	Cpeb3	-

7	73775580	73775585	Fam174b	+
2	131938097	131938102	Prnp;Prn	+
13	99319841	99319846	Ptcd2	-
17	56711394	56711399	Ranbp3	+
5	65450371	65450376	Gm43552;Sr	-
10	121411654	121411659	Rassf3	-
15	99409447	99409452	Tmbim6	+
7	139659615	139659620	Cfap46	-
8	83572409	83572414	Tecr	-
6	82725369	82725374	Hk2	-
1	55091234	55091239	Hspe1	+
16	35697884	35697889	Dirc2	-
2	75641665	75641670	Rps6-ps4	+
2	76704744	76704749	Ttn	-
2	34775900	34775905	Hspa5	+
12	65073889	65073894	Fkbp3	-
2	146306533	146306538	Gm14117	+
19	27253923	27253928	Vldlr	+
2	30097496	30097501	Zer1	-
9	104126719	104126724	Acad11	+
8	104628401	104628406	Rrad	-
2	90894836	90894841	Ndufs3	-
14	8166257	8166262	Pdhb	-
13	64367897	64367902	Ctsl	-
11	58994765	58994770	Obscn	-
11	120105769	120105774	Slc38a10	-
14	63142336	63142341	Ctsb	+
7	28352547	28352552	Rps16	+
13	62082470	62082475	Gm48228	-
4	44032484	44032489	Clta	+
6	56891542	56891547	Nt5c3	-
8	95866922	95866927	Got2	-
8	34170471	34170476	Saraf	+
19	34244933	34244938	Acta2	-
11	78290442	78290447	2610507B11	+
3	52982512	52982517	Cog6	-
9	120015471	120015476	Xirp1;Cx3cr1	-
10	33198475	33198480	Trdn	+
17	56259495	56259500	Fem1a	+
17	15477370	15477375	Psmb1	-
2	76705817	76705822	Ttn	-
5	67355586	67355591	Slc30a9	+
7	114264878	114264883	Psma1	-
4	130315372	130315377	Fabp3	+
4	134164269	134164274	Cep85	+

17	12911502	12911507	Mrpl18	-
11	69917200	69917205	Eif5a	-
11	95831499	95831504	Phospho1;Zf	+
15	3319067	3319072	Ghr	-
11	50230689	50230694	Mgat4b	+
10	128048496	128048501	Naca	+
14	32180524	32180529	Timm23	-
17	24864320	24864325	Hagh	+
11	70664013	70664018	Spag7	-
13	99319938	99319943	Ptcd2	-
11	94339756	94339761	Ankrd40	+
5	124493462	124493467	Rilpl1	-
15	76344942	76344947	Cyc1	+
7	81768818	81768823	Ramac	+
3	89408905	89408910	Flad1	-
12	87273611	87273616	Ahsa1	+
2	122052221	122052226	Eif3j1	+
9	108085495	108085500	Apeh	-
4	40947884	40947889	Bag1	-
12	8499872	8499877	Rhob	-
1	153908687	153908692	Glul	+
6	142490411	142490416	Ldhb	-
12	84343669	84343674	Zfp410	+
11	120369811	120369816	Faap100	-
11	60864784	60864789	Tmem11	-
17	56613915	56613920	Rpl36	+
10	24597722	24597727	Ccn2	+
2	30402369	30402374	Crat	-
7	128546365	128546370	Bag3	+
3	152237543	152237548	Nexn	-
14	8169773	8169778	Pdhb	-
14	20500765	20500770	Ppp3cb	-
2	33730070	33730075	Mvb12b	-
8	72257229	72257234	Ap1m1	+
13	63301839	63301844	2010111I01F	+
10	81559637	81559642	Aes	+
15	76904407	76904412	Rpl8	+
3	101582789	101582794	Atp1a1	-
12	113144470	113144475	Crip2	+
13	46429168	46429173	Rbm24	+
5	112315187	112315192	Tpst2	+
3	95622692	95622697	Rps10-ps1	+
4	129715600	129715605	Khdrbs1	-
15	76354083	76354088	Maf1	+
14	32082884	32082889	Dph3	-

17	26699520	26699525	Atp6v0e	+
1	10034023	10034028	Cops5	-
8	13174767	13174772	Lamp1	+
2	131178086	131178091	Cenpb;Spef1	-
4	129142873	129142878	Fndc5	+
15	101274442	101274447	Nr4a1	+
4	123715892	123715897	Ndufs5	-
8	72814636	72814641	Large1	-
2	121546066	121546071	Frmd5	-
4	149234801	149234806	Kif1b	-
17	31521671	31521676	Ndufv3	+
19	43500410	43500415	Got1	-
1	180802654	180802659	H3f3a	-
18	77782350	77782355	Atp5a1	+
5	122457179	122457184	Atp2a2	-
11	70527211	70527216	Psmb6	+
4	155821076	155821081	Ccnl2	+
19	41214970	41214975	Tm9sf3	-
3	67393986	67393991	Mlf1	+
17	33692028	33692033	March2	-
13	58127253	58127258	Hnrnpa0	-
12	91806478	91806483	Sel1l	-
14	120946964	120946969	Ipo5	+
11	69089186	69089191	Vamp2	+
12	100121122	100121127	Psmc1	+
14	57917213	57917218	Micu2	-
11	102404837	102404842	Slc25a39	-
6	47525802	47525807	Cul1	+
10	42502439	42502444	Snx3	+
17	34957100	34957105	Hspa1b	-
9	104127123	104127128	Acad11	+
7	80390457	80390462	Furin	-
4	138313448	138313453	Pink1	-
2	163725902	163725907	Pkig	+
4	140966568	140966573	Sdhb	+
12	85301859	85301864	Nek9	-
7	73776418	73776423	Fam174b	+
7	28377482	28377487	Zfp36	-
6	83051342	83051347	Htra2	-
3	36091225	36091230	Gm43079;Ac	+
19	45012627	45012632	Twnk	+
17	25876546	25876551	Mettl26;Gm2	+
2	130278932	130278937	Nop56	+
12	79156358	79156363	Vti1b	-
9	53595320	53595325	Acat1	-

13	49203083	49203088	Card19	-
7	102111062	102111067	Art1	+
5	36471074	36471079	Grpel1	+
11	52122162	52122167	Ppp2ca	+
10	61702385	61702390	Tysnd1	+
3	52982254	52982259	Cog6	-
8	95865236	95865241	Got2	-
3	96528581	96528586	Hfe2	+
12	21324034	21324039	Adam17	-
9	66518066	66518071	Usp3	-
3	135466548	135466553	Ube2d3	+
8	121596062	121596067	Map1lc3b;Gr	+
12	32846720	32846725	Nampt	+
12	31331945	31331950	Dld	-
9	120014598	120014603	Xirp1;Cx3cr1	-
7	141496017	141496022	Chid1	-
6	85134040	85134045	Spr	-
2	17343538	17343543	Pmepa1	-
2	127247533	127247538	Ciao1	-
7	3704391	3704396	Rps9	+
11	55500264	55500269	G3bp1	+
17	70996729	70996734	Myl12a;Myl1:	-
19	5918104	5918109	Cdc42ep2	-
16	22872035	22872040	Dnajb11	+
9	7752094	7752099	Gm10709	+
10	80145455	80145460	Atp5d	+
1	180811905	180811910	H3f3a	-
12	87164119	87164124	Gstz1	+
5	116013902	116013907	Prkab1	-
19	6262157	6262162	Atg2a	+
15	76721824	76721829	Lrrc24;C030(	-
8	3151596	3151601	Insr	-
19	4035855	4035860	Gstp1	-
1	182487740	182487745	Capn2	-
7	16116809	16116814	Napa	+
4	138317428	138317433	Pink1	-
8	22580415	22580420	Vdac3	-
8	22578052	22578057	Vdac3	-
17	35895755	35895760	2310061I04F	-
11	54991416	54991421	Anxa6	-
15	83116543	83116548	Serhl	+
2	91130899	91130904	Mybpc3	+
10	120781188	120781193	Msrb3	-
2	168210549	168210554	Gm20716;Dp	-
15	79249170	79249175	Pick1;Gm49	+

11	51985944	51985949	Ube2b;Gm26	-
17	74201173	74201178	Memo1	-
9	110982410	110982415	Lrrc2	+
2	121548835	121548840	Frmd5	-
2	26347872	26347877	Gpsm1	+
11	5704633	5704638	Mrps24	-
2	174465477	174465482	Prelid3b	-
1	171463751	171463756	F11r	+
16	33947738	33947743	Itgb5	+
15	12575917	12575922	Gm2606	+
17	43456491	43456496	Adgrf5	+
6	83797230	83797235	Nagk	+
2	74876446	74876451	Mtx2	+
9	54604087	54604092	Idh3a	+
15	81914884	81914889	Aco2	+
5	122794445	122794450	Anapc5	-
2	119606541	119606546	1700020I14F	+
4	46115311	46115316	Tmod1	+
8	94394702	94394707	Herpud1	+
6	24573053	24573058	Asb15	+
2	76914533	76914538	Ttn	-
7	25630157	25630162	Bckdha	-
3	95032776	95032781	Psmd4	-
5	124570849	124570854	Eif2b1	-
4	119278256	119278261	Ybx1	-
11	95492229	95492234	Spop	+
2	90849645	90849650	Mtch2	+
10	81396385	81396390	Nfic	-
7	28826223	28826228	Ech1	+
8	124909358	124909363	Egln1	-
5	117091923	117091928	Suds3	-
7	114047000	114047005	Rras2	-
7	45589509	45589514	Bcat2	+
4	116069916	116069921	Uqcrh	-
11	98384604	98384609	Tcap	+
17	29282216	29282221	BC004004	+
1	58900853	58900858	Trak2	-
9	21008636	21008641	Mrpl4	+
16	4706845	4706850	Dnaja3	+
8	11454053	11454058	Rab20	-
2	26636307	26636312	Fam69b	+
5	122458193	122458198	Atp2a2	-
11	40749518	40749523	Ccng1	-
10	13515125	13515130	Fuca2	+
1	135850708	135850713	Tnnt2	+

11	77459942	77459947	Ssh2	+
10	128957742	128957747	Itga7	+
7	141160676	141160681	Rnh1	-
16	4049448	4049453	Trap1	-
8	123892278	123892283	Acta1	-
1	151356185	151356190	Ivns1abp	+
17	10211477	10211482	Qk	-
6	72153527	72153532	St3gal5	+
6	56882885	56882890	Nt5c3	-
11	40750365	40750370	Ccng1	-
11	102434752	102434757	Grn	+
9	86582713	86582718	Me1	-
18	12156337	12156342	Riok3	+
7	142376199	142376204	Gm49369;Ct	-
13	99415316	99415321	Mrps27	+
5	88671205	88671210	Grsf1	-
14	37122134	37122139	Ghitm	-
19	3455431	3455436	Ppp6r3	-
7	97530408	97530413	Ints4	+
11	40749346	40749351	Ccng1	-
8	124909927	124909932	Egln1	-
5	135786308	135786313	Mdh2	+
6	142501390	142501395	Ldhb	-
2	76706922	76706927	Ttn	-
11	116172007	116172012	Acox1	-
9	54595207	54595212	Idh3a	+
12	13219441	13219446	Ddx1	-
3	122015808	122015813	Arhgap29	+
2	26392521	26392526	Pmpca	+
1	125393530	125393535	Actr3	-
11	21565852	21565857	Mdh1	-
14	19812101	19812106	Rtraf	-
8	23148298	23148303	Ank1	+
11	58995451	58995456	Obscn	-
3	20125343	20125348	Gyg	-
13	12304315	12304320	Actn2	-
1	183327301	183327306	Mia3	-
10	57522980	57522985	Serinc1	-
4	82290621	82290626	Nfib	-
15	25971917	25971922	Retreg1	+
6	108822106	108822111	Arl8b	+
2	103088565	103088570	Apip	+
7	105557845	105557850	Smpd1	+
8	104629826	104629831	Rrad	-
5	115801650	115801655	Rab35	-

10	80899948	80899953	Timm13	-
18	46502514	46502519	Fem1c	-
1	125405897	125405902	Actr3	-
5	122453606	122453611	Atp2a2	-
15	89417188	89417193	Gm44502;Cp	-
7	130764579	130764584	Tacc2	+
12	3249153	3249158	Rab10	-
7	126489874	126489879	Tufm	+
2	30284470	30284475	Dolk	-
5	24444193	24444198	Fastk	-
5	134238095	134238100	Gtf2i	-
8	94854475	94854480	Coq9	+
5	3236768	3236773	Gm15772	+
7	19295983	19295988	Rtn2	+
11	116173260	116173265	Acox1	-
13	97176129	97176134	Gfm2	+
5	124493417	124493422	Rilpl1	-
19	5493945	5493950	Cfl1	+
7	137459277	137459282	Glrx3	+
11	59013302	59013307	Obscn	-
10	13515273	13515278	Fuca2	+
8	119323954	119323959	Cdh13	+
4	107200541	107200546	Tmem59	+
1	120119920	120119925	Dbi	-
2	131178016	131178021	Cenpb;Spef1	-
7	98717932	98717937	Thap12	+
10	80900234	80900239	Timm13	-
10	53345162	53345167	Pln	+
2	25223113	25223118	Tubb4b	-
5	20886633	20886638	Tmem60	+
7	105742698	105742703	Ilk	+
7	126780276	126780281	Ypel3	+
9	21230520	21230525	Keap1	-
3	88568539	88568544	Ubqln4	+
12	103321241	103321246	Asb2	-
9	118063283	118063288	Azi2	+
5	115117642	115117647	Acads	-
11	59012327	59012332	Obscn	-
2	10056078	10056083	Atp5c1	-
11	115415804	115415809	Atp5h	-
14	120946812	120946817	Ipo5	+
15	57871286	57871291	Derl1	-
8	122883528	122883533	Gm20388	-
1	156006259	156006264	Tor1aip1	-
4	8578490	8578495	Rab2a	+

8	104628183	104628188	Rrad	-
5	124460649	124460654	Kmt5a	+
3	146839034	146839039	Gm10288	-
5	137782046	137782051	Mepce	-
7	43454528	43454533	Etfb;Gm452	+
6	50564510	50564515	Cycs	-
17	56259341	56259346	Fem1a	+
19	4035509	4035514	Gstp1	-
19	46032870	46032875	Ldb1	-
2	168182225	168182230	Gm20716;Ac	-
9	106888840	106888845	Manf	-
14	77251840	77251845	Tsc22d1	-
12	13219401	13219406	Ddx1	-
2	91263842	91263847	Pacsin3	+
1	75367459	75367464	Des	+
10	120781909	120781914	Msrb3	-
5	17828977	17828982	Cd36	-
13	38500738	38500743	Txndc5	-
6	87845206	87845211	Cnbp	-
16	18778116	18778121	Cldn5	+
10	24596566	24596571	Ccn2	+
13	12270837	12270842	Actn2	-
16	4054890	4054895	Trap1	-
6	72369265	72369270	Vamp5	-
16	91563728	91563733	Ifngr2	+
6	67267332	67267337	Serbp1	+
2	162934597	162934602	Srsf6	+
19	43504898	43504903	Got1	-
1	181242326	181242331	Rpl35a-ps2	-
2	181148690	181148695	Eef1a2	-
19	3909209	3909214	Ndufs8	-
11	69980893	69980898	Elp5	+
19	5708100	5708105	Ehbp1l1	-
2	163467260	163467265	Fitm2	-
6	35261575	35261580	1810058I24R	+
15	76721518	76721523	Lrrc24;C030	-
7	46855196	46855201	Ldha	+
11	78290099	78290104	2610507B11	+
19	5456671	5456676	Ccdc85b	-
16	20663096	20663101	Psmd2	+
6	39598296	39598301	Ndufb2	+
13	30540963	30540968	Uqcrfs1	-
4	42957820	42957825	Dnajb5	+
7	101823175	101823180	Inppl1	-
9	37649435	37649440	Tbrg1	-

11	98384831	98384836	Tcap	+
1	161241343	161241348	Prdx6	-
5	129746355	129746360	Nipsnap2	+
13	58126617	58126622	Hnrnpa0	-
2	76709800	76709805	Ttn	-
14	76507283	76507288	Tsc22d1	+
16	11144242	11144247	Gm23935	+
4	129143631	129143636	Fndc5	+
4	57370845	57370850	Ptpn3	-
1	135852075	135852080	Tnnt2	+
17	10206822	10206827	Qk	-
3	10208462	10208467	Fabp4	-
18	35613435	35613440	Paip2	+
7	74275988	74275993	Slco3a1	-
5	125386629	125386634	Ubc	-
11	106788288	106788293	Ddx5	-
18	68266610	68266615	Fam210a	-
14	8170385	8170390	Pdhb	-
17	35682323	35682328	Ddr1	-
2	69683735	69683740	Klhl41	+
6	6042206	6042211	Slc25a13	-
17	73242684	73242689	Lclat1	+
5	139338056	139338061	Cox19	-
1	73914233	73914238	Tns1	-
2	181856445	181856450	Pcmtd2	+
9	51943618	51943623	Fdx1	-
17	66495132	66495137	Rab12	-
3	90487855	90487860	Ilf2	+
8	95713907	95713912	Ndrg4	+
11	49218000	49218005	Zfp62	+
6	99877883	99877888	Tpt1-ps3	-
15	83574026	83574031	Tspo	+
6	145187380	145187385	Gm15543	+
7	102110973	102110978	Art1	+
19	45005577	45005582	Mrpl43	-
11	43424740	43424745	Pttg1	-
9	44742629	44742634	Arcn1	-
14	75848177	75848182	Tpt1	+
1	55148620	55148625	Mob4	+
2	80501032	80501037	Nckap1	-
9	54603285	54603290	Idh3a	+
15	81849000	81849005	Tob2	-
2	145916191	145916196	Naa20	+
2	25561966	25561971	Edf1	+
6	145216212	145216217	Etfrf1	+

19	7199145	7199150	Otub1	-
2	153014075	153014080	Pdrg1	-
10	80036097	80036102	Polr2e	-
11	60864687	60864692	Tmem11	-
3	102146095	102146100	Casq2	+
4	132186760	132186765	Ythdf2	-
13	34988303	34988308	Eci2	-
11	96817583	96817588	Nfe2l1	-
14	32180438	32180443	Timm23	-
5	95862263	95862268	Gm5559	+
5	139394782	139394787	Gpr146	+
14	65980854	65980859	Clu	+
11	6418226	6418231	Ppia	+
5	143514778	143514783	Rac1	-
18	60777817	60777822	Rps14	+
11	115514226	115514231	Jpt1	-
8	70895987	70895992	Rpl18a	-
13	64368003	64368008	Ctsl	-
11	52383912	52383917	Vdac1	+
4	132899426	132899431	Fam76a	-
16	57316233	57316238	Cmss1	-
4	15917574	15917579	Decr1	-
9	64182467	64182472	Snapc5	+
15	58784379	58784384	Tmem65	-
10	77597216	77597221	Pttg1ip	+
5	110274483	110274488	Pxmp2	-
5	134148361	134148366	Rcc1l	-
17	86999228	86999233	Rhoq	+
19	9984191	9984196	Fth1	+
5	122101019	122101024	Myl2	+
4	129849499	129849504	Ptp4a2	+
8	84246509	84246514	D8Ertd738e	-
1	151363568	151363573	Ivns1abp	+
15	75896385	75896390	Eef1d	-
4	141620276	141620281	Slc25a34	-
11	94214987	94214992	Tob1	+
1	43141771	43141776	Fhl2	-
7	103826635	103826640	Hbb-bs	-
2	75641412	75641417	Rps6-ps4	+
15	65872383	65872388	Efr3a	+
13	49623177	49623182	Ogn	+
15	81826243	81826248	Tef	+
10	53344592	53344597	Pln	+
19	8969981	8969986	Eef1g	+
14	63143008	63143013	Ctsb	+

7	90208716	90208721	Picalm	+
9	65660117	65660122	Rbpms2	+
18	9314677	9314682	Ccny	-
5	31141637	31141642	Mpv17	-
7	45916880	45916885	Tmem143	+
4	109454399	109454404	Rnf11	-
13	43401827	43401832	Nol7	+
15	96689784	96689789	Slc38a2	-
3	32577219	32577224	Mfn1	+
1	93407950	93407955	Hdlbp	-
15	80968966	80968971	Adsl	+
17	83502008	83502013	Cox7a2l	-
14	51905983	51905988	Ndrg2	-
15	86139326	86139331	Cerk	-
2	150831372	150831377	Pygb	+
4	119418839	119418844	Ppcs	-
13	64367015	64367020	Ctsl	-
17	71252246	71252251	Emilin2	-
10	80666609	80666614	Mknk2	-
15	27593783	27593788	Ank	+
9	54954688	54954693	Psma4	+
7	121922964	121922969	Cog7	-
5	17828800	17828805	Cd36	-
18	14625761	14625766	Ss18	-
17	26506204	26506209	Dusp1	-
11	30874161	30874166	Psme4	+
6	142588039	142588044	Abcc9	-
15	85393429	85393434	Atxn10	+
11	58315885	58315890	Zfp672	-
4	105029689	105029694	Gm12715	-
18	35806120	35806125	AC141471.2;	+
7	19565187	19565192	Gemin7	-
14	61545809	61545814	Sprydd7	-
1	151360161	151360166	Ivns1abp	+
19	6982843	6982848	Vegfb	-
9	40803386	40803391	Hspa8	+
11	102306311	102306316	Ubtf	-
8	15104083	15104088	Myom2	+
4	108044383	108044388	Podn;Scp2	-
9	21589266	21589271	Carm1	+
5	135909314	135909319	Ywhag	-
17	31531155	31531160	Ndufv3	+
9	64185852	64185857	Map2k1	-
15	83116537	83116542	Serhl	+
4	3973283	3973288	Gm11808	-

15	76085657	76085662	Nrbp2	-
14	31000088	31000093	Spcs1	-
8	104628341	104628346	Rrad	-
13	30540999	30541004	Uqcrfs1	-
1	134755371	134755376	Ppp1r12b	-
9	66955805	66955810	Lactb	-
17	33838250	33838255	Ndufa7	+
11	70644391	70644396	Slc25a11	-
1	171129529	171129534	Sdhc	-
19	7585644	7585649	Pla2g16	+
1	151363781	151363786	Ivns1abp	+
5	135889071	135889076	Hspb1	+
11	88211436	88211441	Mrps23	+
17	56995893	56995898	Clpp	+
8	105856590	105856595	Thap11	+
5	24565792	24565797	Abcf2	-
7	99839771	99839776	Spcs2	-
3	101595095	101595100	Atp1a1	-
9	108340224	108340229	Gpx1	+
4	119281642	119281647	Ybx1	-
10	94220942	94220947	Ndufa12	+
9	50605121	50605126	Timm8b	+
17	56120078	56120083	Lrg1	-
7	27638893	27638898	Akt2	+
5	20760594	20760599	Phtf2	-
8	71586188	71586193	Slc27a1	+
10	58423123	58423128	Lims1	+
6	52546329	52546334	Hibadh	-
8	27275329	27275334	Eif4ebp1	+
7	140112468	140112473	Echs1	-
11	101259291	101259296	Vps25;Ramp	+
19	36118320	36118325	Ankrd1	-
2	24974414	24974419	Mrpl41	-
18	38317005	38317010	Rnf14	+
9	56146514	56146519	Tspan3	-
16	20722173	20722178	Polr2h	+
16	16268685	16268690	Pkp2	+
10	79710785	79710790	Bsg	+
4	49513481	49513486	Mrpl50	-
5	17782842	17782847	Cd36	-
17	29282195	29282200	BC004004	+
4	129595323	129595328	Eif3i	-
2	38590698	38590703	Psmb7	-
14	8164764	8164769	Pxk	+
11	60227511	60227516	Tom1l2	-

11	93949824	93949829	Nme2;Gm20	-
12	69182950	69182955	Rpl36al;Gm4	-
19	24683347	24683352	Pgm5	-
5	117376509	117376514	Wsb2	+
7	131445801	131445806	Acadsb	+
18	46588308	46588313	Tmed7	-
7	67231927	67231932	Mef2a	-
6	5483729	5483734	Pdk4	-
5	73635625	73635630	Sgcb	-
4	144927570	144927575	Dhrs3	+
2	108949699	108949704	Gm13910	+
19	55067883	55067888	Gpam	-
11	52388790	52388795	Vdac1	+
15	83484054	83484059	Ttll1	-
14	54942363	54942368	Myh6	-
5	30119639	30119644	Hadha	-
11	5192549	5192554	Kremen1	-
17	34616715	34616720	Gm20460;Pp	-
15	73751642	73751647	Ptp4a3	+
7	23947064	23947069	Gm10175	-
1	92460399	92460404	Ndufa10	-
9	110966477	110966482	Lrrc2	+
8	61503084	61503089	Cbr4	+
18	34946627	34946632	Hspa9	-
10	41908204	41908209	Sesn1	+
17	26124623	26124628	Mrpl28	+
9	44686916	44686921	Phldb1	-
3	106034886	106034891	Gm4540	+
4	40732994	40732999	Dnaja1	+
2	180037447	180037452	Psma7	-
2	119172197	119172202	Gchfr	+
3	104656953	104656958	Slc16a1	+
14	63142580	63142585	Ctsb	+
1	165214990	165214995	Tiprl	-
17	8141609	8141614	Gm49673;Rr	-
15	73751846	73751851	Ptp4a3	+
10	81396530	81396535	Nfic	-
19	4127786	4127791	Tmem134	+
1	182124861	182124866	Srp9	+
10	40249624	40249629	Gtf3c6	-
3	104656424	104656429	Slc16a1	+
1	153909610	153909615	Glul	+
11	70238177	70238182	Gm21988;Rr	-
2	121461101	121461106	Mfap1b	-
12	111669514	111669519	Ckb	-

17	27631243	27631248	Rps10;RPS1	-
5	30119566	30119571	Hadha	-
10	128558736	128558741	Pa2g4	-
5	24813808	24813813	Rheb	-
7	126489411	126489416	Tufm	+
17	26695394	26695399	Atp6v0e	+
1	151349480	151349485	Ivns1abp	+
10	80255107	80255112	Ndufs7	+
7	141473157	141473162	Polr2l	-
12	101968204	101968209	Ndufb1-ps	-
10	80828982	80828987	Oaz1	+
9	111224166	111224171	Lrrfip2	+
4	15918749	15918754	Decr1	-
18	77781326	77781331	Atp5a1	+
6	71124664	71124669	Rpl34-ps1	+
14	101934207	101934212	Lmo7	+
2	35314232	35314237	Stom	-
11	116171990	116171995	Acox1	-
15	81914863	81914868	Aco2	+
2	13582501	13582506	Vim	+
1	135301913	135301918	Timm17a	-
14	45459965	45459970	Fermt2	-
8	70700106	70700111	Jund	+
10	81367120	81367125	Fzr1	-
15	79029415	79029420	H1f0	+
7	27581707	27581712	2310022A10I	+
7	28788166	28788171	Sirt2	+
4	40948030	40948035	Bag1	-
11	46728669	46728674	Gm12174	+
9	54596065	54596070	Idh3a	+
1	16102725	16102730	Rpl7	-
8	104631137	104631142	Rrad	-
7	19415022	19415027	Ckm	+
11	22956523	22956528	Gm28048;Cc	-
7	64872051	64872056	Nsmce3;Fan	-
7	30184204	30184209	Cox7a1	+
7	111072658	111072663	Eif4g2	-
2	155817978	155817983	BC029722	-
3	116549828	116549833	Dbt	+
5	110363355	110363360	Fbrsl1	-
5	77087305	77087310	Hopx	-
5	129757307	129757312	Nipsnap2	+
8	123095187	123095192	Spg7;Gm203	+
8	69901546	69901551	Ndufa13;Yjef	-
7	103827476	103827481	Hbb-bs	-

13	90104489	90104494	Tmem167	+
9	118565588	118565593	Golga4	+
4	147985937	147985942	Nppb	+
17	24861341	24861346	Hagh	+
6	125193453	125193458	Mrpl51	+
17	46018200	46018205	Vegfa	-
11	101259150	101259155	Vps25;Ramp	+
12	73281286	73281291	Trmt5	-
1	180167021	180167026	Coq8a	-
17	70994304	70994309	Myl12a;Myl1:	-
6	47523782	47523787	Cul1	+
19	5040604	5040609	B4gat1;AC12	+
15	77015522	77015527	Mb	-
2	75676020	75676025	Nfe2l2	-
18	25132916	25132921	Fhod3	+
11	78255015	78255020	Sdf2	+
11	59407190	59407195	Snap47	-
1	180169686	180169691	Coq8a	-
9	70008038	70008043	Bnip2	+
19	47859475	47859480	Gsto1	+
2	91134438	91134443	Mybpc3	+
11	120646112	120646117	Myadml2	-
10	34284671	34284676	Tspyl1	+
11	102406623	102406628	Slc25a39	-
11	69916746	69916751	Eif5a	-
9	108337114	108337119	Rhoa	+
5	77087205	77087210	Hopx	-
7	79736064	79736069	Pex11a	-
2	108949230	108949235	Gm13910	+
17	5440208	5440213	Tmem242	-
11	100320143	100320148	Eif1	+
10	53344276	53344281	Pln	+
16	10530747	10530752	Dexi	-
6	83057008	83057013	Aup1	+
1	171288760	171288765	Ufc1	-
6	33060068	33060073	Chchd3	-
7	97550491	97550496	Aamdc	-
13	93065225	93065230	Cmya5	-
12	69182811	69182816	Rpl36al	-
9	55482307	55482312	Etfa	-
5	122471187	122471192	Atp2a2	-
9	50753380	50753385	Cryab	+
11	62894575	62894580	Tvp23b	+
14	34527240	34527245	Ldb3	-
14	54941965	54941970	Myh6	-

7	109894559	109894564	Dennd5a	-
9	57531780	57531785	Cox5a	+
15	27544255	27544260	Ank	+
11	70652005	70652010	Pfn1	-
2	181359394	181359399	Arfrp1	-
5	116408652	116408657	Hspb8	-
8	94822913	94822918	Ciapin1	-
13	81672418	81672423	Lysmd3	+
1	43123348	43123353	Fhl2	-
7	101823048	101823053	Inppl1	-
8	124880316	124880321	Gnpat	+
6	95474567	95474572	Suclg2	-
2	120090801	120090806	Ehd4	-
5	97884743	97884748	Antxr2	-
11	54911173	54911178	Tnip1	-
13	33918011	33918016	Serpinb6a	-
19	4004335	4004340	Gm49405;Dc	+
8	85546571	85546576	Dnaja2	-
11	50386092	50386097	Hnrnph1	+
14	8166070	8166075	Pdhb	-
3	14606388	14606393	1810022K09I	-
4	141424993	141424998	Hspb7	+
11	51601325	51601330	Hnrnpab	-
15	101274142	101274147	Nr4a1	+
9	120013941	120013946	Xirp1;Cx3cr1	-
11	69091852	69091857	Vamp2	+
9	57929744	57929749	Ubl7	+
16	91925563	91925568	Atp5o;Atp5o	-
6	136813855	136813860	Wbp11	-
6	32792794	32792799	Chchd3	-
2	104426606	104426611	Hipk3	-
15	97791623	97791628	Slc48a1	+
17	13015243	13015248	Sod2	+
8	83995938	83995943	Prkaca	+
6	32792790	32792795	Chchd3	-
2	69880829	69880834	Mettl5	-
11	46728291	46728296	Gm12174	+
4	136051231	136051236	Rpl11	-
2	73909869	73909874	Atp5g3	-
16	14299374	14299379	Fopnl	-
16	4768519	4768524	Cdip1	-
10	17725161	17725166	Cited2	+
12	80950269	80950274	Srsf5	+
11	20335040	20335045	Gm12033	-
1	86356381	86356386	Ncl	-

4	119278264	119278269	Ybx1	-
1	118609193	118609198	Clasp1	+
10	78272219	78272224	Agpat3	-
2	119607195	119607200	1700020I14R	+
5	117092587	117092592	Suds3	-
4	139485362	139485367	Ubr4	+
9	44333271	44333276	Dpagt1	+
2	174341865	174341870	Gnas	+
4	138439811	138439816	Mul1	+
7	116825446	116825451	Gm4366	-
10	128047744	128047749	Naca	+
8	123892836	123892841	Acta1	-
16	36969467	36969472	Fbxo40	-
11	95031901	95031906	Pdk2	-
14	101696050	101696055	Uchl3	+
15	102116341	102116346	Tns2	+
2	26347906	26347911	Gpsm1	+
5	143902983	143902988	Aimp2	-
10	127281538	127281543	Dctn2	+
3	153922553	153922558	Acadm	-
6	81961930	81961935	Mrpl19	-
3	104656986	104656991	Slc16a1	+
17	17345889	17345894	Oaz1-ps;AC	-
3	150072846	150072851	Rpsa-ps10	-
18	37937179	37937184	Hdac3	-
15	76901072	76901077	Commd5;Gm	+
3	105943727	105943732	Atp5f1	-
13	59634492	59634497	Naa35	+
13	93063469	93063474	Cmya5	-
5	143563481	143563486	Fam220a;Fa	+
11	96818978	96818983	Nfe2l1	-
5	130188862	130188867	Rabgef1	+
13	63302117	63302122	2010111I01R	+
8	123953220	123953225	Abcb10	-
6	125128883	125128888	Chd4	+
11	59212119	59212124	Arf1	-
5	30168544	30168549	Hadhb	+
8	11448691	11448696	Col4a2	+
4	155074703	155074708	Rer1	-
13	12291716	12291721	Actn2	-
9	15262193	15262198	Med17	-
7	28306854	28306859	Timm50	-
10	128048512	128048517	Naca	+
7	25630191	25630196	Bckdha	-
3	152184225	152184230	Dnajb4	-

7	126488748	126488753	Tufm	+
15	81826472	81826477	Tef	+
7	19531138	19531143	Ppp1r37	-
18	16590131	16590136	Cdh2	-
19	4004620	4004625	Gm49405;Dc	+
4	138312098	138312103	Ddost	+
9	31149974	31149979	Aplp2	-
4	14914005	14914010	Pip4p2	+
10	88475406	88475411	Chpt1	-
5	122501796	122501801	Atp2a2	-
3	32577166	32577171	Mfn1	+
9	31149886	31149891	Aplp2	-
13	114316947	114316952	Ndufs4	-
10	128920085	128920090	Rdh5;Bloc1s	-
11	115418437	115418442	Atp5h	-
11	70661123	70661128	Eno3	+
4	41758628	41758633	Galt;Il11ra1	+
11	58316141	58316146	Zfp672	-
2	29816218	29816223	Slc27a4	+
1	169695210	169695215	Rgs5	+
8	111055554	111055559	Aars	+
19	10895369	10895374	Prpf19	+
10	19860797	19860802	Pex7	-
12	113137053	113137058	Mta1	+
3	67394009	67394014	Mlf1	+
8	70508304	70508309	Uba52;Kxd1	-
1	75216209	75216214	Tuba4a	-
19	6262149	6262154	Atg2a	+
9	110980946	110980951	Lrrc2	+
1	36530247	36530252	Ankrd23;Gm	-
7	121076286	121076291	Mettl9	+
19	29021967	29021972	Ak3	-
4	138317307	138317312	Pink1	-
2	103082953	103082958	Apip	+
14	34561472	34561477	Ldb3	-
11	5524864	5524869	Xbp1	+
19	55067848	55067853	Gpam	-
2	131178342	131178347	Cenpb;Spef1	-
10	43532964	43532969	1700021F05I	-
2	90894795	90894800	Ndufs3	-
13	93065345	93065350	Cmya5	-
4	134130161	134130166	Cep85	-
6	71285013	71285018	Krcc1	+
1	37898302	37898307	Mrpl30	+
2	155389554	155389559	Trp53inp2	+

2	155634768	155634773	Trpc4ap	-
8	128733016	128733021	Itgb1	+
17	12975014	12975019	Wtap	-
11	6356188	6356193	Ogdh	+
11	120543132	120543137	Mcrip1	-
1	179687151	179687156	Sccpdh	+
9	57532294	57532299	Cox5a	+
13	91865202	91865207	Ckmt2	-
6	142505542	142505547	Ldhb	-
7	142376717	142376722	Gm49369;Ct	-
8	70700159	70700164	Jund	+
8	124909686	124909691	Egln1	-
7	7282705	7282710	Clcn4	-
7	130981676	130981681	Htra1	+
1	24612203	24612208	Gm28439	-
5	22528265	22528270	Orc5	-
17	23824750	23824755	Elob	-
12	51738732	51738737	Ap4s1	+
2	25560599	25560604	Edf1	+
19	6977001	6977006	Ppp1r14b	+
4	8606762	8606767	Rab2a	+
2	119172322	119172327	Gchfr	+
11	77865076	77865081	Myo18a	+
5	121208488	121208493	Rpl6	+
19	37221810	37221815	March5	+
7	139659911	139659916	Gm4459;Cfa	-
17	24849649	24849654	Fahd1	-
13	63302656	63302661	2010111I01R	+
11	121328245	121328250	Wdr45b	-
19	10058126	10058131	Fads3	+
8	95864414	95864419	Got2	-
9	67028368	67028373	Tpm1	-
14	41061466	41061471	Gm47547	+
15	74958921	74958926	Ly6e	+
5	136248766	136248771	Cux1	-
17	24896086	24896091	Mrps34	+
18	60778496	60778501	Rps14	+
11	97325737	97325742	Mrpl45	+
19	47083509	47083514	Usmg5	-
7	28826217	28826222	Ech1	+
2	152281388	152281393	Csnk2a1	+
10	128086064	128086069	Atp5b	+
19	40292158	40292163	Pdlim1	-
8	40992021	40992026	Mtus1	-
7	128546517	128546522	Bag3	+

10	86732153	86732158	Fabp3-ps1	-
2	132311899	132311904	Cds2	+
13	64186329	64186334	Habp4	+
17	70994682	70994687	Myl12a;Myl1:	-
4	123716005	123716010	Ndufs5	-
19	43499936	43499941	Got1	-
5	30178674	30178679	Hadhb	+
2	119660384	119660389	Ndufaf1	-
13	23531449	23531454	Hist1h4h	+
2	104114564	104114569	Cd59a	+
5	125387708	125387713	Ubc	-
4	63550631	63550636	Atp6v1g1	+
10	77269476	77269481	Pofut2	+
7	80389550	80389555	Furin	-
8	70678036	70678041	Lsm4	+
10	78162512	78162517	D10Jhu81e	-
10	127067464	127067469	Tspan31	-
8	46209396	46209401	Slc25a4	-
11	101580758	101580763	Nbr1	+
2	167608269	167608274	Ube2v1;Gm2	-
7	45458169	45458174	Ftl1;Gm2213	-
12	78843224	78843229	Atp6v1d	-
13	52889114	52889119	Auh	-
11	5192394	5192399	Kremen1	-
2	122028829	122028834	Eif3j1	+
7	19760137	19760142	Bcam	-
11	68210113	68210118	Ntn1	-
2	155250201	155250206	Dynlrb1	+
2	32110053	32110058	Plpp7	+
15	31601641	31601646	Cct5	-
1	183277304	183277309	Brox	-
8	72814793	72814798	Large1	-
10	80833557	80833562	Lingo3	-
8	94393891	94393896	Herpud1	+
3	138555749	138555754	Eif4e	+
2	155848371	155848376	Uqcc1	-
1	155521576	155521581	Gm5532	-
13	63302006	63302011	2010111I01F	+
11	75765540	75765545	Ywhae	+
7	30160556	30160561	Zfp260	-
3	104655991	104655996	Slc16a1	+
11	59205777	59205782	Mrpl55	+
4	109060166	109060171	Nrd1	+
11	48806071	48806076	Rack1	+
14	122384216	122384221	Clybl	+

8	119345700	119345705	Hsbp1	+
9	59679353	59679358	Pkm	+
14	63146680	63146685	Fdft1	-
6	86334137	86334142	Gm10443	+
2	130173165	130173170	Snrpb	-
1	161243579	161243584	Prdx6	-
4	137320001	137320006	Cdc42	-
17	24472555	24472560	Pgp	+
10	80665684	80665689	Mknk2	-
9	98588815	98588820	Mrps22	-
9	31149585	31149590	Aplp2	-
1	16390013	16390018	Stau2	-
2	27020156	27020161	Cacfd1	+
11	70661487	70661492	Eno3	+
13	23531440	23531445	Hist1h4h	+
9	75060251	75060256	Arpp19	+
15	7197005	7197010	Lifr	+
11	70654398	70654403	Pfn1	-
7	45122856	45122861	Rps11	-
1	52904655	52904660	Hibch	+
5	129758010	129758015	Nipsnap2	+
3	96528202	96528207	Hfe2	+
18	32529511	32529516	Gypc	-
9	55488925	55488930	Etfa	-
12	15792008	15792013	Trib2	-
4	107904234	107904239	Cpt2	-
11	96073063	96073068	Atp5g1	-
8	110811425	110811430	Sf3b3	-
1	181903010	181903015	Enah	-
4	119136847	119136852	Slc2a1	+
16	56701181	56701186	Tfg	-
16	59491817	59491822	Riox2	+
2	164018003	164018008	Ywhab	+
5	92444238	92444243	Scarb2	-
6	83109716	83109721	Gm42688;Mı	+
6	133106449	133106454	Smim10l1	+
8	22463186	22463191	Smim19	-
2	30284353	30284358	Dolk	-
13	38196068	38196073	Dsp	+
1	164438319	164438324	Atp1b1	-
15	101274233	101274238	Nr4a1	+
14	56697186	56697191	Mphosph8	+
5	122550375	122550380	Ift81	-
7	16915759	16915764	Calm3	-
10	78162576	78162581	D10Jhu81e	-

9	67028747	67028752	Tpm1	-
11	69036026	69036031	Ctc1	+
4	117932272	117932277	St3gal3	-
10	61702278	61702283	Tysnd1	+
11	96073631	96073636	Atp5g1	-
13	24820784	24820789	Acot13	-
14	19812112	19812117	Rtraf	-
3	32937883	32937888	Usp13	+
5	130188570	130188575	Gm15920;Ra	+
18	9316292	9316297	Ccny	-
9	50645070	50645075	Dlat	-
1	165649763	165649768	Rcsd1	-
4	138439078	138439083	Mul1	+
2	75640124	75640129	Rps6-ps4	+
12	59050295	59050300	Trappc6b	-
19	6987985	6987990	Dnajc4	-
13	70588737	70588742	Ice1	-
3	107663266	107663271	Ahcyl1	-
11	11930691	11930696	Grb10	-
11	53429060	53429065	Uqcrq	-
19	27260987	27260992	AC119982.1	-
18	67405268	67405273	Afg3l2	-
17	47792143	47792148	Tfeb	+
13	33918781	33918786	Serpinb6a	-
16	90342385	90342390	Gm49708	-
15	103344513	103344518	Itga5	-
1	178329333	178329338	Hnrnpu	-
6	128432520	128432525	Fkbp4	-
3	138081209	138081214	1110002E22I	+
18	35806879	35806884	AC141471.2;	+
13	46418860	46418865	Rbm24	+
18	34943282	34943287	Hspa9	-
7	105555404	105555409	Smpd1	+
17	33997425	33997430	H2-K1	-
9	96333278	96333283	Atp1b3	-
1	138484593	138484598	Nek7	-
6	34306612	34306617	Akr1b3	-
8	119410916	119410921	Mlycd	+
7	111077268	111077273	Eif4g2	-
9	107592674	107592679	Ifrd2	+
11	77470051	77470056	Coro6	+
5	144876857	144876862	Smurf1	-
2	28934206	28934211	Gm13394;Cf	-
6	38687752	38687757	Luc7l2	-
3	103056467	103056472	Csde1	+

15	79527863	79527868	Ddx17	-
4	58807756	58807761	Ecpas	-
12	79156303	79156308	Vti1b	-
7	45456320	45456325	Gys1	+
18	80200413	80200418	Rbfa	-
13	100655826	100655831	Taf9;Ak6	+
11	53428955	53428960	Uqcrq	-
5	30955203	30955208	Preb	-
11	117813534	117813539	Syngr2;Gm2	+
3	34069771	34069776	Fxr1	+
14	30999839	30999844	Spcs1	-
7	102110991	102110996	Art1	+
6	72369164	72369169	Vamp5	-
19	5647379	5647384	Rela	+
10	71228029	71228034	Tfam	-
4	49512611	49512616	Mrpl50	-
4	49585626	49585631	Tmem246	-
1	135847762	135847767	Tnnt2	+
14	8166281	8166286	Pdhb	-
8	85546600	85546605	Dnaja2	-
6	85134102	85134107	Spr	-
3	27243879	27243884	Nceh1	+
4	15929792	15929797	Decr1	-
11	94213869	94213874	Tob1	+
17	30135397	30135402	Zfand3	+
18	84879612	84879617	Cyb5a	+
9	50634722	50634727	Dlat	-
14	54961023	54961028	Myh6	-
4	116636599	116636604	Akr1a1	-
9	59390199	59390204	Arih1	-
15	38488475	38488480	Azin1	-
10	95548128	95548133	Nudt4	-
4	134163902	134163907	Cep85	+
7	16738500	16738505	Ap2s1	+
4	15918601	15918606	Decr1	-
11	101635855	101635860	Rdm1	+
15	25971973	25971978	Retreg1	+
15	5118583	5118588	Rpl37	+
3	101576463	101576468	Atp1a1	-
6	24604284	24604289	Lmod2	+
14	51905816	51905821	Ndrg2	-
5	100037929	100037934	Hnrnpdl	-
7	99345795	99345800	Serpinh1	-
15	76344886	76344891	Cyc1	+
3	101583748	101583753	Atp1a1	-

7	141459802	141459807	Pnpla2	+
3	90280480	90280485	Dennd4b	+
9	120959591	120959596	Ctnnb1	+
5	24581534	24581539	Abcf2	-
7	111072727	111072732	Eif4g2	-
5	91625829	91625834	Parm1	+
4	99929764	99929769	Pgm1	+
9	65326822	65326827	Clpx	+
17	44038734	44038739	Rcan2	+
17	47686970	47686975	Tomm6;Gm2	-
7	80094927	80094932	Idh2	-
5	33372550	33372555	Maea	+
19	36117933	36117938	Ankrd1	-
3	116489198	116489203	Rtca	-
14	74742104	74742109	Esd	+
10	42182124	42182129	Foxo3	-
1	75181390	75181395	Gm29253;At	-
2	28934497	28934502	Gm13394;Cf	-
14	24493415	24493420	Rps24	+
11	59185297	59185302	Guk1	-
19	18717854	18717859	D030056L22	+
2	132529655	132529660	Gpcpd1	-
1	189883869	189883874	Smyd2	-
15	57871629	57871634	Derl1	-
1	134424834	134424839	Adipor1	+
1	156007143	156007148	Tor1aip1	-
9	52088504	52088509	Rdx	+
11	58392860	58392865	Gm12251	-
12	44296005	44296010	Pnpla8	+
11	40749986	40749991	Ccng1	-
2	103454725	103454730	Cat	-
3	116570943	116570948	Lrrc39	+
3	107897516	107897521	Gstm5	+
2	156312141	156312146	Scand1	-
9	120124145	120124150	Slc25a38	+
8	95712337	95712342	Ndrg4	+
19	10905101	10905106	Prpf19	+
1	151363499	151363504	Ivns1abp	+
7	130764336	130764341	Tacc2	+
7	139578593	139578598	Inpp5a	+
4	136274790	136274795	Tcea3	+
2	156873197	156873202	Rab5if	+
16	36963762	36963767	Fbxo40	-
3	95663035	95663040	Mcl1	+
8	95714596	95714601	Ndrg4	+

11	58998085	58998090	Obscn	-
16	33058805	33058810	Rpl35a	+
2	108950541	108950546	Gm13910	+
11	5192321	5192326	Kremen1	-
7	13032793	13032798	Chmp2a	-
7	45917066	45917071	Tmem143	+
2	71900952	71900957	Pdk1	+
11	55500363	55500368	G3bp1	+
2	26922042	26922047	Surf4	-
4	41712459	41712464	Rpp25l	-
6	17340459	17340464	Cav1	+
19	24261873	24261878	Fxn	-
4	116033062	116033067	Nsun4	-
1	180802771	180802776	H3f3a	-
12	113144857	113144862	Crip2	+
6	71851825	71851830	Immt	+
18	34349656	34349661	Reep5	-
13	21501237	21501242	Gm11273	-
4	134325637	134325642	Trim63	+
11	21330848	21330853	Ugp2	-
9	67027935	67027940	Tpm1	-
5	115111398	115111403	Acads	-
3	153922928	153922933	Acadm	-
1	58991033	58991038	Stradb	+
4	103564890	103564895	Gm12715	+
2	75640928	75640933	Rps6-ps4	+
9	4386003	4386008	Msantd4	+
8	13256508	13256513	Dcun1d2	-
11	21329913	21329918	Ugp2	-
4	135962460	135962465	Hmgcl	+
19	6389176	6389181	Pygm	+
17	56258203	56258208	Fem1a	+
4	137569741	137569746	Hspg2	+
10	56390270	56390275	Gja1	+
2	71324058	71324063	Slc25a12	-
5	129887208	129887213	Chchd2;Phkg	-
2	155846937	155846942	Uqcc1	-
5	36471442	36471447	Grpel1	+
9	107538351	107538356	Tmem115	+
17	35015738	35015743	Vars	+
5	24441770	24441775	Fastk	-
2	25223237	25223242	Tubb4b	-
4	14914965	14914970	Pip4p2	+
4	15918973	15918978	Decr1	-
8	114133680	114133685	Nudt7	+

10	33196213	33196218	Trdn	+
14	8172940	8172945	Pdhb	-
10	81208630	81208635	Atcayos	+
7	30554809	30554814	Hspb6	+
11	21557559	21557564	Mdh1	-
13	30545274	30545279	Uqcrfs1	-
17	44279519	44279524	Clic5	+
7	128011022	128011027	Trim72	+
6	125362035	125362040	Tnfrsf1a	+
14	31259087	31259092	Bap1	+
8	110882076	110882081	Cog4	+
12	32850861	32850866	Nampt	+
2	163336699	163336704	Jph2	-
5	30119628	30119633	Hadha	-
8	82344417	82344422	Il15	-
2	153009618	153009623	Pdrg1	-
8	71251273	71251278	Haus8	-
11	53261619	53261624	Hspa4	-
16	31457535	31457540	Bdh1	+
18	35244748	35244753	Ctnna1	+
11	70661105	70661110	Eno3	+
11	43424755	43424760	Pttg1	-
1	75181738	75181743	Gm29253;At[illegible]	-
11	58315274	58315279	Zfp672	-
13	58126051	58126056	Hnrnpa0	-
3	90499825	90499830	Chtop	-
6	124704509	124704514	Emg1	-
16	56034626	56034631	Trmt10c	-
4	59022845	59022850	Dnajc25;Gm[illegible]	+
19	45005678	45005683	Mrpl43	-
14	76506721	76506726	Tsc22d1	+
19	44555043	44555048	Ndufb8	-
2	61783849	61783854	Psmd14	+
9	120014412	120014417	Xirp1;Cx3cr1	-
13	91022591	91022596	Atg10	-
5	122113443	122113448	Myl2	+
8	45827564	45827569	Sorbs2	+
17	78947040	78947045	Ndufaf7	+
19	3385364	3385369	Cpt1a	+
14	101634191	101634196	Commd6	-
4	75277587	75277592	Dmac1	-
17	34601485	34601490	Rnf5	-
8	85260970	85260975	Vps35	-
3	90499070	90499075	Chtop	-
3	90499793	90499798	Chtop	-

16	22871749	22871754	Dnajb11	+
7	114269798	114269803	Psma1	-
15	90233167	90233172	Alg10b	+
19	43503031	43503036	Got1	-
11	70978708	70978713	C1qbp	-
1	170156973	170156978	Uap1	-
4	149725988	149725993	Tmem201	-
5	88659887	88659892	Grsf1	-
3	142302713	142302718	Pdlim5	-
5	72159527	72159532	Commd8	-
1	73914429	73914434	Tns1	-
10	60300861	60300866	Psap	+
16	16309342	16309347	Yars2	+
11	6419117	6419122	Ppia	+
2	127298236	127298241	Stard7	+
2	84829870	84829875	Timm10	+
8	22568966	22568971	Slc20a2	+
5	135908717	135908722	Ywhag	-
7	100327403	100327408	Ppme1	-
8	94575023	94575028	Fam192a	-
7	90468975	90468980	Tmem126b	-
19	21281638	21281643	Zfand5	+
7	80100850	80100855	Idh2	-
11	120345744	120345749	Actg1	-
2	163337811	163337816	Jph2	-
10	111508159	111508164	Phlda1	+
9	7932177	7932182	Yap1	-
19	34248452	34248457	Acta2	-
15	4128885	4128890	Oxct1	+
9	48479320	48479325	Rexo2	-
16	38362875	38362880	Popdc2	+
2	181188072	181188077	Ppdpf	+
6	71124622	71124627	Rpl34-ps1	+
16	10530554	10530559	Dexi	-
13	11553924	11553929	Ryr2	-
14	63199922	63199927	Gata4	-
2	26395694	26395699	Pmpca	+
2	74876361	74876366	Mtx2	+
19	4194476	4194481	Ppp1ca	+
9	50756559	50756564	Cryab	+
15	51786813	51786818	Eif3h	-
8	27275371	27275376	Eif4ebp1	+
4	107907078	107907083	Cpt2	-
4	43531919	43531924	Tln1	-
16	4766173	4766178	Hmox2	+

17	45660369	45660374	Tmem63b	-
8	68906127	68906132	Lpl	+
15	10995125	10995130	Amacr	+
3	138081293	138081298	1110002E22I	+
11	69994888	69994893	Gabarap	+
16	23113226	23113231	Eif4a2	+
2	50280072	50280077	Mmadhc	-
7	48832640	48832645	Csrp3	-
2	173033793	173033798	Rbm38	+
11	120104653	120104658	Slc38a10	-
1	171129761	171129766	Sdhc	-
15	58176578	58176583	Fbxo32	-
9	67220941	67220946	Tln2	-
4	136271272	136271277	Tcea3	+
11	45909959	45909964	Clint1	+
10	80615384	80615389	Scamp4;Gm	+
6	128438424	128438429	Fkbp4	-
11	80266019	80266024	Rhot1	+
2	118881192	118881197	Ivd	+
4	108861856	108861861	Txndc12	+
7	44493254	44493259	Emc10	-
6	88013684	88013689	Rab7	-
4	123350641	123350646	Macf1	-
2	35180260	35180265	Rab14	-
12	17280402	17280407	Pdia6	+
9	66511773	66511778	Fbxl22	-
2	30402364	30402369	Crat	-
9	96471791	96471796	Rnf7	-
8	114151666	114151671	Nudt7	+
19	55068487	55068492	Gpam	-
4	140515494	140515499	Arhgef10l	-
2	164834907	164834912	Ctsa	+
17	75537519	75537524	Fam98a	-
8	71475040	71475045	Dda1	+
5	129754856	129754861	Nipsnap2	+
11	54870479	54870484	Hint1	+
1	79781077	79781082	Mrpl44	+
7	5097962	5097967	Epn1	+
6	112472706	112472711	Cav3	+
11	86602048	86602053	Vmp1	-
17	24722211	24722216	Ndufb10	-
4	129309952	129309957	Rbbp4	-
16	91615298	91615303	Dnajc28	-
18	36791261	36791266	Hars2	+
10	117060751	117060756	Cct2	-

2	121461412	121461417	Mfap1b	-
6	72566197	72566202	Elmod3	-
10	71255675	71255680	Ube2d1	-
7	100486300	100486305	Ucp3	+
17	12913896	12913901	Mrpl18	-
6	142588285	142588290	Abcc9	-
15	76345812	76345817	Cyc1	+
9	54714701	54714706	Dnaja4	+
15	51786764	51786769	Eif3h	-
14	25700075	25700080	Ppif	+
6	113336400	113336405	Camk1	-
11	119899129	119899134	Rptor	+
1	172272832	172272837	Atp1a2	-
9	98588204	98588209	Copb2	+
7	108941588	108941593	Eif3f	+
8	72586507	72586512	Tmem38a	+
15	82347997	82348002	Smdt1	+
11	94124943	94124948	Spag9	+
16	38377952	38377957	Popdc2	+
7	116825513	116825518	Gm4366	-
17	20961693	20961698	Ppp2r1a	+
3	57842495	57842500	Pfn2	-
4	49585822	49585827	Tmem246	-
9	54954709	54954714	Psma4	+
18	36683750	36683755	Slc35a4	+
5	100363442	100363447	Sec31a	-
10	128353087	128353092	Cs	+
12	32834987	32834992	Nampt	+
10	81265666	81265671	Mrpl54	-
11	106781845	106781850	Ddx5	-
3	152237293	152237298	Nexn	-
7	81792249	81792254	Btbd1	-
3	36449069	36449074	Anxa5	-
10	128548218	128548223	Rpl41	-
2	121548302	121548307	Frmd5	-
10	53345060	53345065	Pln	+
7	46083905	46083910	Nomo1	+
7	84109602	84109607	Abhd17c	-
4	43028153	43028158	Stoml2	-
4	45398870	45398875	Slc25a51	-
2	131936475	131936480	Prnp;Prn	+
11	120457953	120457958	Oxld1	-
13	93089266	93089271	Cmya5	-
18	80192361	80192366	Rbfa	-
2	142619842	142619847	Kif16b	-

14	32178660	32178665	Ncoa4	+
14	31211381	31211386	Tnnc1	+
5	30163689	30163694	Hadhb	+
19	10903015	10903020	Prpf19	+
8	95865004	95865009	Got2	-
4	73942480	73942485	2310002L09I	-
9	24722493	24722498	Tbx20	-
6	72153670	72153675	St3gal5	+
12	108107497	108107502	Setd3	-
2	156764300	156764305	Dlgap4	+
6	72433324	72433329	Mat2a	-
18	36766638	36766643	Hars	-
1	86909020	86909025	Gm6136	-
1	178448721	178448726	Efcab2	+
3	90061962	90061967	4933434E20I	+
5	5782852	5782857	Gm15459	-
8	71586081	71586086	Slc27a1	+
7	114047027	114047032	Rras2	-
4	86855958	86855963	Rps6	-
3	142302782	142302787	Pdlim5	-
3	153922664	153922669	Acadm	-
15	44435621	44435626	Eny2	+
4	152271781	152271786	Acot7	+
9	120014406	120014411	Xirp1;Cx3cr1	-
8	94394793	94394798	Herpud1	+
15	77015790	77015795	Mb	-
17	84712829	84712834	Lrpprc	-
19	3289067	3289072	Mrpl21	+
10	53345200	53345205	Pln	+
1	75215318	75215323	Tuba4a	-
9	118075126	118075131	Cmc1	-
8	46209339	46209344	Slc25a4	-
8	46207206	46207211	Slc25a4	-
10	95548994	95548999	Nudt4	-
5	135908652	135908657	Ywhag	-
1	63159791	63159796	Ndufs1	-
4	119281581	119281586	Ybx1	-
11	63963496	63963501	Cox10	-
17	87433664	87433669	Calm2	-
3	138555506	138555511	Eif4e	+
1	169694892	169694897	Rgs5	+
14	46776079	46776084	Cnih1	-
3	41068491	41068496	Pgrmc2	-
9	110384794	110384799	Scap	+
6	82725247	82725252	Hk2	-

8	85539240	85539245	Dnaja2	-
13	30540507	30540512	Uqcrfs1	-
3	97690642	97690647	Pde4dip	-
8	85538191	85538196	Dnaja2	-
1	92859136	92859141	Gpc1	+
5	5783141	5783146	Gm15459	-
3	101576279	101576284	Atp1a1	-
11	70661419	70661424	Eno3	+
5	125387313	125387318	Ubc	-
11	40748616	40748621	Ccng1	-
5	129758158	129758163	Nipsnap2	+
3	122925703	122925708	1810037I17R	+
14	54614370	54614375	Psmb5	-
3	135465424	135465429	Ube2d3	+
9	55495637	55495642	Etfa	-
19	4004555	4004560	Gm49405;Dc	+
11	52388699	52388704	Vdac1	+
2	127297176	127297181	Stard7	+
8	94394598	94394603	Herpud1	+
3	97690663	97690668	Pde4dip	-
8	94394899	94394904	Herpud1	+
2	38588150	38588155	Psmb7	-
9	105076997	105077002	Mrpl3	+
17	15476064	15476069	Psmb1	-
14	103081166	103081171	Fbxl3	-
7	73776837	73776842	Fam174b	+
3	116744197	116744202	Agl	-
9	67028135	67028140	Tpm1	-
14	37135232	37135237	Ghitm	-
3	145647516	145647521	Ccn1	-
11	106780890	106780895	Ddx5	-
4	149234415	149234420	Kif1b	-
2	180177274	180177279	Lama5	-
5	146261183	146261188	Cdk8	+
5	36601492	36601497	Gm42936;D5	-
8	94394836	94394841	Herpud1	+
9	66514489	66514494	Fbxl22	-
7	45916721	45916726	Tmem143	+
2	118476104	118476109	Srp14	-
11	98800517	98800522	Msl1	+
9	121785239	121785244	Hhatl	-
17	86997154	86997159	Rhoq	+
7	105379017	105379022	Fam160a2	-
14	101696076	101696081	Uchl3	+
5	136965975	136965980	Fis1	+

3	57834714	57834719	Rnf13	+
2	125673921	125673926	Eid1	+
18	35590662	35590667	Matr3	+
11	60952758	60952763	Map2k3	+
11	5525034	5525039	Xbp1	+
17	26505954	26505959	Dusp1	-
7	45807703	45807708	Cyth2	-
1	66830993	66830998	Acadl	-
9	59656630	59656635	Pkm	+
1	54987264	54987269	Sf3b1	-
5	97885094	97885099	Antxr2	-
6	86513995	86514000	Gm44386	-
6	72957949	72957954	Tmsb10	-
5	88660119	88660124	Grsf1	-
5	116014076	116014081	Prkab1	-
5	73310375	73310380	Ociad1	+
10	80055023	80055028	Gpx4	+
15	44433915	44433920	Eny2	+
14	55581436	55581441	Psme1	+
13	114351491	114351496	Ndufs4	-
14	79461146	79461151	Wbp4	-
7	142376614	142376619	Gm49369;Ct	-
18	11085022	11085027	Gata6	+
15	89416904	89416909	Gm44502;Cp	-
15	89416900	89416905	Gm44502;Cp	-
4	24901767	24901772	Ndufaf4	+
14	66310366	66310371	Trim35	+
9	40804634	40804639	Hspa8	+
11	78523044	78523049	Tnfaip1	-
11	75759404	75759409	Ywhae	+
10	7768126	7768131	Ginm1	-
4	45397324	45397329	Slc25a51	-
1	156366384	156366389	Gm2000	+
5	36471420	36471425	Grpel1	+
17	26506392	26506397	Dusp1	-
10	53345887	53345892	Pln	+
2	120089275	120089280	Ehd4	-
18	35650443	35650448	SPATA24;Pr	-
14	73595819	73595824	Sucla2	+
11	6356405	6356410	Ogdh	+
1	182467660	182467665	Capn2	-
17	24896180	24896185	Mrps34	+
15	103344772	103344777	Itga5	-
4	132854514	132854519	Stx12	-
17	34956928	34956933	Hspa1b	-

10	30611688	30611693	Hint3	-
3	27244458	27244463	Nceh1	+
11	116172759	116172764	Acox1	-
15	81848646	81848651	Tob2	-
14	21845792	21845797	Vdac2	+
10	128044863	128044868	Naca	+
5	122550868	122550873	Ift81	-
2	30443293	30443298	Ptpa	+
9	44094840	44094845	Usp2	+
4	129488156	129488161	Bsdc1	+
17	48432539	48432544	Apobec2	-
13	6581408	6581413	Pfkp	-
6	52546495	52546500	Hibadh	-
13	11553310	11553315	Ryr2	-
15	76171026	76171031	Plec	-
1	157420110	157420115	2810025M15	+
11	70664196	70664201	Spag7	-
3	8802239	8802244	Mrps28	-
17	35910655	35910660	Mrps18b	-
3	57820523	57820528	Rnf13	+
2	120089880	120089885	Ehd4	-
2	166074623	166074628	Sulf2	-
7	44485196	44485201	5430431A17I	+
1	55078343	55078348	Hspd1	-
6	5273083	5273088	Pon2	-
9	122368019	122368024	Abhd5	+
14	34561519	34561524	Ldb3	-
11	100372365	100372370	Jup	-
17	26506555	26506560	Dusp1	-
19	6299305	6299310	Ehd1	+
7	28258332	28258337	Dyrk1b	-
12	108106992	108106997	Setd3	-
14	54942028	54942033	Myh6	-
15	79672615	79672620	Tomm22	+
6	73260526	73260531	Suclg1	+
3	103057382	103057387	Csde1	+
18	60776971	60776976	Rps14	+
6	66875592	66875597	Gm9794	-
6	72433343	72433348	Mat2a	-
15	75891808	75891813	Naprt	-
6	87999590	87999595	Rab7	-
11	54911150	54911155	Tnip1	-
9	31149636	31149641	Aplp2	-
2	162933452	162933457	Srsf6	+
17	23675581	23675586	Tnfrsf12a	-

18	38262138	38262143	Dele1	+
13	91861766	91861771	Ckmt2	-
5	122456933	122456938	Atp2a2	-
2	155277746	155277751	Map1lc3a	+
17	6038039	6038044	Synj2	+
6	125307015	125307020	Ltbr	-
13	21501334	21501339	Gm11273	-
5	17814789	17814794	Cd36	-
16	31948151	31948156	0610012G03	-
9	121850191	121850196	Higd1a	-
11	51685633	51685638	0610009B22I	-
2	120090037	120090042	Ehd4	-
4	40804847	40804852	B4galt1	-
15	31458162	31458167	March6	-
11	95772304	95772309	Zfp652	+
16	18407520	18407525	Comt	-
13	55344049	55344054	Lman2	-
13	93045969	93045974	Cmya5	-
13	119336333	119336338	Nnt;Nnt	-
17	75537166	75537171	Fam98a	-
4	15931126	15931131	Decr1	-
7	114264912	114264917	Psma1	-
11	40684721	40684726	Mat2b	-
1	135848048	135848053	Tnnt2	+
1	172196209	172196214	Dcaf8	+
10	77598700	77598705	Pttg1ip	+
17	56721423	56721428	Ndufa11	+
16	18826265	18826270	Ufd1	+
2	120089782	120089787	Ehd4	-
15	25985095	25985100	Zfp622	+
3	96560925	96560930	Txnip	+
6	122457116	122457121	Gm8430	+
19	45005194	45005199	Mrpl43	-
12	17279655	17279660	Pdia6	+
11	5803528	5803533	Pgam2	-
17	26792197	26792202	Bnip1	+
4	33245754	33245759	Pnrc1	-
2	67525104	67525109	Xirp2	+
11	82943036	82943041	Unc45b	+
11	51685880	51685885	0610009B22I	-
2	164464820	164464825	Sys1;Gm204	+
4	141424135	141424140	Hspb7	+
12	8499454	8499459	Rhob	-
15	76622249	76622254	Vps28	-
8	22583673	22583678	Vdac3	-

8	11199266	11199271	Col4a1	-
6	124810725	124810730	Tpi1	-
11	94971283	94971288	Sgca	-
7	121076514	121076519	Mettl9	+
8	83573245	83573250	Tecr	-
7	143066273	143066278	Cd81	+
4	132553387	132553392	Dnajc8	+
8	79677073	79677078	Otud4	+
9	107598593	107598598	Sema3b	-
10	63015607	63015612	Hnrnph3	-
10	81123818	81123823	Map2k2	+
7	141882084	141882089	Tollip	-
16	31449878	31449883	Bdh1	+
8	121597253	121597258	Map1lc3b;Gr	+
18	65981080	65981085	Lman1	-
6	85460020	85460025	Cct7	+
12	17550705	17550710	Odc1	+
7	79134084	79134089	Mfge8	-
8	83570977	83570982	Ndufb7	+
12	84258541	84258546	Gm5436	-
5	135012153	135012158	Abhd11	+
15	81950542	81950547	Csdc2	+
17	35892707	35892712	2310061I04F	-
2	25505112	25505117	Fbxw5	+
12	86964467	86964472	Cipc	+
10	127067717	127067722	Tspan31	-
18	35222129	35222134	Ctnna1	+
14	21837801	21837806	Vdac2	+
10	80395074	80395079	Mbd3	-
15	9108919	9108924	Nadk2	+
2	120090643	120090648	Ehd4	-
2	132864154	132864159	Crls1	+
13	24813175	24813180	BC005537	+
1	166098453	166098458	Dusp27	-
7	19091691	19091696	Dmpk	+
15	43511779	43511784	Emc2	+
7	28256787	28256792	Dyrk1b	-
17	56281421	56281426	Plin3	-
11	3917421	3917426	Tcn2	-
9	87077009	87077014	Cyb5r4	+
6	14714525	14714530	Ppp1r3a	-
4	149234231	149234236	Kif1b	-
2	6189559	6189564	Echdc3	-
7	120120261	120120266	Tmem159	+
4	116636634	116636639	Akr1a1	-

17	8297587	8297592	Mpc1	+
11	120484736	120484741	Mrpl12	+
12	69803971	69803976	Map4k5	-
11	117813483	117813488	Syngr2;Gm2	+
11	40679349	40679354	Mat2b	-
1	74282484	74282489	Aamp	-
7	141493027	141493032	Tspan4	+
11	68904520	68904525	Rpl26	+
10	79711708	79711713	Bsg	+
9	66955810	66955815	Lactb	-
17	7302564	7302569	Rps6ka2	+
5	5782322	5782327	Gm15459	-
14	73363116	73363121	Itm2b	-
3	102145907	102145912	Casq2	+
9	79770442	79770447	Tmem30a	-
7	28041892	28041897	Psmc4	-
10	95542280	95542285	Ube2n	+
2	73141975	73141980	Ola1	-
4	144927376	144927381	Dhrs3	+
2	127242692	127242697	Ciao1	-
4	139280528	139280533	Capzb	+
17	34970178	34970183	Hspa1a	-
1	172279449	172279454	Atp1a2	-
16	8857958	8857963	1810013L24I	+
4	40960964	40960969	Chmp5	+
19	6982726	6982731	Vegfb	-
7	46855058	46855063	Ldha	+
4	141620096	141620101	Slc25a34	-
19	47864622	47864627	Gsto1	+
5	30120153	30120158	Hadha	-
9	66511830	66511835	Fbxl22	-
7	37960033	37960038	Uri1	-
4	120667365	120667370	Cited4	+
16	95720516	95720521	Ets2	+
9	113919364	113919369	Clasp2	+
1	51302114	51302119	Cavin2	+
19	3910922	3910927	Ndufs8	-
11	104584002	104584007	Myl4	+
3	96560766	96560771	Txnip	+
6	34310947	34310952	Akr1b3	-
2	181856730	181856735	Pcmtd2	+
11	120488154	120488159	Mrpl12	+
4	136268081	136268086	Tcea3	+
11	50202521	50202526	Sqstm1	-
1	134756092	134756097	Ppp1r12b	-

2	155848013	155848018	Uqcc1	-
6	134715959	134715964	Dusp16	-
7	140101348	140101353	Fuom	-
6	17287053	17287058	Cav2	+
13	24818121	24818126	Acot13	-
9	107535125	107535130	Tmem115	+
8	109672063	109672068	Ist1	-
5	31078101	31078106	Cad	+
9	50344501	50344506	Rpl10-ps3	-
17	49993878	49993883	Rftn1	-
11	88211155	88211160	Mrps23	+
11	46971697	46971702	Sgcd	-
12	8936781	8936786	Laptm4a	+
18	64458067	64458072	Fech	-
5	25850267	25850272	Actr3b	+
16	90341737	90341742	Gm49708	-
2	145908590	145908595	Naa20	+
6	88005097	88005102	Rab7	-
11	86689972	86689977	Ptrh2	+
17	65849053	65849058	Ralbp1	-
17	81386338	81386343	Slc8a1	-
3	133367905	133367910	Ppa2	+
10	63317492	63317497	Herc4	+
2	32401588	32401593	Ptges2	+
7	140105783	140105788	Echs1	-
5	17820549	17820554	Cd36	-
2	4936006	4936011	Phyh	+
19	5647373	5647378	Rela	+
4	138800352	138800357	Pla2g5	-
9	107656149	107656154	Slc38a3	-
14	65974906	65974911	Clu	+
12	72793918	72793923	Ppm1a	+
2	35307635	35307640	Gsn	+
5	112692213	112692218	Myo18b	-
7	111072256	111072261	Eif4g2	-
8	124910268	124910273	Egln1	-
19	45005431	45005436	Mrpl43	-
11	116289128	116289133	Exoc7	-
17	24849178	24849183	Fahd1	-
4	126233243	126233248	Map7d1	-
7	25666268	25666273	Exosc5	+
18	80293028	80293033	Pqlc1	-
1	75216049	75216054	Tuba4a	-
2	152281268	152281273	Csnk2a1	+
19	24280470	24280475	Fxn	-

11	3342401	3342406	Pik3ip1	+
8	13256469	13256474	Dcun1d2	-
14	54614226	54614231	Psmb5	-
1	181242024	181242029	Rpl35a-ps2	-
7	109736497	109736502	Tmem9b	-
8	71374502	71374507	Nr2f6	-
13	46677133	46677138	Fam8a1	+
19	32820093	32820098	Pten	+
2	76710217	76710222	Ttn	-
2	30345095	30345100	Sh3glb2	-
2	152148884	152148889	Tcf15	+
12	17548015	17548020	Odc1	+
7	5092863	5092868	Epn1	+
5	24581499	24581504	Abcf2	-
7	118529617	118529622	Coq7	-
2	127245724	127245729	Ciao1	-
2	25504267	25504272	Fbxw5	+
7	31052146	31052151	Fxyd1	-
12	69803872	69803877	Map4k5	-
19	57033575	57033580	Ablim1	-
7	48830533	48830538	Csrp3	-
10	13009010	13009015	Sf3b5	+
7	45127223	45127228	Rpl13a;Gm4	-
12	84431501	84431506	Aldh6a1	-
8	27053904	27053909	Plpbp	+
5	122106762	122106767	Myl2	+
1	74267792	74267797	Arpc2	+
2	174346019	174346024	Gnas	+
1	155521225	155521230	Gm5532	-
1	131053751	131053756	Mapkapk2	-
9	64178480	64178485	Rpl4	+
11	116174686	116174691	Acox1	-
4	33049970	33049975	Ube2j1	+
3	95034689	95034694	Psmd4	-
18	34336459	34336464	Srp19	+
18	80292738	80292743	Pqlc1	-
17	25839230	25839235	Rhot2;Gm20	-
16	58470244	58470249	St3gal6	-
2	76709901	76709906	Ttn	-
12	85343226	85343231	Tmed10	-
11	21565895	21565900	Mdh1	-
3	107663273	107663278	Ahcyl1	-
11	50207257	50207262	Sqstm1	-
2	163466646	163466651	Fitm2	-
14	13949248	13949253	Thoc7	-

14	73595451	73595456	Sucla2	+
5	30173935	30173940	Hadhb	+
19	5843563	5843568	Neat1	-
6	134715850	134715855	Dusp16	-
4	15286666	15286671	Tmem64	+
19	8772134	8772139	Tmem223	+
6	133106559	133106564	Smim10l1	+
4	107907245	107907250	Cpt2	-
12	105838582	105838587	Papola	+
14	69717237	69717242	Chmp7	-
9	65082305	65082310	Dpp8	+
16	23113870	23113875	Eif4a2	+
3	121760214	121760219	Abcd3	-
13	119335743	119335748	Nnt;Nnt	-
11	101259120	101259125	Vps25;Ramp	+
3	101586278	101586283	Atp1a1	-
11	80266686	80266691	Rhot1	+
15	27594170	27594175	Ank	+
10	7768394	7768399	Ginm1	-
13	91859880	91859885	Ckmt2	-
5	135011884	135011889	Abhd11	+
15	31590924	31590929	Cct5	-
7	80099093	80099098	Idh2	-
2	75639317	75639322	Rps6-ps4	+
11	59012385	59012390	Obscn	-
11	23488673	23488678	Usp34	+
17	74200851	74200856	Memo1	-
19	36733852	36733857	Ppp1r3c	-
12	8938636	8938641	Laptm4a	+
8	71404220	71404225	Babam1	+
4	9451459	9451464	Asph	-
2	10063328	10063333	Atp5c1	-
7	45825691	45825696	Grwd1	-
17	45698262	45698267	Mrpl14	+
14	79145374	79145379	Vwa8	-
5	143506684	143506689	Rac1	-
19	21281011	21281016	Zfand5	+
11	95491902	95491907	Spop	+
5	115298517	115298522	Dynll1	-
17	13019427	13019432	Sod2	+
8	116982534	116982539	Gcsh	-
2	154694005	154694010	Chmp4b	+
15	82348034	82348039	Smdt1	+
7	45457972	45457977	Ftl1	-
14	75848205	75848210	Tpt1	+

1	24615424	24615429	Gm28661	-
11	98029503	98029508	Rpl19	+
3	95989172	95989177	Plekho1	-
12	108539337	108539342	Eml1	+
7	132557559	132557564	Oat;Fgfr2	-
13	62083117	62083122	Gm48228	-
5	129718417	129718422	Mrps17	+
9	71579670	71579675	Myzap	-
4	140972952	140972957	Sdhb	+
15	98127743	98127748	Pfkm	+
1	165461352	165461357	Mpc2	+
17	81387999	81388004	Slc8a1	-
11	59791946	59791951	Flcn	-
2	32619829	32619834	St6galnac6	+
4	81278902	81278907	Mpdz	-
2	91135831	91135836	Mybpc3	+
2	125257502	125257507	Dut	+
1	161244315	161244320	Prdx6	-
1	161244218	161244223	Prdx6	-
5	35099182	35099187	Lrpap1	-
5	45511949	45511954	Lap3	+
2	164792531	164792536	Snx21	+
2	153009729	153009734	Pdrg1	-
18	34938283	34938288	Hspa9	-
12	105040437	105040442	Glrx5	+
1	82751688	82751693	Mff	+
2	174461103	174461108	Atp5e	-
9	50636190	50636195	Dlat	-
2	163726064	163726069	Pkig	+
8	61503290	61503295	Cbr4	+
18	80211677	80211682	Gm16286;Tx	+
1	118608946	118608951	Clasp1	+
5	41625688	41625693	Rab28	-
13	91859201	91859206	Ckmt2	-
2	75640757	75640762	Rps6-ps4	+
4	116692885	116692890	Prdx1	+
1	92920163	92920168	Rnpepl1	+
6	125130253	125130258	Chd4	+
12	113145284	113145289	Crip2	+
5	122104899	122104904	Myl2	+
12	110692431	110692436	Hsp90aa1	-
4	154158062	154158067	Tprgl	-
2	84442051	84442056	Tfpi	-
6	72609712	72609717	Tgoln1	-
6	86525235	86525240	Pcbp1	-

17	31531122	31531127	Ndufv3	+
11	75704299	75704304	Crk	+
2	150831142	150831147	Pygb	+
11	70660871	70660876	Eno3	+
4	99964726	99964731	Pgm1	+
16	64766601	64766606	4930453N24	-
11	52389226	52389231	Vdac1	+
9	72749434	72749439	Nedd4	+
2	76708663	76708668	Ttn	-
19	5279579	5279584	Sf3b2	-
11	77530793	77530798	Taok1	-
5	30184461	30184466	Hadhb	+
12	103417581	103417586	Ddx24	-
12	78861443	78861448	Atp6v1d	-
11	93952855	93952860	Nme2;Gm20	-
2	84765783	84765788	Serping1	-
13	86045844	86045849	Cox7c	-
1	30941986	30941991	Ptp4a1;Ptp4a	-
18	34904749	34904754	Etf1	-
8	11834666	11834671	Arhgef7	+
19	8880360	8880365	Uqcc3	-
10	128048069	128048074	Naca	+
10	81180206	81180211	Eef2	+
19	23973336	23973341	Fam189a2	-
3	152185078	152185083	Dnajb4	-
16	3902007	3902012	Naa60;Gm2(	+
6	72433624	72433629	Mat2a	-
5	124570859	124570864	Eif2b1	-
2	150619342	150619347	Acss1	-
3	51256060	51256065	Elf2	-
17	24856637	24856642	Hagh	+
17	27125197	27125202	Uqcc2	-
6	35261329	35261334	1810058I24R	+
5	121445002	121445007	Erp29	-
1	45347177	45347182	Col3a1	+
18	75009847	75009852	BC031181	+
9	121857521	121857526	Higd1a	-
8	111622772	111622777	Znrf1	+
14	34567410	34567415	Ldb3	-
5	20886717	20886722	Tmem60	+
7	24888453	24888458	Rps19	+
4	129849581	129849586	Ptp4a2	+
14	48079583	48079588	Gm6055	-
7	120657639	120657644	Uqcrc2	+
11	116023663	116023668	H3f3b	-

19	7427270	7427275	Rtn3	-
2	121457690	121457695	Serf2;Hypk	+
6	99877813	99877818	Tpt1-ps3	-
4	151048542	151048547	Vamp3;Camt	-
11	52244985	52244990	Skp1a	+
19	47083470	47083475	Usmg5	-
10	127067793	127067798	Tspan31	-
17	25261073	25261078	Tsr3	-
8	27053845	27053850	Plpbp	+
19	29047693	29047698	Ak3	-
10	128044355	128044360	Naca	+
4	109061128	109061133	Nrd1	+
2	156873119	156873124	Rab5if	+
8	83571584	83571589	Ndufb7	+
14	8171295	8171300	Pdhb	-
11	59211999	59212004	Arf1	-
5	21738938	21738943	Pmpcb	+
13	106795316	106795321	Ipo11	-
1	171288865	171288870	Ufc1	-
9	106463855	106463860	Pcbp4	+
13	43394801	43394806	Sirt5	+
8	4267985	4267990	Timm44	-
17	17345472	17345477	Oaz1-ps;AC	-
5	114249756	114249761	Acacb	+
11	21557469	21557474	Mdh1	-
3	135466407	135466412	Ube2d3	+
14	34343843	34343848	Glud1	+
7	46850930	46850935	Ldha	+
2	150831035	150831040	Pygb	+
10	13505840	13505845	Fuca2	+
4	126098397	126098402	Lsm10	+
6	51467001	51467006	Hnrnpa2b1	-
15	102474634	102474639	Pcbp2	+
17	27583227	27583232	Nudt3;RPS1(	-
2	121551248	121551253	Frmd5	-
3	96559168	96559173	Txnip	+
1	24613956	24613961	Gm28437	-
2	103463292	103463297	Cat	-
18	68265817	68265822	Fam210a	-
2	30446621	30446626	Ptpa	+
17	40961040	40961045	Mut	+
11	120347844	120347849	Actg1	-
15	79029216	79029221	H1f0	+
12	8498126	8498131	Rhob	-
18	25112554	25112559	Fhod3	+

15	83547072	83547077	Mcat	-
3	94886770	94886775	Psmb4	-
2	76705956	76705961	Ttn	-
6	71214397	71214402	Smyd1	-
12	32850683	32850688	Nampt	+
7	126489205	126489210	Tufm	+
3	87915871	87915876	Hdgf	+
6	87999640	87999645	Rab7	-
4	133266635	133266640	Tmem222	-
8	46535185	46535190	Acsl1	+
2	143915640	143915645	Dstn	+
6	97189536	97189541	Uba3	-
19	4006971	4006976	Doc2g	+
3	7444362	7444367	Pkia	+
9	79771054	79771059	Tmem30a	-
5	115110331	115110336	Acads	-
5	135011609	135011614	Abhd11	+
8	13172607	13172612	Lamp1	+
5	122456929	122456934	Atp2a2	-
14	51911480	51911485	Ndrg2	-
4	44988993	44988998	Grhpr	+
11	55394998	55395003	Sparc	-
4	41769097	41769102	Il11ra1	+
5	36795893	36795898	Mrfap1	-
14	54953863	54953868	Myh6	-
6	24518742	24518747	Ndufa5	-
1	175614785	175614790	Fh1	-
3	88058376	88058381	Naxe	-
16	90226201	90226206	Sod1	+
9	71504550	71504555	Myzap	-
9	88454103	88454108	Gm20537;Sy	-
17	34028632	34028637	Gm20427;Sl	-
6	142589513	142589518	Abcc9	-
11	116172966	116172971	Acox1	-
7	28257361	28257366	Dyrk1b	-
13	21501129	21501134	Gm11273	-
2	50291456	50291461	Mmadhc	-
6	124914018	124914023	Ptms	-
11	100715676	100715681	Rab5c	-
13	93041437	93041442	Cmya5	-
4	130127792	130127797	Pef1	+
6	97184843	97184848	Uba3	-
8	13171277	13171282	Lamp1	+
11	50230859	50230864	Mgat4b	+
15	74994889	74994894	Ly6a	-

19	24876757	24876762	Gm10053	+
11	52383998	52384003	Vdac1	+
19	9984559	9984564	Fth1	+
8	85736124	85736129	Itfg1	-
6	71510711	71510716	Rnf103	+
11	52389370	52389375	Vdac1	+
2	167609015	167609020	Ube2v1;Gm2	-
17	23675688	23675693	Tnfrsf12a	-
14	120429163	120429168	Mbnl2	+
4	136268048	136268053	Tcea3	+
10	62621415	62621420	Ddx50	-
18	34334300	34334305	Srp19	+
18	58679508	58679513	Isoc1	+
8	22569409	22569414	Slc20a2	+
18	36766583	36766588	Hars	-
9	21594357	21594362	Timm29	+
5	24581676	24581681	Gm10221	-
8	13242520	13242525	Adprhl1	-
1	135848023	135848028	Tnnt2	+
17	35956983	35956988	Abcf1	-
2	132249470	132249475	Pcna	-
8	33782841	33782846	Rbpms	-
4	135871619	135871624	Pnrc2	-
2	181670349	181670354	Sox18	-
12	84774659	84774664	Isca2	+
4	59618240	59618245	Hsdl2	+
4	134329294	134329299	Trim63	+
17	48419342	48419347	Apobec2	-
2	71282492	71282497	Slc25a12	-
13	114288733	114288738	Ndufs4	-
1	169695090	169695095	Rgs5	+
2	167689998	167690003	Cebpb	+
8	72481893	72481898	Slc35e1	-
2	30403601	30403606	Crat	-
18	23892214	23892219	Mapre2	+
18	32529013	32529018	Gypc	-
14	37121050	37121055	Ghitm	-
2	153009493	153009498	Pdrg1	-
11	78522534	78522539	Poldip2	+
12	52568430	52568435	Arhgap5	+
19	6906723	6906728	Prdx5	-
7	105557945	105557950	Smpd1	+
8	70895706	70895711	Rpl18a	-
12	110976535	110976540	Ankrd9	-
9	108684377	108684382	Slc25a20	+

8	122613989	122613994	Trappc2l;Gm	+
15	99409582	99409587	Tmbim6	+
17	44039103	44039108	Rcan2	+
6	91487769	91487774	Tmem43	+
11	69942970	69942975	Slc2a4	-
10	86732047	86732052	Fabp3-ps1	-
4	138315693	138315698	Pink1	-
11	78522397	78522402	Poldip2	+
2	84673058	84673063	Gm28635;Tr	-
11	118164495	118164500	Cyth1	-
10	117815039	117815044	Rap1b	-
15	58784632	58784637	Tmem65	-
17	34112952	34112957	Brd2	-
11	98380760	98380765	Stard3	+
2	39015328	39015333	Arpc5l	+
9	53591404	53591409	Acat1	-
8	94394634	94394639	Herpud1	+
19	43735360	43735365	Cox15	-
1	60080527	60080532	Wdr12	-
11	55500410	55500415	G3bp1	+
3	107926490	107926495	Gstm7	-
3	88929471	88929476	Dap3	-
19	40623176	40623181	Entpd1	+
8	22463127	22463132	Smim19	-
11	20823943	20823948	Lgalsl	-
1	131848792	131848797	Slc41a1	+
11	50171788	50171793	Tbc1d9b	+
5	30132279	30132284	Hadha	-
7	13033926	13033931	Chmp2a	-
4	63548588	63548593	Atp6v1g1	+
3	97702286	97702291	Pde4dip	-
9	50596687	50596692	Sdhd	-
6	83806292	83806297	Paip2b	-
11	59228202	59228207	Arf1	-
3	153922547	153922552	Acadm	-
16	21934794	21934799	Tmem41a	-
3	96528098	96528103	Hfe2	+
7	80099036	80099041	Idh2	-
15	25973051	25973056	Retreg1	+
3	86141267	86141272	Rps3a1	-
3	142352776	142352781	Pdlim5	-
10	88472877	88472882	Chpt1	-
17	10207954	10207959	Qk	-
15	31593524	31593529	Cct5	-
10	86732163	86732168	Fabp3-ps1	-

19	6982558	6982563	Vegfb	-
5	21800552	21800557	Psmc2	+
17	12974816	12974821	Wtap	-
11	50295632	50295637	Canx	-
18	34938346	34938351	Hspa9	-
9	94520162	94520167	1190002N15	-
7	139525775	139525780	Inpp5a	+
10	117277989	117277994	Lyz2	-
6	125307044	125307049	Ltbr	-
1	66854674	66854679	Acadl	-
2	174465121	174465126	Prelid3b	-
11	116848965	116848970	Mettl23	+
17	26786702	26786707	Bnip1	+
15	41860498	41860503	Oxr1	+
4	123936382	123936387	Rragc	+
13	118380559	118380564	Mrps30	-
8	70699827	70699832	Jund	+
12	65062466	65062471	Fkbp3	-
14	31000098	31000103	Spcs1	-
11	30649324	30649329	Acyp2	-
13	55725738	55725743	Txndc15	+
7	141459404	141459409	Pnpla2	+
10	95547048	95547053	Nudt4	-
14	73560473	73560478	Sucla2	+
5	103989849	103989854	Hsd17b11	-
14	20320456	20320461	Ecd	-
8	68906540	68906545	Lpl	+
11	75599737	75599742	Pitpna	-
5	120627434	120627439	Ddx54	+
5	73639421	73639426	Sgcb	-
4	152271651	152271656	Acot7	+
10	71330565	71330570	Cisd1	-
5	24581840	24581845	Gm10221	-
18	37937093	37937098	Hdac3	-
19	27253658	27253663	Vldlr	+
4	126706200	126706205	Psmb2	+
4	123298079	123298084	Pabpc4	+
5	30623179	30623184	Kcnk3	+
15	31589938	31589943	Cmbl	+
4	34566417	34566422	Akirin2	+
11	95678057	95678062	Phb	+
10	128361286	128361291	Cs	+
7	27469380	27469385	Blvrb	-
11	100957156	100957161	Cavin1	-
2	74869344	74869349	Mtx2	+

13	24812966	24812971	BC005537	+
5	138259329	138259334	Lamtor4	+
7	30187518	30187523	Capns1	-
2	76704435	76704440	Ttn	-
8	121597693	121597698	Map1lc3b;Gr	+
14	26915193	26915198	Asb14	+
4	136551001	136551006	Kdm1a	-
1	171129411	171129416	Sdhc	-
10	53345189	53345194	Pln	+
9	54603781	54603786	Idh3a	+
5	140441069	140441074	Eif3b	+
2	24974340	24974345	Mrpl41	-
11	6468080	6468085	Purb	-
3	135439721	135439726	Ube2d3	+
19	36117859	36117864	Ankrd1	-
8	71368859	71368864	Use1	+
15	58176276	58176281	Fbxo32	-
14	37121169	37121174	Ghitm	-
11	100715967	100715972	Rab5c	-
10	40289228	40289233	Amd1	-
9	71579637	71579642	Myzap	-
6	29461692	29461697	Flnc	+
14	75847310	75847315	Tpt1	+
11	55499551	55499556	G3bp1	+
19	6988007	6988012	Dnajc4	-
18	35251183	35251188	Ctnna1	+
19	43663860	43663865	Slc25a28	-
9	49041728	49041733	Usp28	+
19	5602448	5602453	Rnaseh2c	+
11	102436190	102436195	Grn	+
14	34344578	34344583	Glud1	+
1	37995730	37995735	Txndc9	-
1	151355177	151355182	Ivns1abp	+
3	103057066	103057071	Csde1	+
19	42123610	42123615	Avpi1	-
7	28788381	28788386	Sirt2	+
16	35697700	35697705	Dirc2	-
9	51948607	51948612	Fdx1	-
9	21589121	21589126	Carm1	+
2	76704123	76704128	Ttn	-
2	84433169	84433174	Tfpi	-
11	82942874	82942879	Unc45b	+
8	3680150	3680155	Trappc5	+
17	46018619	46018624	Vegfa	-
13	99415073	99415078	Mrps27	+

18	75009336	75009341	BC031181	+
3	36091405	36091410	Gm43079;Ac	+
17	56412597	56412602	Ptprs	-
15	98136022	98136027	Asb8	-
6	24603562	24603567	Lmod2	+
11	84821056	84821061	Dhrs11	-
10	18011320	18011325	Abracl	-
1	24615412	24615417	Gm28661	-
4	108861701	108861706	Txndc12	+
1	75309381	75309386	Dnpep	-
11	102436483	102436488	Grn	+
4	58801737	58801742	Ecpas	-
9	65690117	65690122	Oaz2	+
11	120646924	120646929	Myadml2	-
10	57516428	57516433	Serinc1	-
17	34969409	34969414	Hspa1a	-
9	123592278	123592283	Sacm1l	+
14	51910097	51910102	Ndrg2	-
5	23506582	23506587	Srpk2	-
16	38374219	38374224	Popdc2	+
10	79977218	79977223	Tmem259	-
2	121437635	121437640	Pdia3	+
1	66854654	66854659	Acadl	-
17	84993842	84993847	Ppm1b	+
11	5525212	5525217	Xbp1	+
5	97885766	97885771	Antxr2	-
2	64912792	64912797	Grb14	-
15	99725754	99725759	Cox14	+
19	9829803	9829808	AC132253.9	-
4	41757233	41757238	Galt;Il11ra1	+
9	50635206	50635211	Dlat	-
4	130127901	130127906	Pef1	+
4	149896322	149896327	Spsb1	-
19	7588057	7588062	Pla2g16	+
7	45337306	45337311	Hrc	+
11	46441173	46441178	Med7	+
9	98588198	98588203	Copb2	+
2	26911425	26911430	Rpl7a	+
9	59563284	59563289	Hexa	+
19	46740958	46740963	As3mt	+
9	108337224	108337229	Rhoa	+
2	91119406	91119411	Mybpc3	+
1	156366277	156366282	Gm2000	+
6	124959026	124959031	Cops7a	-
9	54595260	54595265	Idh3a	+

11	70010725	70010730	Acadvl	-
2	131178065	131178070	Cenpb;Spef1	-
14	67016263	67016268	Ppp2r2a	-
6	81961874	81961879	Mrpl19	-
17	25831118	25831123	Stub1	-
16	96228774	96228779	Sh3bgr	+
10	128919948	128919953	Rdh5;Bloc1s	-
11	22899864	22899869	Gm28048;Cc	-
9	50597238	50597243	Sdhd	-
15	31457235	31457240	March6	-
10	81487440	81487445	Ncln	-
7	35548099	35548104	Nudt19	-
4	123716063	123716068	Ndufs5	-
15	77015620	77015625	Mb	-
7	125467684	125467689	Nsmce1	-
10	79688282	79688287	Cdc34	+
3	95989575	95989580	Plekho1	-
2	148871795	148871800	Cst3	-
17	33996466	33996471	H2-K1	-
8	72320838	72320843	Klf2	+
11	54910807	54910812	Tnip1	-
16	38362993	38362998	Popdc2	+
5	65571577	65571582	Ube2k	+
12	56373515	56373520	Ralgapa1	-
7	138894511	138894516	Bnip3	-
4	139291190	139291195	Capzb	+
7	45585520	45585525	Bcat2	+
2	153009429	153009434	Pdrg1	-
5	117287576	117287581	Pebp1	-
11	55499908	55499913	G3bp1	+
7	114705467	114705472	Gm15500	-
5	148315042	148315047	Mtus2	+
9	59678205	59678210	Pkm	+
7	142383462	142383467	Ctsd	-
5	30119462	30119467	Hadha	-
6	72369261	72369266	Vamp5	-
12	110692195	110692200	Hsp90aa1	-
3	116549960	116549965	Dbt	+
7	105557648	105557653	Smpd1	+
1	4845705	4845710	Lypla1;Gm37	+
13	100736253	100736258	Mrps36	-
6	87999671	87999676	Rab7	-
7	19701376	19701381	Tomm40	-
19	23979439	23979444	Fam189a2	-
11	75672735	75672740	Myo1c	+

14	54950087	54950092	Myh6	-
8	71374397	71374402	Nr2f6	-
7	126796863	126796868	Aldoa	-
14	61221262	61221267	Sgcg	-
11	69990124	69990129	Ctdnep1	+
19	8880727	8880732	Uqcc3	-
11	60715709	60715714	Flii	-
1	59639498	59639503	Sumo1	-
15	80256402	80256407	Atf4	+
2	114062021	114062026	C130080G1(	+
11	120105298	120105303	Slc38a10	-
17	26508407	26508412	Dusp1	-
19	44553679	44553684	Ndufb8	-
10	24597964	24597969	Ccn2	+
2	76708237	76708242	Ttn	-
11	21557499	21557504	Mdh1	-
15	37000561	37000566	Zfp706	-
19	57033371	57033376	Ablim1	-
11	50295106	50295111	Canx	-
4	154897092	154897097	Fam213b	-
15	31456461	31456466	March6	-
5	122459512	122459517	Atp2a2	-
11	84823078	84823083	Dhrs11	-
16	17045001	17045006	Mapk1	+
11	33156071	33156076	Npm1	-
19	9829127	9829132	AC132253.9	-
2	74869415	74869420	Mtx2	+
17	27579635	27579640	Nudt3	-
15	3333469	3333474	Ghr	-
2	180257992	180257997	Rps21	+
15	55534140	55534145	Mrpl13	-
10	77644733	77644738	Ube2g2	+
2	173034480	173034485	Rbm38	+
11	96820179	96820184	Nfe2l1	-
8	105530973	105530978	Atp6v0d1	-
1	131055061	131055066	Mapkapk2	-
3	152237855	152237860	Nexn	-
4	24903458	24903463	Ndufaf4	+
12	84774998	84775003	Isca2	+
15	76352749	76352754	Maf1	+
17	46018128	46018133	Vegfa	-
17	35098960	35098965	Abhd16a	+
5	72300254	72300259	Corin	-
17	24897080	24897085	Mrps34;Nme	+
4	139648891	139648896	Gm21969;Al(	+

13	64364150	64364155	Ctsl	-
8	119410521	119410526	Mlycd	+
1	75216214	75216219	Tuba4a	-
10	117818533	117818538	Rap1b	-
8	61512013	61512018	Palld	-
4	144927178	144927183	Dhrs3	+
5	118061728	118061733	Tesc	+
6	117924024	117924029	Hnrnpf	+
5	30144162	30144167	Hadha	-
4	106662287	106662292	Ttc4	-
7	142376007	142376012	Gm49369;Ct	-
7	28256715	28256720	Dyrk1b	-
15	74995270	74995275	Ly6a	-
10	59396537	59396542	Gm10273	+
1	37419868	37419873	Coa5	-
4	156220364	156220369	Perm1	+
3	27244621	27244626	Nceh1	+
2	130173756	130173761	Snrpb	-
16	4062397	4062402	Trap1	-
14	99035675	99035680	Mzt1	-
4	138206540	138206545	Eif4g3	+
10	85016390	85016395	Ric8b	+
4	149449717	149449722	Rbp7	-
17	24849333	24849338	Fahd1	-
16	91575413	91575418	Tmem50b	-
12	110873244	110873249	Cinp	-
9	121770197	121770202	Zfp651	+
4	135215555	135215560	Clic4	-
7	79449783	79449788	Polg	-
4	151060781	151060786	Camta1	-
11	106781616	106781621	Ddx5	-
5	30658284	30658289	Slc35f6	+
15	81848594	81848599	Tob2	-
4	73941821	73941826	2310002L09I	-
2	114062138	114062143	C130080G1(	+
10	33186654	33186659	Trdn	+
15	31589860	31589865	Cmbl	+
1	40855611	40855616	Tmem182	+
1	42898473	42898478	Mrps9	+
2	151718322	151718327	Psmf1	-
11	58932457	58932462	Rnf187	-
10	43532880	43532885	1700021F05I	-
15	89371919	89371924	Sco2	-
14	26656296	26656301	Arf4	+
2	103470402	103470407	Cat	-

1	54990543	54990548	Sf3b1	-
12	31340910	31340915	Dld	-
6	138156579	138156584	Mgst1	+
9	121453401	121453406	Trak1	+
14	8170373	8170378	Pdhb	-
11	120345946	120345951	Actg1	-
9	123791068	123791073	AC165425.1;	-
8	4259758	4259763	Timm44	-
8	4260044	4260049	Timm44	-
11	120579185	120579190	Arhgdia	-
13	91861835	91861840	Ckmt2	-
14	8166820	8166825	Pdhb	-
4	135214093	135214098	Clic4	-
10	79994849	79994854	Cnn2	+
6	56878381	56878386	Fkbp9	+
7	128205643	128205648	Cox6a2	-
17	12227980	12227985	Map3k4	-
15	25971962	25971967	Retreg1	+
5	137747592	137747597	Tsc22d4	+
2	29738115	29738120	Rapgef1	+
2	32619586	32619591	St6galnac6	+
13	38500499	38500504	Txndc5	-
1	75558887	75558892	Slc4a3	+
1	55078186	55078191	Hspd1	-
4	107197522	107197527	Tmem59	+
12	87164069	87164074	Gstz1	+
7	46850175	46850180	Ldha	+
11	102403692	102403697	Slc25a39	-
18	77777576	77777581	Atp5a1	+
5	31141379	31141384	Mpv17	-
8	126937127	126937132	Tomm20	-
11	95675631	95675636	Phb	+
16	20662096	20662101	Psmd2	+
5	145104498	145104503	Arpc1a	+
6	86525755	86525760	Pcbp1	-
15	78894099	78894104	Gga1	+
4	116639017	116639022	Akr1a1	-
7	16115631	16115636	Napa	+
3	150072873	150072878	Rpsa-ps10	-
3	27244049	27244054	Nceh1	+
6	32792675	32792680	Chchd3	-
7	16116790	16116795	Napa	+
1	75245054	75245059	Dnajb2	+
18	80322588	80322593	Kcng2	-
9	21008750	21008755	Mrpl4	+

4	155808696	155808701	Mrpl20	+
4	73942151	73942156	2310002L09I	-
2	122263203	122263208	Sord	+
1	93407814	93407819	Hdlbp	-
17	56613935	56613940	Rpl36	+
11	88830837	88830842	Akap1	-
5	110784748	110784753	Ulk1	-
2	158117522	158117527	Tgm2	-
8	93972437	93972442	Amfr	-
13	6579421	6579426	Pitrm1	+
2	84671641	84671646	Gm28635;Tr	-
15	79671922	79671927	Tomm22	+
1	170705316	170705321	Atf6	-
15	41861002	41861007	Oxr1	+
5	95862474	95862479	Gm5559	+
5	115110787	115110792	Acads	-
6	17665783	17665788	Capza2	+
8	70677958	70677963	Lsm4	+
9	44344089	44344094	Hmbs	-
4	147987154	147987159	Nppb	+
19	60865311	60865316	Prdx3	-
14	65980981	65980986	Clu	+
11	59012372	59012377	Obscn	-
15	73757027	73757032	Ptp4a3	+
11	84821699	84821704	Dhrs11	-
4	24903212	24903217	Ndufaf4	+
3	90499959	90499964	Chtop	-
2	150831575	150831580	Pygb	+
6	126844796	126844801	Ndufa9	-
9	83409829	83409834	Rps27a-ps2	+
7	101823383	101823388	Inppl1	-
4	139291571	139291576	Capzb	+
10	82620161	82620166	1190007I07F	-
11	100361825	100361830	Gm10039	-
6	67035636	67035641	Gadd45a	-
2	75640839	75640844	Rps6-ps4	+
8	124908731	124908736	Egln1	-
2	25505405	25505410	Fbxw5	+
2	75191447	75191452	Rps6-ps4	-
7	89140363	89140368	Tmem135	-
11	62894120	62894125	Tvp23b	+
3	68617639	68617644	Iqschfp;Schi	+
11	62317998	62318003	Ncor1	-
7	80100865	80100870	Idh2	-
14	54468622	54468627	Lrp10	+

7	140099606	140099611	Fuom	-
2	17344191	17344196	Nebl	-
2	167610342	167610347	Ube2v1;Gm2	-
11	106782017	106782022	Ddx5	-
15	76643954	76643959	Cyhr1	-
3	95659380	95659385	Mcl1	+
7	100485859	100485864	Ucp3	+
7	19696648	19696653	Apoe	-
9	108206872	108206877	Dag1	-
17	20965778	20965783	Ppp2r1a	+
17	56721996	56722001	Ndufa11	+
11	98384548	98384553	Tcap	+
17	24849516	24849521	Fahd1	-
11	120347078	120347083	Actg1	-
13	118383041	118383046	Mrps30	-
14	54944459	54944464	Myh6	-
2	75642486	75642491	Rps6-ps4	+
19	34246224	34246229	Acta2	-
14	37125952	37125957	Ghitm	-
14	59586596	59586601	Cdadc1	-
2	4937701	4937706	Phyh	+
15	77915805	77915810	Txn2	-
4	33245982	33245987	Pnrc1	-
4	141576237	141576242	Fblim1	-
12	111670238	111670243	Ckb	-
9	37537849	37537854	Esam	+
11	97328693	97328698	Mrpl45	+
3	146729603	146729608	Prkacb	-
7	78780585	78780590	Mrpl46	-
9	21067905	21067910	Gm49373;Gr	-
6	88000302	88000307	Rab7	-
17	48431519	48431524	Apobec2	-
19	6054795	6054800	Mrpl49	-
4	147874425	147874430	Mfn2	-
17	34970208	34970213	Hspa1a	-
2	76704740	76704745	Ttn	-
9	53581729	53581734	Acat1	-
6	72850409	72850414	Kcmf1	-
7	90450829	90450834	Tmem126a	-
4	137569797	137569802	Hspg2	+
1	189880660	189880665	Smyd2	-
17	36255768	36255773	Rpp21	-
7	19701370	19701375	Tomm40	-
5	114250210	114250215	Acacb	+
9	50598873	50598878	Sdhd	-

11	20335775	20335780	Gm12033	-
1	64994483	64994488	Rpl10a-ps1	-
14	120947051	120947056	Ipo5	+
6	115618972	115618977	Raf1	-
15	27593513	27593518	Ank	+
5	135734919	135734924	Por	+
10	79682520	79682525	Cdc34	+
8	19493279	19493284	Rpl19-ps11	+
6	99878315	99878320	Tpt1-ps3	-
3	36091028	36091033	Gm43079;Ac	+
7	143514057	143514062	Nap1l4	-
9	14501950	14501955	Cwc15	+
9	21508192	21508197	Tmed1	-
19	24876295	24876300	Gm10053	+
3	142302498	142302503	Pdlim5	-
11	65174261	65174266	Stau2	-
8	71375847	71375852	Nr2f6	-
1	175619101	175619106	Fh1	-
11	4481554	4481559	Mtmr3	-
13	49203123	49203128	Card19	-
5	140758563	140758568	Gna12	-
2	19394406	19394411	Msrb2	+
9	107565523	107565528	Tusc2	+
1	172122267	172122272	Copa	+
3	35959946	35959951	Mccc1	-
11	75765551	75765556	Ywhae	+
8	121549608	121549613	Fbxo31	-
2	151560706	151560711	Fkbp1a	+
8	46534823	46534828	Acsl1	+
3	95676350	95676355	Adamtsl4	-
3	104794224	104794229	Rhoc	+
2	103454534	103454539	Cat	-
5	115111338	115111343	Acads	-
4	134329403	134329408	Trim63	+
6	87843667	87843672	Cnbp	-
19	36112695	36112700	Ankrd1	-
7	99479979	99479984	Rps3	-
10	117215618	117215623	Yeats4	-
10	71331064	71331069	Cisd1	-
4	147987173	147987178	Nppb	+
15	73751330	73751335	Ptp4a3	+
11	51602607	51602612	Hnrnpab	-
11	75513089	75513094	Rilp	+
11	96818707	96818712	Nfe2l1	-
16	33948812	33948817	Itgb5	+

8	70182606	70182611	Tmem161a	+
14	61221621	61221626	Sgcg	-
2	103454640	103454645	Cat	-
6	24605210	24605215	Lmod2	+
4	109453805	109453810	Rnf11	-
9	55486726	55486731	Etfa	-
3	53692679	53692684	Gm6204	-
13	97135322	97135327	Nsa2	-
16	33770948	33770953	Heg1	+
14	34577227	34577232	Ldb3	-
1	131055051	131055056	Mapkapk2	-
15	79672606	79672611	Tomm22	+
2	153008936	153008941	Pdrg1	-
7	93179452	93179457	Gm15501	-
11	40749017	40749022	Ccng1	-
1	170925521	170925526	Gm2962;Fcr	-
17	25831127	25831132	Stub1	-
9	107545571	107545576	Nprl2	+
2	164830896	164830901	Neurl2	-
1	155099065	155099070	Ier5	-
7	73776633	73776638	Fam174b	+
4	148146037	148146042	Fbxo6	-
10	128548200	128548205	Rpl41	-
10	40287960	40287965	Amd1	-
4	49586006	49586011	Tmem246	-
6	86334032	86334037	Gm10443	+
12	31926977	31926982	Hbp1	-
2	71293671	71293676	Slc25a12	-
8	107585731	107585736	Psmd7	-
10	60300229	60300234	Psap	+
15	99409756	99409761	Tmbim6	+
15	99402059	99402064	Tmbim6	+
4	115811690	115811695	Atpaf1	+
19	32673528	32673533	Atad1	-
13	73319956	73319961	Ndufs6	-
14	120946946	120946951	Ipo5	+
3	57294664	57294669	Tm4sf1	-
3	123034910	123034915	Myoz2	-
1	55072196	55072201	Coq10b	+
4	14810190	14810195	Otud6b	-
2	90917620	90917625	Ptpmt1	-
16	91679015	91679020	Son	+
16	65539713	65539718	Chmp2b	-
17	83502423	83502428	Cox7a2l	-
11	101577210	101577215	Nbr1	+

3	89272233	89272238	Efna1	-
15	79135966	79135971	Micall1	+
13	33918270	33918275	Serpinb6a	-
2	150819892	150819897	Pygb	+
7	112110233	112110238	Usp47	+
6	86369584	86369589	Fam136a	+
10	57516459	57516464	Serinc1	-
9	67997633	67997638	Tln2	+
6	127961787	127961792	Tspan9	-
17	56281579	56281584	Plin3	-
7	101787704	101787709	Clpb	+
14	61556532	61556537	Spryd7	-
5	115111930	115111935	Acads	-
11	88210914	88210919	Mrps23	+
8	46208473	46208478	Slc25a4	-
5	127955273	127955278	Slc15a4	+
12	3309366	3309371	Rab10	-
19	34243411	34243416	Acta2	-
10	128365035	128365040	Coq10a	-
13	114288780	114288785	Ndufs4	-
17	56998549	56998554	Alkbh7	+
11	21353391	21353396	Ugp2	-
17	28747451	28747456	Mapk14	+
8	33783417	33783422	Rbpms	-
10	7630864	7630869	Pcmt1	-
1	36423483	36423488	Lman2l	-
8	13256871	13256876	Dcun1d2	-
8	86633959	86633964	Lonp2	+
1	155558569	155558574	Acbd6	+
5	30140974	30140979	Hadha	-
7	54412327	54412332	Gm5776	-
11	49678815	49678820	Gm12191;Cr	-
4	42980409	42980414	Vcp	-
14	63199674	63199679	Gata4	-
8	70184778	70184783	Slc25a42	-
5	88670307	88670312	Grsf1	-
5	30178699	30178704	Hadhb	+
9	108388303	108388308	Usp4	+
6	5483755	5483760	Pdk4	-
15	82041545	82041550	Snu13	-
4	133292564	133292569	Wdtc1	-
18	60776946	60776951	Rps14	+
4	156220879	156220884	Perm1	+
7	19565028	19565033	Gemin7	-
2	28934116	28934121	Gm13394;Cf	-

9	54596152	54596157	Idh3a	+
6	120888676	120888681	Bcl2l13	+
8	72815433	72815438	Large1	-
12	110858411	110858416	Wdr20	-
10	128560140	128560145	Pa2g4	-
1	165649618	165649623	Rcsd1	-
10	88473574	88473579	Chpt1	-
9	21233529	21233534	Keap1	-
6	147070802	147070807	Mrps35	+
7	111077261	111077266	Eif4g2	-
4	141425300	141425305	Hspb7	+
4	130127954	130127959	Pef1	+
5	121208495	121208500	Rpl6	+
5	143564370	143564375	Fam220a;Fa	+
2	84444883	84444888	Tfpi	-
10	94220755	94220760	Ndufa12	+
1	92920221	92920226	Rnpepl1	+
11	52387466	52387471	Vdac1	+
12	85134026	85134031	Dlst	+
2	127295502	127295507	Stard7	+
11	70661433	70661438	Eno3	+
2	76707131	76707136	Ttn	-
4	155740946	155740951	Atad3a	-
7	102107252	102107257	Art1	+
4	123936894	123936899	Rragc	+
9	31157811	31157816	Aplp2	-
8	122813586	122813591	Acsf3;Gm20	+
18	53406698	53406703	Ppic	-
1	128277248	128277253	Ubxn4	+
7	80099062	80099067	Idh2	-
9	65690191	65690196	Oaz2	+
8	56591637	56591642	Fbxo8	+
2	143942545	143942550	Dstn	+
8	106573002	106573007	Gm10073	-
14	69704032	69704037	R3hcc1	-
2	76705065	76705070	Ttn	-
7	103827552	103827557	Hbb-bs	-
11	70525882	70525887	Psmb6	+
5	67355011	67355016	Slc30a9	+
7	103813443	103813448	Hbb-bt	-
18	32529227	32529232	Gypc	-
11	78290172	78290177	2610507B11	+
16	20124690	20124695	Klhl24	+
12	8498942	8498947	Rhob	-
12	21324367	21324372	Adam17	-

7	114047255	114047260	Rras2	-
8	72319440	72319445	Klf2	+
11	101278217	101278222	Coa3	-
8	107418073	107418078	Nob1	-
6	17340004	17340009	Cav1	+
7	3297106	3297111	Myadm;Prkc	+
5	121572707	121572712	Aldh2	-
2	120507293	120507298	Zfp106	-
5	125387459	125387464	Ubc	-
6	50565102	50565107	Cycs	-
17	24596585	24596590	Tsc2	-
9	19927868	19927873	Chordc1	-
19	53588636	53588641	Nutf2-ps1	-
6	113737958	113737963	Sec13	-
5	65390429	65390434	Rpl9	-
3	123034229	123034234	Myoz2	-
11	58933066	58933071	Rnf187	-
4	114614676	114614681	Trabd2b	+
18	35617028	35617033	Paip2	+
14	79201525	79201530	Vwa8	+
10	7768453	7768458	Ginm1	-
3	101594248	101594253	Atp1a1	-
7	45143292	45143297	Aldh16a1	-
14	54952258	54952263	Myh6	-
9	101105787	101105792	Ppp2r3a	-
8	116888959	116888964	Cmc2	-
7	81818231	81818236	Btbd1	-
8	104516145	104516150	Nae1	-
8	84977404	84977409	Junb	-
3	101590679	101590684	Atp1a1	-
11	48825809	48825814	Gm12184	-
1	152775455	152775460	Arpc5	+
11	73176022	73176027	Emc6	-
11	55500013	55500018	G3bp1	+
15	81365199	81365204	St13	-
10	128358353	128358358	Cs	+
19	29022150	29022155	Ak3	-
4	101153178	101153183	Jak1	-
19	18704616	18704621	Carnmt1	+
14	74742038	74742043	Esd	+
3	116927582	116927587	Palmd	-
13	14612965	14612970	Mrpl32	-
17	86999625	86999630	Rhoq	+
10	79711906	79711911	Bsg	+
17	35243421	35243426	Ddx39b	+

11	71010230	71010235	Derl2	-
10	13008969	13008974	Sf3b5	+
10	69291709	69291714	Rhobtb1	+
8	111055098	111055103	Aars	+
5	104092628	104092633	Sparcl1	-
2	143942873	143942878	Dstn	+
4	107907577	107907582	Cpt2	-
13	63302529	63302534	2010111I01F	+
5	3641379	3641384	Gatad1	-
9	44333303	44333308	Dpagt1	+
9	4316940	4316945	Kbtbd3	+
10	41529929	41529934	Cd164	+
2	4938713	4938718	Phyh	+
10	81561268	81561273	Aes	+
8	95420528	95420533	Cfap20	-
12	113136866	113136871	Mta1	+
14	79065048	79065053	Vwa8	+
3	30818797	30818802	Sec62	+
2	32109708	32109713	Plpp7	+
9	20645449	20645454	Ubl5	+
17	12919850	12919855	Tcp1	+
14	66310560	66310565	Trim35	+
11	96819906	96819911	Nfe2l1	-
10	57519547	57519552	Serinc1	-
12	69692401	69692406	L2hgdh	-
7	28258191	28258196	Dyrk1b	-
19	32674261	32674266	Atad1	-
10	19609991	19609996	Ifngr1	+
15	76070524	76070529	Puf60	-
17	44279681	44279686	Clic5	+
7	28305752	28305757	Timm50	-
11	4766037	4766042	Nf2	-
15	12170818	12170823	Zfr	+
9	72748408	72748413	Nedd4	+
15	98931926	98931931	Tuba1b	-
3	105955896	105955901	Atp5f1	-
2	75642408	75642413	Rps6-ps4	+
15	82350244	82350249	Ndufa6	-
16	11804916	11804921	Cpped1	-
11	120560515	120560520	P4hb	-
13	98724042	98724047	Fcho2	-
19	3909044	3909049	Ndufs8	-
7	101787548	101787553	Clpb	+
17	25840073	25840078	Rhot2;Gm20	-
7	73776474	73776479	Fam174b	+

18	44355525	44355530	Myot	+
1	53347659	53347664	Gm28551;As	-
3	97694066	97694071	Pde4dip	-
2	127297853	127297858	Stard7	+
1	80281547	80281552	Cul3	-
1	24613499	24613504	Gm28437	-
17	34026187	34026192	H2-Ke6;Gm2	-
14	66086972	66086977	Ephx2	-
6	86516077	86516082	Gm44386	-
4	43447551	43447556	Tesk1	+
2	25271315	25271320	Ssna1	-
12	81359239	81359244	Gm20498;Cc	-
2	104426561	104426566	Hipk3	-
1	75367152	75367157	Des	+
14	55898344	55898349	Sdr39u1	-
9	108684399	108684404	Slc25a20	+
18	74798461	74798466	Acaa2	+
12	85133067	85133072	Dlst	+
7	81609575	81609580	Homer2	-
5	124570992	124570997	Eif2b1	-
9	22448432	22448437	Rp9	-
7	105556944	105556949	Smpd1	+
5	17798257	17798262	Cd36	-
8	84970466	84970471	Prdx2	+
17	47601482	47601487	Bysl	-
3	152185047	152185052	Dnajb4	-
2	127298072	127298077	Stard7	+
11	5801770	5801775	Pgam2	-
17	48416857	48416862	Oard1	+
10	80340239	80340244	Adamtsl5	-
6	32892350	32892355	Chchd3	-
5	100799593	100799598	Mrps18c;Gm	+
4	119294467	119294472	Ybx1	-
18	61589549	61589554	Csnk1a1	+
7	47053919	47053924	Tmem86a	+
7	45455820	45455825	Gys1	+
11	23648860	23648865	Pex13	-
2	180162273	180162278	Osbpl2	+
4	45399347	45399352	Slc25a51	-
19	45026253	45026258	Lzts2	+
15	81914280	81914285	Aco2	+
17	53671701	53671706	Kat2b	+
14	67740578	67740583	Kctd9	+
16	20128249	20128254	Klhl24	+
4	133965136	133965141	Hmgn2	-

11	87980343	87980348	Dynll2	-
18	20031412	20031417	Dsc2	-
11	70980013	70980018	C1qbp	-
6	17340577	17340582	Cav1	+
1	182279208	182279213	Degs1	-
16	91558025	91558030	Ifngr2	+
11	115503111	115503116	Jpt1	-
13	73816460	73816465	Slc12a7	+
6	124715967	124715972	Phb2	+
7	47054166	47054171	Tmem86a	+
10	91118062	91118067	Slc25a3	-
4	43447719	43447724	Tesk1	+
10	59371208	59371213	P4ha1	+
13	64290138	64290143	Aaed1	-
2	174122300	174122305	Npepl1	+
1	125411343	125411348	Actr3	-
10	84614315	84614320	Tcp11l2	+
10	79709731	79709736	Bsg	+
9	120124199	120124204	Slc25a38	+
9	55454636	55454641	Etfa	-
9	22014003	22014008	Prkcsh	+
6	119925989	119925994	Wnk1	-
7	142080004	142080009	Dusp8	-
3	122266831	122266836	Gclm	+
16	91925550	91925555	Atp5o;Atp5o	-
19	9116615	9116620	Asrgl1	-
15	77915273	77915278	Txn2	-
2	101628938	101628943	B230118H07	-
7	102107045	102107050	Art1	+
17	86998953	86998958	Rhoq	+
8	3153945	3153950	Insr	-
14	31211583	31211588	Tnnc1	+
10	95175669	95175674	Cradd	-
2	30822443	30822448	Ntmt1	+
11	21321518	21321523	Ugp2	-
1	24614665	24614670	Gm10925	-
2	153009231	153009236	Pdrg1	-
2	122809445	122809450	Sqor	+
11	94491042	94491047	Epn3	-
19	25434031	25434036	Kank1	+
3	32934966	32934971	Usp13	+
5	97885088	97885093	Antxr2	-
18	16624234	16624239	Cdh2	-
4	43426075	43426080	Rusc2	+
1	172273040	172273045	Atp1a2	-

1	72712193	72712198	Rpl37a	+
11	97161607	97161612	Kpnb1	-
11	100321635	100321640	Eif1	+
9	78479665	78479670	Eef1a1	-
15	81912246	81912251	Aco2	+
13	41017158	41017163	Tmem14c	+
2	108949076	108949081	Gm13910	+
15	98132057	98132062	Pfkm	+
19	10900196	10900201	Prpf19	+
7	46855446	46855451	Ldha	+
2	131937604	131937609	Prnp;Prn	+
11	120105427	120105432	Slc38a10	-
12	40176491	40176496	Lsmem1	-
7	140877667	140877672	Sirt3	-
11	115523484	115523489	Sumo2	-
1	151363617	151363622	Ivns1abp	+
19	8770841	8770846	Nxf1	+
16	58493819	58493824	St3gal6	-
13	41021127	41021132	Tmem14c	+
11	59836995	59837000	Cops3	-
12	84314838	84314843	Ptgr2	+
15	83153665	83153670	Cyb5r3	-
17	56187441	56187446	Dpp9	-
9	22185726	22185731	Rpl15-ps3	-
2	119599698	119599703	1700020I14F	+
8	82127444	82127449	Inpp4b	+
2	94412312	94412317	Api5	-
14	45459868	45459873	Fermt2	-
4	121059333	121059338	Zmpste24	-
12	84377231	84377236	Entpd5	-
2	91134496	91134501	Mybpc3	+
1	30941594	30941599	Ptp4a1;Ptp4a	-
17	12915653	12915658	Mrpl18	-
1	167631034	167631039	Rxrg	+
8	119508747	119508752	Mbtps1	-
7	140150355	140150360	Mtg1	+
4	123936351	123936356	Rragc	+
18	46588363	46588368	Tmed7	-
14	20316543	20316548	Nudt13	+
17	47599407	47599412	Ccnd3	+
2	18675865	18675870	Commd3	+
12	32850836	32850841	Nampt	+
6	72154331	72154336	St3gal5	+
5	45507203	45507208	Lap3	+
6	122456962	122456967	Gm8430	+

3	94448383	94448388	Mrpl9	+
19	5365109	5365114	Banf1	-
2	71275137	71275142	Slc25a12	-
3	54482725	54482730	Gm5641	+
11	116250842	116250847	Srp68	-
10	79709746	79709751	Bsg	+
2	32681952	32681957	Eng	+
4	15917438	15917443	Decr1	-
1	171238315	171238320	Ndufs2	-
7	139659651	139659656	Cfap46	-
11	120104694	120104699	Slc38a10	-
7	108934751	108934756	Eif3f	+
19	3908935	3908940	Ndufs8	-
1	65159156	65159161	Idh1	-
6	34317385	34317390	Akr1b3	-
8	110881394	110881399	Cog4	+
19	24683586	24683591	Pgm5	-
8	56591678	56591683	Fbxo8	+
14	54368504	54368509	Oxa1l	+
9	118582245	118582250	Golga4	+
17	85062434	85062439	Ppm1b	-
2	121458267	121458272	Serf2;Hypk	+
5	30184288	30184293	Hadhb	+
2	34775800	34775805	Hspa5	+
17	46248318	46248323	Yipf3	+
4	126854028	126854033	AU040320	+
1	135846734	135846739	Tnnt2	+
11	30939582	30939587	Erlec1	-
5	92444185	92444190	Scarb2	-
1	134428079	134428084	Adipor1	+
2	121311108	121311113	Ppip5k1	-
11	120347249	120347254	Actg1	-
2	152280869	152280874	Csnk2a1	+
17	56070924	56070929	Ubxn6	-
9	67029668	67029673	Tpm1	-
15	76904614	76904619	Rpl8	+
10	57516586	57516591	Serinc1	-
9	21067672	21067677	Gm49373;Gı	-
10	127067877	127067882	Tspan31	-
5	45434526	45434531	Qdpr	-
5	30623883	30623888	Kcnk3	+
9	110983493	110983498	Lrrc2	+
2	156873258	156873263	Rab5if	+
10	42312837	42312842	Afg1l	-
4	139280501	139280506	Capzb	+

11	115534630	115534635	Sumo2	-
8	13781502	13781507	Cdc16	+
10	18856731	18856736	Perp	+
12	54861699	54861704	Cfl2	-
17	25864645	25864650	Mcrip2	-
9	107300545	107300550	Cish	+
7	140099637	140099642	Fuom	-
18	80295074	80295079	Kcng2	-
10	29345620	29345625	Echdc1	+
1	37898120	37898125	Mrpl30	+
4	46114884	46114889	Tmod1	+
7	140886560	140886565	Psmd13	+
4	141576338	141576343	Fblim1	-
1	51302616	51302621	Cavin2	+
8	11198673	11198678	Col4a1	-
15	98123838	98123843	Pfkm	+
15	58899288	58899293	Rnf139;Gm4	+
10	63016171	63016176	Hnrnph3	-
7	122093652	122093657	Ndufab1	-
12	4247590	4247595	Ncoa1	-
5	115343057	115343062	Triap1	+
11	50296034	50296039	Canx	-
5	129788258	129788263	Cct6a	+
17	24722763	24722768	Ndufb10	-
4	147946819	147946824	2510039O18	+
5	5537915	5537920	Gtpbp10	-
10	117046587	117046592	Lrrc10	+
11	101288949	101288954	Becn1	-
7	74275639	74275644	Slco3a1	-
5	24409840	24409845	Abcb8	+
7	28258230	28258235	Dyrk1b	-
6	142589755	142589760	Abcc9	-
5	140441007	140441012	Eif3b	+
3	95897057	95897062	Aph1a	+
3	138459175	138459180	Metap1	-
13	11556695	11556700	Ryr2	-
7	19565225	19565230	Gemin7	-
8	13378338	13378343	Tfdp1	+
2	24974403	24974408	Mrpl41	-
13	75849430	75849435	Glrx	+
1	165480986	165480991	Mpc2	+
5	33600654	33600659	Fam53a	-
11	75511311	75511316	Rilp	+
8	71381308	71381313	Nr2f6	-
4	141619541	141619546	Slc25a34	-

13	74360190	74360195	Lrrc14b	-
10	77486835	77486840	Fam207a	-
7	141447970	141447975	Rplp2	+
1	120226905	120226910	Steap3	-
15	4153735	4153740	Oxct1	+
6	88828009	88828014	Mgll	+
8	13253949	13253954	Adprhl1	-
4	123715929	123715934	Ndufs5	-
12	110691599	110691604	Hsp90aa1	-
15	25971892	25971897	Retreg1	+
1	190118093	190118098	Prox1	-
9	55221067	55221072	Fbxo22	+
9	119484992	119484997	Scn5a	-
4	108044395	108044400	Podn;Scp2	-
2	174346393	174346398	Gnas	+
18	32529268	32529273	Gypc	-
6	148232770	148232775	Tmtc1	-
15	3457972	3457977	Ghr	-
15	12185148	12185153	Zfr	+
10	13009034	13009039	Sf3b5	+
2	25222639	25222644	Tubb4b	-
1	58405842	58405847	Bzw1	+
19	8772280	8772285	Tmem223	+
7	25711370	25711375	Ccdc97	-
17	27580161	27580166	Nudt3	-
17	35957173	35957178	Abcf1	-
14	31259714	31259719	Bap1	+
7	29247430	29247435	Yif1b	+
1	86350937	86350942	Ncl	-
12	113153824	113153829	Crip1	+
7	46851925	46851930	Ldha	+
3	87922041	87922046	Mrpl24	+
2	67526306	67526311	Xirp2	+
18	38838986	38838991	Fgf1	-
7	126546943	126546948	Eif3c	-
6	17340421	17340426	Cav1	+
6	99625383	99625388	Eif4e3;Gm20	-
7	73776602	73776607	Fam174b	+
3	137918613	137918618	Lamtor3	+
7	141493176	141493181	Tspan4	+
15	83484150	83484155	Ttll1	-
4	62509231	62509236	Alad	-
5	121572084	121572089	Aldh2	-
9	122176061	122176066	Ano10	-
5	112692341	112692346	Myo18b	-

9	100495455	100495460	Nck1	-
11	119898688	119898693	Rptor	+
9	65330090	65330095	Clpx	+
12	111545948	111545953	Eif5	+
7	126294297	126294302	Sbk1	+
13	75847233	75847238	Glrx	+
16	37647702	37647707	Ndufb4	-
11	32283723	32283728	Hba-a1	+
4	63550510	63550515	Atp6v1g1	+
1	118299093	118299098	Tsn	-
2	78872444	78872449	Ube2e3	+
9	64174964	64174969	Rpl4	+
17	81386630	81386635	Slc8a1	-
6	83055229	83055234	Aup1	+
10	41487261	41487266	Smpd2	-
11	5872283	5872288	Pold2	-
14	45459366	45459371	Fermt2	-
14	105682003	105682008	Gm10076	+
13	86045771	86045776	Cox7c	-
7	100872461	100872466	Arhgef17	-
7	130979389	130979394	Htra1	+
1	80267219	80267224	Cul3	-
11	78181585	78181590	Rpl23a	-
9	114796046	114796051	Cmtm8	-
3	146838903	146838908	Gm10288	-
14	37133193	37133198	Ghitm	-
9	50636136	50636141	Dlat	-
17	86832329	86832334	Epas1	+
3	123026109	123026114	Myoz2	-
7	25711442	25711447	Ccdc97	-
3	105954107	105954112	Atp5f1	-
11	59212570	59212575	Arf1	-
2	28470677	28470682	Mrps2	+
11	20335005	20335010	Steap3	-
19	32466335	32466340	Rpl9-ps6	-
18	31796479	31796484	Polr2d	+
3	133310261	133310266	Ppa2	+
9	120959899	120959904	Ctnnb1	+
6	147070578	147070583	Mrps35	+
14	31000011	31000016	Spcs1	-
4	86656726	86656731	Plin2	-
11	68209600	68209605	Ntn1	-
12	85299763	85299768	Nek9	-
4	132314266	132314271	Trnau1ap	-
17	44038727	44038732	Rcan2	+

8	13219487	13219492	Lamp1	-
1	16570504	16570509	Ube2w	-
13	48880640	48880645	Fam120a	-
6	71868729	71868734	Immt	+
6	32792408	32792413	Chchd3	-
4	150248681	150248686	Eno1	+
6	108823204	108823209	Arl8b	+
1	74287309	74287314	Pnkd	+
13	73331646	73331651	Mrpl36	+
1	85851158	85851163	Cab39	+
5	114249866	114249871	Acacb	+
12	44205954	44205959	Dnajb9	-
4	46083594	46083599	Tmod1	+
4	140966590	140966595	Sdhb	+
9	106463589	106463594	Pcbp4	+
4	123350283	123350288	Macf1	-
18	20032255	20032260	Dsc2	-
5	33654105	33654110	Tmem129	-
9	108339467	108339472	Gpx1	+
6	32792470	32792475	Chchd3	-
6	29959075	29959080	Strip2	+
9	116040907	116040912	Gm9385	-
4	119419085	119419090	Ppcs	-
2	164832837	164832842	Neurl2	-
6	32792696	32792701	Chchd3	-
13	107414232	107414237	Apoo-ps	-
19	9830954	9830959	AC132253.9	-
13	34978682	34978687	Eci2	-
8	13884908	13884913	Coprs	-
2	75640826	75640831	Rps6-ps4	+
4	14811053	14811058	Otud6b	-
15	98131438	98131443	Pfkm	+
7	67231653	67231658	Mef2a	-
11	59185954	59185959	Guk1	-
6	148232806	148232811	Tmtc1	-
17	46128657	46128662	Mrps18a	+
19	57034943	57034948	Ablim1	-
11	95831122	95831127	Phospho1;Zf	+
8	94180231	94180236	Mt1	+
8	85260774	85260779	Vps35	-
5	115645983	115645988	Rab35	+
4	107907607	107907612	Cpt2	-
8	95864518	95864523	Got2	-
10	120224823	120224828	Tmbim4	+
9	106212964	106212969	Twf2	+

5	121372350	121372355	Trafd1	-
7	132562602	132562607	Oat;Fgfr2	-
17	44039066	44039071	Rcan2	+
7	116104300	116104305	1110004F10I	+
17	24436206	24436211	Eci1	+
6	131365253	131365258	Ybx3	-
15	27593549	27593554	Ank	+
5	117377415	117377420	Wsb2	+
2	174122152	174122157	Npepl1	+
9	44913351	44913356	Atp5l	-
1	185287182	185287187	Iars2	-
15	5120989	5120994	Gm10250	-
15	4154833	4154838	Oxct1	+
18	73639221	73639226	Smad4	-
16	4874807	4874812	Ubald1	-
11	100627582	100627587	Nkiras2	+
10	80256700	80256705	Ndufs7	+
11	102436509	102436514	Grn	+
9	107614547	107614552	Gnai2	-
11	30943057	30943062	Erlec1	-
13	12278795	12278800	Actn2	-
14	54470055	54470060	Lrp10	+
12	103417594	103417599	Ddx24	-
12	85132649	85132654	Dlst	+
13	119335896	119335901	Nnt;Nnt	-
6	142501414	142501419	Ldhb	-
4	147874858	147874863	Mfn2	-
17	24437493	24437498	Eci1	+
6	29461452	29461457	Flnc	+
11	106074099	106074104	Taco1	+
6	113515473	113515478	Emc3	-
15	88864864	88864869	Pim3	+
17	24164330	24164335	Atp6v0c	-
1	37897822	37897827	Mrpl30	+
7	139578424	139578429	Inpp5a	+
19	6908072	6908077	Prdx5	-
11	98770288	98770293	Nr1d1	-
9	101107222	101107227	Ppp2r3a	-
12	80950191	80950196	Srsf5	+
1	135850031	135850036	Tnnt2	+
8	110880917	110880922	Cog4	+
9	49440373	49440378	Ttc12	-
3	97690147	97690152	Pde4dip	-
12	30895664	30895669	Acp1	-
12	54859456	54859461	Cfl2	-

5	123512033	123512038	Diablo	-
4	94571801	94571806	Plaa	-
11	101294029	101294034	Becn1	-
9	118062196	118062201	Azi2	+
1	66838373	66838378	Acadl	-
16	23109158	23109163	Eif4a2	+
8	67514701	67514706	Gm9755	-
1	58994408	58994413	Stradb	+
13	62083113	62083118	Gm48228	-
6	82726477	82726482	Hk2	-
9	107300897	107300902	Cish	+
1	55071951	55071956	Coq10b	+
11	21562945	21562950	Mdh1	-
4	151048625	151048630	Vamp3;Camt	-
2	155634631	155634636	Trpc4ap	-
4	6398851	6398856	Nsmaf	-
7	44830609	44830614	Nup62;Il4i1	+
11	95389120	95389125	Slc35b1	+
8	34161342	34161347	Saraf	+
17	87433819	87433824	Calm2	-
5	72976076	72976081	Slain2	+
7	120120885	120120890	Tmem159	+
13	99948836	99948841	Mccc2	-
19	5844023	5844028	Neat1	-
14	51908380	51908385	Ndrg2	-
2	173701312	173701317	Rab22a	+
19	52995474	52995479	Xpnpep1	-
14	69703985	69703990	R3hcc1	-
9	25307598	25307603	Sept7	+
18	56575949	56575954	Phax	+
17	34216195	34216200	Tap2	+
11	46972719	46972724	Sgcd	-
18	5118500	5118505	Svil	+
8	94673966	94673971	Arl2bp	+
3	95662623	95662628	Mcl1	+
4	42958110	42958115	Dnajb5	+
16	57154173	57154178	Tomm70a	+
5	17828783	17828788	Cd36	-
4	147874558	147874563	Mfn2	-
3	32554160	32554165	Mfn1	+
8	72581213	72581218	Tmem38a	+
6	83056993	83056998	Aup1	+
10	120217567	120217572	Tmbim4	+
12	31331588	31331593	Dld	-
8	121549797	121549802	Fbxo31	-

9	108681998	108682003	Slc25a20	+
7	143062579	143062584	Cd81	+
6	127125166	127125171	Ccnd2	-
12	16985152	16985157	Rock2	+
11	117813586	117813591	Syngr2;Gm2	+
8	40990951	40990956	Mtus1	-
12	85343248	85343253	Tmed10	-
8	11199272	11199277	Col4a1	-
5	24581484	24581489	Abcf2	-
3	145879035	145879040	Rpl36a-ps2;[	+
4	42983483	42983488	Vcp	-
10	121397054	121397059	Gns	+
16	87483467	87483472	Usp16	+
8	85539129	85539134	Dnaja2	-
8	93972929	93972934	Amfr	-
3	37714811	37714816	Rps23-ps1	+
10	75772738	75772743	Ddt;Gm2044	-
11	74669626	74669631	Cluh	+
10	127022908	127022913	Tsfm	-
5	31141531	31141536	Mpv17	-
12	110858134	110858139	Wdr20	-
8	114151884	114151889	Nudt7	+
8	11448953	11448958	Col4a2	+
6	99877863	99877868	Tpt1-ps3	-
8	120081490	120081495	Zdhhc7	-
18	44346013	44346018	Myot	+
1	171238296	171238301	Ndufs2	-
17	25782692	25782697	Narfl	+
15	57871350	57871355	Derl1	-
2	119605875	119605880	1700020I14F	+
7	19565274	19565279	Gemin7	-
7	90457143	90457148	Tmem126a	-
19	5100533	5100538	Rab1b	-
18	34938949	34938954	Hspa9	-
11	93959307	93959312	Gm20390;Nr	-
9	50635777	50635782	Dlat	-
9	120013975	120013980	Xirp1;Cx3cr1	-
15	98934345	98934350	Tuba1b;Gm4	-
14	63142434	63142439	Ctsb	+
17	83502665	83502670	Cox7a2l	-
8	111768502	111768507	Cfdp1	-
8	109675740	109675745	Ist1	-
13	113663033	113663038	Hspb3	-
2	70023082	70023087	Ubr3	+
1	45909268	45909273	Slc40a1	-

19	5707957	5707962	Ehbp1l1	-
17	73243247	73243252	Lclat1	+
2	167608675	167608680	Ube2v1;Gm2	-
2	61722749	61722754	Psmd14	+
13	38198328	38198333	Dsp	+
4	127247098	127247103	Smim12	+
16	95720570	95720575	Ets2	+
16	36964366	36964371	Fbxo40	-
6	72370195	72370200	Vamp5	-
17	10206985	10206990	Qk	-
7	111071162	111071167	Eif4g2	-
6	142490462	142490467	Ldhb	-
7	43454559	43454564	Etfb;Gm4523	+
14	61219386	61219391	Sgcg	-
5	21740514	21740519	Pmpcb	+
2	177478537	177478542	Zfp970	+
5	138163809	138163814	Cops6	+
15	76345685	76345690	Cyc1	+
14	55581660	55581665	Emc9	-
6	86737109	86737114	Anxa4	-
10	88474853	88474858	Chpt1	-
2	125831948	125831953	Cops2	-
4	119418768	119418773	Ppcs	-
17	56751351	56751356	Nrtn	-
13	14625521	14625526	Psma2	+
9	77659704	77659709	Klhl31	+
6	119925066	119925071	Wnk1	-
3	88568847	88568852	Ubqln4	+
5	140760111	140760116	Gna12	-
17	8297252	8297257	Mpc1	+
1	9557019	9557024	Adhfe1	+
14	54957398	54957403	Myh6	-
3	10204480	10204485	Fabp4	-
17	25185118	25185123	BC003965	+
6	55348159	55348164	Aqp1	+
2	121458275	121458280	Serf2;Hypk	+
7	103826661	103826666	Hbb-bs	-
7	100326797	100326802	Ppme1	-
4	139649028	139649033	Gm21969;Al	+
19	5843115	5843120	Neat1	-
11	6355424	6355429	Ogdh	+
4	127027305	127027310	Sfpq	+
14	66108068	66108073	Ephx2	-
6	32792460	32792465	Chchd3	-
13	74172236	74172241	Exoc3	-

14	76506799	76506804	Tsc22d1	+
19	5707995	5708000	Ehbp1l1	-
10	79977585	79977590	Tmem259	-
13	64186164	64186169	Habp4	+
8	105280008	105280013	Nol3	+
19	44139627	44139632	Bloc1s2	-
3	89415689	89415694	Cks1b	-
19	40293276	40293281	Pdlim1	-
11	106781743	106781748	Ddx5	-
6	113734649	113734654	Sec13	-
5	93182660	93182665	Ccni	-
9	25286716	25286721	Sept7	+
19	53588857	53588862	Nutf2-ps1	-
16	17281062	17281067	Pi4ka	-
13	74359796	74359801	Lrrc14b	-
14	79056646	79056651	Vwa8	+
9	59679217	59679222	Pkm	+
10	128048270	128048275	Naca	+
17	44037785	44037790	Rcan2	+
5	77349320	77349325	Igfbp7	-
6	5483474	5483479	Pdk4	-
4	117675126	117675131	Dmap1	-
17	46775586	46775591	Rpl7l1	-
11	96819054	96819059	Nfe2l1	-
3	97701593	97701598	Pde4dip	-
8	124837227	124837232	2810004N23	-
9	55485293	55485298	Etfa	-
8	11448749	11448754	Col4a2	+
14	21845511	21845516	Vdac2	+
8	94854417	94854422	Coq9	+
1	55083770	55083775	Hspd1	-
1	78462878	78462883	Farsb	-
3	89271982	89271987	Efna1	-
2	181856725	181856730	Pcmtd2	+
9	55223273	55223278	Fbxo22	+
6	73263964	73263969	Suclg1	+
6	24603471	24603476	Lmod2	+
13	12270896	12270901	Actn2	-
17	39846659	39846664	Tns1	+
7	138891856	138891861	Bnip3	-
7	84110517	84110522	Abhd17c	-
11	4091685	4091690	Mtfp1	-
4	126232536	126232541	Map7d1	-
17	56259506	56259511	Fem1a	+
3	88729326	88729331	Rit1	+

15	74959001	74959006	Ly6e	+
11	102404518	102404523	Slc25a39	-
5	135735164	135735169	Por	+
13	115088759	115088764	Pelo;Itga1;Gr	-
5	140758749	140758754	Gna12	-
15	103244406	103244411	Hnrnpa1	+
11	120560425	120560430	P4hb	-
17	53671925	53671930	Kat2b	+
13	63302741	63302746	2010111I01F	+
2	25271521	25271526	Ssna1	-
1	161241405	161241410	Prdx6	-
8	85075259	85075264	Wdr83	-
17	46549471	46549476	Srf	-
15	81911943	81911948	Aco2	+
7	116825507	116825512	Gm4366	-
10	17844732	17844737	Txlnb	+
11	94972311	94972316	Sgca	-
15	4153923	4153928	Oxct1	+
16	23113824	23113829	Eif4a2	+
19	7578999	7579004	Pla2g16	+
11	29154163	29154168	Pnpt1	+
10	56390004	56390009	Gja1	+
11	69990034	69990039	Ctdnep1	+
3	138455291	138455296	Adh5	+
4	109098530	109098535	Osbpl9	-
2	76705103	76705108	Ttn	-
8	85538535	85538540	Dnaja2	-
10	61702697	61702702	Tysnd1	+
11	30099574	30099579	Sptbn1	-
19	9119261	9119266	Asrgl1	-
6	124811376	124811381	Tpi1	-
4	135214641	135214646	Clic4	-
18	67429163	67429168	Afg3l2	-
10	81565632	81565637	Aes	+
8	13253898	13253903	Adprhl1	-
11	78522041	78522046	Poldip2	+
17	71252350	71252355	Emilin2	-
7	19090943	19090948	Dmpk	+
5	121207169	121207174	Rpl6	+
19	9829041	9829046	AC132253.9	-
8	105856392	105856397	Thap11	+
6	24603761	24603766	Lmod2	+
8	46535255	46535260	Acsl1	+
17	87435142	87435147	Calm2	-
10	115290440	115290445	Rab21	-

12	108107193	108107198	Setd3	-
11	58995898	58995903	Obscn	-
11	69554566	69554571	Efnb3	-
12	44311877	44311882	Pnpla8	+
19	6390369	6390374	Pygm	+
9	53582017	53582022	Acat1	-
2	90898680	90898685	Ndufs3	-
11	57806153	57806158	Sap30l	+
12	3248689	3248694	Rab10	-
9	54715949	54715954	Dnaja4	+
3	101582794	101582799	Atp1a1	-
18	36798055	36798060	Zmat2	+
5	100547645	100547650	Cops4	+
11	109669456	109669461	Prkar1a	+
12	9035923	9035928	Ttc32	+
7	19081931	19081936	Dmwd	+
7	31054376	31054381	Fxyd1	-
4	156219860	156219865	Perm1	+
10	24598335	24598340	Ccn2	+
5	92202039	92202044	Uso1	+
4	45107996	45108001	Tomm5	-
17	46147864	46147869	Mad2l1bp	-
4	120530047	120530052	Scmh1	+
16	20125200	20125205	Klhl24	+
11	49678706	49678711	Gm12191;Cr	-
8	33783059	33783064	Rbpms	-
13	115088684	115088689	Pelo;Itga1;Gr	-
19	37221662	37221667	March5	+
5	121531646	121531651	Adam1a;Gm	-
13	38197193	38197198	Dsp	+
3	61008896	61008901	P2ry1	+
14	55561261	55561266	Dcaf11	+
11	106200476	106200481	Ccdc47;Stra	-
1	175606147	175606152	Fh1	-
8	85075272	85075277	Wdr83	-
4	135214031	135214036	Clic4	-
2	173778757	173778762	Vapb	+
2	32287940	32287945	Swi5	-
9	107538013	107538018	Tmem115	+
6	90645916	90645921	Slc41a3	+
9	119485594	119485599	Scn5a	-
7	138836054	138836059	Mapk1ip1	-
4	126275506	126275511	Trappc3	+
5	20759102	20759107	Phtf2	-
8	45827829	45827834	Sorbs2	+

5	58132478	58132483	Pcdh7	+
3	79813823	79813828	Tmem144	-
8	111626346	111626351	Ldhd	-
3	132660533	132660538	Aimp1	-
18	76965126	76965131	Hdhd2;Hdhd:	+
8	128722581	128722586	Itgb1	+
7	105558689	105558694	Apbb1	-
4	107923438	107923443	Cpt2	-
12	110898139	110898144	Rps19-ps6;T	+
17	46548783	46548788	Srf	-
3	135458863	135458868	Ube2d3	+
19	6134627	6134632	Arl2	-
4	141618935	141618940	Slc25a34	-
7	23947015	23947020	Gm10175	-
11	29154886	29154891	Pnpt1	+
2	152143930	152143935	Tcf15	+
5	124550407	124550412	Tmed2	+
12	84306031	84306036	Ptgr2	+
1	90215647	90215652	Ackr3	+
13	23740027	23740032	Hist1h1c	+
1	181242129	181242134	Rpl35a-ps2	-
4	151981055	151981060	Dnajc11	+
7	45917127	45917132	Tmem143	+
4	57371551	57371556	Gm12537	-
1	181904053	181904058	Enah	-
5	69516898	69516903	Yipf7	-
12	81500126	81500131	Gm20498;Sy	-
9	86582804	86582809	Me1	-
9	50596427	50596432	Sdhd	-
7	126490320	126490325	Tufm	+
10	13515383	13515388	Fuca2	+
6	57689535	57689540	Gm26712;Py	-
7	47053854	47053859	Tmem86a	+
19	45752007	45752012	Mgea5	-
1	135301821	135301826	Timm17a	-
11	62552851	62552856	Ubb	+
1	164437746	164437751	Atp1b1	-
15	89131041	89131046	Mapk12	-
6	86369804	86369809	Fam136a	+
15	79721433	79721438	Gtpbp1	+
2	114049571	114049576	Actc1	-
15	79030320	79030325	H1f0	+
15	76329660	76329665	Exosc4	+
6	122457073	122457078	Gm8430	+
18	6203674	6203679	Kif5b	-

11	96819429	96819434	Nfe2l1	-
11	59012399	59012404	Obscn	-
4	116033922	116033927	Nsun4	-
8	95864336	95864341	Got2	-
15	77962559	77962564	Eif3d	-
12	102744826	102744831	Tmem251	+
11	120646175	120646180	Myadml2	-
9	107592823	107592828	Ifrd2	+
17	71126544	71126549	Myom1;Gm2	+
2	121548460	121548465	Frmd5	-
17	57235086	57235091	Gpr108	-
11	95679821	95679826	Phb	+
8	105472515	105472520	Zdhhc1	-
9	75057158	75057163	Arpp19	+
19	40624586	40624591	Entpd1	+
11	77470284	77470289	Coro6	+
1	74280124	74280129	Aamp	-
2	131937443	131937448	Prnp;Prn	+
17	34969866	34969871	Hspa1a	-
10	81367217	81367222	Fzr1	-
14	19819948	19819953	Rtraf	-
13	24813691	24813696	BC005537	+
15	31598004	31598009	Cct5	-
11	59876184	59876189	Nt5m	+
2	180162188	180162193	Osbpl2	+
9	50344319	50344324	Rpl10-ps3	-
5	124549634	124549639	Tmed2	+
7	126547612	126547617	Eif3c	-
3	65380174	65380179	Ssr3	-
8	91667404	91667409	Fto	+
12	84382353	84382358	Entpd5	-
15	89420783	89420788	Gm44502;Cp	-
9	64177741	64177746	Rpl4	+
1	58591087	58591092	Ndufb3	+
13	58126189	58126194	Hnrnpa0	-
17	66500338	66500343	Rab12	-
4	132186841	132186846	Ythdf2	-
11	115607252	115607257	Mrps7	+
6	125130457	125130462	Chd4	+
10	121412448	121412453	Rassf3	-
18	63664265	63664270	Txnl1	-
10	128086104	128086109	Atp5b	+
4	33021145	33021150	Rragd	+
13	58381847	58381852	2210016F16I	-
14	34561616	34561621	Ldb3	-

17	26123792	26123797	Mrpl28	+
19	6390374	6390379	Pygm	+
11	40749694	40749699	Ccng1	-
5	122453730	122453735	Atp2a2	-
18	16627666	16627671	Cdh2	-
16	90342012	90342017	Gm49708	-
14	34344435	34344440	Glud1	+
2	32707681	32707686	Cdk9	-
18	64457914	64457919	Fech	-
11	120578906	120578911	Arhgdia	-
16	18301164	18301169	Tango2	-
1	161241364	161241369	Prdx6	-
18	31583565	31583570	Slc25a46	-
11	77470359	77470364	Coro6	+
12	25094357	25094362	Id2	-
1	24614505	24614510	Gm10925	-
18	25125845	25125850	Fhod3	+
5	129746422	129746427	Nipsnap2	+
18	68267194	68267199	Fam210a	-
6	31489340	31489345	Mkln1	+
19	46330637	46330642	Cuedc2	-
5	115348567	115348572	Cox6a1	-
10	128363343	128363348	Coq10a	-
2	132530159	132530164	Gpcpd1	-
11	46971648	46971653	Sgcd	-
1	180182163	180182168	Coq8a	-
4	21757554	21757559	Tstd3	-
10	77597437	77597442	Pttg1ip	+
12	54645521	54645526	Sptssa	-
18	16590223	16590228	Cdh2	-
18	25133026	25133031	Fhod3	+
11	87743371	87743376	Supt4a	+
5	129128463	129128468	Rps16-ps2	-
5	91625266	91625271	Parm1	+
15	74747950	74747955	Lynx1	-
12	100301070	100301075	Ttc7b	-
12	54861227	54861232	Cfl2	-
11	59013688	59013693	Obscn	-
8	85074303	85074308	Dhps	+
14	55569693	55569698	Dcaf11	+
17	27630434	27630439	Rps10;RPS1	-
12	44311430	44311435	Pnpla8	+
13	58392230	58392235	Hnrnpk	-
11	20335491	20335496	Gm12033	-
3	90235707	90235712	Jtb	+

5	113774303	113774308	Iscu	+
1	85850559	85850564	Cab39	+
15	103241459	103241464	Hnrnpa1	+
4	86657133	86657138	Plin2	-
2	30346446	30346451	Sh3glb2	-
16	64766143	64766148	4930453N24	-
5	129022979	129022984	Ran	+
2	91264365	91264370	Pacsin3	+
5	122169099	122169104	Ppp1cc	+
9	40804274	40804279	Hspa8	+
7	89918773	89918778	Hikeshi	-
11	60731617	60731622	Mief2	+
4	138313777	138313782	Pink1	-
17	23676581	23676586	Tnfrsf12a	-
14	13951210	13951215	Thoc7	-
17	70996762	70996767	Myl12a;Myl1:	-
2	94412165	94412170	Api5	-
9	108378547	108378552	Usp4	+
15	76171735	76171740	Plec	-
9	123791478	123791483	Fyco1	-
9	50596843	50596848	Sdhd	-
1	134075696	134075701	Btg2	-
13	30381188	30381193	Agtr1a	+
4	33246125	33246130	Pnrc1	-
15	76070344	76070349	Puf60	-
17	66079538	66079543	Ndufv2	-
8	12868477	12868482	Atp11a	+
12	56373794	56373799	Cox6c2	-
15	25970135	25970140	Retreg1	+
8	128732427	128732432	Itgb1	+
5	24441567	24441572	Fastk	-
6	87843806	87843811	Cnbp	-
12	32976593	32976598	Sypl	+
11	94125183	94125188	Spag9	+
4	45396661	45396666	Slc25a51	-
6	120818370	120818375	Atp6v1e1	-
2	163469482	163469487	Fitm2	-
6	67284097	67284102	Serbp1	+
12	71123530	71123535	Timm9	-
17	35957106	35957111	Abcf1	-
9	119349366	119349371	Acaa1a	+
5	143506041	143506046	Rac1	-
1	90215300	90215305	Ackr3	+
12	53150378	53150383	Akap6	+
12	4877833	4877838	Sf3b6	-

10	79709709	79709714	Bsg	+
2	158117460	158117465	Tgm2	-
3	19691691	19691696	Trim55	+
12	72794129	72794134	Ppm1a	+
2	155389760	155389765	Trp53inp2	+
1	159324944	159324949	Cop1	+
12	69296793	69296798	Klhdc2	+
2	114049190	114049195	Actc1	-
2	35301542	35301547	Gsn	+
11	58995466	58995471	Obscn	-
5	137310785	137310790	Trip6	-
10	81181244	81181249	Eef2	+
12	31934308	31934313	Hbp1	-
11	55005126	55005131	Anxa6	-
19	44554958	44554963	Ndufb8	-
18	77781856	77781861	Atp5a1	+
8	3153660	3153665	Insr	-
10	58422999	58423004	Lims1	+
1	131055027	131055032	Mapkapk2	-
13	32701402	32701407	Mylk4	-
3	96527257	96527262	Hfe2	+
8	45826155	45826160	Sorbs2	+
19	41214408	41214413	Tm9sf3	-
1	4846072	4846077	Lypla1;Gm37	+
19	53379758	53379763	Smndc1	-
9	66514466	66514471	Fbxl22	-
18	35590632	35590637	Matr3	+
11	63962837	63962842	Cox10	-
16	4477841	4477846	Usf3	-
5	121525139	121525144	Adam1a;Gm	-
4	91851837	91851842	Gm12671	-
8	13465742	13465747	Gas6	-
5	105272888	105272893	Gbp6;Gm43	-
10	105847213	105847218	Ccdc59	+
5	129845925	129845930	Cct6a	+
3	58102777	58102782	Pfn2	-
7	139579411	139579416	Inpp5a	+
16	4482783	4482788	Srl	-
15	93284562	93284567	Yaf2	-
4	134529393	134529398	Mtfr1l	-
11	50202551	50202556	Sqstm1	-
10	128352776	128352781	Cs	+
4	43663971	43663976	Hint2	+
11	97048627	97048632	Mrpl10	+
17	56259389	56259394	Fem1a	+

8	122614369	122614374	Trappc2l;Gm	+
9	107593513	107593518	Ifrd2	-
14	69717839	69717844	Chmp7	-
3	67475014	67475019	Gfm1	+
5	121567897	121567902	Aldh2	-
7	30641496	30641501	Rbm42;Gm2	-
13	91865350	91865355	Ckmt2	-
17	23827543	23827548	Elob	-
9	57923200	57923205	Ubl7	+
2	130681413	130681418	Itpa	+
3	95662286	95662291	Mcl1	+
9	56136636	56136641	Tspan3	-
4	106440991	106440996	Usp24	+
2	76705715	76705720	Ttn	-
8	12868130	12868135	Atp11a	+
1	36530419	36530424	Ankrd23;Gm	-
2	181152806	181152811	Eef1a2	-
16	33948877	33948882	Itgb5	+
1	92919864	92919869	Rnpepl1	+
1	155212988	155212993	BC034090	-
16	90341603	90341608	Gm49708	-
5	112760382	112760387	Myo18b	-
3	95036710	95036715	Psmd4	-
11	97328759	97328764	Mrpl45	+
6	71874538	71874543	Immt	+
5	116408761	116408766	Hspb8	-
7	19416818	19416823	Ckm	+
10	3366662	3366667	Ppp1r14c	+
6	125193361	125193366	Mrpl51	+
4	134323044	134323049	Trim63	+
12	103333462	103333467	Asb2	-
17	56258584	56258589	Fem1a	+
2	155848061	155848066	Uqcc1	-
18	68268341	68268346	Fam210a	-
19	23675975	23675980	Gm6563	+
8	93166226	93166231	Ces1d	-
5	115111130	115111135	Acads	-
6	13087815	13087820	Tmem106b	+
4	95050197	95050202	Jun	-
1	7172575	7172580	Pcmtd1	+
11	59212018	59212023	Arf1	-
7	114705995	114706000	Gm15500	-
15	76500877	76500882	Hsf1	+
6	50564544	50564549	Cycs	-
15	81914321	81914326	Aco2	+

16	91425653	91425658	Il10rb	+
17	26789699	26789704	Bnip1	+
10	75772867	75772872	Ddt;Gm2044	-
19	46657121	46657126	Wbp1l	+
16	91425657	91425662	Il10rb	+
17	35830763	35830768	Flot1	+
11	6355391	6355396	Ogdh	+
7	102111636	102111641	Art1	+
18	50091055	50091060	Tnfaip8	+
11	95026282	95026287	Pdk2	-
19	6062290	6062295	Znhit2	+
1	36530278	36530283	Ankrd23;Gm	-
14	49682339	49682344	Armh4	-
16	58470499	58470504	St3gal6	-
4	107904350	107904355	Cpt2	-
19	6951783	6951788	Bad	+
9	108340101	108340106	Gpx1	+
16	31456978	31456983	Bdh1	+
10	41907699	41907704	Sesn1	+
11	59013284	59013289	Obscn	-
18	84096185	84096190	Zadh2	+
10	80900736	80900741	Timm13	-
10	81208623	81208628	Atcayos	+

Supplemental Table 2 – Echocardiography Parameters

	Control Diet		Western Diet		2 Way ANOVA
**Echo**	**Control**	**M3KO**	**Control**	**M3KO**	**Multiple Comparisons**
**IVS;d (mm)**	1.101 ± 0.079	1.142 ± 0.059	0.986 ± 0.058	1.226 ± 0.057	ns
**IVS;s (mm)**	1.481 ± 0.124	1.613 ± 0.089	1.474 ± 0.060	1.747 ± 0.069	ns
**LVID;d (mm)**	3.912 ± 0.196	4.125 ± 0.117	3.988 ± 0.137	3.846 ± 0.069	ns
**LVID;s (mm)**	2.77 ± 0.148	2.929 ± 0.117	2.676 ± 0.137	2.647 ± 0.087	ns
**LVPW;d (mm)**	0.989 ± 0.090	0.892 ± 0.031	0.886 ± 0.066	0.994 ± 0.066	ns
**LVPW;s (mm)**	1.156 ± 0.095	1.187 ± 0.038	1.156 ± 0.051	1.209 ± 0.065	ns
**EF (%)**	56.554 ± 2.096	56.158 ± 2.111	62.007 ± 2.362	59.595 ± 2.019	ns
**FS (%)**	29.2 ± 1.361	29.108 ± 1.385	33.017 ± 1.604	31.254 ± 1.33	ns
**HR (bpm)**	506.667 ± 7.478	484.875 ± 15.293	464.800 ± 10.599	478.25 ± 15.356	ns

Supplemental Table 2: Echocardiography analysis after 12 weeks of western or control diet, shown as mean. *n* = (6; 8; 5; 8) for (CD Ctrl; CD M3KO; WD Ctrl; WD M3KO). Data shown as mean ± SEM. 2-way ANOVA with multiple comparisons test was used. IVS;d = Interventricular Septum Thickness; diastole. IVS;s = Interventricular Septum Thickness; systole. LVID;d = Left Ventricular Internal Diameter; diastole. LVID;s = Left Ventricular Internal Diameter; systole. LVPW;d = Left Ventricular Posterior Wall Thickness; diastole. LVPW;s = Left Ventricular Posterior Wall Thickness; systole. EF = Ejection Fraction. FS = Fractional Shortening. HR = Heart Rate.

Supplemental Table 3. Differentially Expressed Proteins of Interest
Label Free Mass Spectrometry was performed on METTL3 fl/fl hearts
after 2 weeks of control or western diet

Differentially expressed proteins are those with a
log2FC > 1 or < -1

Fold Change is Western Diet / Control Diet

60 Transcript/Protein pairs were conserved between
LC-MS/MS and Nanopore targets (Supplemental Table 1)

Accession	Gene ID	log2FC
O35071	Kif1c	4.906891
Q91W90	Txndc5	4.321928
Q3THF9	Coq10b	4.321928
Q8K1N1	Pnpla8	3.321928
Q99KX1	Mlf2	3.321928
Q9WUD1	Stub1	3.321928
Q91YQ3	Csdc2	3.321928
Q9D7H3	RtcA	3.321928
Q64010	Crk	2.321928
Q6A0D4	Rftn1	2.321928
Q91ZP3	Lpin1	2
P63024	Vamp3	2
Q9JIF0	Prmt1	2
Q8BVI4	Qdpr	1.807355
Q63932	Map2k2	1.736966
Q60575	Kif1b	1.584963
Q811I0	Atpaf1	1.584963
Q9QYI5	Dnajb2	1.584963
P62627	Dynlrb1	1.584963
Q60749	Khdrbs1	1.584963
Q9WUQ2	Preb	1.584963
Q924T2	Mrps2	1.584963
P61148	Fgf1	1.584963
Q99JW4	Lims1	1.584963
P53810	Pitpna	1.321928
Q9CYT6	Cap2	1.137504
P62192	Psmc1	1.115477
Q9R0P5	Dstn	1.115477
Q9QYA2	Tomm40	-1.321928
Q9DCH4	Eif3f	-1.321928
P62843	Rps15	-1.321928
Q99N89	Mrpl43	-1.321928

P51859	Hdgf	-1.584963
Q91VC9	Ghitm	-1.584963
O35114	Scarb2	-1.584963
P19253	Rpl13a	-1.584963
Q9D1B9	Mrpl28	-1.584963
Q91YE3	Egln1	-1.584963
P35282	Rab21	-1.584963
Q3V009	Tmed1	-1.584963
Q3UHD3	Mtus2	-1.584963
Q99KI3	Emc3	-1.584963
O08715	Akap1	-1.807355
O70252	Hmox2	-2
Q99PL6	Ubxn6	-2
Q9CQN7	Mrpl41	-2
P62751	Rpl23a	-2.584963
P08122	Col4a2	-3.321928
Q64152	Btf3	-3.321928
Q9CQL4	Mrpl20	-3.321928
P55302	Lrpap1	-3.321928
Q3UJD6	Usp19	-3.321928
Q9JI46	Nudt3	-3.321928
Q8K3K7	Agpat2	-3.321928
P01901	H2-K1	-4.321928
P62849	Rps24	-4.321928
Q9D1M0	Sec13	-4.321928
Q5I043	Usp28	-4.321928
Q6DFX2	Antxr2	-4.321928
Q99J95	Cdk9	-4.906891
