# Supplementary material for: The cardiac METTL3/m6A pathway regulates the systemic response to Western diet
Source: JCI Insight. 2025 Apr 24;10(11):e188414. doi: 10.1172/jci.insight.188414 (PMC12220958; doi:10.1172/jci.insight.188414)

Figure 1A

METTL3

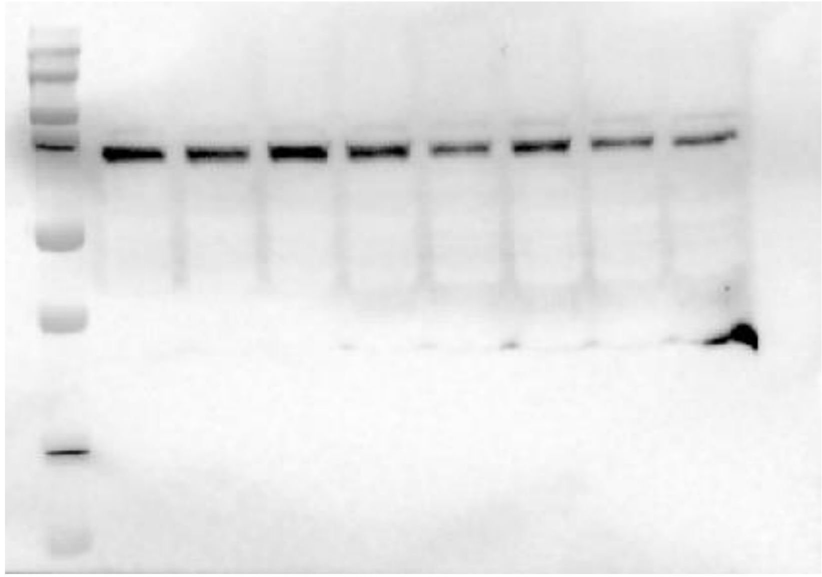

Ponceau

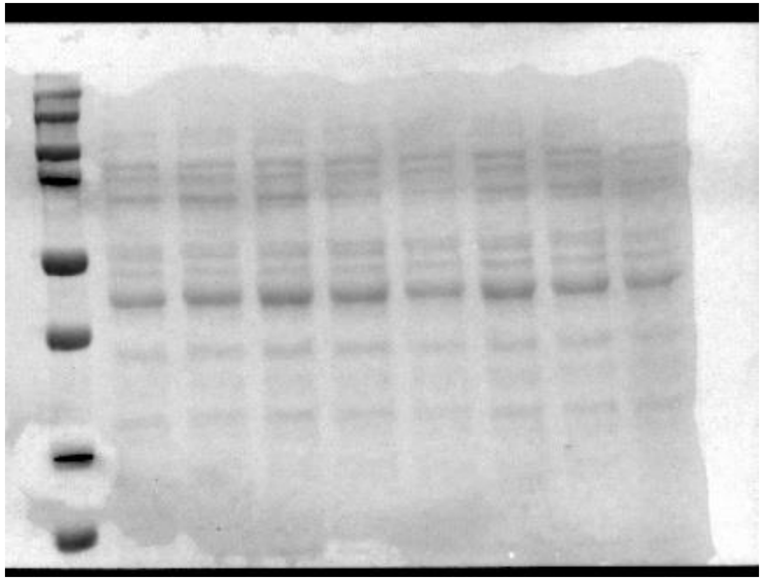

Vinculin

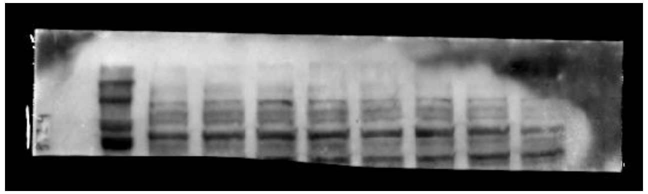

Figure 6K

METTL3

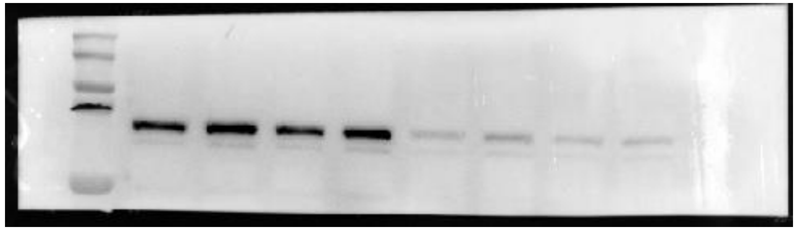

Ponceau

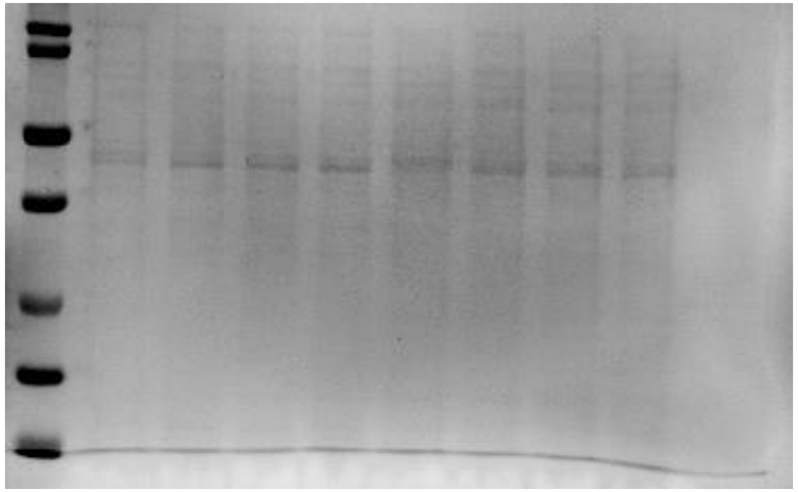

FGF1

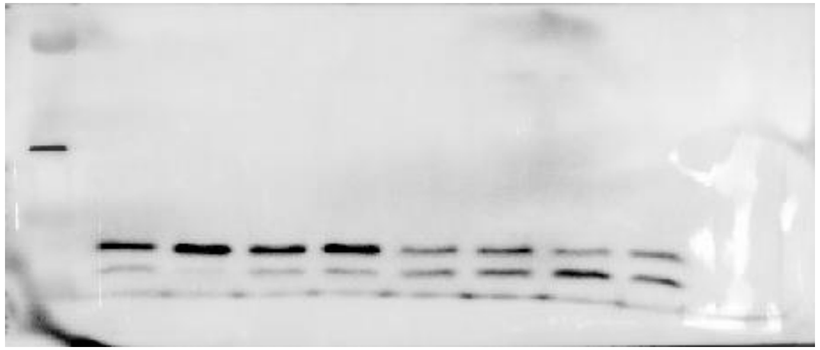

Beta Actin

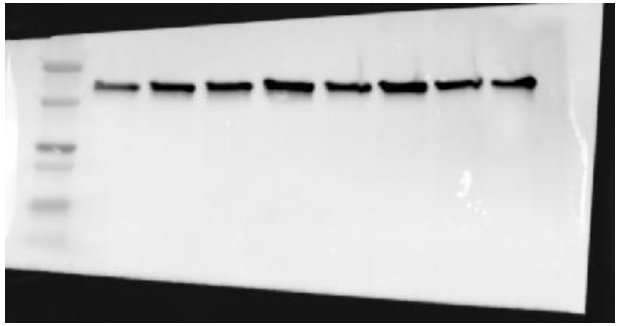

Figure 7B

Ponceau

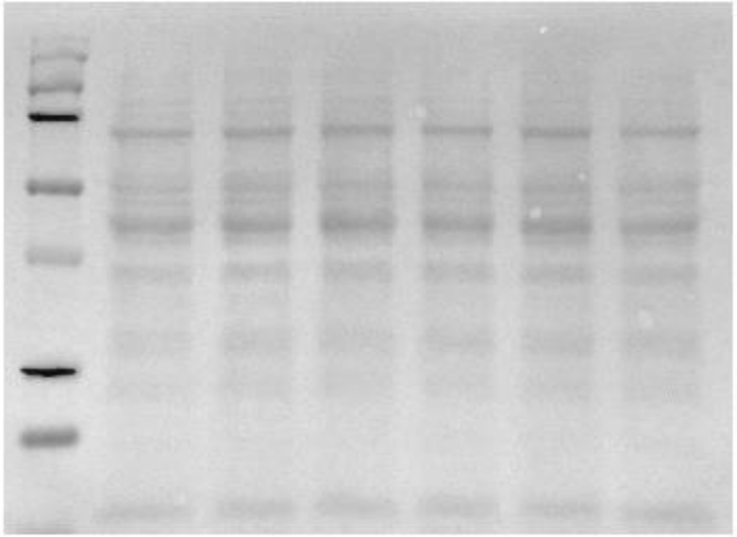

FGF1

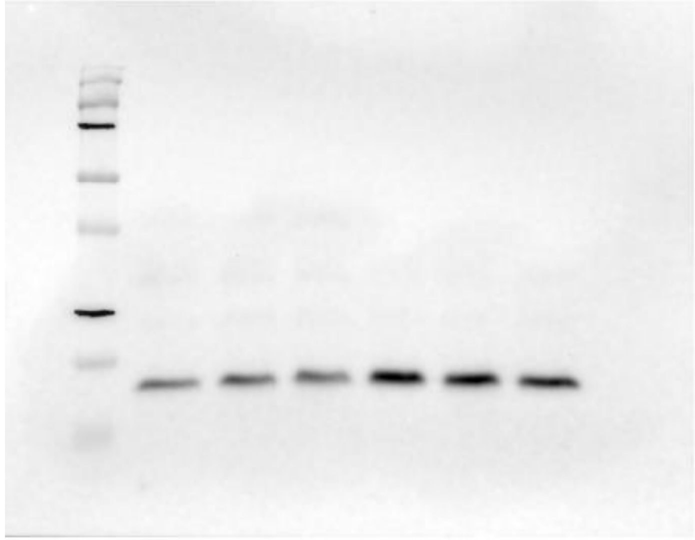

Vinculin

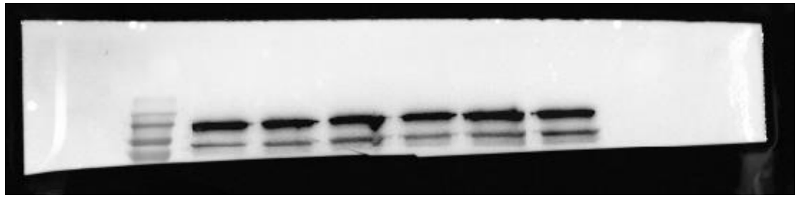

Supplemental Figure 2

Ponceau

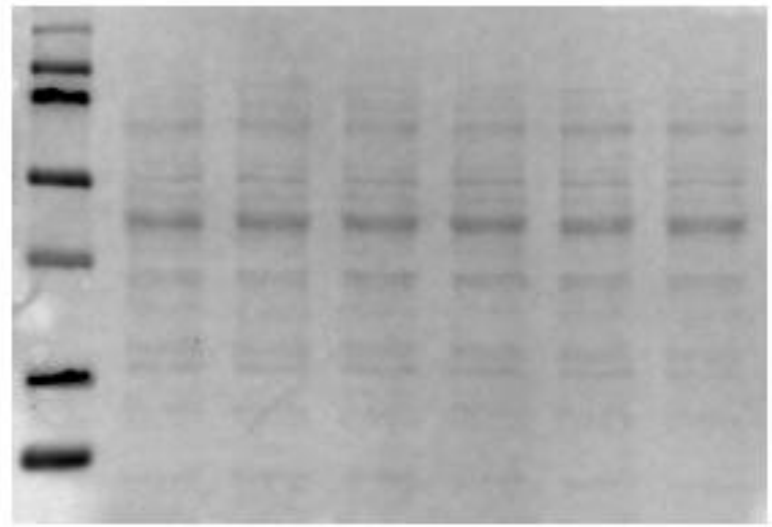

FGF1

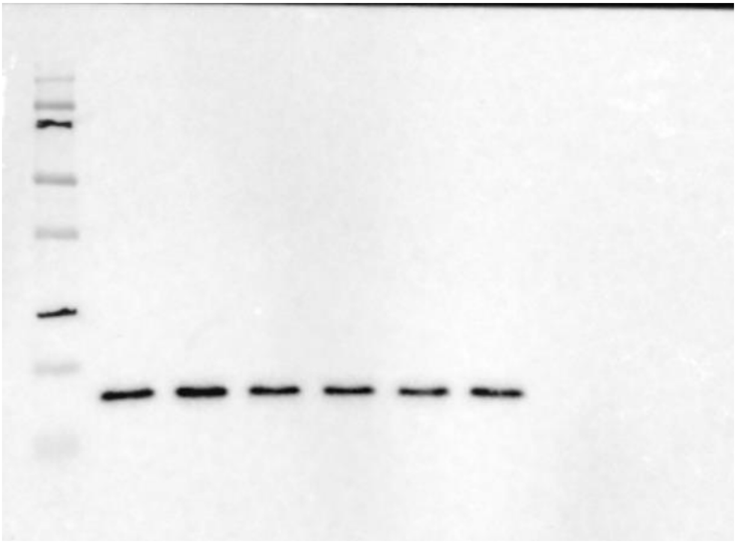

Supplement: Unedited blot and gel images [file jciinsight-10-188414-s053.pdf]
